# Fine mapping and discovery of candidate genes for seed size in watermelon by genome survey sequencing

**DOI:** 10.1038/s41598-018-36104-w

**Published:** 2018-12-14

**Authors:** Na Li, Jianli Shang, Jiming Wang, Dan Zhou, Nannan Li, Shuangwu Ma

**Affiliations:** Zhengzhou Fruit Research Institute, Chinese Academy of Agricultural Sciences, The Laboratory of Melon Crops, Zhengzhou, Henan Province 450009 China

## Abstract

Fine mapping and discovery of candidate genes underlying seed size are important for modern watermelon breeding. Here, by using a high-resolution genetic map and whole-genome genetic variation detection aided by genome survey sequencing, we fine mapped and discovered candidate genes for seed size in watermelon. QTL (quantitative trait locus) mapping identified two pleiotropic QTLs for seed size, namely, *qSS4* and *qSS*6, using a high-density genetic map constructed by specific length amplified fragment sequencing. *qSS6* explained 93.00%, 94.11% and 95.26% of the phenotypic variation in thousand-seed weight, seed length and seed width, respectively, and was defined as a major QTL. Then, high-coverage re-sequencing of two parental lines detected a total of 193,395 SNPs (single nucleotide polymorphisms) and 45,065 indels (insertions/deletions), which corresponded to a frequency of 534 SNPs/Mb and 124 indels/Mb. Based on the genetic variation in the two parental lines, newly developed PCR-based markers allowed the region of *qSS6* to be narrowed to 55.5 kb. Three potential candidates were identified, including a known seed size regulator in rice, *SRS3*. Taken together, our results reveal successful rapid fine mapping and discovery of candidate genes for seed size in watermelon, which could be applied to many traits of interest in plants.

## Introduction

In most plants, the seed links the start and end of the life cycle, and is thus of particular importance. Seeds are formed by the coordinated growth of the maternal sporophytic and zygotic tissues^1^. Hundreds of genes have been identified that influence seed size by parent-of-origin effects, acting in the maternal and/or zygotic tissues, and have been discussed in several excellent reviews^2–6^. The functions and discovery methods of several seed-size regulators are similar in *Arabidopsis* and rice; therefore, converting basic research on seed size into practical applications in crops and horticultural plants, such as watermelon [*Citrullus lanatus* (Thunb.) Matsum. & Nakai var. *lanatus*], is promising.

Seed size is a heritable and important trait in watermelon, with small seeds preferred in fruit sold for consumption but large seeds preferred for planting and edible seeds. Therefore, revealing genes/QTLs (quantitative trait loci) of seed size is valuable for breeding watermelon cultivars with the desired seed size. Seed size in watermelon is highly diverse and can be classified into six representative groups as follows: giant seed, big seed, medium seed, small seed, micro seed and tomato seed^7^. The Mendelian inheritance of seed size in watermelon has been determined. Early research showed that the light-weight phenotype was monogenic dominant over the heavy-weight in watermelon^8^. Two recessive genes, *s* and *l*, determine the long and short seeds, respectively, and *s* is epistatic to *l*^9^. Results have shown that seed size is genetically controlled, as medium-sized seeds were dominant over both short and long seeds; thus, *L-S-* produces medium-sized seeds, *llS-* produces long seeds, and *–ss* produces short seeds. The monohybrid inheritance of medium over long for seed size was confirmed by later studies^10,11^. Moreover, the dominant genes *Ti* for tiny seed^12^ and *ts* gene for tomato seed were also described^13,14^, which seems contradictory to the previous findings.

Advances in molecular marker and sequencing technologies have made it possible to locate genetic markers linked to traits of interest. Several random amplified polymorphism DNA loci were identified to be loosely linked to SL (seed length) and SW (seed width)^15^. Prothro *et al*. identified 13 main-effect QTLs (M-QTLs) on four linkage groups for seed weight, SW and SL^16^. Of these, major M-QTLs were identified at the same location in both populations for all three traits in an overlapping region between 5.80 Mb and 6.41 Mb on chromosome 6^17^. A major QTL for seed weight^18^ mapped in the same chromosomal region on chromosome 6 was described previously^16,17^, which suggested that this region is associated with seed size in watermelon from diverse genetic backgrounds. Another QTL for SL was detected by Meru and McGregor^18^. One QTL analysis for the medium seed and tomato seed phenotypes indicated a major QTL on chromosome 2^19^. These current SSR (microsatellite/simple sequence repeat)/SNP (single nucleotide polymorphism) /CAPS (cleaved amplified polymorphic sequence)-based genetic maps are of high quality and accuracy but lack the marker density required to build high-resolution, integrated genetic and physical maps; thus, none of these QTLs have been fine mapped, not to mention the genes underlying them.

The advent of next-generation sequencing revolutionized genomic approaches to biology. These new sequencing tools are also valuable for high-density genetic map construction^20,21^, genome-wide genetic marker discovery and genotyping^22^, and so on. Previous research conducted in our laboratory constructed a high-density genetic map based on an F_2_ population derived from two watermelon cultivars with significant differences in several fruit and seed traits using low-coverage sequencing^20^. In the present study, using a high-resolution genetic map and whole-genome genetic variation detection aided by genome survey sequencing, we fine mapped and discovered candidate genes for seed size in watermelon. Specifically, the main objectives of this study were as follows: (1) genome-wide QTL mapping of seed size traits (including TSW (thousand-seed weight), SL, SW and ST (seed thickness)) was performed using linkage analysis; (2) genome-wide genetic variation in two parental lines was detected using high-coverage re-sequencing; (3) the major QTLs for seed size were fine mapped using newly developed PCR (polymerase chain reaction)-based markers in two populations; and (4) potential candidate genes were analysed.

## Results

### QTL analysis of seed size

#### Phenotypic variation in the parents and segregating population

**Figure 1:**
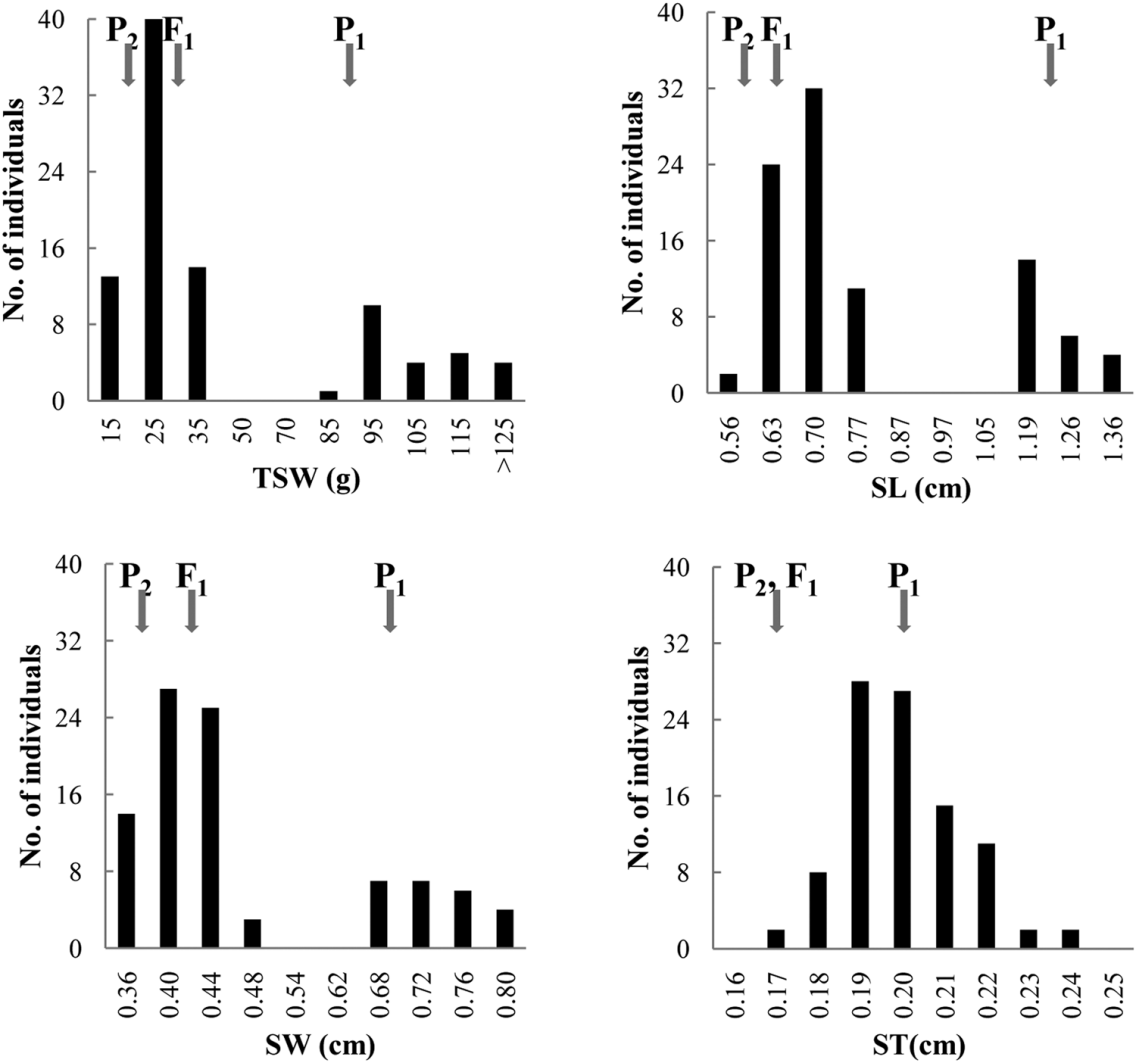
Frequency distribution of thousand-seed weight, seed length, seed width and seed thickness in the F_2_ population. P_1_ and P_2_ indicate ZXG01478 (the female parent) and 14CB11 (the male parent), respectively.

The female parent, ZXG01478, had large seeds with a TSW of 93.45 ± 9.60 g, an SL of 1.22 ± 0.03 cm, an SW of 0.70 ± 0.03 cm, and an ST of 0.20 ± 0.01 cm, whereas the male parent, 14CB11, had small seeds with a TSW of 20.80 ± 2.33 g, an SL of 0.62 ± 0.03 cm, an SW of 0.39 ± 0.01 cm, and an ST of 0.17 ± 0.01 cm (Supplementary Fig 1). Transgressive segregation was observed for all traits, indicating the presence of favourable alleles in both parents. The F_1_ generation gave rise to small seeds with a TSW of 30.13 ± 0.98 g, an SL of 0.68 ± 0.03 cm, an SW of 0.42 cm ± 0.01 cm, and an ST of 0.17 ± 0.01 cm, suggesting that the production of small seeds was dominant. In the F_2_ population, bimodal distributions for TSW, SL and SW were observed in Fig. 1, suggesting the existence of major genes for the three traits. The frequency distribution of ST in the F_2_ population was normal, suggesting a quantitative inheritance pattern. Correlations among TSW, SL and SW (Supplementary Table 1) were statistically significant and very strong (average r = 0.99). In contrast, the correlations between ST and the other three seed traits were also significant but somewhat lower, with an average r of 0.78 (p < 0.001).

#### QTL mapping of seed size

**Figure 2:**
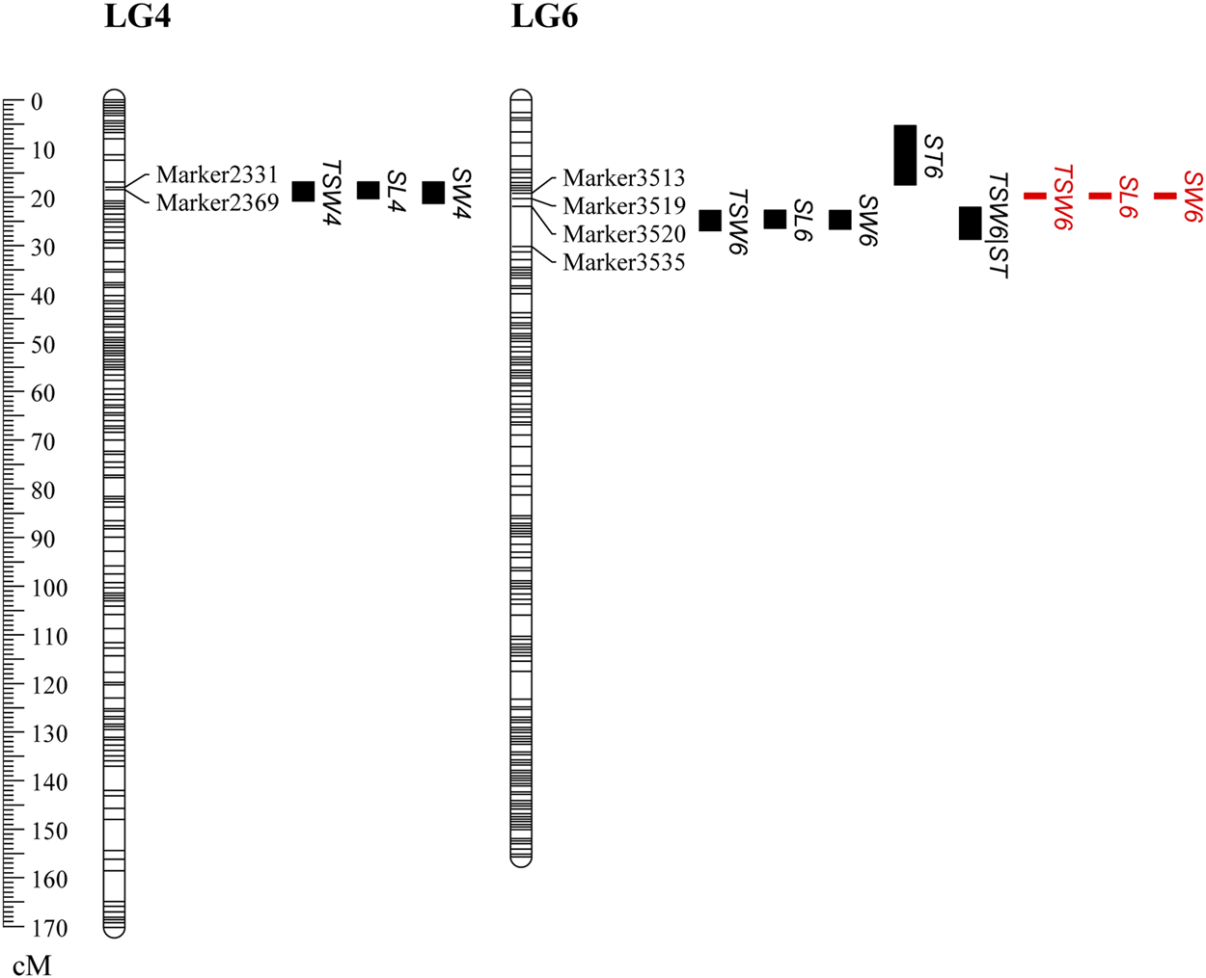
QTL scanning of thousand-seed weight, seed length, seed width and seed thickness. TSW, SL, SW and ST indicate thousand-seed weight, seed length, seed width and seed thickness, respectively. Black and red represents the results from CIM and GCIM, respectively.

A high-density genetic map containing 2,634 SNP markers was previously constructed in our laboratory^20^. The map covered a total of 1,906.31 cM (centimorgans) of the watermelon genome and had an average distance between adjacent markers of 0.72 cM^20^. Genome-wide QTL scanning detected seven significant QTLs associated with TSW, SW, SL and ST (Fig. 2, Table 1). Two significant QTLs for TSW were detected on LG4 and LG6. Of these QTLs, *TSW4* showed a peak LOD (logarithm of odds) score of 4.23 and explained 8.87% of the phenotypic variation, with the elite allele from the male parent, 14CB11, increasing the TSW by 11.99 g. *TSW6* showed a peak LOD score of 46.92 and explained 93.00% of the phenotypic variation, with the elite allele from the female parent, ZXG01478, increasing the TSW by 42.74 g. For SL, *SL4* showed a peak LOD score of 4.41 and explained 8.73% of the phenotypic variation, with the elite allele from the male parent, 14CB11, increasing the SL by 0.06 cm. Another QTL, *SL6*, showed a peak LOD score of 60.24 and explained 94.11% of the phenotypic variation, with the elite allele from the female parent, ZXG01478, increasing the SL by 0.30 cm. For SW, *SW4* showed a peak LOD score of 4.78 and explained 10.03% of the phenotypic variation, with the elite allele from the male parent, 14CB11, increasing the SW by 0.05 cm. Another QTL, *SW6*, showed a peak LOD score of 48.68 and explained 95.26% of the phenotypic variation, with the elite allele from the female parent, ZXG01478, increasing the SW by 0.18 cm. Only one significant QTL for ST was identified. *ST6* showed a peak LOD score of 18.05 and explained 37.92% of the phenotypic variation, with the elite allele from the female parent, ZXG01478, increasing the ST by 0.01 cm. These QTLs on LG6 for TSW, SL and SW were further confirmed by the GCIM (genome-wide composite interval mapping) method (Table 1, Fig. 2). Note that this region wasn’t associated with ST.

**Table 1:** QTLs identified by CIM and GCIM analysis.

Description	Unique QTL	Identified QTL	Trait^a^	LGs	CIM						GCIM					
					Peak position (cM)	LOD	R^2^	Flanking markers	Additive effect	Dominant effect	Peak position (cM)	LOD	R^2^	Flanking markers	Additive effect	Dominant effect
**Original linkage map**	*qSS4*	*TSW4*	TSW	LG4	18.01	4.23	8.87%	Marker7734-Marker2376	−11.99	13.44						
		*SL4*	SL	LG4	18.01	4.41	8.73%	Marker7734-Marker2376	−0.06	0.12						
		*SW4*	SW	LG4	18.01	4.78	10.03%	Marker7734-Marker7737	−0.05	0.06						
	*qSS6*	*TSW6*	TSW	LG6	24.06	46.9	93.00%	Marker3519-Marker3535	42.8	35.28	19.57	32.6	72.80%	Marker3513-Marker3519	39.26	27.57
		*SL6*	SL	LG6	24.06	60.24	94.11%	Marker3519-Marker3535	0.3	0.23	19.57	29.6	63.12%	Marker3513-Marker3519	0.26	0.14
		*SW6*	SW	LG6	24.06	48.68	95.26%	Marker3519-Marker3535	0.18	0.13	19.57	24.6	49.54%	Marker3513-Marker3519	0.14	0.05
	*qST6*	*ST6*	ST	LG6	15.86	18.05	37.92%	Marker3469-Marker3495	0.01	0.01						
		*TSW6|ST* ^*b*^		LG6	25.06	7.56	38.78%	Marker3519-Marker3535	15.64	17.40	19.57	3.95	7.46%	Marker3513-Marker3519	8.75	
**Re-constructed LG6**	*qSS6*	*TSW6*	TSW	LG6	28.91	66.2	88.64%	dcaps6_S6-indel14_S6	41.7	34.65	28.96	68.9	91.40%	dcaps9_S6	42.83	34.00
		*SL6*	SL	LG6	28.91	77.1	92.39%	dcaps6_S6-indel14_S6	0.3	0.22	28.96	71.7	79.05%	dcaps9_S6	0.29	0.18
		*SW6*	SW	LG6	28.91	68.5	94.10%	dcaps6_S6-indel14_S6	0.18	0.13	28.96	55.3	67.70%	dcaps9_S6	0.16	0.08
	*qST6*	*ST6*	ST	LG6	29.01	15.9	35.29%	caps1_S6-caps5_S6	0.01	0.01						

^a^TSW, SL, SW and ST indicate thousand-seed weight, seed length, seed width and seed thickness, respectively;

^b^*TSW6|ST* indicates trait TSW is conditioned by trait ST.

Interestingly, three and four QTLs were co-localized to regions on LG4 and LG6, respectively, suggesting the existence of multiple closely linked QTLs or one pleiotropic QTL for these traits. To make clear this issue, two approaches were adopted. One is to use multi-trait composite interval mapping to identify QTL for the four seed size related traits. As a result, the co-localized regions on LG4 and LG6 were found to be associated with TSW, SL, SW and SL (Table 2). Meanwhile, the lowest LOD score of the co-localized region on both LG4 and LG6 was found to be associated with TSW and ST. Another is to use conditional QTL mapping. Both *TSW4* and *TSW6* disappeared (the LOD score was lower than the significant threshold) when SL or SW was included as a covariate in the QTL scan. Therefore, co-localized QTLs for TSW, SL and SW on LG4 and LG6 were designated as unique QTLs: *qSS4* (“SS” was the abbreviation for “seed size”) and *qSS6*, respectively. However, when TSW was conditioned by ST, *TSW6|ST* was still significant (Fig. 2, Table 1), suggesting a genetic basis of *TSW6* that does not entirely overlap with that of *ST6*.

**Table 2:** Mapping QTLs for seed size related traits using multi-trait composite interval mapping method.

Trait^a^	LGs	Peak position (cM)	LOD	Flanking markers
TSW-SL	LG4	18.01	4.87	Marker7734-Marker2376
TSW-SW	LG4	18.01	5.35	Marker7734-Marker2376
TSW-ST	LG4	18.01	4.75	Marker7734-Marker2376
TSW-SL-SW	LG4	18.01	5.43	Marker7734-Marker2376
TSW-SL-SW-SL	LG4	18.01	5.59	Marker7734-Marker2376
TSW-SL	LG6	24.06	60.53	Marker3519-Marker3535
TSW-SW	LG6	24.06	53.58	Marker3519-Marker3535
TSW-ST	LG6	24.06	49.06	Marker3519-Marker3535
TSW-SL-SW	LG6	24.06	60.96	Marker3519-Marker3535
TSW-SL-SW-SL	LG6	24.06	62.05	Marker3519-Marker3535

^a^TSW, SL, SW and ST indicate thousand-seed weight, seed length, seed width and seed thickness, respectively.

### Mining of genome-wide genetic variations

#### Identification and genomic distribution of SNPs and indels (insertions/deletions)

**Figure 3:**
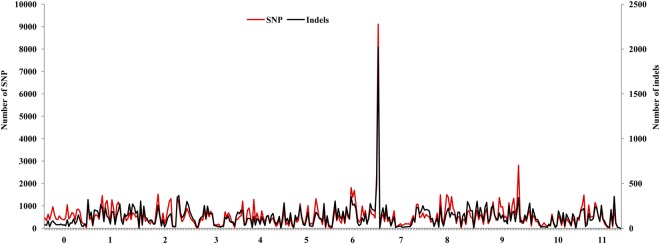
Genome-wide distribution of SNPs and indels in the chromosomes of watermelon. The horizontal axis shows the chromosomes, which are divided into 1-Mb intervals. Note that 0 was assembled but not anchored to the chromosome in the reference genome 97103.

The genomic distribution of SNPs and indels was investigated based on the chromosomes of reference genome 97103. A total of 193,395 SNPs and 45,065 indels were identified between ZXG01478 and 14CB11, corresponding to a frequency of 534 SNPs/Mb and 124 indels/Mb within an approximately 362-Mb physical distance. The distributions of SNPs and indels, which were generally similar, were not evenly distributed along each chromosome (Fig. 3). The frequencies of SNPs in the 1-Mb genomic intervals were significantly positively correlated with those of indels (Spearman correlation coefficient = 0.83, p < 0.0001). The normalized average occurrence of SNPs and indels varied across the chromosomes, falling within the range of approximately 260-1,002 SNPs/Mb and 80–231 indels/Mb, respectively. The polymorphism densities were higher on chromosomes 1, 6 and 9 than on other chromosomes, and one of the two telomeres of chromosomes 6 and 9 was a particularly polymorphism-rich region. However, the polymorphism densities were lower on chromosomes 4 and 10. Based on this distribution of SNPs and indels, it was possible to construct high-density genetic maps and select SNPs and indels within specific regions for fine mapping.

The association of predicted genes with SNPs and indels was also determined. The frequencies of genes in the 1-Mb genomic intervals were significantly positively correlated with those of SNPs (Spearman correlation coefficient = 0.39, p < 0.0001), but showed no correlation with those of indels (Spearman correlation coefficient = −0.08, p = 0.12). Moreover, the genomic distributions of genes and SSRs were investigated. The number of SSRs and genes was high at/near both ends but low in/near the middle of all the chromosomes (Supplementary Fig. 2), which likely corresponded to the peri-telomere and centromere, respectively^23^.

#### Validation of SNPs by SLAF-seq (specific length amplified fragment sequencing)

A total of 6,960 polymorphic SNPs (Supplementary Table 2) were obtained from ZXG01478 and 14CB11 by both SLAF-seq^20^ and the re-sequencing of two parental lines in this study. Of these SNPs, 6,712 (96.4%) were entirely consistent based on two independent sequence analyses, whereas 21 (0.3%) showed different sequences for one parental line. The remaining 227 SNPs (3.3%) showed confusing results because of uncertain sequences. A total of 2,431 SNPs anchored to the high-density genetic map were identified in the present re-sequencing analysis. Of these SNPs, 97.7% (2,375) were accurate, whereas 2.3% showed an error or uncertain sequences. Based on these data, the SNP accuracy of the present study was at least 96.4%.

### Fine mapping of the major QTL, *qSS6*

#### Development of PCR-based markers in the *qSS6* QTL region

The unique QTL, *qSS6*, showed a large effect for TSW, SL and SW and was regarded as a major QTL for seed size. To fine map this major pleiotropic QTL, the indels and SNPs in the QTL region were converted into PCR-based markers that can be rapidly and reliably analysed. To narrow down the QTL region gradually, three steps were used: first, indel markers were developed for indels with a length greater than 35 bp (base pairs); second, indel markers for indels with a length greater than 15 bp were developed based on the narrowed region; and third, CAPS and dCAPS (derived CAPS) markers were developed based on SNPs located in the re-narrowed region. Ultimately, 6, 3 and 3 indel, CAPS and dCAPS markers were developed, respectively, for further analysis (Supplementary Table 3).

#### Re-analysis of *qSS6* after adding newly developed markers

**Figure 4:**
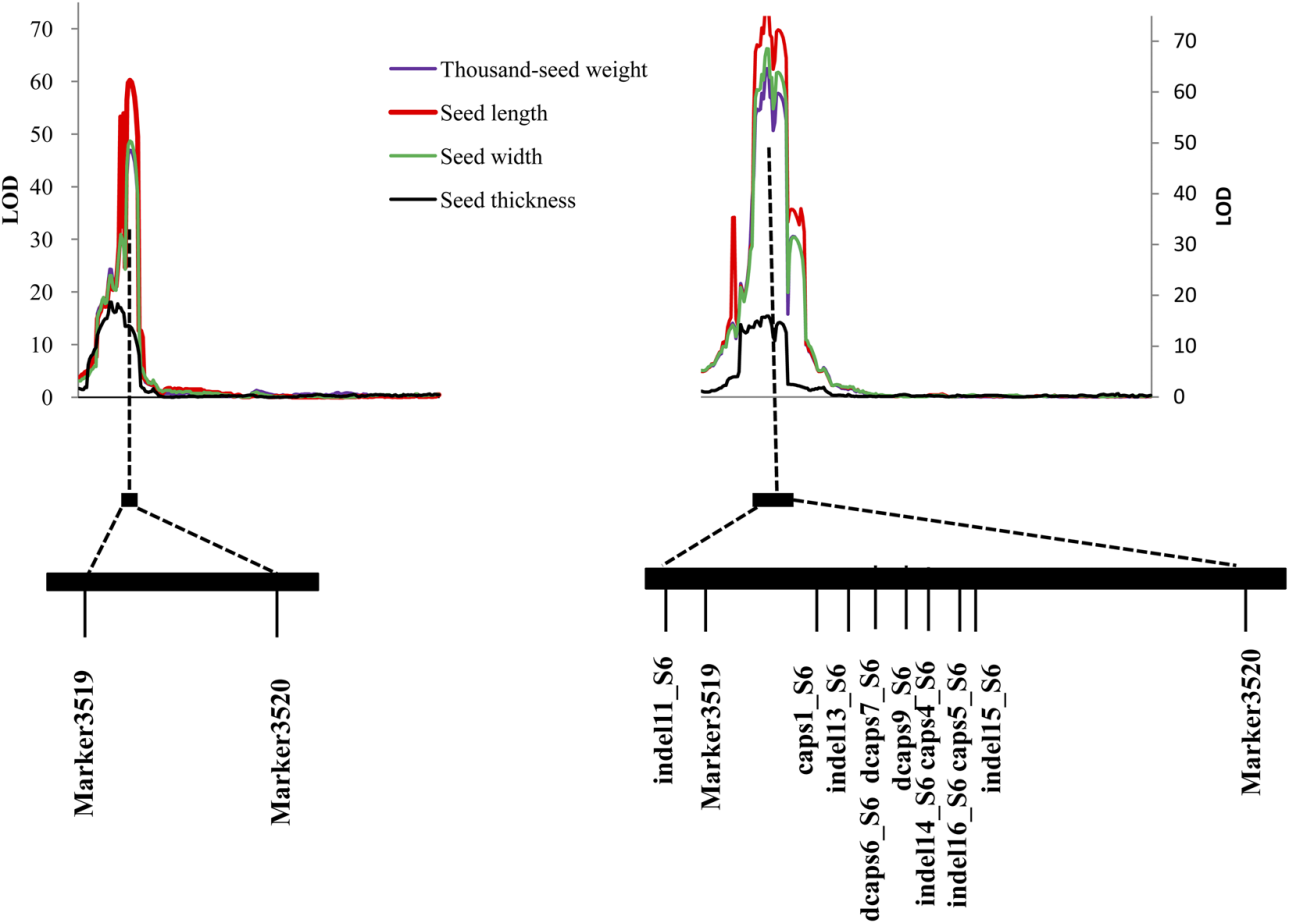
The comparison of LOD scanning before (left) and after (right) adding PCR-based markers to the QTL region. The markers are in the order of the partial map of LG6.

A total of 11 newly developed PCR-based markers were genotyped in the F_2_ population. Of these, dcaps6_S6 and dcaps7_S6, indel14_S6 and caps4_S6, and indel16_S6 and caps5_S6 were co-segregating in the F_2_ population. With SNP markers on LG6, the LG6 was re-constructed using JoinMap 4.0. QTL scanning was also performed using the re-constructed LG6 and other LGs. All of the peak LOD scores for TSW, SL and SW significantly increased after the newly developed markers were added (Fig. 4, Table 1), which suggested the importance of these markers. However, the LOD score for ST showed similar results after the newly developed markers were added, which again suggested that tight linkage rather than pleiotropy was the likely genetic basis of the co-localization of ST and other seed-size traits.

#### Fine mapping of *qSS6* using F_2_ and RIL (recombinant inbred line) populations

**Figure 5:**
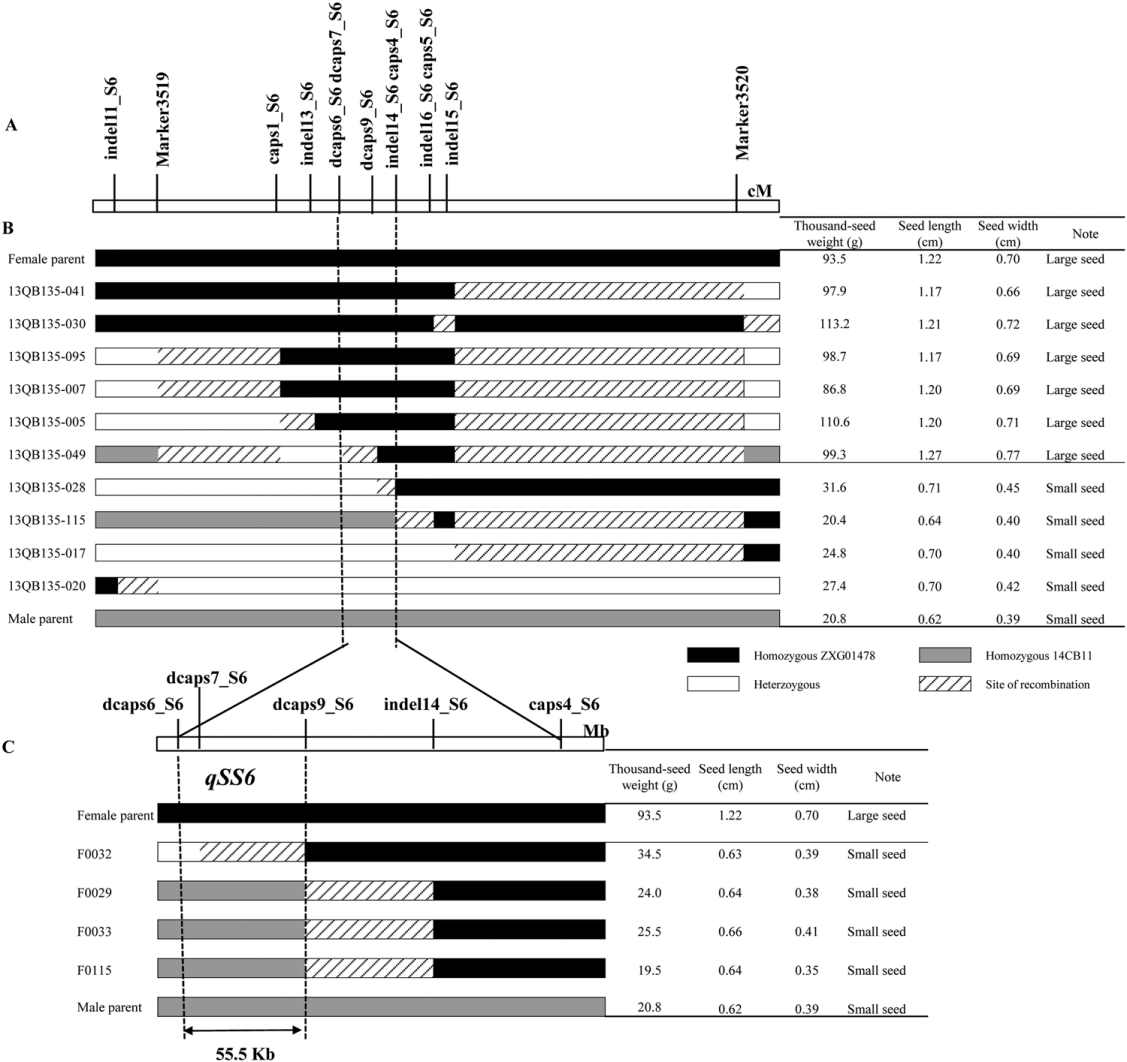
Graphical genotype of the selected recombinants and their seed size (including thousand-seed weight, seed length and seed width) in the F_2_ and RIL populations. (**A**) A high-resolution genetic map of the *qSS6* region on LG6. The number of recombinants between adjacent markers is indicated below the map. (**B**) The *qSS6* locus was narrowed to dcaps6_S6 and caps4_S6 by analysing the genotypes and phenotypes of the ten recombinants. (**C**) The *qSS6* locus was delimited to a 55.5-kb interval between dcaps6_S6 and dcaps9_S6 using five flanking markers and four recombinants in the RIL population.

Using flanking markers (Marker3519 and Marker3520), we identified ten recombinants around the *qSS6* locus. Using 11 newly developed co-dominant markers, a partial map of 17.22 cM was obtained (Fig. 5A). The target region was narrowed using these 10 recombinants in the F_2_ population (Fig. 5B). Of the six recombinants with large seeds, 13QB135–041 showed a ZXG01478 homozygous genotype to the left of indel15_S6 and was heterozygous for Marker3520, thus placing *qSS6* in a region upstream of Marker3520. Similarly, 13QB135-030 placed *qSS6* in a region upstream of indel15_S6, 13QB135-095 and 13QB135-007 placed *qSS6* in a region downstream of Marker3519, 13QB135-005 placed *qSS6* in a region downstream of caps1_S6, and 13QB135-049 delimited *qSS6* to a region downstream of dcaps6_S6 and dcaps7_S6. *qSS6* was narrowed to a region between dcaps6_S6 and indel15_S6 using six large-seed recombinants. Of the four recombinants with small seeds, 13QB135-028 delimited *qSS6* to a region upstream of indel14_S6 and caps4_S6, 13QB135-115 placed *qSS6* in a region upstream of indel16_S6 and caps5_S5, 13QB135-017 placed *qSS6* in a region upstream of Marker3510, and 13QB135-020 placed *qSS6* in a region downstream of indel11_S6. *qSS6* was narrowed to a region between indel11_S6 and caps4_S6 using four small-seed recombinants. As a result, *qSS6* was narrowed down to a 177.4-kb region between dcaps6_S6 and caps4_S6.

To further delimit *qSS6*, dcaps6_S6, dcaps7_S6, dcaps9_S6, indel14_S6 and caps4_S6 were used to screen recombinants in RILs, and four critical recombinants with small seeds were identified (Fig. 5C). F0032 showed a heterozygous genotype to the left of dcaps7_S6 and a ZXG01478 homozygous genotype to the right of dcaps9_S6, thus placing *qSS6* in a region downstream of dcaps9_S6. F0029, F0033 and F0115 showed a 14CB11 homozygous genotype to the left of dcaps9_S6 and a ZXG01478 homozygous genotype to the right of indel14_S6, thus delimiting *qSS6* to a region downstream of indel14_S6. Finally, *qSS6* was narrowed to a 55.5-kb region between dcaps6_S6 and dcaps9_S6. In this interval, the LOD scores of *TSW6*, *SL6* and *SW6* were 65.62, 60.08 and 71.75, respectively, which were also the peak values. Taken together, these results indicated that *qSS6* should be positioned in a 55.5-kb region between dcaps6_S6 and dcaps9_S6.

#### Homology and functional prediction of potential candidate genes underlying *qSS6*

**Figure 6:**
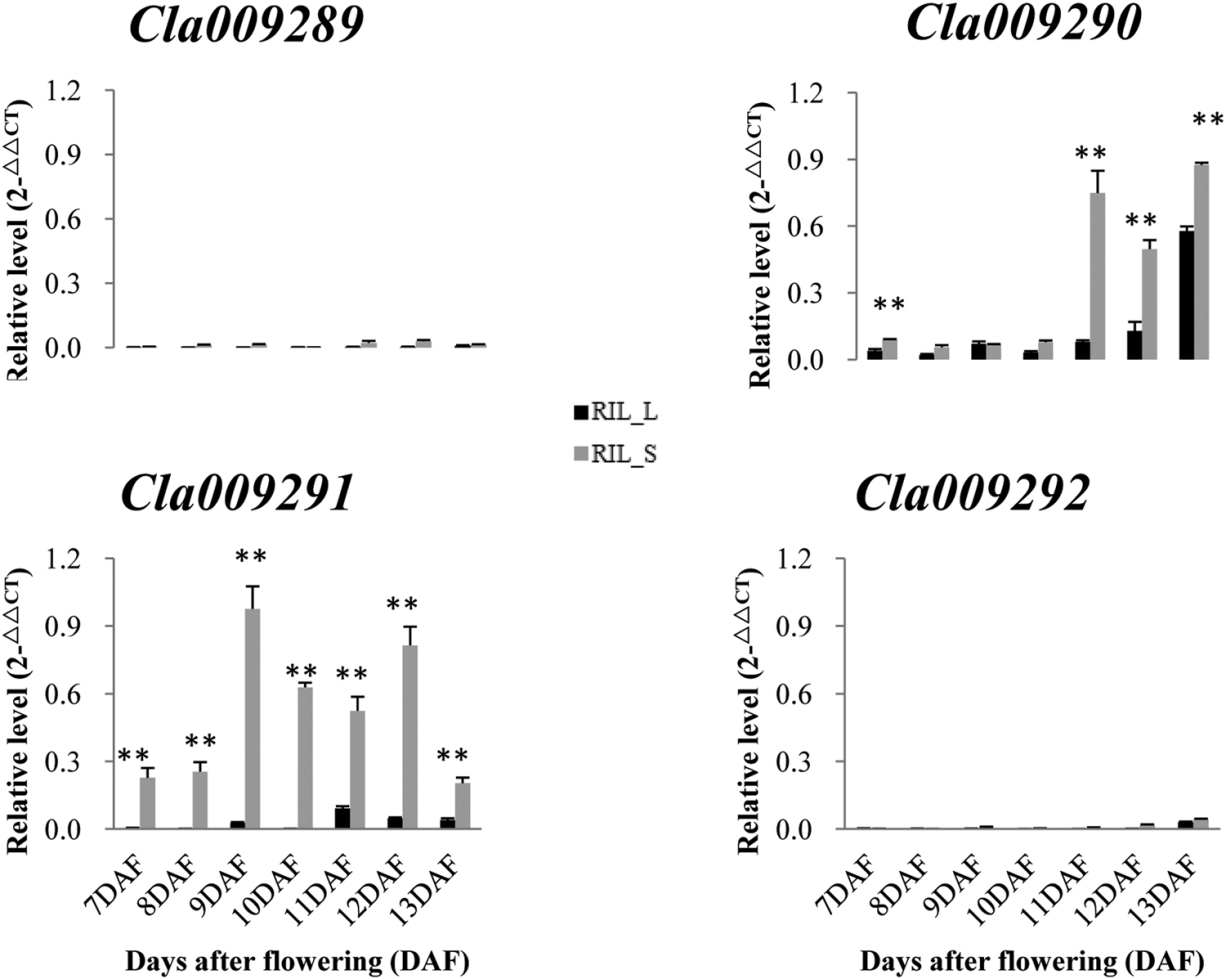
The expression profiles of potential candidate genes in seeds at different developmental stages using qRT-PCR. DAF is the abbreviation for days after flowering. Watermelon actin was used as an internal control. Data are expressed as the means of the three biological replicates; error bars indicates SDs; ** indicates significance at p < 0.001.

Based on the genome sequence of watermelon (http://www.icugi.org/), four putative genes were annotated (*Cla009289*, *Cla009290*, *Cla009291* and *Cla009292*) in the 55.5-kb interval. Protein BLAST pairwise alignment against the *Arabidopsis* database for *Cla009289* gave significant hits with the ATL gene family, which comprises a large number of putative ubiquitin ligases of the RING-H_2_ type^24^. *Cla009290* gave significant hits with *AT3G06840*.*1*, which encodes an unknown protein. *Cla009291* gave significant hits with MATE (multidrug and toxic compound extrusion) efflux family protein and putatively encoded the MDR (multidrug resistance) protein *mdtK*. *Cla009292* encoded *MARD1* (Mediator of ABA-Regulated Dormancy 1), which is an important downstream component of the ABA signalling pathway that mediates ABA-regulated seed dormancy in *Arabidopsis*^25^. qRT-PCR (quantitative RT-PCR) of RILs with large and small seeds indicated that only *Cla009291* was significantly differentially expressed at different seed developmental stages (Fig. 6). The marker caps5_S6, based on an SNP (G to T, in the first exon, S/end^4^) revealed a stop-gain effect for *Cla009310*. The putative gene *Cla009310* contained 195 amino acids and encoded an unknown protein. Moreover, near the QTL region, one annotated gene, *Cla009301*, encoded BY-kinesin-like protein 10, which contains a kinesin motor domain and is a homologue of *SRS3* (a gene regulating SL in rice^26^), was also a potential candidate gene under *qSS6*.

## Discussion

Here, using a high-resolution genetic map and whole-genome genetic variation detection aided by genome survey sequencing, we fine mapped and discovered candidate genes for seed size in watermelon. By simplifying genomic data using a reduced representation library (i.e., SLAF-seq), linkage and QTL identification were performed in a cost-effective manner and revealed a major pleiotropic QTL for seed size, *qSS6*, in an F_2_ population including 93 individuals (Fig. 2). Then, new PCR-based markers were developed based on deep re-sequencing of two parental lines, and fine mapping of *qSS6* was carried out to narrow the QTL to a 55.5-kb stretch flanked by dcaps6_S6 and dcaps9_S6, a segment that contained only four putative proteins, in the F_2_ and RIL populations (Fig. 5). One gene/QTL can be fine mapped to a small region if it exhibits the characteristics of segregation of a single Mendelian factor^27^ in an ideal mapping population, i.e., if the segregation pattern shows the following characteristics: (1) the frequency distribution of the trait shows clear discontinuous or bimodal segregation^27,28^; (2) the trait segregation exhibits a ratio of 3:1 or 1:2:1^29^. In this study, the bimodal segregation pattern of the data for TSW, SL and SW suggested that one major QTL controlled these traits (Fig. 1) in the F_2_ population. Therefore, it was possible to preliminarily fine map the major QTLs in this F_2_ population. In experiments aimed at fine mapping genes/QTLs, it is often necessary to saturate a specific target region with a large number of markers^30^. A total of 193,395 SNPs and 45,065 indels were identified between two parental lines in the present study. Of the SNPs, only 3.6% (6,960) showed polymorphism as detected by SLAF-seq, which ensured a high enough marker density when fine mapping the target QTL. Therefore, we have successfully narrowed the confidence interval of the target QTL to a physical distance of 55.5 kb. Compared with the traditional NIL (near-isogenic line)-based fine mapping strategy^31^, this method does not require the genotyping of a large-scale NIL segregating population and is labour and time saving. This rapid fine mapping strategy highlights the direction of gene mining, which could be applied to many traits of interest in plants.

Several previous studies used different mapping populations to identify QTLs for seed size in watermelon. Prothro *et al*.^16^ identified a major QTL for seed weight on chromosome 6 in the elite × elite [flanking markers NW0251236 (5.80 Mb) and NW0250242 (6.44 Mb)] and elite × citron [flanking markers NW0248118 (5.04 Mb) and NW0248583 (6.41 Mb)] populations. Ren *et al*. (2014) anchored these markers to an integrated map and found that a 0.62 Mb region (from 5.80 to 6.41 Mb) overlapped between the flanking markers in these populations. Meru and McGregor ^18^ found that the closest marker to a major QTL for 100-seed weight, SL and SW on chromosome 6 was NW0250854 (4.58 Mb). In the present study, according to the development of indel, CAPS and dCAPS markers based on indel and SNP information for the QTL region, the pleiotropic major QTL for seed size, *qSS6*, was narrowed to a physical distance of 55.5 kb (from 5.37 to 5.42 Mb) on chromosome 6 and only contained only four predicted genes. Notably, the allelic variation determined by the marker dcaps9_S6 (5.42 Mb) co-segregated with seed size in the F_2_ population. Our results confirmed the major QTL for seed size on LG6 and further corrected its precise location compared with previous studies^16,18^. In addition, the overlapping of the simple confidence intervals of different QTLs might have masked the correct position^17^. The major QTL for tomato seed was also identified on chromosome 2^19^. Two minor QTLs on chromosome 2 and one on chromosome 8 were identified^16,17^. We also identified a novel QTL for seed size on LG4.

As for the four putative candidate genes located in the narrowed *qSS6* region, only *Cla009291* was significantly differentially expressed at different seed developmental stages between large- and small-seeded lines. The annotated gene *Cla009291* encodes the MDR protein *mdtK*. The MDR transporter genes are an emerging group involved in a diverse array of developmental and metabolic processes in plants. For example, the MDR-like proteins *AtMDR1* and *AtPGP1* are required for phytohormone auxin-mediated plant development^32,33^. *ZmMrp3*, an MDR-associated protein, is required for the anthocyanin transport process in maize^34^. A MATE family member in *Arabidopsis thaliana*, *AtDTX50*, functions as an ABA efflux transporter^35^. *TT12* (*TRANSPARENT TESTA12*) encodes a multidrug secondary transporter-like protein required for flavonoid sequestration in vacuoles of the seed coat endothelium^36^. *ABS3* and *ABS4*, a subgroup of MATE family transporters, are potential negative regulators of hypocotyl cell elongation in *Arabidopsis*^37^. The determination of exact transport substrates and how they are linked to plant development is one of the major challenges for MATE transporters. *Cl009291* likely transports a small molecule(s) that is important for many aspects of seed development and may act as a seed growth regulator through its direct or indirect actions; thus, *Cl009291* is an important potential candidate gene in *qSS6*. Some SNPs can alter a protein directly, such as non-synonymous SNPs, stopped-gained or stop-lost SNPs, frameshift SNPs or SNPs in splice sites^38^. A stop-gained SNP in the first exon (S/end^4^) of *Cla009310* significantly altered the protein. Therefore, *Cla009310*, the gene near the narrowed *qSS6* region, is also a potential candidate. In rice, one causal gene for novel small and round seed phenotypes of *srs3* codes for a protein that contains a kinesin motor domain^26^. Comparative analysis suggested that the annotated watermelon gene *Cla009301*, a homologue of *SRS3*, encoding BY-kinesin-like protein 10, which contains a kinesin motor domain, is one of the important candidate genes for seed size in the QTL, *qSS6*.

In summary, using a high-resolution genetic map and whole-genome genetic variation detection aided by genome survey sequencing, we fine mapped *qSS6* and identified three candidate genes. The method could be applied to many traits of interest in plants. We also found useful PCR-based markers near *qSS6* that may assist in breeding for seed size and can lay the foundation for functional validation of the candidate genes to elucidate the molecular mechanism underlying seed size. In further studies, functional analysis of parental lines or NILs will be used to validate the candidate genes by sequence analysis and genetic transformation.

## Methods

### Plant materials, field experiments, trait evaluation and statistical analysis

A large F_2_ population of 536 individuals derived from a cross between ZXG01478 and 14CB11 showed significant differences in seed weight, SL and SW^20^. A total of 93 F_2_ individuals were randomly selected for genotyping and QTL analysis. A total of 122 F_2_ individuals were randomly selected and subjected to single-seed descent for four generations, which produced 106 RILs. Both parents, the F_1_s, F_2_ progeny and the RILs were planted at the Zhengzhou Fruits Research Institute of the Chinese Academy of Agricultural Sciences in Zhengzhou. The two parents and the F_1_s were grown in triplicate, with 10 plants each. Then, 93 F_2_ individuals and 106 RILs were planted in a green house following essentially regular breeding practices. All fruits were harvested at full maturity between 35 to 40 days after pollination, and a single fruit per plant was harvested. The seed weight of each fruit was measured based on 50 fully developed seeds. The average seed weight was then converted into TSW. The SL, SW and ST were measured using Vernier callipers based on 20 randomly selected seeds per fruit.

Pearson and Spearman correlation coefficients were calculated using SAS software^39^.

### QTL mapping of seed size

Genome-wide QTL mapping of TSW, SL, SW and ST was performed using the composite interval mapping (CIM) program^40^ in WinQTL Cartographer 2.5 software (http://statgen.ncsu.edu/qtlcart/WQTLCart.htm). The LOD threshold was determined via a permutation test with 1000 repetitions^41^. LOD scores corresponding to p = 0.05 were used to identify significant QTLs (4.22, 4.17, 4.27 and 4.16 for TSW, SL, SW and ST, respectively). The QTL intervals were established by 2-LOD as approximately 95% QTL confidence intervals. The GCIM method^42^ was also conducted to validate the above detected QTLs for seed size related traits.

To distinguish multiple closely linked QTLs from one pleiotropic QTL in one narrow interval, two approaches were adopted. One is to use multi-trait composite interval mapping^43^ implemented in WinQTL Cartographer v2.5. Another is to use conditional QTL mapping. The conditional phenotypic values y (T1/T2) were obtained by a mixed model approach for conditional analysis of quantitative traits^44^ using QGAStation 1.0 (http://ibi.zju.edu.cn/software/qga/index.htm), where T1|T2 indicates that trait 1 is conditioned by trait 2. Then, the conditional mapping of the QTLs was conducted according to the conditional phenotypic values using the same method as that for the unconditional QTLs mentioned above.

### Genome-wide identification of SNPs, indels and SSRs

ZXG01478 and 14CB11 were used in re-sequencing for genome-wide detection of SNPs and indels. The paired-end reads from the two parental watermelon lines were aligned to the reference genome of watermelon^22^ using the BWA (Burrows-Wheeler alignment tool)^45^. Sequence alignment file conversions were performed using SAMtools^46^. A total of 18.6 G of data was output from the two lines, which covered an average of 22 × of the watermelon genome. SNP and indel mining were performed using GATK (Genome Analysis Toolkit)^47^. The reference watermelon genome was used as a “bridge” to sequentially detect SNP and indel polymorphisms between two re-sequenced parental watermelon lines.

The identification and localization of SSRs in watermelon reference genome 97103 were accomplished by MISA (MIcroSAtellite, an SSR mining tool; http://pgrc.ipk-gatersleben.de/misa/misa.html). The minimum repeat unit was defined as 12, 6, 4, 3, 3 and 3 for mono-, di-, tri-, tetra-, penta- and hexa-nucleotides, respectively.

### PCR-based marker development

Extracting 500 bp before and after each SNP/indel locus was performed with a self-compiled script in Perl. To develop PCR-based CAPS and dCAPS markers, the free Web-based software dCAP Finder 2.0 (http://helix.wustl.edu/dcaps/dcaps.html) was used to find appropriate restriction enzymes for detecting SNPs^48^. Primer 5.0^49^ and Oligo 7^50^ were used to design the respective PCR primer sets (including CAPS, dCAPS and indel markers).

### Genotyping of CAPS, dCAPS and indel markers in the F_2_ and RIL populations

Genomic DNA was extracted from fresh leaves of the parents, F_1_s, F_2_ progeny and RILs using a modified CTAB method^51^. PCR was performed in 25-μl reaction volumes containing 12.5 μl 2 × Power Taq PCR MasterMix (Bioteke, Beijing, China) with 10 μM of each primer and approximately 50 ng of genomic DNA as a template. Thermocycling was started at 94 °C for 5 min, followed by 35 cycles of 94 °C for 20 s, 55 °C for 1 min and 72 °C for 30 s, with a final extension at 72 °C for 5 min. The PCR products were separated on 8% polyacrylamide gel and visualized by silver staining.

### Re-linkage analysis

Re-linkage analysis was performed using JoinMap 4.0 software (http://www.kyazma.nl/index.php/mc.JoinMap) with a goodness-of-fit threshold of ≦5, a recombination frequency of <0.4 and a minimum LOD score of 2.0. All genetic distances were expressed in cM as derived by the Kosambi function^52^.

### qRT-PCR analysis

To further narrow the candidate genes, the seeds were collected from 7 DAF (days after flowering) to 13 DAF to investigate the SL, SW and ST of the selected RILs (Supplementary Fig. 3) with large and small seeds: RIL_L (F0043) and RIL_S (F0055-1), respectively. The results showed that seeds expanded rapidly after flowering. Seeds generally expanded to their final size at 8 DAF and 12-13 DAF for RIL_L and RIL_S, respectively.

For the qRT-PCR template, the reverse transcription reaction was performed using a PrimeScript^TM^ II 1st Strand cDNA Synthesis Kit (Takara, Beijing, China). qRT-PCR was performed using a Bio-Rad IQ5 with SYBR Green detection. Relative expression levels were evaluated using the 2^−△△CT^ method. The watermelon actin gene was used as an internal control to normalize transcript levels. The primer details are shown in Supplementary Table 3. A cycling temperature of 57 °C and the criterion of a single peak on the melting curve were used to confirm the specificity of designed primer pairs.

## Electronic supplementary material

Supplementary information

## Data Availability

All data generated or analysed during this study are included in this published article and its Supplementary Information files.
