# Supplementary material for: Fine mapping and discovery of candidate genes for seed size in watermelon by genome survey sequencing
Source: Sci Rep. 2018 Dec 14;8:17843. doi: 10.1038/s41598-018-36104-w (PMC6294751; doi:10.1038/s41598-018-36104-w)
Supplement: Supplementary file 1 — Supplementary information [file 41598_2018_36104_MOESM1_ESM.pdf]

Fine mapping and discovery of candidate genes for seed size in watermelon by genome survey sequencing

Na Li, Jianli Shang, Jiming Wang, Dan Zhou, Nannan Li and Shuangwu Ma

Legends for Supplementary Figures and Tables

Supplementary Figure 1 The seeds of the two parents, F_1s and typical F_2 individuals. Bar = 1 cm.

Supplementary Figure 2 Genome-wide distribution of microsatellites and genes in the chromosomes of watermelon. The horizontal axis shows the chromosomes, which are divided into 1-Mb intervals. Note that 0 was assembled but not anchored to chromosomes in reference genome 97103.

Supplementary Figure 3 The trends in seed length, width and thickness in RIL_L and RIL_S during seed development. These traits were measured from 7 to 13 DAF. At least three individuals were used to reduce the experimental error. The bars represent means + SDs.

Supplementary Table 1 Pearson correlations for thousand-seed weight, seed length, seed width and seed thickness in the F_2 population.

Supplementary Table 2 Polymorphic SNPs between ZXG01478 and 14CB11 obtained by both SLAF-sequencing and re-sequencing.

Supplementary Table 3 Detailed information on the primer pairs.

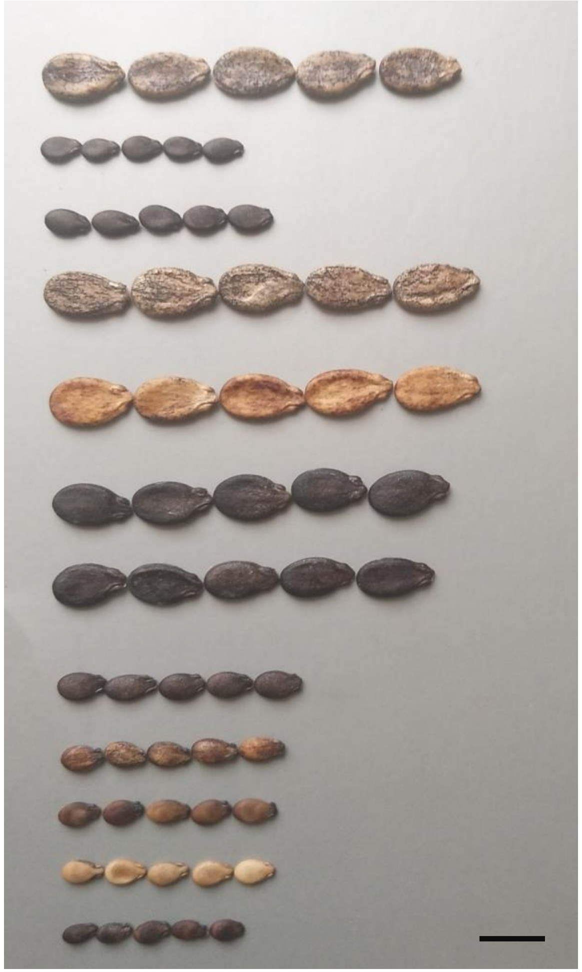

Female parent

Male parent

F_1

F_2: 13QB135-006

F_2: 13QB135-118

F_2: 13QB135-020

F_2: 13QB135-013

F_2: 13QB135-111

F_2: 13QB135-060

F_2: 13QB135-048

F_2: 13QB135-115

F_2: 13QB135-019

Supplementary Figure 1 The seeds of the two parents, F_1s and typical F_2 individuals. Bar = 1 cm.

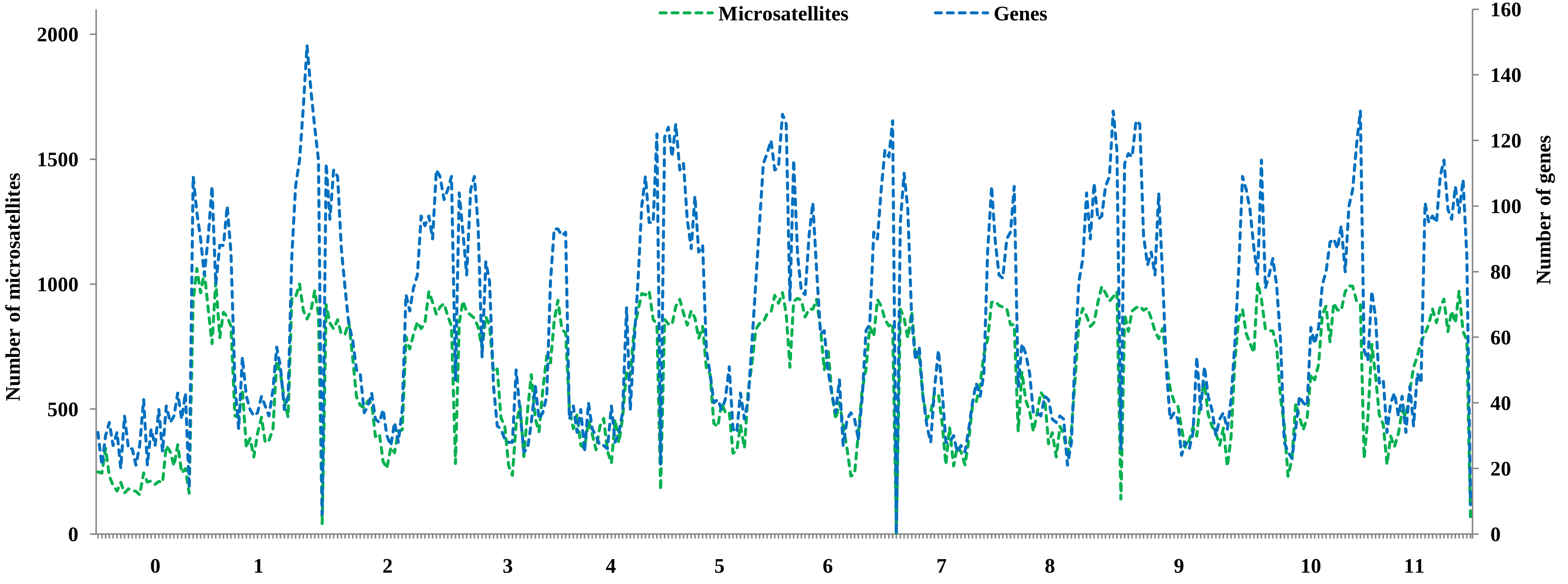

Supplementary Figure 2 Genome-wide distribution of microsatellites and genes in the chromosomes of watermelon.

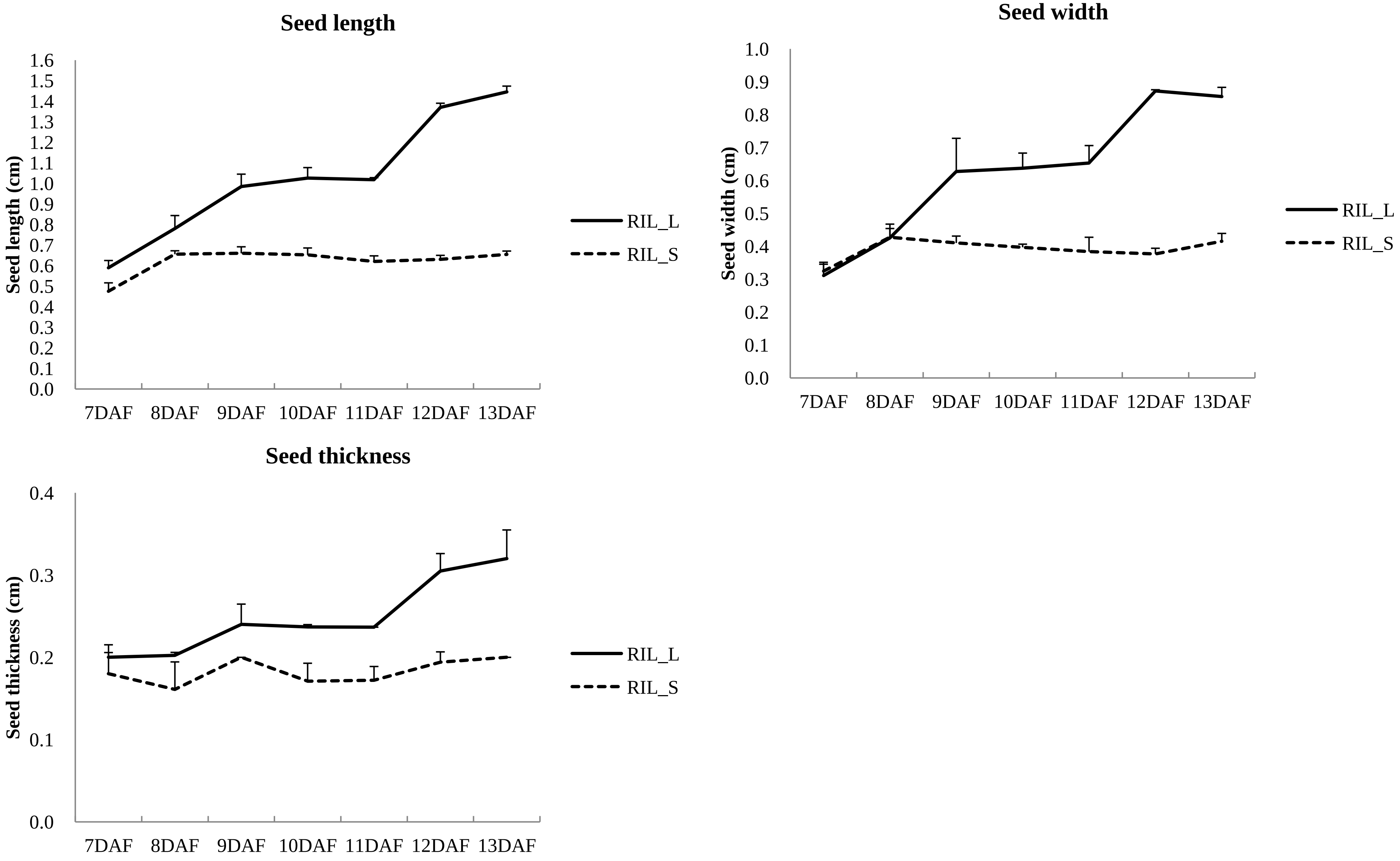

Supplementary Figure 3 The trends in seed length, width and thickness in RIL_L and RIL_S during seed development.

Supplementary Table 1 Pearson correlations for thousand-seed weight, seed length, seed width and seed thickness in the F_2 population.

	Thousand-seed weight	Seed length	Seed width
**Seed length**	0.989*[a]		
**Seed width**	0.987*	0.994*	
**Seed thickness**	0.800*	0.777*	0.766*

[a]: * indicates significant correlations at $P < 0.001$.

Supplementary Table 2 Polymorphic SNPs between ZXG01478 and 14CB11 obtained by both SLAF-sequencing and re-sequencing.

Marker ID	LG	Genetic distance	Chr.	Position	Reference	SLAF-seq		Re-seq [a]		Note
						Female	Male	Female	Male	
Marker770	LG01	0.00	Chr. 1	27209483	T	C	T	C	T	Accurate
Marker780	LG01	0.00	Chr. 1	27464340	T	C	T	C	T	Accurate
Marker761	LG01	0.00	Chr. 1	26605067	A	C	A	C	A	Accurate
Marker750	LG01	1.08	Chr. 1	26438229	G	A	G	A	G	Accurate
Marker744	LG01	3.23	Chr. 1	26154902	A	G	A	G	A	Accurate
Marker746	LG01	3.76	Chr. 1	26176109	C	T	C	T	C	Accurate
Marker224	LG01	3.76	Chr. 1	8496423	A	G	A	G	A	Accurate
Marker225	LG01	4.30	Chr. 1	8496628	G	T	G	T	G	Accurate
Marker227	LG01	6.45	Chr. 1	8644243	G	G	T	G	T	Accurate
Marker252	LG01	11.10	Chr. 1	9421854	G	G	A	G	A	Accurate
Marker255	LG01	13.61	Chr. 1	9549097	A	A	G	A	G	Accurate
Marker257	LG01	17.51	Chr. 1	9712104	T	C	T	C	T	Accurate
Marker261	LG01	22.54	Chr. 1	10225390	T	A	T	A	T	Accurate
Marker264	LG01	23.61	Chr. 1	10253791	A	G	A	G	A	Accurate
Marker265	LG01	24.74	Chr. 1	10253817	T	G	T	G	T	Accurate
Marker278	LG01	26.04	Chr. 1	10580284	T	C	T	C	T	Accurate
Marker280	LG01	27.12	Chr. 1	10606216	C	A	C	A	C	Accurate
Marker368	LG01	27.12	Chr. 1	11980510	G	A	G	A	G	Accurate
Marker377	LG01	27.12	Chr. 1	12046071	T	C	T	C	T	Accurate
Marker351	LG01	28.19	Chr. 1	11873636	C	T	C	T	C	Accurate
Marker375	LG01	28.19	Chr. 1	12030434	T	A	T	A	T	Accurate
Marker322	LG01	28.19	Chr. 1	11506918	T	C	T	C	T	Accurate
Marker330	LG01	28.19	Chr. 1	11544172	T	C	T	C	T	Accurate
Marker347	LG01	28.19	Chr. 1	11849536	C	T	C	T	C	Accurate
Marker327	LG01	28.73	Chr. 1	11525500	A	G	A	G	A	Accurate
Marker329	LG01	28.73	Chr. 1	11539426	T	C	T	C	T	Accurate
Marker339	LG01	28.73	Chr. 1	11823099	G	A	G	A	G	Accurate
Marker353	LG01	28.73	Chr. 1	11877419	G	A	G	A	G	Accurate
Marker359	LG01	28.73	Chr. 1	11905893	C	T	C	T	C	Accurate
Marker365	LG01	28.73	Chr. 1	11934001	C	T	C	T	C	Accurate
Marker374	LG01	28.73	Chr. 1	12011363	G	T	G	T	G	Accurate
Marker376	LG01	28.73	Chr. 1	12040407	A	G	A	G	A	Accurate
Marker393	LG01	33.26	Chr. 1	13183217	A	T	A	T	A	Accurate
Marker392	LG01	33.79	Chr. 1	13182940	T	C	T	C	T	Accurate
Marker388	LG01	35.41	Chr. 1	13151468	C	T	C	T	C	Accurate
Marker295	LG01	38.10	Chr. 1	10813193	A	T	A	T	A	Accurate
Marker292	LG01	39.17	Chr. 1	10783944	T	A	T	A	T	Accurate
Marker293	LG01	39.17	Chr. 1	10793986	C	A	C	A	C	Accurate
Marker312	LG01	40.25	Chr. 1	11176332	T	C	T	C	T	Accurate
Marker311	LG01	40.79	Chr. 1	11111547	C	T	C	T	C	Accurate
Marker420	LG01	41.86	Chr. 1	13847395	C	T	C	T	C	Accurate
Marker7975	LG01	42.40	Chr. 0	20410928	C	T	C	T	C	Accurate

Marker7872	LG01	42.94	Chr. 0	15213654	G	A	G	A	G	Accurate
Marker7890	LG01	43.48	Chr. 0	16784533	G	A	G	A	G	Accurate
Marker7891	LG01	43.48	Chr. 0	16786567	T	C	T	C	T	Accurate
Marker7973	LG01	43.48	Chr. 0	20263258	C	A	C	A	C	Accurate
Marker7971	LG01	44.01	Chr. 0	20245282	G	C	G	C	G	Accurate
Marker7892	LG01	44.01	Chr. 0	16795638	C	T	C	T	C	Accurate
Marker317	LG01	44.55	Chr. 1	11376695	A	G	A	G	A	Accurate
Marker316	LG01	45.09	Chr. 1	11376520	T	A	T	A	T	Accurate
Marker7919	LG01	46.16	Chr. 0	17806374	G	A	G	A	N	Uncertain
Marker410	LG01	48.32	Chr. 1	13471605	A	G	A	G	A	Accurate
Marker394	LG01	49.93	Chr. 1	13297519	A	G	A	G	A	Accurate
Marker396	LG01	49.93	Chr. 1	13367290	T	C	T	C	T	Accurate
Marker408	LG01	49.93	Chr. 1	13456773	C	A	C	A	C	Accurate
Marker412	LG01	49.93	Chr. 1	13495864	G	T	G	T	G	Accurate
Marker413	LG01	49.93	Chr. 1	13509960	A	C	A	C	A	Accurate
Marker400	LG01	50.47	Chr. 1	13415385	T	C	T	C	T	Accurate
Marker487	LG01	52.08	Chr. 1	17094112	A	G	A	G	A	Accurate
Marker486	LG01	53.69	Chr. 1	16856798	T	C	T	C	T	Accurate
Marker532	LG01	55.85	Chr. 1	17597707	A	T	A	T	A	Accurate
Marker495	LG01	56.92	Chr. 1	17255822	G	A	G	A	G	Accurate
Marker524	LG01	56.92	Chr. 1	17538284	T	G	T	G	T	Accurate
Marker530	LG01	56.92	Chr. 1	17583366	A	G	A	G	A	Accurate
Marker533	LG01	56.92	Chr. 1	17618386	C	T	C	T	C	Accurate
Marker537	LG01	56.92	Chr. 1	17678989	T	G	T	G	T	Accurate
Marker554	LG01	56.92	Chr. 1	18233752	A	A	G	A	G	Accurate
Marker526	LG01	57.46	Chr. 1	17538566	G	C	G	C	G	Accurate
Marker561	LG01	58.53	Chr. 1	18359876	T	G	T	G	T	Accurate
Marker614	LG01	61.22	Chr. 1	21205281	C	C	T	C	T	Accurate
Marker713	LG01	65.10	Chr. 1	24442393	C	C	T	C	T	Accurate
Marker580	LG01	65.64	Chr. 1	18919615	A	A	T	A	T	Accurate
Marker712	LG01	66.72	Chr. 1	24423352	T	T	C	T	C	Accurate
Marker723	LG01	68.87	Chr. 1	25313967	C	T	C	T	C	Accurate
Marker720	LG01	69.94	Chr. 1	25200617	A	G	A	G	A	Accurate
Marker727	LG01	70.48	Chr. 1	25493793	T	C	T	C	T	Accurate
Marker629	LG01	70.48	Chr. 1	21462035	A	G	A	G	A	Accurate
Marker630	LG01	70.48	Chr. 1	21467799	T	C	T	C	T	Accurate
Marker634	LG01	70.48	Chr. 1	21569587	G	C	G	C	G	Accurate
Marker639	LG01	70.48	Chr. 1	21762146	T	A	T	A	T	Accurate
Marker640	LG01	70.48	Chr. 1	21774040	G	A	G	A	G	Accurate
Marker730	LG01	70.48	Chr. 1	25546891	C	T	C	T	C	Accurate
Marker732	LG01	70.48	Chr. 1	25625444	T	A	T	A	T	Accurate
Marker736	LG01	70.48	Chr. 1	25895480	T	A	T	A	T	Accurate
Marker739	LG01	70.48	Chr. 1	25906467	A	G	A	G	A	Accurate
Marker740	LG01	70.48	Chr. 1	25925209	C	T	C	T	C	Accurate
Marker741	LG01	70.48	Chr. 1	25963851	A	G	A	G	A	Accurate
Marker618	LG01	71.02	Chr. 1	21402974	C	T	C	T	C	Accurate
Marker617	LG01	71.56	Chr. 1	21390123	G	A	G	A	G	Accurate

Marker726	LG01	71.56	Chr. 1	25372029	A	G	A	G	A	Accurate
Marker620	LG01	72.09	Chr. 1	21413540	A	G	A	G	A	Accurate
Marker724	LG01	72.09	Chr. 1	25368724	G	A	G	A	G	Accurate
Marker725	LG01	72.09	Chr. 1	25371998	G	T	G	T	G	Accurate
Marker721	LG01	73.39	Chr. 1	25202543	G	A	G	A	G	Accurate
Marker626	LG01	73.93	Chr. 1	21451386	T	C	T	C	T	Accurate
Marker705	LG01	76.08	Chr. 1	23749582	A	G	A	G	A	Accurate
Marker708	LG01	77.69	Chr. 1	23756820	T	C	T	C	T	Accurate
Marker703	LG01	78.23	Chr. 1	23702739	T	A	T	A	T	Accurate
Marker693	LG01	79.95	Chr. 1	22871846	T	T	C	T	C	Accurate
Marker694	LG01	80.72	Chr. 1	22884955	T	T	C	T	C	Accurate
Marker656	LG01	83.51	Chr. 1	22238809	T	T	C	T	C	Accurate
Marker657	LG01	84.05	Chr. 1	22239742	A	A	G	A	G	Accurate
Marker663	LG01	85.13	Chr. 1	22289687	G	G	A	G	A	Accurate
Marker689	LG01	85.67	Chr. 1	22685963	C	C	T	C	T	Accurate
Marker699	LG01	86.20	Chr. 1	23118302	A	A	G	A	G	Accurate
Marker697	LG01	86.74	Chr. 1	22919006	C	C	T	C	T	Accurate
Marker692	LG01	87.28	Chr. 1	22781604	A	A	C	A	C	Accurate
Marker662	LG01	87.82	Chr. 1	22289157	C	C	G	C	G	Accurate
Marker668	LG01	87.82	Chr. 1	22399594	G	G	A	G	A	Accurate
Marker672	LG01	87.82	Chr. 1	22408407	G	G	A	G	A	Accurate
Marker674	LG01	87.82	Chr. 1	22423098	A	A	G	A	G	Accurate
Marker675	LG01	87.82	Chr. 1	22476655	G	G	T	G	T	Accurate
Marker676	LG01	87.82	Chr. 1	22485373	G	G	A	G	A	Accurate
Marker678	LG01	87.82	Chr. 1	22522456	G	G	A	G	A	Accurate
Marker679	LG01	87.82	Chr. 1	22552262	A	A	C	A	C	Accurate
Marker680	LG01	87.82	Chr. 1	22572775	A	A	G	A	G	Accurate
Marker687	LG01	87.82	Chr. 1	22667990	T	T	A	T	A	Accurate
Marker690	LG01	87.82	Chr. 1	22691192	A	A	C	A	C	Accurate
Marker664	LG01	88.35	Chr. 1	22293859	G	G	A	G	A	Accurate
Marker659	LG01	88.89	Chr. 1	22253385	A	A	T	A	T	Accurate
Marker660	LG01	88.89	Chr. 1	22264208	G	G	A	G	A	Accurate
Marker644	LG01	90.73	Chr. 1	21880807	T	T	A	T	A	Accurate
Marker645	LG01	90.73	Chr. 1	21927576	C	C	A	C	A	Accurate
Marker650	LG01	90.73	Chr. 1	21944788	T	T	G	T	G	Accurate
Marker647	LG01	92.34	Chr. 1	21934099	G	G	A	G	A	Accurate
Marker651	LG01	92.88	Chr. 1	21950765	T	T	G	T	G	Accurate
Marker653	LG01	92.88	Chr. 1	22010823	T	T	C	T	C	Accurate
Marker217	LG01	94.49	Chr. 1	8175430	T	T	A	T	A	Accurate
Marker214	LG01	95.03	Chr. 1	8159004	A	A	T	A	T	Accurate
Marker218	LG01	95.03	Chr. 1	8177015	G	G	A	G	A	Accurate
Marker210	LG01	97.29	Chr. 1	7901337	T	T	C	T	C	Accurate
Marker208	LG01	97.83	Chr. 1	7818010	G	G	A	G	A	Accurate
Marker207	LG01	98.90	Chr. 1	7807699	A	A	G	A	G	Accurate
Marker204	LG01	101.16	Chr. 1	7423030	G	G	T	G	T	Accurate
Marker194	LG01	102.24	Chr. 1	7313183	T	T	C	T	C	Accurate
Marker195	LG01	102.24	Chr. 1	7338755	T	T	C	T	C	Accurate

Marker197	LG01	102.77	Chr. 1	7375654	C	C	T	C	T	Accurate
Marker200	LG01	102.77	Chr. 1	7387802	C	C	T	C	T	Accurate
Marker190	LG01	103.31	Chr. 1	7279421	T	T	C	T	C	Accurate
Marker188	LG01	104.39	Chr. 1	7218410	G	G	A	G	A	Accurate
Marker180	LG01	105.46	Chr. 1	7116280	T	T	C	T	C	Accurate
Marker172	LG01	106.00	Chr. 1	7018380	A	A	T	A	T	Accurate
Marker171	LG01	106.54	Chr. 1	6943094	C	C	G	C	G	Accurate
Marker176	LG01	106.54	Chr. 1	7049165	C	C	T	N	T	Uncertain
Marker178	LG01	106.54	Chr. 1	7057270	A	A	G	A	G	Accurate
Marker179	LG01	106.54	Chr. 1	7110218	T	T	C	T	C	Accurate
Marker181	LG01	107.07	Chr. 1	7116617	G	G	T	G	T	Accurate
Marker182	LG01	108.69	Chr. 1	7136041	C	C	T	C	T	Accurate
Marker185	LG01	108.69	Chr. 1	7194478	T	T	G	T	G	Accurate
Marker206	LG01	108.69	Chr. 1	7458568	T	T	A	T	A	Accurate
Marker184	LG01	109.23	Chr. 1	7150873	G	G	A	G	A	Accurate
Marker189	LG01	109.23	Chr. 1	7268066	C	C	T	C	T	Accurate
Marker162	LG01	110.52	Chr. 1	6655856	G	A	G	A	G	Accurate
Marker168	LG01	110.52	Chr. 1	6809783	T	C	T	C	T	Accurate
Marker159	LG01	111.60	Chr. 1	6595218	C	T	C	T	C	Accurate
Marker165	LG01	111.60	Chr. 1	6759571	G	C	G	C	G	Accurate
Marker167	LG01	111.60	Chr. 1	6776569	G	A	G	A	G	Accurate
Marker170	LG01	112.14	Chr. 1	6822014	A	G	A	G	A	Accurate
Marker169	LG01	112.67	Chr. 1	6810130	G	A	G	A	G	Accurate
Marker158	LG01	114.29	Chr. 1	6508990	G	A	G	A	G	Accurate
Marker145	LG01	114.82	Chr. 1	6400849	G	A	G	A	G	Accurate
Marker150	LG01	114.82	Chr. 1	6484044	T	A	T	A	T	Accurate
Marker151	LG01	114.82	Chr. 1	6491034	T	C	T	C	T	Accurate
Marker147	LG01	116.98	Chr. 1	6413322	A	C	A	C	A	Accurate
Marker140	LG01	119.23	Chr. 1	5877652	G	A	G	A	G	Accurate
Marker141	LG01	120.85	Chr. 1	6035306	T	A	T	A	T	Accurate
Marker143	LG01	120.85	Chr. 1	6077688	C	T	C	T	C	Accurate
Marker142	LG01	121.39	Chr. 1	6056675	A	T	A	T	A	Accurate
Marker139	LG01	121.93	Chr. 1	5872373	C	T	C	T	C	Accurate
Marker124	LG01	123.00	Chr. 1	4789040	A	G	A	G	A	Accurate
Marker127	LG01	123.00	Chr. 1	4797659	C	T	C	T	C	Accurate
Marker130	LG01	123.00	Chr. 1	4809452	C	T	C	T	C	Accurate
Marker134	LG01	123.54	Chr. 1	4856456	G	A	G	A	G	Accurate
Marker129	LG01	124.30	Chr. 1	4798622	G	A	G	A	G	Accurate
Marker114	LG01	125.38	Chr. 1	4329145	C	A	C	A	C	Accurate
Marker119	LG01	126.45	Chr. 1	4564819	A	G	A	G	A	Accurate
Marker115	LG01	126.99	Chr. 1	4524914	G	A	G	A	G	Accurate
Marker133	LG01	127.53	Chr. 1	4852383	C	A	C	A	C	Accurate
Marker118	LG01	128.60	Chr. 1	4564183	A	T	A	T	A	Accurate
Marker104	LG01	134.79	Chr. 1	4123394	C	C	T	C	T	Accurate
Marker137	LG01	137.70	Chr. 1	5776641	G	A	G	A	G	Accurate
Marker106	LG01	142.22	Chr. 1	4144747	T	T	C	T	C	Accurate
Marker138	LG01	143.83	Chr. 1	5845927	G	T	G	T	G	Accurate

Marker102	LG01	144.37	Chr. 1	3990831	G	C	G	C	G	Accurate
Marker100	LG01	145.98	Chr. 1	3885694	T	C	T	C	T	Accurate
Marker94	LG01	147.06	Chr. 1	3809091	T	C	T	C	T	Accurate
Marker98	LG01	147.60	Chr. 1	3832328	A	T	A	T	A	Accurate
Marker87	LG01	150.94	Chr. 1	3362753	C	T	C	T	C	Accurate
Marker86	LG01	152.01	Chr. 1	3362266	T	C	T	C	T	Accurate
Marker78	LG01	153.62	Chr. 1	3239490	C	T	C	T	C	Accurate
Marker68	LG01	154.70	Chr. 1	2957561	A	A	G	A	G	Accurate
Marker57	LG01	155.24	Chr. 1	2811926	T	T	C	T	C	Accurate
Marker73	LG01	155.24	Chr. 1	3169001	A	G	A	G	A	Accurate
Marker84	LG01	156.53	Chr. 1	3261641	T	C	T	C	T	Accurate
Marker50	LG01	158.15	Chr. 1	2750510	G	G	A	G	A	Accurate
Marker54	LG01	159.22	Chr. 1	2775421	C	C	A	C	A	Accurate
Marker49	LG01	159.98	Chr. 1	2715565	C	C	T	C	T	Accurate
Marker19	LG01	161.06	Chr. 1	2118564	G	T	G	T	G	Accurate
Marker47	LG01	161.06	Chr. 1	2714827	T	T	C	T	C	Accurate
Marker42	LG01	161.59	Chr. 1	2629826	C	T	C	T	C	Accurate
Marker28	LG01	162.13	Chr. 1	2264699	G	T	G	T	G	Accurate
Marker33	LG01	162.13	Chr. 1	2343955	A	G	A	G	A	Accurate
Marker34	LG01	162.13	Chr. 1	2346719	T	T	C	T	C	Accurate
Marker41	LG01	162.13	Chr. 1	2570116	A	C	A	C	A	Accurate
Marker27	LG01	162.67	Chr. 1	2264362	G	G	A	G	A	Accurate
Marker35	LG01	162.67	Chr. 1	2439525	T	C	T	C	T	Accurate
Marker37	LG01	162.67	Chr. 1	2526887	T	C	T	C	T	Accurate
Marker48	LG01	163.21	Chr. 1	2715303	C	C	G	C	G	Accurate
Marker62	LG01	163.75	Chr. 1	2907393	C	C	T	C	T	Accurate
Marker61	LG01	164.28	Chr. 1	2907219	A	A	G	A	G	Accurate
Marker59	LG01	165.04	Chr. 1	2862376	G	T	G	T	G	Accurate
Marker77	LG01	165.04	Chr. 1	3239115	G	T	G	T	G	Accurate
Marker67	LG01	165.58	Chr. 1	2956594	G	G	A	G	A	Accurate
Marker74	LG01	165.58	Chr. 1	3212102	T	G	T	G	T	Accurate
Marker71	LG01	166.65	Chr. 1	2961296	T	T	C	T	C	Accurate
Marker72	LG01	167.73	Chr. 1	3055255	A	T	A	T	A	Accurate
Marker20	LG01	169.34	Chr. 1	2125592	T	C	T	C	T	Accurate
Marker52	LG01	172.26	Chr. 1	2767860	G	G	C	G	C	Accurate
Marker44	LG01	173.98	Chr. 1	2683757	C	T	C	T	C	Accurate
Marker21	LG01	176.78	Chr. 1	2144284	A	T	A	T	A	Accurate
Marker16	LG01	180.36	Chr. 1	1272888	G	G	A	G	A	Accurate
Marker14	LG01	186.85	Chr. 1	704880	T	T	A	T	A	Accurate
Marker7	LG01	191.37	Chr. 1	349952	A	G	A	G	A	Accurate
Marker3	LG01	193.09	Chr. 1	193333	T	A	T	A	T	Accurate
Marker9	LG01	194.16	Chr. 1	403593	G	G	C	G	C	Accurate
Marker4	LG01	194.70	Chr. 1	341499	A	T	A	T	A	Accurate
Marker8	LG01	194.70	Chr. 1	403257	C	C	T	C	T	Accurate
Marker5	LG01	195.24	Chr. 1	341730	C	T	C	T	C	Accurate
Marker13	LG01	196.31	Chr. 1	465934	C	T	C	T	C	Accurate
Marker1252	LG02	0.00	Chr. 2	12640674	C	C	T	C	T	Accurate

Marker1264	LG02	1.61	Chr. 2	14821489	T	C	T	C	T	Accurate
Marker1265	LG02	1.61	Chr. 2	14855569	C	G	C	G	C	Accurate
Marker1266	LG02	1.61	Chr. 2	14858653	C	T	C	T	C	Accurate
Marker1257	LG02	4.85	Chr. 2	14716787	T	A	T	A	T	Accurate
Marker1259	LG02	4.85	Chr. 2	14741573	T	C	T	C	T	Accurate
Marker1260	LG02	6.31	Chr. 2	14800849	C	G	C	G	C	Accurate
Marker1010	LG02	7.92	Chr. 2	450638	C	T	C	T	C	Accurate
Marker1026	LG02	9.88	Chr. 2	1007450	G	G	T	G	T	Accurate
Marker1020	LG02	12.14	Chr. 2	673582	C	G	C	G	C	Accurate
Marker1028	LG02	12.91	Chr. 2	1037492	G	G	A	G	A	Accurate
Marker1032	LG02	13.67	Chr. 2	1048777	C	T	C	T	C	Accurate
Marker1016	LG02	14.43	Chr. 2	650475	C	T	C	T	C	Accurate
Marker1014	LG02	15.50	Chr. 2	464642	G	A	G	A	G	Accurate
Marker1004	LG02	16.04	Chr. 2	385475	G	C	G	C	G	Accurate
Marker1008	LG02	16.04	Chr. 2	402373	A	G	A	G	A	Accurate
Marker1009	LG02	16.04	Chr. 2	450556	C	T	C	T	C	Accurate
Marker1017	LG02	16.04	Chr. 2	665782	A	G	A	G	A	Accurate
Marker1022	LG02	16.04	Chr. 2	857274	A	A	C	A	C	Accurate
Marker1002	LG02	17.12	Chr. 2	369637	A	G	A	G	A	Accurate
Marker1003	LG02	17.65	Chr. 2	382872	A	G	A	G	A	Accurate
Marker1006	LG02	19.49	Chr. 2	393271	A	G	A	G	A	Accurate
Marker989	LG02	21.21	Chr. 2	59975	T	T	G	T	G	Accurate
Marker999	LG02	22.28	Chr. 2	156359	T	T	C	T	C	Accurate
Marker991	LG02	22.82	Chr. 2	80831	G	G	T	G	T	Accurate
Marker996	LG02	22.82	Chr. 2	128180	C	C	T	C	T	Accurate
Marker997	LG02	22.82	Chr. 2	140901	G	G	A	G	A	Accurate
Marker1034	LG02	22.82	Chr. 2	2052985	T	G	T	G	T	Accurate
Marker1038	LG02	22.82	Chr. 2	2181703	C	C	T	C	T	Accurate
Marker985	LG02	23.90	Chr. 2	57122	A	A	T	A	T	Accurate
Marker1036	LG02	23.90	Chr. 2	2155283	A	A	G	A	G	Accurate
Marker1039	LG02	23.90	Chr. 2	2350094	T	G	T	G	T	Accurate
Marker1044	LG02	23.90	Chr. 2	2424029	C	C	T	C	T	Accurate
Marker986	LG02	24.97	Chr. 2	57145	G	G	A	G	A	Accurate
Marker988	LG02	25.51	Chr. 2	59188	G	G	T	G	T	Accurate
Marker1040	LG02	25.51	Chr. 2	2353645	A	A	T	A	T	Accurate
Marker1042	LG02	26.05	Chr. 2	2414847	A	A	G	A	G	Accurate
Marker1048	LG02	26.59	Chr. 2	2663301	T	T	C	T	C	Accurate
Marker1045	LG02	27.12	Chr. 2	2427447	A	G	A	G	A	Accurate
Marker1046	LG02	28.20	Chr. 2	2427497	C	T	C	T	C	Accurate
Marker1052	LG02	29.81	Chr. 2	3087301	T	T	G	T	G	Accurate
Marker1053	LG02	30.89	Chr. 2	3087347	G	G	A	G	A	Accurate
Marker1049	LG02	31.43	Chr. 2	2998170	T	C	T	C	T	Accurate
Marker1057	LG02	31.43	Chr. 2	3314961	C	C	T	C	T	Accurate
Marker1062	LG02	31.43	Chr. 2	3404740	A	A	G	A	G	Accurate
Marker1051	LG02	32.52	Chr. 2	3043491	T	T	C	T	C	Accurate
Marker1059	LG02	34.14	Chr. 2	3316359	T	T	A	T	A	Accurate
Marker1054	LG02	35.21	Chr. 2	3293591	C	C	T	C	T	Accurate

Marker1061	LG02	36.29	Chr. 2	3388233	T	T	A	T	A	Accurate
Marker1055	LG02	39.09	Chr. 2	3309755	C	C	G	C	G	Accurate
Marker1086	LG02	44.05	Chr. 2	5810830	G	A	G	A	G	Accurate
Marker1094	LG02	46.85	Chr. 2	6156074	A	T	A	T	A	Accurate
Marker1092	LG02	48.30	Chr. 2	6106206	C	T	C	T	C	Accurate
Marker1087	LG02	49.59	Chr. 2	5811339	T	C	T	C	T	Accurate
Marker1093	LG02	51.21	Chr. 2	6134420	T	C	T	C	T	Accurate
Marker1107	LG02	54.54	Chr. 2	7899722	T	T	C	T	C	Accurate
Marker1106	LG02	55.08	Chr. 2	7899501	A	A	T	A	T	Accurate
Marker1105	LG02	56.16	Chr. 2	7898412	T	T	G	T	G	Accurate
Marker1101	LG02	58.96	Chr. 2	7503708	G	G	T	G	T	Accurate
Marker1102	LG02	60.57	Chr. 2	7503747	G	G	A	G	A	Accurate
Marker7712	LG02	62.18	Chr. 0	4358800	C	T	C	T	C	Accurate
Marker7713	LG02	62.94	Chr. 0	4365258	T	C	T	C	T	Accurate
Marker1103	LG02	62.94	Chr. 2	7811857	A	G	A	G	A	Accurate
Marker1117	LG02	64.78	Chr. 2	9971327	G	A	G	A	G	Accurate
Marker1116	LG02	65.85	Chr. 2	9935501	C	T	C	T	C	Accurate
Marker1114	LG02	66.93	Chr. 2	8872888	G	A	G	A	G	Accurate
Marker1108	LG02	68.00	Chr. 2	8463738	C	C	T	C	T	Accurate
Marker1207	LG02	71.23	Chr. 2	11444073	G	G	A	G	A	Accurate
Marker1206	LG02	71.99	Chr. 2	11443859	C	C	T	C	T	Accurate
Marker1132	LG02	73.60	Chr. 2	10875465	C	C	T	N	T	Uncertain
Marker1136	LG02	73.60	Chr. 2	10928040	T	T	C	T	C	Accurate
Marker1144	LG02	74.14	Chr. 2	10956164	C	C	G	C	G	Accurate
Marker1187	LG02	74.14	Chr. 2	11224598	T	T	A	T	A	Accurate
Marker1188	LG02	74.68	Chr. 2	11224742	T	T	C	T	C	Accurate
Marker1204	LG02	74.68	Chr. 2	11436970	T	T	C	T	C	Accurate
Marker1210	LG02	74.68	Chr. 2	11535131	A	A	G	A	G	Accurate
Marker1215	LG02	74.68	Chr. 2	11566242	T	T	C	T	C	Accurate
Marker1216	LG02	74.68	Chr. 2	11572936	A	A	G	A	G	Accurate
Marker1133	LG02	75.75	Chr. 2	10907328	A	A	G	A	G	Accurate
Marker1143	LG02	75.75	Chr. 2	10951977	G	G	A	G	A	Accurate
Marker1149	LG02	75.75	Chr. 2	10984220	A	A	T	A	T	Accurate
Marker1152	LG02	75.75	Chr. 2	11020039	A	A	G	A	G	Accurate
Marker1163	LG02	75.75	Chr. 2	11109797	T	T	A	T	A	Accurate
Marker1209	LG02	75.75	Chr. 2	11528139	A	A	G	A	G	Accurate
Marker1213	LG02	76.83	Chr. 2	11565704	T	T	C	T	C	Accurate
Marker1126	LG02	77.37	Chr. 2	10669529	G	G	A	G	A	Accurate
Marker1195	LG02	77.37	Chr. 2	11407922	G	A	G	A	G	Accurate
Marker1208	LG02	77.37	Chr. 2	11453500	A	A	T	A	T	Accurate
Marker1135	LG02	77.91	Chr. 2	10923021	G	G	C	G	C	Accurate
Marker1192	LG02	77.91	Chr. 2	11401347	C	T	C	T	C	Accurate
Marker1205	LG02	77.91	Chr. 2	11442849	A	A	G	A	G	Accurate
Marker1169	LG02	78.44	Chr. 2	11126434	T	T	C	T	C	Accurate
Marker1198	LG02	78.44	Chr. 2	11429348	A	A	G	A	G	Accurate
Marker1197	LG02	78.98	Chr. 2	11429344	T	T	C	T	C	Accurate
Marker1177	LG02	80.06	Chr. 2	11166844	A	A	G	A	G	Accurate

Marker1186	LG02	80.59	Chr. 2	11223184	T	T	G	T	G	Accurate
Marker1179	LG02	81.13	Chr. 2	11174986	A	A	G	A	G	Accurate
Marker1178	LG02	81.67	Chr. 2	11174736	G	G	A	G	A	Accurate
Marker1154	LG02	82.21	Chr. 2	11073555	T	T	G	T	G	Accurate
Marker1121	LG02	83.28	Chr. 2	10433244	T	G	T	G	T	Accurate
Marker1122	LG02	84.36	Chr. 2	10519725	C	A	C	A	N	Uncertain
Marker1285	LG02	86.51	Chr. 2	16216316	G	G	A	G	A	Accurate
Marker1286	LG02	87.05	Chr. 2	16216366	A	A	G	A	G	Accurate
Marker1255	LG02	88.12	Chr. 2	14039049	G	G	A	G	A	Accurate
Marker1280	LG02	88.66	Chr. 2	16168696	G	G	C	G	C	Accurate
Marker1288	LG02	88.66	Chr. 2	16231218	A	A	G	A	G	Accurate
Marker7744	LG02	88.66	Chr. 0	5706511	G	G	A	G	A	Accurate
Marker7745	LG02	89.20	Chr. 0	5734069	A	A	G	A	G	Accurate
Marker1295	LG02	89.20	Chr. 2	17376200	T	T	C	T	C	Accurate
Marker1297	LG02	89.20	Chr. 2	17392385	T	T	C	T	C	Accurate
Marker1298	LG02	89.20	Chr. 2	17399963	C	C	T	C	T	Accurate
Marker1299	LG02	89.20	Chr. 2	17410430	A	A	G	A	G	Accurate
Marker7698	LG02	90.81	Chr. 0	2330432	A	G	A	G	A	Accurate
Marker1334	LG02	91.89	Chr. 2	18708061	C	C	T	C	T	Accurate
Marker1333	LG02	92.42	Chr. 2	18707812	A	A	G	A	G	Accurate
Marker1326	LG02	92.96	Chr. 2	18636402	T	T	A	T	A	Accurate
Marker1341	LG02	92.96	Chr. 2	18770436	T	T	A	T	A	Accurate
Marker1349	LG02	92.96	Chr. 2	18820900	G	A	G	A	G	Accurate
Marker1351	LG02	92.96	Chr. 2	18860936	A	G	A	G	A	Accurate
Marker1347	LG02	93.50	Chr. 2	18813423	G	A	G	A	G	Accurate
Marker1368	LG02	93.50	Chr. 2	19147504	G	T	G	T	G	Accurate
Marker1370	LG02	93.50	Chr. 2	19153518	C	T	C	T	C	Accurate
Marker1375	LG02	93.50	Chr. 2	19201004	T	T	C	T	C	Accurate
Marker1395	LG02	93.50	Chr. 2	19468027	G	A	G	A	G	Accurate
Marker1399	LG02	93.50	Chr. 2	19485356	C	T	C	T	C	Accurate
Marker1403	LG02	93.50	Chr. 2	19521765	C	T	C	T	C	Accurate
Marker1331	LG02	94.04	Chr. 2	18696147	A	A	G	A	G	Accurate
Marker1352	LG02	94.04	Chr. 2	18890713	A	G	A	G	A	Accurate
Marker1323	LG02	94.57	Chr. 2	18628196	T	T	C	T	C	Accurate
Marker1366	LG02	95.11	Chr. 2	19095807	C	G	C	G	C	Accurate
Marker1401	LG02	95.65	Chr. 2	19505131	C	A	C	A	C	Accurate
Marker1308	LG02	96.19	Chr. 2	17890113	T	T	A	N	A	Uncertain
Marker1312	LG02	96.19	Chr. 2	17943680	T	A	T	A	T	Accurate
Marker1315	LG02	96.19	Chr. 2	17956817	T	C	T	C	T	Accurate
Marker1318	LG02	96.19	Chr. 2	18070254	A	G	A	G	A	Accurate
Marker1320	LG02	96.19	Chr. 2	18112223	C	T	C	T	C	Accurate
Marker1377	LG02	96.19	Chr. 2	19203347	G	G	A	G	A	Accurate
Marker1379	LG02	96.19	Chr. 2	19263808	G	G	T	G	T	Accurate
Marker1382	LG02	96.19	Chr. 2	19277298	T	T	C	N	C	Uncertain
Marker1389	LG02	96.19	Chr. 2	19397815	A	G	A	G	A	Accurate
Marker1390	LG02	96.19	Chr. 2	19426915	T	C	T	C	T	Accurate
Marker1392	LG02	96.19	Chr. 2	19440190	T	C	T	C	T	Accurate

Marker1398	LG02	96.19	Chr. 2	19479776	G	A	G	A	G	Accurate
Marker1400	LG02	96.19	Chr. 2	19488422	G	A	G	A	G	Accurate
Marker1407	LG02	96.19	Chr. 2	19830947	C	C	G	C	G	Accurate
Marker1409	LG02	96.19	Chr. 2	19850288	C	C	T	C	T	Accurate
Marker1410	LG02	96.19	Chr. 2	19898652	A	A	G	A	R	Uncertain
Marker1412	LG02	96.19	Chr. 2	19950430	G	G	A	G	A	Accurate
Marker1373	LG02	97.26	Chr. 2	19181961	T	T	C	T	C	Accurate
Marker1372	LG02	98.34	Chr. 2	19181461	T	T	C	T	C	Accurate
Marker1381	LG02	98.88	Chr. 2	19273384	C	T	C	T	C	Accurate
Marker1380	LG02	99.41	Chr. 2	19273360	G	A	G	A	G	Accurate
Marker1560	LG02	110.26	Chr. 2	25288120	G	A	G	A	G	Accurate
Marker1541	LG02	115.00	Chr. 2	24876935	G	A	G	A	G	Accurate
Marker1559	LG02	117.15	Chr. 2	25212985	T	C	T	C	N	Uncertain
Marker1575	LG02	117.69	Chr. 2	25852268	C	T	C	T	C	Accurate
Marker1565	LG02	119.30	Chr. 2	25384494	C	T	C	T	C	Accurate
Marker1592	LG02	119.30	Chr. 2	26475011	T	C	T	C	T	Accurate
Marker1593	LG02	119.84	Chr. 2	26475071	A	G	A	G	A	Accurate
Marker1534	LG02	120.92	Chr. 2	24830110	T	C	T	C	T	Accurate
Marker1540	LG02	120.92	Chr. 2	24875917	A	G	A	G	A	Accurate
Marker1542	LG02	120.92	Chr. 2	24885945	G	C	G	C	G	Accurate
Marker1545	LG02	121.45	Chr. 2	24891894	T	C	T	C	T	Accurate
Marker1567	LG02	121.45	Chr. 2	25588609	A	A	T	A	T	Accurate
Marker1526	LG02	122.53	Chr. 2	24699164	G	T	G	T	G	Accurate
Marker1517	LG02	124.14	Chr. 2	24540473	A	T	A	T	A	Accurate
Marker1523	LG02	124.14	Chr. 2	24666759	T	C	T	C	T	Accurate
Marker1528	LG02	124.14	Chr. 2	24740084	G	A	G	A	G	Accurate
Marker1508	LG02	125.22	Chr. 2	24375832	G	A	G	A	G	Accurate
Marker1514	LG02	126.29	Chr. 2	24493443	G	A	G	A	G	Accurate
Marker1492	LG02	128.44	Chr. 2	24170068	T	G	T	G	T	Accurate
Marker1499	LG02	128.44	Chr. 2	24249362	A	C	A	C	A	Accurate
Marker1504	LG02	128.44	Chr. 2	24257608	T	C	T	C	T	Accurate
Marker1515	LG02	128.44	Chr. 2	24526744	G	A	G	A	G	Accurate
Marker1516	LG02	128.98	Chr. 2	24536651	A	G	A	G	A	Accurate
Marker1518	LG02	128.98	Chr. 2	24560002	C	T	C	T	C	Accurate
Marker1519	LG02	132.21	Chr. 2	24560416	C	T	C	T	C	Accurate
Marker1493	LG02	134.90	Chr. 2	24178138	C	T	C	T	C	Accurate
Marker1495	LG02	136.73	Chr. 2	24184248	C	T	C	T	C	Accurate
Marker1500	LG02	138.99	Chr. 2	24252432	C	A	C	A	C	Accurate
Marker1813	LG02	152.71	Chr. 2	34260038	A	C	A	C	A	Accurate
Marker1817	LG02	157.90	Chr. 2	34320795	T	A	T	A	T	Accurate
Marker1818	LG02	159.86	Chr. 2	34320801	G	A	G	A	G	Accurate
Marker1819	LG02	162.23	Chr. 2	34328697	T	C	T	C	T	Accurate
Marker1815	LG02	162.77	Chr. 2	34270552	T	G	T	G	T	Accurate
Marker1812	LG02	166.65	Chr. 2	34178120	T	C	T	C	T	Accurate
Marker1799	LG02	167.73	Chr. 2	32982717	T	T	C	T	C	Accurate
Marker1803	LG02	168.91	Chr. 2	33025351	G	G	A	G	A	Accurate
Marker1804	LG02	168.91	Chr. 2	33087079	T	T	C	T	C	Accurate

Marker1797	LG02	169.98	Chr. 2	32855662	C	T	C	T	C	Accurate
Marker1800	LG02	169.98	Chr. 2	32984965	G	A	G	A	G	Accurate
Marker1788	LG02	170.52	Chr. 2	32367334	G	G	A	G	A	Accurate
Marker1790	LG02	171.06	Chr. 2	32395891	G	A	G	A	G	Accurate
Marker1791	LG02	171.06	Chr. 2	32401035	A	G	A	G	A	Accurate
Marker1785	LG02	171.60	Chr. 2	32358424	C	C	G	C	G	Accurate
Marker1786	LG02	171.60	Chr. 2	32363511	T	T	A	T	A	Accurate
Marker1802	LG02	171.60	Chr. 2	32997246	G	G	T	G	T	Accurate
Marker1838	LG03	0.00	Chr. 3	3175090	G	A	G	A	G	Accurate
Marker1837	LG03	0.54	Chr. 3	3175088	G	A	G	A	G	Accurate
Marker1839	LG03	0.54	Chr. 3	3187198	G	A	G	A	G	Accurate
Marker1830	LG03	1.08	Chr. 3	2950993	A	A	G	A	G	Accurate
Marker1825	LG03	1.61	Chr. 3	2749474	T	T	C	T	C	Accurate
Marker1826	LG03	2.69	Chr. 3	2749858	G	G	C	G	C	Accurate
Marker1827	LG03	3.23	Chr. 3	2751525	C	C	G	C	G	Accurate
Marker1823	LG03	3.76	Chr. 3	2698081	A	A	T	A	T	Accurate
Marker1822	LG03	4.30	Chr. 3	2684008	A	A	G	N	G	Uncertain
Marker1841	LG03	5.38	Chr. 3	3270850	A	T	A	T	A	Accurate
Marker1840	LG03	5.91	Chr. 3	3270632	T	C	T	C	T	Accurate
Marker1842	LG03	6.45	Chr. 3	3346108	T	T	G	T	G	Accurate
Marker1850	LG03	10.26	Chr. 3	3891611	C	T	C	T	C	Accurate
Marker1844	LG03	11.33	Chr. 3	3647004	C	A	C	A	C	Accurate
Marker1847	LG03	11.33	Chr. 3	3728682	A	G	A	G	A	Accurate
Marker1853	LG03	11.33	Chr. 3	3919260	T	C	T	C	T	Accurate
Marker1845	LG03	11.87	Chr. 3	3649791	G	A	G	A	G	Accurate
Marker1848	LG03	11.87	Chr. 3	3735808	A	C	A	C	A	Accurate
Marker1852	LG03	11.87	Chr. 3	3902756	T	C	T	C	T	Accurate
Marker1849	LG03	12.41	Chr. 3	3864258	G	A	G	A	G	Accurate
Marker1857	LG03	14.67	Chr. 3	4253674	A	C	A	C	A	Accurate
Marker1856	LG03	15.43	Chr. 3	4253485	A	G	A	G	A	Accurate
Marker1854	LG03	15.97	Chr. 3	4243772	T	A	T	A	T	Accurate
Marker1865	LG03	18.87	Chr. 3	4707153	G	A	G	A	G	Accurate
Marker1859	LG03	19.95	Chr. 3	4597757	G	A	G	A	G	Accurate
Marker1888	LG03	21.02	Chr. 3	5105050	C	T	C	T	C	Accurate
Marker1879	LG03	22.10	Chr. 3	5026240	A	G	A	G	A	Accurate
Marker1905	LG03	22.64	Chr. 3	5247131	G	C	G	C	G	Accurate
Marker1894	LG03	23.71	Chr. 3	5166528	A	G	A	G	A	Accurate
Marker1885	LG03	24.26	Chr. 3	5104630	C	T	C	T	C	Accurate
Marker1870	LG03	24.79	Chr. 3	4762212	G	C	G	C	G	Accurate
Marker1887	LG03	24.79	Chr. 3	5104889	A	G	A	G	A	Accurate
Marker1897	LG03	25.33	Chr. 3	5174598	G	A	G	A	G	Accurate
Marker1863	LG03	25.87	Chr. 3	4654087	A	G	A	G	A	Accurate
Marker1874	LG03	25.87	Chr. 3	5003109	T	A	T	A	T	Accurate
Marker1875	LG03	25.87	Chr. 3	5006957	C	T	C	T	C	Accurate
Marker1876	LG03	25.87	Chr. 3	5009280	A	G	A	G	A	Accurate
Marker1878	LG03	25.87	Chr. 3	5011771	G	A	G	A	N	Uncertain
Marker1883	LG03	25.87	Chr. 3	5065632	T	C	T	C	T	Accurate

Marker1884	LG03	25.87	Chr. 3	5093147	T	C	T	C	T	Accurate
Marker1890	LG03	25.87	Chr. 3	5134099	T	C	T	C	T	Accurate
Marker1891	LG03	25.87	Chr. 3	5141669	C	T	C	T	C	Accurate
Marker1898	LG03	25.87	Chr. 3	5174787	G	A	G	A	G	Accurate
Marker1900	LG03	25.87	Chr. 3	5186216	A	C	A	C	A	Accurate
Marker1860	LG03	26.41	Chr. 3	4647745	A	T	A	T	A	Accurate
Marker1880	LG03	27.48	Chr. 3	5028295	C	T	C	T	C	Accurate
Marker1907	LG03	29.09	Chr. 3	5392975	A	G	A	G	A	Accurate
Marker1911	LG03	30.17	Chr. 3	5517345	C	A	C	A	C	Accurate
Marker1912	LG03	30.17	Chr. 3	5531235	C	T	C	T	C	Accurate
Marker1915	LG03	31.78	Chr. 3	5673366	T	C	T	C	T	Accurate
Marker1928	LG03	31.78	Chr. 3	5905736	A	T	A	T	A	Accurate
Marker1923	LG03	32.86	Chr. 3	5841046	T	C	T	C	T	Accurate
Marker1913	LG03	33.40	Chr. 3	5659430	G	A	G	A	G	Accurate
Marker1926	LG03	35.01	Chr. 3	5884787	T	A	T	A	T	Accurate
Marker1930	LG03	36.09	Chr. 3	6519607	T	T	G	T	G	Accurate
Marker1931	LG03	38.46	Chr. 3	6663664	G	G	A	G	A	Accurate
Marker1933	LG03	40.62	Chr. 3	6906496	T	G	T	G	T	Accurate
Marker1936	LG03	40.62	Chr. 3	6976623	C	G	C	G	C	Accurate
Marker1938	LG03	40.62	Chr. 3	7029803	A	A	T	A	T	Accurate
Marker1939	LG03	41.15	Chr. 3	7040320	A	A	G	A	G	Accurate
Marker1940	LG03	41.69	Chr. 3	7045518	T	T	C	T	C	Accurate
Marker1944	LG03	43.31	Chr. 3	7143226	C	A	C	A	C	Accurate
Marker1951	LG03	43.31	Chr. 3	7221251	G	A	G	A	G	Accurate
Marker1941	LG03	43.84	Chr. 3	7073370	T	T	G	N	G	Uncertain
Marker1943	LG03	43.84	Chr. 3	7143006	G	A	G	A	G	Accurate
Marker1949	LG03	43.84	Chr. 3	7203182	T	C	T	C	T	Accurate
Marker1953	LG03	43.84	Chr. 3	7236288	C	G	C	G	C	Accurate
Marker1946	LG03	44.38	Chr. 3	7184406	T	C	T	C	T	Accurate
Marker1955	LG03	44.38	Chr. 3	7271885	C	T	C	T	C	Accurate
Marker1959	LG03	44.38	Chr. 3	7476171	A	C	A	C	A	Accurate
Marker1962	LG03	44.38	Chr. 3	7511627	C	G	C	G	C	Accurate
Marker1963	LG03	44.38	Chr. 3	7536461	C	A	C	A	C	Accurate
Marker1964	LG03	45.99	Chr. 3	7601784	G	G	A	G	A	Accurate
Marker1967	LG03	45.99	Chr. 3	7631830	C	C	A	C	A	Accurate
Marker1966	LG03	46.53	Chr. 3	7609478	T	T	A	T	A	Accurate
Marker1972	LG03	50.09	Chr. 3	8014759	C	T	C	T	C	Accurate
Marker1979	LG03	54.30	Chr. 3	8577525	A	A	G	A	G	Accurate
Marker2017	LG03	56.56	Chr. 3	9717370	G	G	A	G	A	Accurate
Marker2013	LG03	57.86	Chr. 3	9646236	G	G	A	G	A	Accurate
Marker2016	LG03	59.05	Chr. 3	9712923	T	T	G	T	G	Accurate
Marker2015	LG03	60.36	Chr. 3	9707659	C	C	A	C	A	Accurate
Marker2018	LG03	62.73	Chr. 3	9718017	T	T	A	T	A	Accurate
Marker2026	LG03	65.10	Chr. 3	10037445	C	C	T	C	T	Accurate
Marker2052	LG03	67.72	Chr. 3	10617115	C	T	C	T	C	Accurate
Marker2053	LG03	68.79	Chr. 3	10618572	A	C	T	C	C	Inconsistent
Marker2054	LG03	69.87	Chr. 3	10630047	G	A	G	A	G	Accurate

Marker2056	LG03	71.59	Chr. 3	10633597	T	G	T	G	T	Accurate
Marker2057	LG03	72.13	Chr. 3	10633634	G	A	G	A	G	Accurate
Marker2055	LG03	73.20	Chr. 3	10633419	T	C	T	C	T	Accurate
Marker2158	LG03	83.36	Chr. 3	21615062	C	T	C	T	C	Accurate
Marker2151	LG03	83.90	Chr. 3	21509662	T	C	T	C	T	Accurate
Marker2154	LG03	84.44	Chr. 3	21541499	G	T	G	T	G	Accurate
Marker2123	LG03	84.44	Chr. 3	20590632	G	A	G	A	G	Accurate
Marker2130	LG03	84.44	Chr. 3	20702507	G	A	G	A	G	Accurate
Marker2135	LG03	84.44	Chr. 3	20800879	G	A	G	A	G	Accurate
Marker2075	LG03	84.44	Chr. 3	18532656	C	C	T	C	T	Accurate
Marker2081	LG03	84.44	Chr. 3	18563588	C	C	T	C	T	Accurate
Marker2086	LG03	84.44	Chr. 3	18595496	T	T	A	T	A	Accurate
Marker2066	LG03	84.97	Chr. 3	18506860	G	G	A	G	A	Accurate
Marker2076	LG03	84.97	Chr. 3	18533450	C	C	G	C	G	Accurate
Marker2079	LG03	84.97	Chr. 3	18540296	G	G	T	G	T	Accurate
Marker2085	LG03	84.97	Chr. 3	18588082	A	A	G	A	G	Accurate
Marker2115	LG03	84.97	Chr. 3	20495334	C	T	C	T	C	Accurate
Marker2129	LG03	84.97	Chr. 3	20658297	A	G	A	G	A	Accurate
Marker2156	LG03	84.97	Chr. 3	21579614	G	A	G	A	G	Accurate
Marker2157	LG03	84.97	Chr. 3	21601042	A	G	A	G	A	Accurate
Marker2063	LG03	85.51	Chr. 3	17798816	A	A	T	A	T	Accurate
Marker2070	LG03	85.51	Chr. 3	18523593	A	A	T	A	T	Accurate
Marker2084	LG03	85.51	Chr. 3	18572939	G	G	A	G	A	Accurate
Marker2065	LG03	86.05	Chr. 3	18501584	T	T	C	T	C	Accurate
Marker2112	LG03	87.12	Chr. 3	20480014	C	T	C	Y	C	Uncertain
Marker2080	LG03	87.66	Chr. 3	18542502	T	T	G	T	G	Accurate
Marker2122	LG03	87.66	Chr. 3	20568577	C	T	C	T	C	Accurate
Marker2124	LG03	87.66	Chr. 3	20594615	G	A	G	A	G	Accurate
Marker2083	LG03	88.74	Chr. 3	18566066	A	A	C	A	C	Accurate
Marker2088	LG03	90.46	Chr. 3	18658724	G	G	A	G	A	Accurate
Marker2136	LG03	90.46	Chr. 3	20953017	A	G	A	G	A	Accurate
Marker2138	LG03	90.46	Chr. 3	20966296	T	C	T	C	T	Accurate
Marker2141	LG03	90.46	Chr. 3	21194272	T	C	T	C	T	Accurate
Marker2146	LG03	90.46	Chr. 3	21279369	G	A	G	A	G	Accurate
Marker2139	LG03	91.00	Chr. 3	20970069	G	A	G	A	G	Accurate
Marker2143	LG03	91.53	Chr. 3	21242627	T	A	T	A	T	Accurate
Marker2092	LG03	92.07	Chr. 3	19220880	G	C	G	C	G	Accurate
Marker2093	LG03	93.68	Chr. 3	19221462	G	A	G	A	G	Accurate
Marker2099	LG03	95.30	Chr. 3	19287135	C	T	C	T	C	Accurate
Marker2103	LG03	95.30	Chr. 3	19341578	A	C	A	C	A	Accurate
Marker2102	LG03	95.84	Chr. 3	19331777	A	G	A	G	A	Accurate
Marker2104	LG03	95.84	Chr. 3	19364431	A	C	A	C	A	Accurate
Marker2105	LG03	95.84	Chr. 3	19408787	C	G	C	G	C	Accurate
Marker2100	LG03	96.91	Chr. 3	19289356	T	C	T	C	T	Accurate
Marker2101	LG03	96.91	Chr. 3	19315654	G	A	G	A	G	Accurate
Marker2163	LG03	101.43	Chr. 3	22639309	C	T	C	T	C	Accurate
Marker2166	LG03	103.15	Chr. 3	22651361	C	A	C	A	C	Accurate

Marker2168	LG03	103.15	Chr. 3	22693794	T	G	T	G	T	Accurate
Marker2172	LG03	104.33	Chr. 3	22758985	A	A	G	A	G	Accurate
Marker2173	LG03	104.87	Chr. 3	22758988	C	C	A	C	A	Accurate
Marker2171	LG03	105.95	Chr. 3	22746407	A	A	G	A	G	Accurate
Marker2164	LG03	106.71	Chr. 3	22642463	G	A	G	A	G	Accurate
Marker2165	LG03	108.32	Chr. 3	22649471	G	A	G	A	G	Accurate
Marker2169	LG03	109.50	Chr. 3	22704816	C	T	C	T	C	Accurate
Marker2178	LG03	112.81	Chr. 3	23459752	C	C	T	C	T	Accurate
Marker2179	LG03	114.53	Chr. 3	23485233	T	T	C	N	C	Uncertain
Marker2181	LG03	118.73	Chr. 3	23983205	C	T	C	T	C	Accurate
Marker2189	LG03	122.55	Chr. 3	25705561	T	T	C	T	C	Accurate
Marker2200	LG03	126.53	Chr. 3	25991994	C	T	C	T	C	Accurate
Marker2194	LG03	128.79	Chr. 3	25959358	A	G	A	G	A	Accurate
Marker2198	LG03	128.79	Chr. 3	25973110	C	T	C	T	C	Accurate
Marker2199	LG03	128.79	Chr. 3	25991636	C	A	C	A	C	Accurate
Marker2202	LG03	128.79	Chr. 3	26045052	T	G	T	G	T	Accurate
Marker2206	LG03	128.79	Chr. 3	26069273	C	A	C	A	C	Accurate
Marker2209	LG03	128.79	Chr. 3	26206082	G	G	T	G	T	Accurate
Marker2216	LG03	128.79	Chr. 3	26476994	T	C	T	C	N	Uncertain
Marker2210	LG03	130.41	Chr. 3	26367794	A	G	A	G	A	Accurate
Marker2212	LG03	132.02	Chr. 3	26441123	A	T	A	T	A	Accurate
Marker2214	LG03	132.02	Chr. 3	26454832	T	C	T	C	T	Accurate
Marker2213	LG03	132.78	Chr. 3	26454604	G	T	G	T	G	Accurate
Marker2211	LG03	133.85	Chr. 3	26385504	T	C	T	C	T	Accurate
Marker2217	LG03	135.57	Chr. 3	26477244	A	G	A	G	N	Uncertain
Marker2235	LG03	138.37	Chr. 3	27164769	T	T	A	T	A	Accurate
Marker2226	LG03	139.45	Chr. 3	27104154	G	A	G	A	G	Accurate
Marker2232	LG03	141.71	Chr. 3	27129784	A	G	A	G	A	Accurate
Marker2225	LG03	143.32	Chr. 3	27094812	G	T	G	T	G	Accurate
Marker2239	LG03	143.32	Chr. 3	27357976	A	A	C	A	C	Accurate
Marker2234	LG03	143.86	Chr. 3	27138819	A	G	A	G	A	Accurate
Marker2223	LG03	144.40	Chr. 3	27084465	G	C	G	C	G	Accurate
Marker2233	LG03	144.40	Chr. 3	27138612	A	T	A	T	A	Accurate
Marker2238	LG03	144.40	Chr. 3	27346003	C	C	A	C	A	Accurate
Marker2228	LG03	144.93	Chr. 3	27112990	G	T	G	T	G	Accurate
Marker2237	LG03	144.93	Chr. 3	27180270	A	T	A	T	A	Accurate
Marker2229	LG03	145.47	Chr. 3	27118344	C	A	C	A	C	Accurate
Marker2227	LG03	146.55	Chr. 3	27105839	T	C	T	C	T	Accurate
Marker2240	LG03	150.53	Chr. 3	27591693	G	G	A	G	A	Accurate
Marker2242	LG03	153.87	Chr. 3	27709886	G	G	A	G	A	Accurate
Marker2258	LG03	157.21	Chr. 3	28401183	C	G	C	G	C	Accurate
Marker2263	LG03	157.75	Chr. 3	28495177	C	A	C	A	C	Accurate
Marker2257	LG03	158.82	Chr. 3	28400585	C	T	C	T	C	Accurate
Marker2262	LG03	158.82	Chr. 3	28470684	G	A	G	A	G	Accurate
Marker2266	LG03	158.82	Chr. 3	28550129	G	A	G	A	G	Accurate
Marker2269	LG03	158.82	Chr. 3	28595648	T	G	T	G	T	Accurate
Marker2244	LG03	160.44	Chr. 3	28265536	C	A	C	A	C	Accurate

Marker2250	LG03	160.44	Chr. 3	28324091	C	A	C	A	C	Accurate
Marker2260	LG03	160.44	Chr. 3	28431633	T	A	T	A	T	Accurate
Marker2248	LG03	160.97	Chr. 3	28316392	A	T	A	T	A	Accurate
Marker2252	LG03	160.97	Chr. 3	28341602	C	A	C	A	C	Accurate
Marker2255	LG03	160.97	Chr. 3	28375059	T	C	T	C	T	Accurate
Marker2256	LG03	160.97	Chr. 3	28390805	T	A	T	A	T	Accurate
Marker2328	LG04	0.00	Chr. 4	684136	A	G	A	G	A	Accurate
Marker2340	LG04	0.54	Chr. 4	3190150	G	A	G	A	G	Accurate
Marker2355	LG04	1.08	Chr. 4	3261835	A	G	A	G	A	Accurate
Marker2345	LG04	1.61	Chr. 4	3197745	T	C	T	C	T	Accurate
Marker2353	LG04	1.61	Chr. 4	3243975	T	C	T	C	T	Accurate
Marker2362	LG04	1.61	Chr. 4	3309005	T	C	T	C	T	Accurate
Marker2351	LG04	2.15	Chr. 4	3237799	T	C	T	C	T	Accurate
Marker7802	LG04	2.15	Chr. 0	11647473	A	G	A	G	A	Accurate
Marker7702	LG04	2.69	Chr. 0	2761079	C	T	C	T	C	Accurate
Marker7705	LG04	2.69	Chr. 0	2789643	C	T	C	T	C	Accurate
Marker2285	LG04	2.69	Chr. 4	208663	T	T	G	T	G	Accurate
Marker2295	LG04	3.23	Chr. 4	319511	C	C	T	C	T	Accurate
Marker2277	LG04	4.30	Chr. 4	161806	A	A	G	A	G	Accurate
Marker2287	LG04	4.30	Chr. 4	227000	A	A	T	A	T	Accurate
Marker2289	LG04	4.30	Chr. 4	273761	G	G	A	G	A	Accurate
Marker2294	LG04	4.30	Chr. 4	300712	T	T	C	T	C	Accurate
Marker2309	LG04	4.30	Chr. 4	453708	A	A	T	A	T	Accurate
Marker2313	LG04	4.30	Chr. 4	486194	A	G	A	G	A	Accurate
Marker2276	LG04	4.84	Chr. 4	161484	T	T	A	T	A	Accurate
Marker2306	LG04	4.84	Chr. 4	439432	T	T	C	T	C	Accurate
Marker2357	LG04	4.84	Chr. 4	3295182	G	A	G	A	G	Accurate
Marker2364	LG04	4.84	Chr. 4	3337813	T	C	T	C	T	Accurate
Marker2365	LG04	4.84	Chr. 4	3374394	C	A	C	A	C	Accurate
Marker2341	LG04	4.84	Chr. 4	3190254	A	G	A	G	A	Accurate
Marker2343	LG04	5.38	Chr. 4	3197207	T	A	T	A	T	Accurate
Marker2292	LG04	6.14	Chr. 4	293218	T	T	A	T	A	Accurate
Marker2319	LG04	6.67	Chr. 4	558239	G	G	T	G	T	Accurate
Marker2356	LG04	7.98	Chr. 4	3291233	G	T	G	T	G	Accurate
Marker2333	LG04	11.31	Chr. 4	2469076	G	G	A	G	A	Accurate
Marker2334	LG04	12.39	Chr. 4	2489124	G	G	A	G	A	Accurate
Marker7734	LG04	16.92	Chr. 0	5116191	T	C	T	C	T	Accurate
Marker2331	LG04	17.99	Chr. 4	1422758	G	A	G	A	G	Accurate
Marker2369	LG04	18.53	Chr. 4	3709462	T	C	T	C	T	Accurate
Marker2376	LG04	20.79	Chr. 4	4621602	C	T	C	T	C	Accurate
Marker7738	LG04	21.33	Chr. 0	5193565	T	A	T	A	T	Accurate
Marker7737	LG04	21.87	Chr. 0	5134261	A	G	A	G	A	Accurate
Marker2375	LG04	22.40	Chr. 4	4615927	A	G	A	G	A	Accurate
Marker7739	LG04	23.48	Chr. 0	5206048	A	G	A	G	A	Accurate
Marker2374	LG04	24.55	Chr. 4	4615907	G	A	G	A	G	Accurate
Marker2372	LG04	25.09	Chr. 4	3857396	A	T	A	T	A	Accurate
Marker2384	LG04	26.17	Chr. 4	4770618	T	C	T	C	T	Accurate

Marker2386	LG04	27.24	Chr. 4	4773868	T	C	T	C	T	Accurate
Marker2382	LG04	28.86	Chr. 4	4752562	G	A	G	A	G	Accurate
Marker2383	LG04	28.86	Chr. 4	4761438	A	G	A	G	A	Accurate
Marker2391	LG04	28.86	Chr. 4	5071201	A	G	A	G	A	Accurate
Marker2392	LG04	29.39	Chr. 4	5192667	C	T	C	T	C	Accurate
Marker2381	LG04	30.47	Chr. 4	4733919	A	G	A	G	A	Accurate
Marker2388	LG04	30.47	Chr. 4	4821668	G	T	G	T	G	Accurate
Marker2396	LG04	33.27	Chr. 4	5647715	C	T	C	T	C	Accurate
Marker2410	LG04	34.88	Chr. 4	5809870	C	T	C	T	N	Uncertain
Marker2403	LG04	37.57	Chr. 4	5739175	A	C	A	C	A	Accurate
Marker2406	LG04	38.11	Chr. 4	5798974	C	T	C	T	C	Accurate
Marker2407	LG04	38.65	Chr. 4	5798978	G	T	G	T	G	Accurate
Marker2562	LG04	40.26	Chr. 4	14167581	T	C	T	C	T	Accurate
Marker2404	LG04	41.34	Chr. 4	5761952	A	G	A	G	A	Accurate
Marker2530	LG04	41.34	Chr. 4	12112100	A	G	A	G	A	Accurate
Marker7848	LG04	41.34	Chr. 0	14824888	A	G	A	G	A	Accurate
Marker7861	LG04	41.34	Chr. 0	14930604	A	G	A	G	A	Accurate
Marker7847	LG04	41.87	Chr. 0	14824614	T	C	T	C	T	Accurate
Marker2467	LG04	41.87	Chr. 4	8077543	C	T	C	T	C	Accurate
Marker2506	LG04	42.95	Chr. 4	9872738	G	A	G	A	G	Accurate
Marker2498	LG04	43.49	Chr. 4	9813274	C	A	C	A	C	Accurate
Marker2508	LG04	43.49	Chr. 4	10023123	C	C	A	C	A	Accurate
Marker2499	LG04	44.56	Chr. 4	9823258	T	C	T	C	T	Accurate
Marker2536	LG04	44.56	Chr. 4	12186954	A	C	A	C	A	Accurate
Marker7787	LG04	44.56	Chr. 0	9632136	G	A	G	R	G	Uncertain
Marker7856	LG04	45.10	Chr. 0	14880025	T	C	T	C	T	Accurate
Marker7858	LG04	45.10	Chr. 0	14880319	T	C	T	C	T	Accurate
Marker2547	LG04	46.17	Chr. 4	13025409	A	G	A	G	A	Accurate
Marker2521	LG04	46.71	Chr. 4	12005594	T	C	T	C	T	Accurate
Marker2431	LG04	47.79	Chr. 4	7712004	G	A	G	A	G	Accurate
Marker2405	LG04	48.86	Chr. 4	5795448	C	T	C	T	C	Accurate
Marker2466	LG04	49.40	Chr. 4	8074941	C	T	C	T	C	Accurate
Marker7910	LG04	49.40	Chr. 0	17633578	G	C	G	C	G	Accurate
Marker7911	LG04	49.40	Chr. 0	17633607	T	C	T	C	T	Accurate
Marker7784	LG04	49.94	Chr. 0	9613467	G	T	G	T	G	Accurate
Marker2432	LG04	49.94	Chr. 4	7712914	A	G	A	G	A	Accurate
Marker2435	LG04	49.94	Chr. 4	7802650	A	G	A	G	A	Accurate
Marker2526	LG04	49.94	Chr. 4	12076054	T	G	T	G	T	Accurate
Marker2546	LG04	49.94	Chr. 4	13024010	C	T	C	T	C	Accurate
Marker2436	LG04	50.48	Chr. 4	7809167	T	C	T	C	T	Accurate
Marker7771	LG04	51.01	Chr. 0	7705249	G	A	G	A	G	Accurate
Marker7823	LG04	51.01	Chr. 0	13130115	G	A	G	A	G	Accurate
Marker2422	LG04	51.01	Chr. 4	7672833	A	G	A	G	A	Accurate
Marker2469	LG04	51.01	Chr. 4	8080867	T	C	T	C	T	Accurate
Marker2550	LG04	51.01	Chr. 4	13692007	G	C	G	C	G	Accurate
Marker2461	LG04	51.55	Chr. 4	8027342	T	C	T	C	T	Accurate
Marker7855	LG04	52.09	Chr. 0	14865553	G	A	G	A	G	Accurate

Marker7859	LG04	52.09	Chr. 0	14908601	T	C	T	C	T	Accurate
Marker7868	LG04	52.09	Chr. 0	14952381	C	T	C	T	C	Accurate
Marker7773	LG04	52.63	Chr. 0	8045477	G	T	G	T	G	Accurate
Marker2414	LG04	52.63	Chr. 4	7487067	G	C	G	C	G	Accurate
Marker2459	LG04	52.63	Chr. 4	8018540	T	C	T	C	T	Accurate
Marker2442	LG04	53.39	Chr. 4	7901692	G	C	G	C	G	Accurate
Marker2501	LG04	53.39	Chr. 4	9824766	C	T	C	T	C	Accurate
Marker2531	LG04	53.39	Chr. 4	12112109	C	G	C	G	C	Accurate
Marker2527	LG04	53.93	Chr. 4	12076552	A	G	A	G	A	Accurate
Marker2412	LG04	54.47	Chr. 4	7425666	G	A	G	A	G	Accurate
Marker2413	LG04	54.47	Chr. 4	7466995	A	G	A	G	A	Accurate
Marker2417	LG04	54.47	Chr. 4	7553187	C	T	C	T	C	Accurate
Marker2421	LG04	54.47	Chr. 4	7582451	C	A	C	A	C	Accurate
Marker2428	LG04	54.47	Chr. 4	7684116	A	G	A	G	A	Accurate
Marker2433	LG04	54.47	Chr. 4	7713126	C	T	C	T	C	Accurate
Marker2434	LG04	54.47	Chr. 4	7790359	A	G	A	G	A	Accurate
Marker2443	LG04	54.47	Chr. 4	7919460	T	C	T	C	T	Accurate
Marker2523	LG04	54.47	Chr. 4	12034376	G	A	G	A	G	Accurate
Marker2534	LG04	54.47	Chr. 4	12139517	G	A	G	A	G	Accurate
Marker2549	LG04	54.47	Chr. 4	13677132	G	A	G	A	G	Accurate
Marker2554	LG04	54.47	Chr. 4	13711500	T	C	T	C	T	Accurate
Marker7913	LG04	54.47	Chr. 0	17638002	C	A	C	A	C	Accurate
Marker7914	LG04	54.47	Chr. 0	17646895	G	A	G	A	G	Accurate
Marker7841	LG04	54.47	Chr. 0	14792187	C	T	C	T	C	Accurate
Marker7866	LG04	54.47	Chr. 0	14944542	T	C	T	C	T	Accurate
Marker7846	LG04	55.00	Chr. 0	14816322	C	A	C	A	C	Accurate
Marker7840	LG04	55.54	Chr. 0	14675380	T	C	T	C	T	Accurate
Marker7862	LG04	55.54	Chr. 0	14930618	A	T	A	T	A	Accurate
Marker2439	LG04	55.54	Chr. 4	7825872	A	G	A	G	A	Accurate
Marker2441	LG04	55.54	Chr. 4	7899682	T	C	T	C	T	Accurate
Marker2438	LG04	56.62	Chr. 4	7825692	C	T	C	T	C	Accurate
Marker2564	LG04	57.69	Chr. 4	14201874	A	G	A	G	A	Accurate
Marker2558	LG04	59.53	Chr. 4	14154294	A	G	A	G	A	Accurate
Marker2556	LG04	60.60	Chr. 4	14148315	T	C	T	C	T	Accurate
Marker2557	LG04	60.60	Chr. 4	14153634	A	G	A	G	A	Accurate
Marker2477	LG04	63.29	Chr. 4	9259715	C	C	T	C	Y	Uncertain
Marker2479	LG04	63.29	Chr. 4	9263505	C	C	T	C	T	Accurate
Marker2492	LG04	63.29	Chr. 4	9629053	T	C	T	C	T	Accurate
Marker2493	LG04	64.90	Chr. 4	9636798	A	G	A	G	A	Accurate
Marker2504	LG04	65.98	Chr. 4	9863745	C	T	C	T	C	Accurate
Marker2474	LG04	67.06	Chr. 4	9139247	A	A	G	A	G	Accurate
Marker2478	LG04	67.59	Chr. 4	9260219	A	A	G	R	G	Uncertain
Marker2475	LG04	68.35	Chr. 4	9139989	C	C	T	C	T	Accurate
Marker2473	LG04	69.97	Chr. 4	9127084	C	T	C	T	C	Accurate
Marker2540	LG04	72.34	Chr. 4	12383426	T	T	C	T	C	Accurate
Marker2541	LG04	72.88	Chr. 4	12422055	A	A	C	A	C	Accurate
Marker2544	LG04	74.49	Chr. 4	12422555	T	T	C	T	C	Accurate

Marker2543	LG04	75.56	Chr. 4	12422264	A	A	G	A	G	Accurate
Marker2538	LG04	77.18	Chr. 4	12340929	G	G	A	G	A	Accurate
Marker2539	LG04	77.72	Chr. 4	12341208	C	C	G	C	G	Accurate
Marker2574	LG04	81.59	Chr. 4	15569673	A	A	G	A	G	Accurate
Marker2575	LG04	82.13	Chr. 4	15569991	T	T	G	T	G	Accurate
Marker2572	LG04	82.67	Chr. 4	15564712	A	A	G	A	G	Accurate
Marker2576	LG04	83.75	Chr. 4	15575972	C	C	G	C	G	Accurate
Marker2570	LG04	86.54	Chr. 4	15534416	T	T	A	T	A	Accurate
Marker2568	LG04	87.62	Chr. 4	15534382	G	G	A	G	A	Accurate
Marker2569	LG04	88.16	Chr. 4	15534385	T	T	C	T	C	Accurate
Marker2567	LG04	89.88	Chr. 4	15424876	C	C	A	C	A	Accurate
Marker2579	LG04	92.78	Chr. 4	15696758	C	C	T	C	T	Accurate
Marker2580	LG04	95.81	Chr. 4	15963043	T	T	G	T	G	Accurate
Marker2590	LG04	99.25	Chr. 4	16264147	A	A	G	A	G	Accurate
Marker2589	LG04	100.33	Chr. 4	16263914	T	T	C	T	C	Accurate
Marker2592	LG04	101.40	Chr. 4	16264210	C	C	T	C	T	Accurate
Marker2591	LG04	101.94	Chr. 4	16264179	A	A	C	A	C	Accurate
Marker2598	LG04	102.48	Chr. 4	16331383	G	G	A	G	A	Accurate
Marker2593	LG04	103.02	Chr. 4	16290809	C	C	T	C	T	Accurate
Marker2595	LG04	103.02	Chr. 4	16294810	A	A	C	A	C	Accurate
Marker2597	LG04	104.09	Chr. 4	16303240	T	T	G	T	G	Accurate
Marker2588	LG04	105.81	Chr. 4	16262755	A	A	G	A	G	Accurate
Marker2601	LG04	108.72	Chr. 4	16926507	G	G	A	G	A	Accurate
Marker2603	LG04	111.63	Chr. 4	17039560	G	G	C	G	C	Accurate
Marker2604	LG04	112.70	Chr. 4	17339243	C	C	A	C	A	Accurate
Marker2600	LG04	114.32	Chr. 4	16851480	A	A	G	A	G	Accurate
Marker2608	LG04	117.65	Chr. 4	17649157	G	G	A	G	A	Accurate
Marker2617	LG04	119.81	Chr. 4	18179935	G	G	A	G	A	Accurate
Marker2616	LG04	120.34	Chr. 4	18178387	G	A	G	A	G	Accurate
Marker2628	LG04	123.03	Chr. 4	18563926	G	G	A	G	A	Accurate
Marker2630	LG04	125.19	Chr. 4	18586732	A	A	T	A	T	Accurate
Marker2625	LG04	125.72	Chr. 4	18479253	T	T	C	T	C	Accurate
Marker2635	LG04	126.80	Chr. 4	18755094	A	C	A	C	A	Accurate
Marker2637	LG04	126.80	Chr. 4	18785752	C	T	C	T	C	Accurate
Marker2644	LG04	126.80	Chr. 4	18845134	A	G	A	G	A	Accurate
Marker2633	LG04	127.34	Chr. 4	18738210	T	T	C	T	C	Accurate
Marker2643	LG04	127.34	Chr. 4	18809203	C	T	C	T	C	Accurate
Marker2627	LG04	128.41	Chr. 4	18563212	G	G	T	G	T	Accurate
Marker2645	LG04	128.95	Chr. 4	19044390	C	C	A	C	A	Accurate
Marker2647	LG04	128.95	Chr. 4	19061444	C	C	T	C	T	Accurate
Marker2651	LG04	129.49	Chr. 4	19222101	A	A	G	A	G	Accurate
Marker2652	LG04	131.10	Chr. 4	19228742	T	T	C	T	C	Accurate
Marker2657	LG04	131.64	Chr. 4	19353342	G	T	G	T	G	Accurate
Marker2653	LG04	132.71	Chr. 4	19229622	G	G	A	G	A	Accurate
Marker2656	LG04	133.79	Chr. 4	19331804	A	G	A	G	A	Accurate
Marker2654	LG04	134.86	Chr. 4	19255754	C	C	G	C	G	Accurate
Marker2663	LG04	135.94	Chr. 4	19655107	C	C	G	C	S	Uncertain

Marker2658	LG04	137.02	Chr. 4	19534675	C	C	T	C	T	Accurate
Marker2680	LG04	142.03	Chr. 4	20765862	G	G	T	G	T	Accurate
Marker2683	LG04	143.10	Chr. 4	20849618	T	T	C	T	C	Accurate
Marker2678	LG04	145.75	Chr. 4	20746891	C	C	T	C	T	Accurate
Marker2685	LG04	148.01	Chr. 4	20912899	T	T	C	T	C	Accurate
Marker2686	LG04	154.37	Chr. 4	21643021	T	C	T	C	T	Accurate
Marker2700	LG04	156.21	Chr. 4	22457399	C	C	T	C	T	Accurate
Marker2701	LG04	158.59	Chr. 4	22765333	C	C	T	C	T	Accurate
Marker2705	LG04	164.86	Chr. 4	23122351	T	C	T	C	T	Accurate
Marker2707	LG04	165.94	Chr. 4	23143506	A	T	A	T	A	Accurate
Marker2706	LG04	167.01	Chr. 4	23127973	G	A	G	A	G	Accurate
Marker2710	LG04	168.09	Chr. 4	23650216	A	A	G	A	G	Accurate
Marker2716	LG04	168.63	Chr. 4	23732381	G	G	A	G	A	Accurate
Marker2717	LG04	168.63	Chr. 4	23760617	G	G	A	G	A	Accurate
Marker2718	LG04	169.17	Chr. 4	23774249	C	C	T	C	T	Accurate
Marker2722	LG04	169.17	Chr. 4	23837146	C	C	T	C	T	Accurate
Marker2720	LG04	170.24	Chr. 4	23828001	T	T	C	T	C	Accurate
Marker2730	LG05	0.00	Chr. 5	400986	T	T	G	T	G	Accurate
Marker2737	LG05	0.54	Chr. 5	454672	C	C	G	C	G	Accurate
Marker2738	LG05	1.08	Chr. 5	643167	C	G	C	G	C	Accurate
Marker2746	LG05	1.08	Chr. 5	1212460	C	T	C	T	C	Accurate
Marker2753	LG05	1.08	Chr. 5	1313667	G	A	G	A	G	Accurate
Marker2736	LG05	1.61	Chr. 5	421342	C	C	G	C	G	Accurate
Marker2745	LG05	1.61	Chr. 5	1025005	A	G	A	G	A	Accurate
Marker2748	LG05	1.61	Chr. 5	1229834	A	T	A	T	A	Accurate
Marker2752	LG05	1.61	Chr. 5	1250697	G	C	G	C	G	Accurate
Marker2757	LG05	1.61	Chr. 5	1331928	T	C	T	C	T	Accurate
Marker2750	LG05	2.15	Chr. 5	1248827	C	T	C	T	C	Accurate
Marker2758	LG05	2.69	Chr. 5	1338343	T	C	T	C	T	Accurate
Marker2768	LG05	2.69	Chr. 5	1440980	A	C	A	C	A	Accurate
Marker2772	LG05	2.69	Chr. 5	1481748	A	G	A	G	A	Accurate
Marker2751	LG05	3.23	Chr. 5	1250670	C	T	C	T	C	Accurate
Marker2762	LG05	3.23	Chr. 5	1350101	C	T	C	T	C	Accurate
Marker2764	LG05	3.23	Chr. 5	1369153	C	T	C	T	C	Accurate
Marker2766	LG05	3.76	Chr. 5	1373920	G	T	G	T	G	Accurate
Marker2765	LG05	4.30	Chr. 5	1373906	G	A	G	A	G	Accurate
Marker2775	LG05	4.84	Chr. 5	1496094	C	T	C	T	C	Accurate
Marker2783	LG05	4.84	Chr. 5	1651346	A	A	G	A	G	Accurate
Marker2790	LG05	4.84	Chr. 5	1726778	C	T	C	T	C	Accurate
Marker2773	LG05	5.91	Chr. 5	1495857	C	T	C	T	C	Accurate
Marker2776	LG05	5.91	Chr. 5	1635079	G	A	G	A	G	Accurate
Marker2784	LG05	5.91	Chr. 5	1651360	C	C	T	C	T	Accurate
Marker2793	LG05	5.91	Chr. 5	1780714	G	G	T	G	T	Accurate
Marker2792	LG05	6.99	Chr. 5	1767589	T	T	C	T	C	Accurate
Marker2788	LG05	9.68	Chr. 5	1723453	T	G	T	G	T	Accurate
Marker2799	LG05	10.76	Chr. 5	1923535	G	A	G	A	G	Accurate
Marker2795	LG05	11.29	Chr. 5	1811197	T	C	T	C	T	Accurate

Marker2794	LG05	12.91	Chr. 5	1810995	A	G	A	G	A	Accurate
Marker2798	LG05	16.14	Chr. 5	1891404	C	T	C	T	C	Accurate
Marker2806	LG05	22.28	Chr. 5	2616760	A	G	A	G	A	Accurate
Marker2810	LG05	22.81	Chr. 5	2630932	T	A	T	A	T	Accurate
Marker2814	LG05	25.94	Chr. 5	3516108	C	T	C	T	C	Accurate
Marker2817	LG05	28.32	Chr. 5	3559197	C	T	C	T	C	Accurate
Marker2816	LG05	30.28	Chr. 5	3528976	T	C	T	C	T	Accurate
Marker2815	LG05	32.65	Chr. 5	3528957	T	C	T	C	T	Accurate
Marker2833	LG05	35.88	Chr. 5	4953868	G	A	G	A	G	Accurate
Marker2836	LG05	36.95	Chr. 5	5059349	G	A	G	A	G	Accurate
Marker2841	LG05	36.95	Chr. 5	5112133	A	G	A	G	A	Accurate
Marker2850	LG05	36.95	Chr. 5	5183193	A	G	A	G	A	Accurate
Marker2838	LG05	37.49	Chr. 5	5060636	T	C	T	C	T	Accurate
Marker2843	LG05	37.49	Chr. 5	5127444	G	C	G	C	G	Accurate
Marker2834	LG05	38.03	Chr. 5	5039472	G	A	G	A	G	Accurate
Marker2837	LG05	38.03	Chr. 5	5060614	C	A	C	A	C	Accurate
Marker2851	LG05	38.03	Chr. 5	5218817	G	A	G	A	G	Accurate
Marker2856	LG05	38.03	Chr. 5	5574115	A	G	A	G	A	Accurate
Marker2830	LG05	38.57	Chr. 5	4950475	T	C	T	C	T	Accurate
Marker2840	LG05	38.57	Chr. 5	5070314	T	C	T	C	T	Accurate
Marker2847	LG05	38.57	Chr. 5	5167878	C	G	C	G	C	Accurate
Marker2848	LG05	38.57	Chr. 5	5175565	C	A	C	A	C	Accurate
Marker2831	LG05	39.10	Chr. 5	4950489	T	C	T	C	T	Accurate
Marker2842	LG05	40.18	Chr. 5	5126634	C	T	C	T	C	Accurate
Marker2825	LG05	40.72	Chr. 5	4567473	G	A	G	A	G	Accurate
Marker2827	LG05	41.25	Chr. 5	4567705	T	C	T	C	T	Accurate
Marker2824	LG05	41.80	Chr. 5	4567455	A	T	A	T	A	Accurate
Marker2846	LG05	42.34	Chr. 5	5158671	A	G	A	G	A	Accurate
Marker2859	LG05	42.34	Chr. 5	5582811	T	A	T	A	T	Accurate
Marker2845	LG05	43.63	Chr. 5	5158640	A	G	A	G	A	Accurate
Marker2867	LG05	44.71	Chr. 5	6340249	T	C	T	C	T	Accurate
Marker2864	LG05	46.00	Chr. 5	5997033	G	A	G	A	G	Accurate
Marker2866	LG05	49.46	Chr. 5	6284045	T	C	T	C	T	Accurate
Marker2868	LG05	51.07	Chr. 5	7444478	T	C	T	C	T	Accurate
Marker2884	LG05	51.61	Chr. 5	8855355	A	G	A	G	A	Accurate
Marker2883	LG05	53.44	Chr. 5	8855205	A	G	A	G	A	Accurate
Marker2882	LG05	55.11	Chr. 5	8840673	C	T	C	T	C	Accurate
Marker2876	LG05	56.21	Chr. 5	8715560	C	G	C	G	C	Accurate
Marker2875	LG05	57.63	Chr. 5	8715552	G	A	G	A	G	Accurate
Marker2881	LG05	58.39	Chr. 5	8820821	C	T	C	T	C	Accurate
Marker2869	LG05	58.39	Chr. 5	8465860	C	T	C	T	C	Accurate
Marker2871	LG05	59.46	Chr. 5	8496548	G	A	C	T	T	Inconsistent
Marker2904	LG05	62.16	Chr. 5	9895636	C	T	C	T	C	Accurate
Marker2870	LG05	63.23	Chr. 5	8489845	A	A	G	A	G	Accurate
Marker2903	LG05	64.84	Chr. 5	9895418	A	G	A	G	A	Accurate
Marker2914	LG05	65.92	Chr. 5	10255172	G	A	G	A	G	Accurate
Marker2898	LG05	66.46	Chr. 5	9863465	G	G	T	G	T	Accurate

Marker2893	LG05	67.00	Chr. 5	9837539	G	A	G	A	G	Accurate
Marker2899	LG05	67.00	Chr. 5	9864793	A	G	A	G	A	Accurate
Marker2912	LG05	67.00	Chr. 5	9906955	G	T	G	T	G	Accurate
Marker2910	LG05	68.07	Chr. 5	9901919	G	A	G	A	G	Accurate
Marker2907	LG05	69.15	Chr. 5	9899011	A	G	A	G	A	Accurate
Marker2908	LG05	70.76	Chr. 5	9899365	G	A	G	A	G	Accurate
Marker2953	LG05	73.99	Chr. 5	11481260	A	T	A	T	A	Accurate
Marker2958	LG05	73.99	Chr. 5	11615810	T	T	C	T	C	Accurate
Marker2965	LG05	73.99	Chr. 5	11633108	C	C	T	C	T	Accurate
Marker2935	LG05	74.52	Chr. 5	11265660	A	A	G	A	G	Accurate
Marker2949	LG05	75.06	Chr. 5	11413231	G	A	G	A	G	Accurate
Marker2957	LG05	75.06	Chr. 5	11615808	T	T	C	T	C	Accurate
Marker2971	LG05	75.06	Chr. 5	11691502	T	C	T	C	T	Accurate
Marker2926	LG05	75.60	Chr. 5	10964855	G	A	G	A	G	Accurate
Marker2939	LG05	75.60	Chr. 5	11347799	G	A	G	A	G	Accurate
Marker2956	LG05	75.60	Chr. 5	11561770	G	C	G	C	G	Accurate
Marker2941	LG05	76.14	Chr. 5	11359095	T	T	G	T	G	Accurate
Marker2946	LG05	77.75	Chr. 5	11397042	A	A	T	A	T	Accurate
Marker2982	LG05	78.51	Chr. 5	12716396	G	A	G	A	G	Accurate
Marker2990	LG05	78.51	Chr. 5	12860243	C	T	C	T	C	Accurate
Marker2952	LG05	79.05	Chr. 5	11479875	C	T	C	T	C	Accurate
Marker2999	LG05	80.66	Chr. 5	12923297	G	G	C	G	C	Accurate
Marker2981	LG05	82.81	Chr. 5	12694507	A	G	A	G	A	Accurate
Marker2986	LG05	83.35	Chr. 5	12725441	T	C	T	C	T	Accurate
Marker2998	LG05	83.89	Chr. 5	12918636	C	C	A	C	A	Accurate
Marker3008	LG05	84.96	Chr. 5	15359707	C	A	C	A	C	Accurate
Marker3009	LG05	85.50	Chr. 5	15435085	T	T	C	T	C	Accurate
Marker3010	LG05	86.04	Chr. 5	15435119	C	C	G	C	G	Accurate
Marker3012	LG05	86.04	Chr. 5	15455844	A	G	A	G	A	Accurate
Marker3015	LG05	86.58	Chr. 5	15456510	C	T	C	T	C	Accurate
Marker3016	LG05	87.65	Chr. 5	15500183	C	G	C	G	C	Accurate
Marker3005	LG05	89.26	Chr. 5	15220062	C	T	C	T	C	Accurate
Marker3020	LG05	90.88	Chr. 5	15968805	T	T	C	T	C	Accurate
Marker3023	LG05	90.88	Chr. 5	16095539	T	T	G	N	G	Uncertain
Marker3011	LG05	91.42	Chr. 5	15443182	A	A	G	A	G	Accurate
Marker3021	LG05	92.49	Chr. 5	15979028	C	C	A	C	A	Accurate
Marker3029	LG05	93.03	Chr. 5	16550483	C	C	T	C	T	Accurate
Marker3030	LG05	93.57	Chr. 5	16550665	G	G	A	G	A	Accurate
Marker3034	LG05	94.10	Chr. 5	16625707	G	G	T	G	T	Accurate
Marker3036	LG05	94.10	Chr. 5	16666642	A	A	G	A	G	Accurate
Marker3044	LG05	94.10	Chr. 5	16701267	C	C	T	C	T	Accurate
Marker3048	LG05	94.10	Chr. 5	16807920	G	A	G	A	G	Accurate
Marker3053	LG05	94.10	Chr. 5	16825425	G	A	G	A	G	Accurate
Marker3055	LG05	94.10	Chr. 5	16860113	T	C	T	C	T	Accurate
Marker3031	LG05	94.64	Chr. 5	16557448	T	T	C	T	C	Accurate
Marker3032	LG05	94.64	Chr. 5	16593345	G	G	A	G	A	Accurate
Marker3035	LG05	94.64	Chr. 5	16654057	C	C	T	C	T	Accurate

Marker3037	LG05	94.64	Chr. 5	16666848	C	C	T	C	T	Accurate
Marker3050	LG05	94.64	Chr. 5	16816440	C	A	C	A	C	Accurate
Marker3040	LG05	95.18	Chr. 5	16696334	A	A	G	A	G	Accurate
Marker3059	LG05	96.79	Chr. 5	16904509	C	G	C	G	C	Accurate
Marker3057	LG05	97.87	Chr. 5	16899225	T	C	T	C	T	Accurate
Marker3049	LG05	99.48	Chr. 5	16811904	T	A	T	A	T	Accurate
Marker3126	LG05	103.58	Chr. 5	21567280	C	T	C	T	C	Accurate
Marker3054	LG05	104.66	Chr. 5	16856606	G	A	G	A	G	Accurate
Marker3093	LG05	106.27	Chr. 5	21056898	G	A	G	A	G	Accurate
Marker3095	LG05	108.42	Chr. 5	21083890	A	T	A	T	A	Accurate
Marker3091	LG05	108.96	Chr. 5	21030201	T	C	T	C	T	Accurate
Marker3102	LG05	109.50	Chr. 5	21217632	A	G	A	G	A	Accurate
Marker3090	LG05	110.04	Chr. 5	21030195	A	G	A	G	N	Uncertain
Marker3123	LG05	111.11	Chr. 5	21474141	T	G	T	G	T	Accurate
Marker3092	LG05	111.65	Chr. 5	21030349	A	G	A	G	A	Accurate
Marker3088	LG05	112.19	Chr. 5	21029865	C	T	C	T	C	Accurate
Marker3117	LG05	112.19	Chr. 5	21318689	C	T	C	T	C	Accurate
Marker3073	LG05	112.72	Chr. 5	20437277	A	T	A	T	A	Accurate
Marker3076	LG05	112.72	Chr. 5	20458492	T	C	T	C	T	Accurate
Marker3096	LG05	112.72	Chr. 5	21084510	T	C	T	C	T	Accurate
Marker3098	LG05	112.72	Chr. 5	21175743	G	T	G	T	G	Accurate
Marker3101	LG05	112.72	Chr. 5	21202207	T	C	T	C	T	Accurate
Marker3109	LG05	112.72	Chr. 5	21264627	G	C	G	C	G	Accurate
Marker3116	LG05	112.72	Chr. 5	21305296	T	G	T	G	T	Accurate
Marker3119	LG05	112.72	Chr. 5	21353094	T	C	T	C	T	Accurate
Marker3129	LG05	112.72	Chr. 5	21572034	G	C	G	C	G	Accurate
Marker3133	LG05	112.72	Chr. 5	21699906	T	C	T	C	T	Accurate
Marker3134	LG05	112.72	Chr. 5	21718269	C	T	C	T	C	Accurate
Marker3066	LG05	112.72	Chr. 5	20314286	T	C	T	C	T	Accurate
Marker3068	LG05	112.72	Chr. 5	20361087	G	A	G	A	G	Accurate
Marker3081	LG05	113.26	Chr. 5	20587538	G	A	G	A	G	Accurate
Marker3099	LG05	113.26	Chr. 5	21181114	C	T	C	T	C	Accurate
Marker3113	LG05	113.26	Chr. 5	21294636	G	C	G	C	G	Accurate
Marker3115	LG05	113.26	Chr. 5	21303135	T	C	T	C	T	Accurate
Marker3118	LG05	113.26	Chr. 5	21348479	G	A	G	A	G	Accurate
Marker3120	LG05	113.26	Chr. 5	21362267	A	G	A	G	A	Accurate
Marker3079	LG05	113.80	Chr. 5	20569162	T	G	T	G	T	Accurate
Marker3089	LG05	113.80	Chr. 5	21030107	T	C	T	C	T	Accurate
Marker3104	LG05	113.80	Chr. 5	21227383	G	A	G	A	G	Accurate
Marker3114	LG05	113.80	Chr. 5	21302940	G	A	G	A	G	Accurate
Marker3111	LG05	114.34	Chr. 5	21268992	T	A	T	A	T	Accurate
Marker3084	LG05	114.88	Chr. 5	21023947	C	T	C	T	C	Accurate
Marker3112	LG05	114.88	Chr. 5	21278208	T	C	T	C	T	Accurate
Marker3097	LG05	115.95	Chr. 5	21162788	G	A	G	A	G	Accurate
Marker3151	LG05	115.95	Chr. 5	22322727	C	T	C	T	C	Accurate
Marker3169	LG05	115.95	Chr. 5	22965799	A	G	A	G	A	Accurate
Marker3152	LG05	116.49	Chr. 5	22322768	C	T	C	T	C	Accurate

Marker3154	LG05	116.49	Chr. 5	22375647	C	G	C	G	C	Accurate
Marker3157	LG05	117.03	Chr. 5	22588582	G	A	G	A	G	Accurate
Marker3168	LG05	118.10	Chr. 5	22809770	T	C	T	C	T	Accurate
Marker3164	LG05	119.18	Chr. 5	22761738	T	C	T	C	T	Accurate
Marker3166	LG05	119.18	Chr. 5	22768142	C	T	C	T	C	Accurate
Marker3146	LG05	119.71	Chr. 5	22315945	A	G	A	G	A	Accurate
Marker3160	LG05	119.71	Chr. 5	22713946	C	A	C	A	C	Accurate
Marker3165	LG05	119.71	Chr. 5	22762018	C	T	C	T	C	Accurate
Marker3167	LG05	121.97	Chr. 5	22768196	G	A	G	A	G	Accurate
Marker3171	LG05	122.51	Chr. 5	22982162	C	T	C	T	C	Accurate
Marker3178	LG05	123.05	Chr. 5	23394138	G	A	G	A	G	Accurate
Marker3180	LG05	123.05	Chr. 5	23428550	T	C	T	C	T	Accurate
Marker3181	LG05	123.05	Chr. 5	23434693	A	G	A	G	A	Accurate
Marker3172	LG05	123.59	Chr. 5	22982400	A	C	A	C	A	Accurate
Marker3176	LG05	124.12	Chr. 5	23381728	A	G	A	G	A	Accurate
Marker3175	LG05	124.88	Chr. 5	23381564	C	T	C	T	C	Accurate
Marker3177	LG05	127.49	Chr. 5	23381734	C	T	C	T	C	Accurate
Marker3194	LG05	131.91	Chr. 5	24670786	C	T	C	T	C	Accurate
Marker3195	LG05	135.24	Chr. 5	24680407	C	T	C	T	C	Accurate
Marker3212	LG05	138.58	Chr. 5	25766483	T	C	T	C	T	Accurate
Marker3217	LG05	139.12	Chr. 5	25847075	A	C	A	C	A	Accurate
Marker3216	LG05	140.20	Chr. 5	25846991	G	A	G	A	G	Accurate
Marker3228	LG05	141.27	Chr. 5	26159430	A	A	C	A	C	Accurate
Marker3220	LG05	141.81	Chr. 5	26007106	C	C	T	C	T	Accurate
Marker3227	LG05	142.88	Chr. 5	26127389	T	T	G	T	G	Accurate
Marker3225	LG05	143.96	Chr. 5	26026945	A	A	G	A	G	Accurate
Marker3262	LG05	143.96	Chr. 5	26486183	G	A	G	A	G	Accurate
Marker3261	LG05	144.50	Chr. 5	26486180	C	A	C	A	C	Accurate
Marker3224	LG05	145.03	Chr. 5	26026777	A	A	C	A	C	Accurate
Marker3237	LG05	146.11	Chr. 5	26175653	T	T	C	T	C	Accurate
Marker3223	LG05	146.65	Chr. 5	26019850	T	T	C	T	C	Accurate
Marker3246	LG05	146.65	Chr. 5	26373940	C	C	T	C	T	Accurate
Marker3245	LG05	147.72	Chr. 5	26373867	C	C	T	C	T	Accurate
Marker3254	LG05	148.48	Chr. 5	26432296	T	T	G	T	G	Accurate
Marker3257	LG05	149.02	Chr. 5	26445167	A	A	G	A	G	Accurate
Marker3256	LG05	149.78	Chr. 5	26445129	G	G	A	G	A	Accurate
Marker3272	LG05	153.12	Chr. 5	26769280	C	C	T	N	T	Uncertain
Marker3293	LG05	155.08	Chr. 5	28722913	A	G	A	G	A	Accurate
Marker3287	LG05	156.15	Chr. 5	28672357	T	G	T	G	T	Accurate
Marker3289	LG05	156.91	Chr. 5	28708207	G	A	G	A	G	Accurate
Marker3290	LG05	159.82	Chr. 5	28708385	C	A	C	A	C	Accurate
Marker3274	LG05	160.36	Chr. 5	26797724	A	A	T	A	T	Accurate
Marker3304	LG05	164.35	Chr. 5	29622714	G	G	A	G	A	Accurate
Marker3298	LG05	165.42	Chr. 5	29607595	C	C	T	C	T	Accurate
Marker3297	LG05	166.85	Chr. 5	29607565	A	A	G	A	G	Accurate
Marker3299	LG05	167.60	Chr. 5	29609302	G	G	T	G	T	Accurate
Marker3300	LG05	169.22	Chr. 5	29609529	T	T	C	T	C	Accurate

Marker3309	LG05	172.13	Chr. 5	32052416	G	A	G	A	G	Accurate
Marker3301	LG05	173.21	Chr. 5	29609830	A	A	G	A	G	Accurate
Marker3310	LG05	174.28	Chr. 5	32147524	A	A	G	A	G	Accurate
Marker3313	LG05	176.12	Chr. 5	32296268	C	T	C	T	C	Accurate
Marker3311	LG05	177.73	Chr. 5	32245215	G	G	T	G	T	Accurate
Marker3320	LG05	178.80	Chr. 5	32463962	T	T	C	T	C	Accurate
Marker3322	LG05	179.88	Chr. 5	32616263	T	T	A	T	A	Accurate
Marker3332	LG05	179.88	Chr. 5	32809232	C	T	C	T	C	Accurate
Marker3331	LG05	180.42	Chr. 5	32809162	C	T	C	T	C	Accurate
Marker3343	LG05	180.42	Chr. 5	33042731	G	G	T	G	T	Accurate
Marker3340	LG05	180.96	Chr. 5	33031221	G	G	A	G	A	Accurate
Marker3359	LG05	180.96	Chr. 5	33255748	T	T	C	T	C	Accurate
Marker3339	LG05	182.25	Chr. 5	33013298	C	C	A	C	A	Accurate
Marker3342	LG05	182.25	Chr. 5	33033956	A	A	G	A	G	Accurate
Marker3348	LG05	182.25	Chr. 5	33079841	T	T	C	T	C	Accurate
Marker3351	LG05	182.25	Chr. 5	33097506	A	A	G	A	G	Accurate
Marker3352	LG05	182.25	Chr. 5	33103672	G	G	A	G	A	Accurate
Marker3353	LG05	182.25	Chr. 5	33149001	A	A	G	A	G	Accurate
Marker3356	LG05	182.25	Chr. 5	33235350	T	T	C	T	C	Accurate
Marker3358	LG05	182.25	Chr. 5	33249777	T	T	C	T	C	Accurate
Marker3374	LG05	182.25	Chr. 5	33683087	G	A	G	A	G	Accurate
Marker3379	LG05	182.25	Chr. 5	33714684	G	C	G	C	G	Accurate
Marker3355	LG05	182.79	Chr. 5	33163317	C	C	G	C	G	Accurate
Marker3360	LG05	182.79	Chr. 5	33322007	G	G	C	G	C	Accurate
Marker3363	LG05	182.79	Chr. 5	33407700	A	A	G	A	G	Accurate
Marker3365	LG05	182.79	Chr. 5	33428959	C	C	T	C	T	Accurate
Marker3370	LG05	182.79	Chr. 5	33541793	A	G	A	G	A	Accurate
Marker3372	LG05	182.79	Chr. 5	33583176	T	A	T	A	T	Accurate
Marker3373	LG05	182.79	Chr. 5	33652143	T	C	T	C	T	Accurate
Marker3375	LG05	182.79	Chr. 5	33703279	C	T	C	T	C	Accurate
Marker3383	LG06	0.00	Chr. 6	302928	G	A	G	A	G	Accurate
Marker3398	LG06	0.00	Chr. 6	842914	G	T	G	T	G	Accurate
Marker3412	LG06	0.00	Chr. 6	1619519	T	A	T	A	T	Accurate
Marker3462	LG06	2.61	Chr. 6	3396922	C	T	C	T	C	Accurate
Marker3411	LG06	3.68	Chr. 6	1573823	T	G	T	G	T	Accurate
Marker3469	LG06	4.22	Chr. 6	3952873	A	G	A	G	A	Accurate
Marker3459	LG06	6.59	Chr. 6	3314960	T	G	T	G	T	Accurate
Marker3480	LG06	8.75	Chr. 6	4403759	A	A	C	A	C	Accurate
Marker3479	LG06	11.54	Chr. 6	4403742	C	C	T	C	T	Accurate
Marker3477	LG06	14.34	Chr. 6	4388805	C	C	T	C	T	Accurate
Marker3486	LG06	14.34	Chr. 6	4407747	A	A	G	A	G	Accurate
Marker3488	LG06	14.34	Chr. 6	4624107	A	A	G	A	G	Accurate
Marker3491	LG06	14.34	Chr. 6	4685846	A	A	T	A	T	Accurate
Marker3492	LG06	14.34	Chr. 6	4691559	T	T	C	T	Y	Uncertain
Marker3471	LG06	14.88	Chr. 6	4370506	A	A	G	A	G	Accurate
Marker3474	LG06	14.88	Chr. 6	4380190	G	G	C	G	C	Accurate
Marker3485	LG06	14.88	Chr. 6	4406434	C	C	A	C	A	Accurate

Marker3489	LG06	14.88	Chr. 6	4642545	C	C	G	C	G	Accurate
Marker3497	LG06	15.96	Chr. 6	4778435	T	T	C	T	C	Accurate
Marker3490	LG06	17.03	Chr. 6	4643176	T	T	G	T	G	Accurate
Marker3493	LG06	17.03	Chr. 6	4694583	T	T	C	T	C	Accurate
Marker3495	LG06	17.03	Chr. 6	4718797	G	G	A	G	A	Accurate
Marker3501	LG06	17.57	Chr. 6	4944512	T	A	T	A	T	Accurate
Marker3502	LG06	18.11	Chr. 6	4954453	T	C	T	C	T	Accurate
Marker3509	LG06	18.11	Chr. 6	4999857	G	A	G	A	G	Accurate
Marker3511	LG06	18.11	Chr. 6	5063940	A	A	C	A	C	Accurate
Marker3515	LG06	18.11	Chr. 6	5073723	A	A	G	A	G	Accurate
Marker3516	LG06	18.11	Chr. 6	5079498	C	C	T	C	T	Accurate
Marker3510	LG06	18.64	Chr. 6	5060929	G	G	A	G	A	Accurate
Marker3513	LG06	19.18	Chr. 6	5071384	C	T	C	T	C	Accurate
Marker3519	LG06	20.26	Chr. 6	5281878	C	C	T	C	T	Accurate
Marker3520	LG06	21.87	Chr. 6	5610330	G	G	T	G	T	Accurate
Marker3535	LG06	30.19	Chr. 6	6455671	C	A	C	A	C	Accurate
Marker3524	LG06	31.27	Chr. 6	6370917	C	T	C	T	C	Accurate
Marker3576	LG06	32.88	Chr. 6	7306891	C	A	C	A	C	Accurate
Marker3562	LG06	34.49	Chr. 6	6997359	A	G	A	G	A	Accurate
Marker3578	LG06	34.49	Chr. 6	7415806	T	C	T	C	T	Accurate
Marker3567	LG06	35.03	Chr. 6	7125942	T	C	T	C	T	Accurate
Marker3570	LG06	35.03	Chr. 6	7256697	A	A	G	A	G	Accurate
Marker3569	LG06	35.57	Chr. 6	7240009	G	G	T	G	T	Accurate
Marker3566	LG06	36.11	Chr. 6	7125907	C	A	C	A	C	Accurate
Marker3577	LG06	36.65	Chr. 6	7397809	C	C	T	C	T	Accurate
Marker3583	LG06	38.26	Chr. 6	7750959	G	A	G	A	G	Accurate
Marker3582	LG06	38.80	Chr. 6	7718113	T	C	T	C	T	Accurate
Marker3584	LG06	39.88	Chr. 6	7759027	C	G	C	G	C	Accurate
Marker3607	LG06	43.76	Chr. 6	9117551	T	C	T	C	T	Accurate
Marker3599	LG06	44.83	Chr. 6	8796635	G	A	G	A	G	Accurate
Marker3598	LG06	45.91	Chr. 6	8769345	C	T	C	T	C	Accurate
Marker3589	LG06	46.44	Chr. 6	8663344	G	A	G	A	G	Accurate
Marker3587	LG06	46.98	Chr. 6	8647534	G	A	G	A	G	Accurate
Marker3590	LG06	46.98	Chr. 6	8663492	A	G	A	G	A	Accurate
Marker3603	LG06	48.06	Chr. 6	9019637	C	C	T	C	T	Accurate
Marker3612	LG06	48.06	Chr. 6	9147897	T	T	A	T	A	Accurate
Marker3624	LG06	48.59	Chr. 6	9440804	G	G	A	G	A	Accurate
Marker3625	LG06	49.13	Chr. 6	9440816	T	T	C	T	C	Accurate
Marker3605	LG06	49.67	Chr. 6	9049534	G	G	A	G	A	Accurate
Marker3616	LG06	49.67	Chr. 6	9288847	C	T	C	T	C	Accurate
Marker3619	LG06	49.67	Chr. 6	9308762	T	C	T	C	T	Accurate
Marker3626	LG06	49.67	Chr. 6	9446244	G	G	T	G	T	Accurate
Marker3634	LG06	49.67	Chr. 6	9549578	T	T	C	T	C	Accurate
Marker3638	LG06	49.67	Chr. 6	9578132	T	C	T	C	T	Accurate
Marker3640	LG06	49.67	Chr. 6	9594455	C	T	C	T	C	Accurate
Marker3621	LG06	50.75	Chr. 6	9416050	T	T	C	T	C	Accurate
Marker3623	LG06	50.75	Chr. 6	9436699	C	C	T	C	T	Accurate

Marker3610	LG06	51.82	Chr. 6	9145125	A	A	G	A	G	Accurate
Marker3627	LG06	51.82	Chr. 6	9458056	T	T	C	T	C	Accurate
Marker3628	LG06	51.82	Chr. 6	9460389	G	G	A	G	A	Accurate
Marker3665	LG06	52.90	Chr. 6	10040191	G	A	G	A	G	Accurate
Marker3646	LG06	53.43	Chr. 6	9823648	G	T	G	T	G	Accurate
Marker3655	LG06	53.43	Chr. 6	9865482	A	G	A	G	A	Accurate
Marker3660	LG06	53.43	Chr. 6	9953398	A	G	A	G	A	Accurate
Marker3666	LG06	53.43	Chr. 6	10040197	G	A	G	A	G	Accurate
Marker3671	LG06	53.97	Chr. 6	10269287	T	A	T	A	T	Accurate
Marker3670	LG06	54.51	Chr. 6	10255482	C	T	C	T	C	Accurate
Marker3674	LG06	55.58	Chr. 6	10415832	C	C	T	C	T	Accurate
Marker3678	LG06	55.58	Chr. 6	10543462	A	G	A	G	A	Accurate
Marker3673	LG06	56.12	Chr. 6	10411781	G	G	C	G	C	Accurate
Marker3689	LG06	56.66	Chr. 6	10632593	C	T	C	T	C	Accurate
Marker3699	LG06	56.66	Chr. 6	10693463	T	C	T	C	T	Accurate
Marker3700	LG06	56.66	Chr. 6	10704385	A	G	A	G	A	Accurate
Marker3679	LG06	57.20	Chr. 6	10558204	A	T	A	T	A	Accurate
Marker3743	LG06	58.27	Chr. 6	11635493	A	T	A	T	A	Accurate
Marker3686	LG06	58.81	Chr. 6	10617066	A	G	A	G	A	Accurate
Marker3697	LG06	58.81	Chr. 6	10688826	G	A	G	A	G	Accurate
Marker3698	LG06	58.81	Chr. 6	10691023	A	G	A	G	A	Accurate
Marker3701	LG06	58.81	Chr. 6	10850515	A	A	G	A	G	Accurate
Marker3704	LG06	58.81	Chr. 6	10866567	G	G	A	G	A	Accurate
Marker3705	LG06	58.81	Chr. 6	11096445	G	A	G	A	G	Accurate
Marker3707	LG06	58.81	Chr. 6	11171051	T	G	T	G	T	Accurate
Marker3708	LG06	58.81	Chr. 6	11182899	T	G	T	G	T	Accurate
Marker3715	LG06	58.81	Chr. 6	11394675	C	T	C	T	C	Accurate
Marker3729	LG06	58.81	Chr. 6	11486497	C	A	C	A	C	Accurate
Marker3734	LG06	58.81	Chr. 6	11519467	T	C	T	C	T	Accurate
Marker3735	LG06	58.81	Chr. 6	11534899	C	T	C	T	C	Accurate
Marker3744	LG06	58.81	Chr. 6	11635630	C	G	C	G	C	Accurate
Marker3745	LG06	58.81	Chr. 6	11645101	C	T	C	T	C	Accurate
Marker3746	LG06	58.81	Chr. 6	11685881	T	G	T	G	T	Accurate
Marker3749	LG06	58.81	Chr. 6	11735553	G	T	G	T	G	Accurate
Marker3730	LG06	59.89	Chr. 6	11510373	G	A	G	A	G	Accurate
Marker3754	LG06	60.96	Chr. 6	11966862	C	A	C	A	C	Accurate
Marker3765	LG06	62.57	Chr. 6	12098946	A	A	T	A	T	Accurate
Marker3767	LG06	63.65	Chr. 6	12175219	C	C	T	C	T	Accurate
Marker3768	LG06	63.65	Chr. 6	12238860	T	T	C	T	C	Accurate
Marker3756	LG06	64.19	Chr. 6	12047729	C	C	T	C	T	Accurate
Marker3772	LG06	64.19	Chr. 6	12267517	G	G	C	G	C	Accurate
Marker3780	LG06	64.19	Chr. 6	12655902	A	T	A	T	A	Accurate
Marker3782	LG06	64.19	Chr. 6	12656050	G	T	G	T	G	Accurate
Marker3794	LG06	65.26	Chr. 6	12882331	G	A	G	A	G	Accurate
Marker3797	LG06	65.26	Chr. 6	12992711	T	C	T	C	T	Accurate
Marker3821	LG06	66.34	Chr. 6	16036223	T	T	G	T	G	Accurate
Marker3834	LG06	66.88	Chr. 6	16750985	A	A	G	A	G	Accurate

Marker3856	LG06	69.03	Chr. 6	17977041	C	C	T	C	T	Accurate
Marker3878	LG06	71.41	Chr. 6	18605021	T	T	C	T	C	Accurate
Marker3886	LG06	75.29	Chr. 6	19836104	G	G	A	G	A	Accurate
Marker3895	LG06	81.34	Chr. 6	20547422	T	T	C	N	C	Uncertain
Marker3905	LG06	85.45	Chr. 6	21200328	G	G	A	G	A	Accurate
Marker3917	LG06	85.99	Chr. 6	21478404	G	A	G	A	G	Accurate
Marker3912	LG06	87.06	Chr. 6	21457949	T	G	T	G	T	Accurate
Marker3922	LG06	87.06	Chr. 6	21623348	A	G	A	G	A	Accurate
Marker3923	LG06	87.06	Chr. 6	21625414	C	G	C	G	C	Accurate
Marker3945	LG06	87.60	Chr. 6	21927289	A	A	C	A	C	Accurate
Marker3910	LG06	88.14	Chr. 6	21385394	G	T	G	T	G	Accurate
Marker3918	LG06	88.67	Chr. 6	21482341	G	C	G	C	G	Accurate
Marker3926	LG06	88.67	Chr. 6	21648569	T	C	T	C	T	Accurate
Marker3928	LG06	88.67	Chr. 6	21664781	A	C	A	C	A	Accurate
Marker3938	LG06	88.67	Chr. 6	21819134	T	A	T	A	T	Accurate
Marker3947	LG06	88.67	Chr. 6	21949152	T	T	C	T	C	Accurate
Marker3919	LG06	89.21	Chr. 6	21483322	G	A	G	A	G	Accurate
Marker3927	LG06	89.21	Chr. 6	21660933	A	G	A	G	A	Accurate
Marker3933	LG06	89.21	Chr. 6	21749665	G	A	G	A	G	Accurate
Marker3936	LG06	89.75	Chr. 6	21800049	T	C	T	C	T	Accurate
Marker3934	LG06	91.36	Chr. 6	21750508	T	G	T	G	T	Accurate
Marker3949	LG06	92.98	Chr. 6	22062984	A	A	T	A	T	Accurate
Marker3950	LG06	94.05	Chr. 6	22164653	G	G	T	G	T	Accurate
Marker3954	LG06	94.05	Chr. 6	22206377	G	A	G	A	G	Accurate
Marker3966	LG06	96.20	Chr. 6	22529979	T	T	A	T	A	Accurate
Marker3975	LG06	98.90	Chr. 6	22835712	G	G	A	G	A	Accurate
Marker3979	LG06	99.44	Chr. 6	22945425	G	A	G	A	G	Accurate
Marker3989	LG06	99.44	Chr. 6	23341266	T	T	A	T	A	Accurate
Marker3984	LG06	99.97	Chr. 6	23134292	C	C	T	C	T	Accurate
Marker3988	LG06	100.51	Chr. 6	23339976	T	T	C	T	C	Accurate
Marker3992	LG06	101.59	Chr. 6	23543916	A	A	G	A	G	Accurate
Marker3995	LG06	103.74	Chr. 6	23614879	A	G	A	G	A	Accurate
Marker3997	LG06	105.89	Chr. 6	23793574	C	T	C	T	C	Accurate
Marker4004	LG06	110.31	Chr. 6	24048666	A	T	A	T	A	Accurate
Marker4007	LG06	110.31	Chr. 6	24053255	T	T	C	T	C	Accurate
Marker4013	LG06	111.93	Chr. 6	24285103	A	T	A	T	A	Accurate
Marker4012	LG06	112.47	Chr. 6	24251869	T	T	C	T	C	Accurate
Marker4016	LG06	113.01	Chr. 6	24320013	T	C	T	C	T	Accurate
Marker4015	LG06	113.55	Chr. 6	24287079	A	C	A	C	A	Accurate
Marker4017	LG06	114.31	Chr. 6	24321518	A	G	A	G	A	Accurate
Marker4021	LG06	115.38	Chr. 6	24404311	T	A	T	A	T	Accurate
Marker4023	LG06	117.53	Chr. 6	24440571	A	T	A	T	A	Accurate
Marker4036	LG06	123.15	Chr. 6	25192855	C	T	C	T	C	Accurate
Marker4279	LG06	124.76	Chr. 6	26424889	A	A	G	A	G	Accurate
Marker4282	LG06	125.30	Chr. 6	26425161	C	C	G	C	G	Accurate
Marker4280	LG06	126.91	Chr. 6	26425150	A	A	G	A	G	Accurate
Marker4428	LG06	126.91	Chr. 6	26663386	T	T	C	T	C	Accurate

Marker4281	LG06	127.45	Chr. 6	26425151	T	T	C	T	C	Accurate
Marker4098	LG06	127.99	Chr. 6	25915150	A	A	G	A	G	Accurate
Marker4432	LG06	127.99	Chr. 6	26695012	C	C	G	C	G	Accurate
Marker4050	LG06	129.60	Chr. 6	25826879	A	A	G	A	G	Accurate
Marker4134	LG06	129.60	Chr. 6	26008142	T	T	C	T	C	Accurate
Marker4228	LG06	129.60	Chr. 6	26241364	T	T	A	T	A	Accurate
Marker4240	LG06	129.60	Chr. 6	26266250	C	C	T	C	T	Accurate
Marker4275	LG06	129.60	Chr. 6	26385218	T	T	A	T	A	Accurate
Marker4116	LG06	130.14	Chr. 6	25953919	A	A	C	A	C	Accurate
Marker4140	LG06	130.14	Chr. 6	26012748	C	C	T	C	T	Accurate
Marker4175	LG06	130.14	Chr. 6	26082872	A	A	G	A	G	Accurate
Marker4452	LG06	130.14	Chr. 6	26758572	G	G	T	G	T	Accurate
Marker4461	LG06	130.14	Chr. 6	26848630	C	C	T	C	T	Accurate
Marker4136	LG06	130.90	Chr. 6	26008647	C	C	G	C	G	Accurate
Marker4155	LG06	130.90	Chr. 6	26044789	T	T	G	T	G	Accurate
Marker4159	LG06	130.90	Chr. 6	26060621	T	T	C	T	C	Accurate
Marker4188	LG06	131.44	Chr. 6	26123784	G	G	A	G	A	Accurate
Marker4400	LG06	131.98	Chr. 6	26602832	T	T	G	T	G	Accurate
Marker4399	LG06	132.52	Chr. 6	26602811	T	T	G	T	G	Accurate
Marker4359	LG06	134.13	Chr. 6	26565800	A	A	G	A	G	Accurate
Marker4103	LG06	135.74	Chr. 6	25931234	C	C	G	C	G	Accurate
Marker4129	LG06	136.28	Chr. 6	26000733	T	T	A	T	A	Accurate
Marker4241	LG06	136.82	Chr. 6	26271035	A	A	G	A	G	Accurate
Marker4085	LG06	137.89	Chr. 6	25899822	A	A	G	A	G	Accurate
Marker4067	LG06	138.43	Chr. 6	25859568	G	G	A	G	A	Accurate
Marker4069	LG06	138.43	Chr. 6	25873272	T	T	C	T	C	Accurate
Marker4084	LG06	138.43	Chr. 6	25886570	A	A	G	A	G	Accurate
Marker4089	LG06	138.43	Chr. 6	25900568	C	C	T	C	T	Accurate
Marker4179	LG06	138.43	Chr. 6	26094253	T	T	C	T	C	Accurate
Marker4206	LG06	138.43	Chr. 6	26207307	T	T	A	T	A	Accurate
Marker4447	LG06	138.43	Chr. 6	26737008	A	A	G	A	G	Accurate
Marker4449	LG06	138.43	Chr. 6	26750056	A	A	T	A	T	Accurate
Marker4458	LG06	138.43	Chr. 6	26837739	T	T	C	T	C	Accurate
Marker4047	LG06	138.97	Chr. 6	25767771	A	A	G	A	G	Accurate
Marker4071	LG06	138.97	Chr. 6	25874739	T	T	A	T	A	Accurate
Marker4124	LG06	138.97	Chr. 6	25994192	G	G	A	G	A	Accurate
Marker4178	LG06	138.97	Chr. 6	26093950	G	G	A	G	A	Accurate
Marker4192	LG06	138.97	Chr. 6	26150269	C	C	T	C	T	Accurate
Marker4273	LG06	138.97	Chr. 6	26381100	A	A	T	A	T	Accurate
Marker4288	LG06	138.97	Chr. 6	26426402	T	T	C	T	C	Accurate
Marker4305	LG06	138.97	Chr. 6	26479460	C	C	T	C	T	Accurate
Marker4306	LG06	138.97	Chr. 6	26487151	T	T	C	T	C	Accurate
Marker4344	LG06	138.97	Chr. 6	26537081	G	G	A	G	A	Accurate
Marker4394	LG06	138.97	Chr. 6	26598091	A	A	G	A	G	Accurate
Marker4424	LG06	138.97	Chr. 6	26658605	T	T	A	T	A	Accurate
Marker4431	LG06	138.97	Chr. 6	26689612	C	C	T	C	T	Accurate
Marker4448	LG06	138.97	Chr. 6	26744942	T	T	C	T	C	Accurate

Marker4255	LG06	139.51	Chr. 6	26313818	T	T	C	T	C	Accurate
Marker4278	LG06	139.51	Chr. 6	26418882	T	T	A	T	A	Accurate
Marker4257	LG06	140.04	Chr. 6	26327521	G	G	A	G	A	Accurate
Marker4336	LG06	140.04	Chr. 6	26520640	G	G	A	G	A	Accurate
Marker4350	LG06	140.04	Chr. 6	26555178	A	A	C	A	C	Accurate
Marker4171	LG06	140.58	Chr. 6	26074073	T	T	G	T	G	Accurate
Marker4058	LG06	141.12	Chr. 6	25841104	T	T	C	T	C	Accurate
Marker4216	LG06	141.12	Chr. 6	26229346	C	C	G	C	G	Accurate
Marker4191	LG06	142.30	Chr. 6	26140459	A	A	G	A	G	Accurate
Marker4238	LG06	142.30	Chr. 6	26261607	A	A	G	A	G	Accurate
Marker4057	LG06	142.84	Chr. 6	25839159	G	G	T	G	T	Accurate
Marker4102	LG06	144.14	Chr. 6	25927852	A	A	G	A	G	Accurate
Marker4202	LG06	144.14	Chr. 6	26199595	C	C	A	C	A	Accurate
Marker4222	LG06	144.14	Chr. 6	26231511	C	C	A	C	A	Accurate
Marker4289	LG06	144.14	Chr. 6	26436809	C	C	G	C	G	Accurate
Marker4427	LG06	144.14	Chr. 6	26659611	T	T	C	T	C	Accurate
Marker4346	LG06	144.67	Chr. 6	26553231	A	A	G	A	G	Accurate
Marker4349	LG06	145.21	Chr. 6	26553440	C	C	T	C	T	Accurate
Marker4044	LG06	145.75	Chr. 6	25733658	T	T	G	T	G	Accurate
Marker4194	LG06	145.75	Chr. 6	26192091	G	G	A	G	A	Accurate
Marker4274	LG06	145.75	Chr. 6	26381267	A	A	T	A	T	Accurate
Marker4430	LG06	145.75	Chr. 6	26689607	A	A	G	A	G	Accurate
Marker4287	LG06	146.83	Chr. 6	26425831	G	G	T	G	T	Accurate
Marker4164	LG06	147.36	Chr. 6	26064770	T	T	A	T	A	Accurate
Marker4239	LG06	147.36	Chr. 6	26265964	G	G	C	G	C	Accurate
Marker4322	LG06	147.90	Chr. 6	26512715	T	T	C	T	C	Accurate
Marker4075	LG06	148.44	Chr. 6	25876940	T	T	A	T	A	Accurate
Marker4415	LG06	148.44	Chr. 6	26618024	G	G	A	G	A	Accurate
Marker4425	LG06	148.44	Chr. 6	26658613	G	G	A	G	A	Accurate
Marker4429	LG06	148.44	Chr. 6	26678877	C	C	T	C	T	Accurate
Marker4076	LG06	148.98	Chr. 6	25876977	A	A	C	A	C	Accurate
Marker4078	LG06	149.51	Chr. 6	25877209	G	G	A	G	A	Accurate
Marker4181	LG06	149.51	Chr. 6	26108496	G	G	A	G	A	Accurate
Marker4180	LG06	150.05	Chr. 6	26108491	C	C	A	C	A	Accurate
Marker4157	LG06	151.89	Chr. 6	26051330	G	G	A	G	A	Accurate
Marker4269	LG06	151.89	Chr. 6	26369508	C	C	T	C	T	Accurate
Marker4060	LG06	152.43	Chr. 6	25848040	A	A	T	A	T	Accurate
Marker4242	LG06	152.43	Chr. 6	26272349	G	G	C	G	C	Accurate
Marker4268	LG06	152.43	Chr. 6	26369269	T	T	A	T	A	Accurate
Marker4318	LG06	152.43	Chr. 6	26495438	A	A	G	A	G	Accurate
Marker4326	LG06	152.43	Chr. 6	26517150	G	G	A	G	A	Accurate
Marker4327	LG06	152.98	Chr. 6	26517338	T	T	C	T	C	Accurate
Marker4419	LG06	152.98	Chr. 6	26621362	G	G	A	G	A	Accurate
Marker4422	LG06	152.98	Chr. 6	26655237	A	A	G	A	G	Accurate
Marker4393	LG06	154.05	Chr. 6	26598069	T	T	A	T	A	Accurate
Marker4520	LG07	0.00	Chr. 7	2304793	T	C	T	C	T	Accurate
Marker4530	LG07	0.00	Chr. 7	2527722	G	A	G	A	G	Accurate

Marker4525	LG07	0.54	Chr. 7	2331615	C	G	C	G	C	Accurate
Marker4524	LG07	1.08	Chr. 7	2331447	A	G	A	G	A	Accurate
Marker4512	LG07	1.61	Chr. 7	1969255	C	T	C	T	C	Accurate
Marker4498	LG07	2.15	Chr. 7	1568551	C	T	C	T	C	Accurate
Marker4517	LG07	2.15	Chr. 7	2131914	A	G	A	G	A	Accurate
Marker4518	LG07	2.15	Chr. 7	2136170	G	A	G	A	G	Accurate
Marker4519	LG07	2.15	Chr. 7	2148773	G	A	G	A	G	Accurate
Marker4504	LG07	2.69	Chr. 7	1790529	T	C	T	C	T	Accurate
Marker4514	LG07	2.69	Chr. 7	1984895	C	T	C	T	C	Accurate
Marker4497	LG07	3.76	Chr. 7	1524600	G	T	G	T	G	Accurate
Marker4501	LG07	3.76	Chr. 7	1770290	T	G	T	G	T	Accurate
Marker4499	LG07	7.22	Chr. 7	1762645	A	G	A	G	A	Accurate
Marker4470	LG07	9.91	Chr. 7	261221	C	T	C	T	C	Accurate
Marker4467	LG07	13.25	Chr. 7	218453	G	C	G	C	G	Accurate
Marker4462	LG07	17.23	Chr. 7	16270	C	T	C	T	C	Accurate
Marker4493	LG07	21.33	Chr. 7	1385355	T	A	T	A	T	Accurate
Marker4495	LG07	22.95	Chr. 7	1386960	C	T	C	T	C	Accurate
Marker4494	LG07	23.48	Chr. 7	1386691	C	T	C	T	C	Accurate
Marker4560	LG07	28.56	Chr. 7	3718830	T	T	C	T	C	Accurate
Marker4549	LG07	31.89	Chr. 7	3381259	G	G	T	N	T	Uncertain
Marker4542	LG07	32.97	Chr. 7	3313460	C	C	T	C	T	Accurate
Marker4548	LG07	32.97	Chr. 7	3347134	T	T	A	T	A	Accurate
Marker4539	LG07	34.04	Chr. 7	3271298	G	G	A	G	A	Accurate
Marker4540	LG07	34.58	Chr. 7	3298607	A	A	T	A	T	Accurate
Marker4543	LG07	34.58	Chr. 7	3323001	C	C	T	C	T	Accurate
Marker4566	LG07	38.57	Chr. 7	4129496	A	A	C	A	C	Accurate
Marker4575	LG07	40.83	Chr. 7	4364797	C	C	A	C	A	Accurate
Marker4573	LG07	42.98	Chr. 7	4361613	G	G	C	G	C	Accurate
Marker4581	LG07	44.06	Chr. 7	4880311	G	G	A	G	A	Accurate
Marker4582	LG07	44.59	Chr. 7	4889465	C	C	A	C	A	Accurate
Marker4580	LG07	45.13	Chr. 7	4700232	G	A	G	A	G	Accurate
Marker4572	LG07	45.67	Chr. 7	4306631	A	A	G	A	G	Accurate
Marker4576	LG07	45.67	Chr. 7	4553726	C	C	A	C	A	Accurate
Marker4571	LG07	47.28	Chr. 7	4280973	A	T	A	T	A	Accurate
Marker4585	LG07	48.36	Chr. 7	5352976	G	G	A	G	A	Accurate
Marker4590	LG07	51.16	Chr. 7	5481934	G	G	A	G	A	Accurate
Marker4589	LG07	51.69	Chr. 7	5430321	G	G	A	G	A	Accurate
Marker4587	LG07	52.23	Chr. 7	5396592	C	C	A	C	A	Accurate
Marker4593	LG07	53.85	Chr. 7	5535277	G	G	A	G	A	Accurate
Marker4602	LG07	55.46	Chr. 7	5973670	G	C	G	C	G	Accurate
Marker7770	LG07	61.95	Chr. 0	7692904	T	C	T	C	T	Accurate
Marker4631	LG07	62.48	Chr. 7	8847317	A	A	G	A	G	Accurate
Marker4604	LG07	63.02	Chr. 7	7800415	G	A	G	A	G	Accurate
Marker4615	LG07	63.56	Chr. 7	8104105	A	A	G	A	G	Accurate
Marker4624	LG07	63.56	Chr. 7	8697985	A	T	A	T	A	Accurate
Marker4626	LG07	63.56	Chr. 7	8718481	G	A	G	A	G	Accurate
Marker4617	LG07	64.10	Chr. 7	8154176	G	G	A	G	A	Accurate

Marker4638	LG07	64.10	Chr. 7	8915759	G	A	G	A	G	Accurate
Marker4606	LG07	64.63	Chr. 7	7856566	C	T	C	T	C	Accurate
Marker4611	LG07	64.63	Chr. 7	7926445	C	T	C	T	C	Accurate
Marker4625	LG07	66.79	Chr. 7	8708214	G	T	G	T	G	Accurate
Marker4685	LG07	75.54	Chr. 7	20597178	T	C	T	C	T	Accurate
Marker4679	LG07	79.52	Chr. 7	20363519	T	C	T	C	T	Accurate
Marker4669	LG07	81.14	Chr. 7	20025669	T	C	T	C	T	Accurate
Marker4688	LG07	82.86	Chr. 7	20717580	G	A	G	A	G	Accurate
Marker4678	LG07	84.47	Chr. 7	20111573	A	C	A	C	A	Accurate
Marker4674	LG07	85.55	Chr. 7	20081528	T	A	T	A	T	Accurate
Marker4662	LG07	86.09	Chr. 7	19993519	G	A	G	A	G	Accurate
Marker4675	LG07	86.09	Chr. 7	20081529	C	A	C	A	C	Accurate
Marker4672	LG07	87.16	Chr. 7	20038269	T	C	T	C	T	Accurate
Marker4661	LG07	88.24	Chr. 7	19976124	A	G	A	G	A	Accurate
Marker4677	LG07	88.24	Chr. 7	20107629	A	G	A	G	A	Accurate
Marker4664	LG07	88.77	Chr. 7	19999905	G	T	G	T	G	Accurate
Marker4684	LG07	90.50	Chr. 7	20580156	C	T	C	T	C	Accurate
Marker4671	LG07	95.13	Chr. 7	20034303	G	A	G	A	G	Accurate
Marker4879	LG07	105.18	Chr. 7	27185133	G	C	G	C	G	Accurate
Marker4880	LG07	106.26	Chr. 7	27192472	G	C	G	C	G	Accurate
Marker4870	LG07	111.67	Chr. 7	26841054	C	C	G	C	G	Accurate
Marker4871	LG07	112.21	Chr. 7	26841318	A	A	C	A	C	Accurate
Marker4853	LG07	116.20	Chr. 7	26178674	C	T	C	T	C	Accurate
Marker4831	LG07	119.42	Chr. 7	25886045	A	T	A	T	A	Accurate
Marker4833	LG07	120.50	Chr. 7	25895263	C	T	C	T	C	Accurate
Marker4843	LG07	121.04	Chr. 7	26125823	T	C	A	T	C	Inconsistent
Marker4783	LG07	128.61	Chr. 7	24290186	A	G	A	G	A	Accurate
Marker4782	LG07	129.69	Chr. 7	24259542	G	A	G	A	G	Accurate
Marker4792	LG07	131.30	Chr. 7	24470528	C	T	C	T	C	Accurate
Marker4795	LG07	131.30	Chr. 7	24497160	T	C	T	C	T	Accurate
Marker4798	LG07	131.30	Chr. 7	24564290	T	C	T	C	T	Accurate
Marker4785	LG07	131.84	Chr. 7	24426298	A	G	A	G	A	Accurate
Marker4786	LG07	131.84	Chr. 7	24430744	G	T	G	T	G	Accurate
Marker4784	LG07	132.91	Chr. 7	24315038	A	T	A	T	A	Accurate
Marker4780	LG07	133.45	Chr. 7	24255244	C	T	C	T	C	Accurate
Marker4781	LG07	133.45	Chr. 7	24256365	C	A	C	A	C	Accurate
Marker4779	LG07	135.06	Chr. 7	24252498	T	A	T	A	T	Accurate
Marker5011	LG07	139.59	Chr. 7	31276722	C	T	C	T	C	Accurate
Marker5008	LG07	140.13	Chr. 7	31175906	G	A	G	A	G	Accurate
Marker4995	LG07	142.93	Chr. 7	30770799	G	A	G	A	G	Accurate
Marker4996	LG07	144.54	Chr. 7	30780334	G	A	G	A	G	Accurate
Marker4994	LG07	146.16	Chr. 7	30769590	C	T	C	T	C	Accurate
Marker4993	LG07	148.31	Chr. 7	30749146	C	T	C	T	C	Accurate
Marker4965	LG07	155.57	Chr. 7	29909370	G	C	G	C	G	Accurate
Marker4964	LG07	157.30	Chr. 7	29909317	T	C	T	C	T	Accurate
Marker4961	LG07	159.56	Chr. 7	29798791	A	A	T	A	T	Accurate
Marker4959	LG07	160.63	Chr. 7	29782357	C	C	T	C	T	Accurate

Marker4956	LG07	162.35	Chr. 7	29540302	C	T	C	T	C	Accurate
Marker4935	LG07	162.89	Chr. 7	28758597	T	T	G	T	G	Accurate
Marker4957	LG07	162.89	Chr. 7	29540315	T	C	T	C	T	Accurate
Marker4966	LG07	163.43	Chr. 7	29967385	G	T	G	T	G	Accurate
Marker4952	LG07	163.96	Chr. 7	29359362	C	T	C	T	C	Accurate
Marker4946	LG07	164.50	Chr. 7	29265940	C	T	C	T	C	Accurate
Marker4954	LG07	164.50	Chr. 7	29506308	G	A	G	A	G	Accurate
Marker4948	LG07	165.04	Chr. 7	29269728	C	T	C	T	C	Accurate
Marker4934	LG07	167.19	Chr. 7	28748715	T	T	C	T	C	Accurate
Marker4936	LG07	167.19	Chr. 7	28760121	G	G	T	G	T	Accurate
Marker4932	LG07	167.73	Chr. 7	28679249	T	T	C	T	C	Accurate
Marker5038	LG08	0.00	Chr. 8	3231883	T	C	T	C	T	Accurate
Marker5013	LG08	0.54	Chr. 8	31016	T	A	T	A	T	Accurate
Marker5015	LG08	0.54	Chr. 8	81168	G	A	G	A	G	Accurate
Marker5016	LG08	0.54	Chr. 8	92489	G	A	G	A	G	Accurate
Marker5030	LG08	0.54	Chr. 8	3171078	T	C	T	C	T	Accurate
Marker5040	LG08	0.54	Chr. 8	3241866	A	G	A	G	A	Accurate
Marker5047	LG08	0.54	Chr. 8	3334300	C	T	C	T	C	Accurate
Marker5296	LG08	0.54	Chr. 8	12664980	T	C	T	C	T	Accurate
Marker5297	LG08	0.54	Chr. 8	12675687	A	C	A	C	A	Accurate
Marker5301	LG08	0.54	Chr. 8	12754505	G	T	G	T	G	Accurate
Marker5303	LG08	0.54	Chr. 8	12779596	G	A	G	A	G	Accurate
Marker5304	LG08	0.54	Chr. 8	12791127	C	T	C	T	C	Accurate
Marker7881	LG08	0.54	Chr. 0	16451506	G	A	G	A	G	Accurate
Marker5268	LG08	1.08	Chr. 8	11788458	G	T	G	T	G	Accurate
Marker5266	LG08	2.15	Chr. 8	11639076	C	T	C	T	C	Accurate
Marker5245	LG08	2.69	Chr. 8	10664189	A	G	A	G	A	Accurate
Marker5247	LG08	2.69	Chr. 8	10685392	A	G	A	G	A	Accurate
Marker5253	LG08	3.23	Chr. 8	10778633	T	C	T	C	T	Accurate
Marker5256	LG08	3.23	Chr. 8	10792438	A	C	A	C	A	Accurate
Marker5258	LG08	3.23	Chr. 8	10868504	G	A	G	A	G	Accurate
Marker5257	LG08	4.30	Chr. 8	10795752	A	G	A	G	A	Accurate
Marker5189	LG08	7.64	Chr. 8	9410640	G	G	A	G	A	Accurate
Marker5196	LG08	8.94	Chr. 8	9615111	T	T	A	T	A	Accurate
Marker5200	LG08	8.94	Chr. 8	9644309	T	T	C	T	C	Accurate
Marker5181	LG08	9.47	Chr. 8	9124619	A	G	A	G	A	Accurate
Marker5182	LG08	9.47	Chr. 8	9136759	T	C	T	C	T	Accurate
Marker5187	LG08	9.47	Chr. 8	9272339	G	A	G	A	G	Accurate
Marker5195	LG08	9.47	Chr. 8	9459012	T	T	C	T	C	Accurate
Marker5198	LG08	10.55	Chr. 8	9628781	C	C	T	N	T	Uncertain
Marker5158	LG08	10.55	Chr. 8	8939978	G	G	T	G	T	Accurate
Marker5169	LG08	10.55	Chr. 8	9044262	T	C	T	C	T	Accurate
Marker5174	LG08	10.55	Chr. 8	9081945	T	C	T	C	T	Accurate
Marker5176	LG08	10.55	Chr. 8	9093750	A	G	A	G	A	Accurate
Marker7929	LG08	10.55	Chr. 0	18589779	A	A	G	A	G	Accurate
Marker5149	LG08	11.09	Chr. 8	8882901	G	G	A	G	A	Accurate
Marker5154	LG08	11.09	Chr. 8	8909802	T	T	C	T	C	Accurate

Marker5161	LG08	11.09	Chr. 8	8955376	C	C	A	C	A	Accurate
Marker5165	LG08	11.09	Chr. 8	9019408	C	T	C	T	C	Accurate
Marker5168	LG08	11.09	Chr. 8	9036985	T	C	T	C	T	Accurate
Marker5180	LG08	11.09	Chr. 8	9122292	C	T	C	T	C	Accurate
Marker5201	LG08	11.09	Chr. 8	9657837	T	T	A	N	A	Uncertain
Marker5208	LG08	11.09	Chr. 8	9747893	C	C	T	C	T	Accurate
Marker5215	LG08	11.09	Chr. 8	9807940	C	C	T	C	T	Accurate
Marker5219	LG08	11.09	Chr. 8	9891974	G	G	A	G	A	Accurate
Marker5220	LG08	11.09	Chr. 8	9945094	C	A	C	A	C	Accurate
Marker5205	LG08	11.63	Chr. 8	9729203	G	G	A	G	A	Accurate
Marker5152	LG08	12.16	Chr. 8	8902426	T	T	C	T	C	Accurate
Marker5150	LG08	12.70	Chr. 8	8902123	T	T	C	T	C	Accurate
Marker5213	LG08	12.70	Chr. 8	9761249	A	A	T	A	T	Accurate
Marker5191	LG08	13.24	Chr. 8	9446042	G	G	A	G	A	Accurate
Marker5207	LG08	13.24	Chr. 8	9731847	G	G	T	G	T	Accurate
Marker5199	LG08	13.78	Chr. 8	9629676	T	T	C	T	C	Accurate
Marker5162	LG08	14.32	Chr. 8	8973437	C	C	T	C	T	Accurate
Marker5173	LG08	14.32	Chr. 8	9079750	A	G	A	G	A	Accurate
Marker5178	LG08	14.32	Chr. 8	9109488	G	A	G	A	G	Accurate
Marker5183	LG08	14.32	Chr. 8	9140133	C	T	C	T	C	Accurate
Marker5204	LG08	14.86	Chr. 8	9701845	G	G	A	G	A	Accurate
Marker5146	LG08	15.39	Chr. 8	8863252	G	G	A	G	A	Accurate
Marker5282	LG08	15.93	Chr. 8	12135585	A	A	G	A	G	Accurate
Marker5291	LG08	15.93	Chr. 8	12367581	G	G	A	G	A	Accurate
Marker5292	LG08	15.93	Chr. 8	12410662	G	G	A	G	A	Accurate
Marker5347	LG08	15.93	Chr. 8	13315756	G	G	A	G	A	Accurate
Marker5353	LG08	15.93	Chr. 8	13387046	C	C	G	C	G	Accurate
Marker7939	LG08	16.47	Chr. 0	18612308	T	T	G	T	G	Accurate
Marker7940	LG08	16.47	Chr. 0	18650827	G	G	A	G	A	Accurate
Marker7935	LG08	17.01	Chr. 0	18605738	T	T	G	T	G	Accurate
Marker5289	LG08	17.55	Chr. 8	12316333	T	T	C	T	C	Accurate
Marker5225	LG08	18.08	Chr. 8	10032933	A	G	A	R	A	Uncertain
Marker5277	LG08	18.62	Chr. 8	11992828	A	A	C	A	C	Accurate
Marker5279	LG08	18.62	Chr. 8	12102641	G	G	A	G	A	Accurate
Marker5354	LG08	18.62	Chr. 8	13387781	C	C	T	C	T	Accurate
Marker5321	LG08	19.16	Chr. 8	13039844	A	A	G	A	G	Accurate
Marker5226	LG08	19.70	Chr. 8	10039446	G	G	A	G	A	Accurate
Marker5238	LG08	19.70	Chr. 8	10114738	T	T	C	T	C	Accurate
Marker5239	LG08	19.70	Chr. 8	10170158	A	A	C	A	C	Accurate
Marker5243	LG08	19.70	Chr. 8	10362631	T	T	C	T	C	Accurate
Marker5244	LG08	19.70	Chr. 8	10396537	G	G	C	G	C	Accurate
Marker5286	LG08	19.70	Chr. 8	12216568	G	G	A	G	R	Uncertain
Marker5311	LG08	19.70	Chr. 8	12913555	G	G	A	G	A	Accurate
Marker5316	LG08	19.70	Chr. 8	12988431	A	A	C	A	C	Accurate
Marker5324	LG08	19.70	Chr. 8	13057889	C	C	A	C	A	Accurate
Marker5326	LG08	19.70	Chr. 8	13081428	T	T	A	T	A	Accurate
Marker5329	LG08	19.70	Chr. 8	13093737	T	T	G	N	G	Uncertain

Marker5337	LG08	19.70	Chr. 8	13157973	A	A	G	A	G	Accurate
Marker5342	LG08	19.70	Chr. 8	13302659	C	C	T	C	T	Accurate
Marker5352	LG08	19.70	Chr. 8	13383207	C	C	G	N	G	Uncertain
Marker7804	LG08	19.70	Chr. 0	11698208	T	T	C	T	C	Accurate
Marker7838	LG08	19.70	Chr. 0	14279219	A	A	G	A	G	Accurate
Marker7990	LG08	19.70	Chr. 0	21274237	T	T	C	T	C	Accurate
Marker7995	LG08	19.70	Chr. 0	21386454	C	C	T	C	T	Accurate
Marker8000	LG08	19.70	Chr. 0	21424318	T	T	A	T	A	Accurate
Marker8013	LG08	19.70	Chr. 0	22521064	A	A	C	A	C	Accurate
Marker8014	LG08	19.70	Chr. 0	22554291	A	A	G	A	G	Accurate
Marker5325	LG08	20.23	Chr. 8	13076555	T	T	C	T	C	Accurate
Marker5346	LG08	20.23	Chr. 8	13315296	A	A	G	A	G	Accurate
Marker5317	LG08	20.77	Chr. 8	12990414	T	T	A	T	A	Accurate
Marker5345	LG08	20.77	Chr. 8	13315290	C	C	T	C	T	Accurate
Marker5233	LG08	20.77	Chr. 8	10075101	A	A	G	A	G	Accurate
Marker5234	LG08	21.31	Chr. 8	10075338	A	A	G	A	G	Accurate
Marker5242	LG08	21.85	Chr. 8	10293194	T	T	C	T	C	Accurate
Marker5063	LG08	21.85	Chr. 8	5089748	G	T	G	T	G	Accurate
Marker5344	LG08	22.92	Chr. 8	13315185	C	C	T	C	T	Accurate
Marker7943	LG08	24.00	Chr. 0	18705397	T	T	C	T	C	Accurate
Marker5343	LG08	25.07	Chr. 8	13314979	A	A	G	A	G	Accurate
Marker7945	LG08	25.07	Chr. 0	18708697	G	G	A	G	A	Accurate
Marker7944	LG08	25.61	Chr. 0	18708354	A	A	C	A	C	Accurate
Marker5332	LG08	25.61	Chr. 8	13143586	G	G	A	G	A	Accurate
Marker5285	LG08	26.15	Chr. 8	12190035	T	T	C	T	C	Accurate
Marker5323	LG08	27.22	Chr. 8	13041108	A	A	G	A	R	Uncertain
Marker5315	LG08	27.77	Chr. 8	12988164	C	C	G	C	G	Accurate
Marker5322	LG08	27.77	Chr. 8	13041094	C	C	T	C	Y	Uncertain
Marker5136	LG08	28.85	Chr. 8	6911988	C	C	T	C	T	Accurate
Marker5137	LG08	29.92	Chr. 8	6923969	A	A	C	A	C	Accurate
Marker5138	LG08	30.46	Chr. 8	6928637	A	A	G	A	G	Accurate
Marker5051	LG08	31.54	Chr. 8	4439190	A	G	A	G	A	Accurate
Marker5085	LG08	32.07	Chr. 8	5238570	C	A	C	A	C	Accurate
Marker5083	LG08	32.61	Chr. 8	5238322	T	C	T	C	T	Accurate
Marker5084	LG08	33.15	Chr. 8	5238326	A	C	A	C	A	Accurate
Marker5071	LG08	33.69	Chr. 8	5153601	A	A	C	A	C	Accurate
Marker5094	LG08	33.69	Chr. 8	5263343	G	A	G	A	G	Accurate
Marker5096	LG08	33.69	Chr. 8	5283542	G	G	C	G	C	Accurate
Marker5060	LG08	34.23	Chr. 8	5070999	C	T	C	T	C	Accurate
Marker5062	LG08	34.23	Chr. 8	5085616	C	T	C	T	C	Accurate
Marker5067	LG08	34.23	Chr. 8	5095349	T	C	T	C	T	Accurate
Marker5101	LG08	34.23	Chr. 8	5538136	C	A	C	A	C	Accurate
Marker5091	LG08	34.76	Chr. 8	5240559	C	A	C	A	C	Accurate
Marker5089	LG08	35.30	Chr. 8	5240356	A	G	A	G	A	Accurate
Marker5095	LG08	35.30	Chr. 8	5279469	G	G	C	G	C	Accurate
Marker5104	LG08	36.91	Chr. 8	5681312	A	A	T	A	T	Accurate
Marker5102	LG08	37.45	Chr. 8	5590288	C	T	C	T	C	Accurate

Marker5103	LG08	37.45	Chr. 8	5670117	T	T	C	T	C	Accurate
Marker5113	LG08	37.45	Chr. 8	5711889	T	T	C	T	C	Accurate
Marker5118	LG08	37.45	Chr. 8	5845709	A	A	G	A	G	Accurate
Marker5116	LG08	38.53	Chr. 8	5721724	G	G	A	G	A	Accurate
Marker5123	LG08	38.53	Chr. 8	5861407	A	A	G	A	G	Accurate
Marker5128	LG08	38.53	Chr. 8	5915785	T	T	C	N	C	Uncertain
Marker5129	LG08	38.53	Chr. 8	5945247	A	A	G	A	G	Accurate
Marker5126	LG08	40.14	Chr. 8	5895257	T	T	G	T	G	Accurate
Marker5069	LG08	40.68	Chr. 8	5101797	T	C	T	C	T	Accurate
Marker5078	LG08	40.68	Chr. 8	5233963	A	G	A	G	A	Accurate
Marker5087	LG08	41.75	Chr. 8	5240300	T	C	T	C	T	Accurate
Marker5066	LG08	42.83	Chr. 8	5095024	G	A	G	A	G	Accurate
Marker5122	LG08	43.37	Chr. 8	5858739	T	T	C	T	C	Accurate
Marker5121	LG08	43.90	Chr. 8	5858730	A	A	G	A	G	Accurate
Marker5364	LG08	44.98	Chr. 8	14360034	A	G	A	G	A	Accurate
Marker5135	LG08	46.06	Chr. 8	6554121	G	T	G	T	G	Accurate
Marker5134	LG08	46.59	Chr. 8	6554021	T	C	T	C	T	Accurate
Marker5355	LG08	46.59	Chr. 8	14245246	A	G	A	G	A	Accurate
Marker5356	LG08	46.59	Chr. 8	14256104	G	A	G	A	G	Accurate
Marker5360	LG08	46.59	Chr. 8	14317852	T	C	T	C	T	Accurate
Marker5363	LG08	46.59	Chr. 8	14350716	A	C	A	C	A	Accurate
Marker5361	LG08	47.13	Chr. 8	14317865	G	C	G	C	G	Accurate
Marker5357	LG08	47.67	Chr. 8	14290329	G	C	G	C	G	Accurate
Marker5367	LG08	47.67	Chr. 8	14392141	C	T	C	T	C	Accurate
Marker5373	LG08	47.67	Chr. 8	14425198	T	G	T	G	T	Accurate
Marker5377	LG08	47.67	Chr. 8	14488528	C	T	C	T	C	Accurate
Marker5374	LG08	48.21	Chr. 8	14458871	G	A	G	A	G	Accurate
Marker5376	LG08	48.21	Chr. 8	14488473	T	C	T	C	T	Accurate
Marker5365	LG08	49.82	Chr. 8	14382393	G	C	G	C	G	Accurate
Marker5375	LG08	52.96	Chr. 8	14465237	T	A	T	A	T	Accurate
Marker5392	LG08	59.69	Chr. 8	15727393	T	C	T	C	T	Accurate
Marker5389	LG08	61.15	Chr. 8	15705087	T	G	T	G	T	Accurate
Marker5396	LG08	62.76	Chr. 8	15769284	C	T	C	T	C	Accurate
Marker5385	LG08	64.91	Chr. 8	15551820	C	T	C	T	C	Accurate
Marker5401	LG08	68.90	Chr. 8	16501923	C	A	C	A	C	Accurate
Marker5397	LG08	72.35	Chr. 8	16410159	T	C	T	C	T	Accurate
Marker5400	LG08	72.35	Chr. 8	16487102	A	T	A	T	A	Accurate
Marker5405	LG08	74.07	Chr. 8	16593804	T	A	T	A	T	Accurate
Marker5406	LG08	75.14	Chr. 8	16594039	A	C	A	C	A	Accurate
Marker5409	LG08	76.22	Chr. 8	16619614	T	G	T	G	T	Accurate
Marker5403	LG08	76.75	Chr. 8	16574385	C	A	C	A	C	Accurate
Marker5404	LG08	77.29	Chr. 8	16578670	C	A	C	A	C	Accurate
Marker5410	LG08	77.29	Chr. 8	16619804	A	T	A	T	A	Accurate
Marker5418	LG08	81.93	Chr. 8	17012927	G	T	G	T	G	Accurate
Marker5419	LG08	81.93	Chr. 8	17016390	C	T	C	T	C	Accurate
Marker5421	LG08	82.46	Chr. 8	17141236	G	A	G	A	G	Accurate
Marker5423	LG08	82.46	Chr. 8	17225855	T	A	T	A	T	Accurate

Marker5425	LG08	82.46	Chr. 8	17246454	C	A	C	A	C	Accurate
Marker5429	LG08	84.08	Chr. 8	17348793	C	T	C	T	C	Accurate
Marker5431	LG08	84.61	Chr. 8	17450984	C	C	T	C	T	Accurate
Marker5442	LG08	85.15	Chr. 8	17482656	G	A	G	A	G	Accurate
Marker5445	LG08	85.15	Chr. 8	17507730	C	C	A	C	A	Accurate
Marker5447	LG08	85.15	Chr. 8	17600099	C	C	G	C	G	Accurate
Marker5443	LG08	85.69	Chr. 8	17482876	A	G	A	G	A	Accurate
Marker5446	LG08	85.69	Chr. 8	17525166	A	A	G	A	G	Accurate
Marker5450	LG08	85.69	Chr. 8	17780046	C	G	C	G	C	Accurate
Marker5449	LG08	87.30	Chr. 8	17779526	G	T	G	T	G	Accurate
Marker5436	LG08	87.84	Chr. 8	17468394	C	C	T	C	T	Accurate
Marker5440	LG08	88.38	Chr. 8	17481986	T	G	T	G	T	Accurate
Marker5441	LG08	88.92	Chr. 8	17482227	T	C	T	C	T	Accurate
Marker5432	LG08	90.53	Chr. 8	17457959	G	G	T	G	T	Accurate
Marker5451	LG08	95.06	Chr. 8	17953552	G	A	G	A	G	Accurate
Marker5456	LG08	104.46	Chr. 8	19388141	C	A	C	A	C	Accurate
Marker5467	LG08	107.92	Chr. 8	19827414	C	A	C	A	C	Accurate
Marker5458	LG08	110.07	Chr. 8	19736391	T	A	T	A	T	Accurate
Marker5459	LG08	112.22	Chr. 8	19743634	A	C	A	C	A	Accurate
Marker5465	LG08	114.38	Chr. 8	19804348	G	G	T	G	T	Accurate
Marker5463	LG08	115.99	Chr. 8	19767778	T	G	T	G	T	Accurate
Marker5461	LG08	117.07	Chr. 8	19761381	A	G	A	G	A	Accurate
Marker5470	LG08	118.68	Chr. 8	19873332	A	G	A	G	A	Accurate
Marker5473	LG08	119.75	Chr. 8	19900283	T	T	C	T	C	Accurate
Marker5483	LG08	120.83	Chr. 8	20279115	A	A	T	A	T	Accurate
Marker5474	LG08	121.37	Chr. 8	20045863	T	C	T	C	T	Accurate
Marker5481	LG08	122.44	Chr. 8	20273279	G	G	A	G	A	Accurate
Marker5476	LG08	122.98	Chr. 8	20154005	G	A	G	A	G	Accurate
Marker5485	LG08	124.59	Chr. 8	20378448	G	A	G	A	G	Accurate
Marker5489	LG08	126.75	Chr. 8	20970111	T	A	T	A	T	Accurate
Marker5493	LG08	128.90	Chr. 8	21248620	A	G	A	G	A	Accurate
Marker5499	LG08	132.46	Chr. 8	21555932	G	A	G	A	G	Accurate
Marker5503	LG08	135.80	Chr. 8	22041759	A	G	A	G	A	Accurate
Marker5502	LG08	137.52	Chr. 8	22013160	G	G	T	G	T	Accurate
Marker5505	LG08	138.59	Chr. 8	22126178	G	A	G	A	G	Accurate
Marker5504	LG08	140.75	Chr. 8	22059311	G	T	G	T	G	Accurate
Marker5513	LG08	140.75	Chr. 8	22423355	G	G	T	G	T	Accurate
Marker5507	LG08	141.28	Chr. 8	22182332	C	C	A	C	A	Accurate
Marker5506	LG08	142.36	Chr. 8	22174213	T	T	A	T	A	Accurate
Marker5515	LG08	143.44	Chr. 8	22507509	C	A	C	A	C	Accurate
Marker5512	LG08	145.81	Chr. 8	22358337	C	C	A	C	A	Accurate
Marker5525	LG08	146.34	Chr. 8	22636836	G	A	G	A	G	Accurate
Marker5526	LG08	147.42	Chr. 8	22653255	C	C	T	C	T	Accurate
Marker5527	LG08	148.50	Chr. 8	22778092	T	T	G	T	G	Accurate
Marker5528	LG08	151.83	Chr. 8	22780967	G	G	T	G	T	Accurate
Marker5541	LG08	154.09	Chr. 8	23186198	A	A	G	A	G	Accurate
Marker5530	LG08	154.63	Chr. 8	22852240	G	G	A	G	A	Accurate

Marker5536	LG08	154.63	Chr. 8	23162164	C	C	A	C	A	Accurate
Marker5540	LG08	155.70	Chr. 8	23186145	G	G	A	G	A	Accurate
Marker5546	LG08	155.70	Chr. 8	23212747	A	A	G	A	G	Accurate
Marker5551	LG08	155.70	Chr. 8	23242850	G	G	T	G	T	Accurate
Marker5552	LG08	156.24	Chr. 8	23257453	C	C	A	C	A	Accurate
Marker5554	LG08	156.24	Chr. 8	23309232	A	A	G	A	G	Accurate
Marker5543	LG08	156.78	Chr. 8	23203911	G	G	A	G	A	Accurate
Marker5548	LG08	156.78	Chr. 8	23215465	T	T	G	T	G	Accurate
Marker5531	LG08	157.85	Chr. 8	22878365	A	A	G	A	G	Accurate
Marker5539	LG08	157.85	Chr. 8	23184517	A	A	G	A	G	Accurate
Marker5561	LG08	157.85	Chr. 8	23588727	C	T	C	T	C	Accurate
Marker5556	LG08	158.39	Chr. 8	23392871	T	T	A	T	A	Accurate
Marker5557	LG08	160.65	Chr. 8	23426144	T	T	A	T	A	Accurate
Marker5560	LG08	161.73	Chr. 8	23533916	C	C	T	C	T	Accurate
Marker5558	LG08	162.26	Chr. 8	23428393	G	G	T	G	T	Accurate
Marker5559	LG08	163.34	Chr. 8	23479520	A	A	T	A	T	Accurate
Marker5568	LG08	165.60	Chr. 8	23910867	C	C	T	C	T	Accurate
Marker5566	LG08	166.67	Chr. 8	23795877	T	T	C	T	C	Accurate
Marker5567	LG08	167.21	Chr. 8	23803123	A	A	G	A	G	Accurate
Marker5572	LG08	167.75	Chr. 8	23970914	C	C	T	C	T	Accurate
Marker5574	LG08	168.29	Chr. 8	23985254	G	G	A	G	A	Accurate
Marker5576	LG08	168.29	Chr. 8	24027640	C	C	A	C	A	Accurate
Marker5575	LG08	168.83	Chr. 8	24009272	T	T	A	T	A	Accurate
Marker5583	LG08	168.83	Chr. 8	24291653	T	T	C	T	C	Accurate
Marker5578	LG08	169.58	Chr. 8	24149356	A	A	G	N	G	Uncertain
Marker5582	LG08	170.12	Chr. 8	24176100	C	C	T	C	T	Accurate
Marker5592	LG08	173.15	Chr. 8	24379105	C	C	A	C	A	Accurate
Marker5585	LG08	173.69	Chr. 8	24362244	T	T	C	T	C	Accurate
Marker5586	LG08	174.77	Chr. 8	24365150	A	A	G	A	G	Accurate
Marker5587	LG08	175.84	Chr. 8	24365531	G	G	T	G	T	Accurate
Marker5600	LG08	176.92	Chr. 8	24475808	G	G	A	G	A	Accurate
Marker5607	LG08	176.92	Chr. 8	24680690	T	T	G	T	G	Accurate
Marker5611	LG08	176.92	Chr. 8	24775028	G	G	A	G	A	Accurate
Marker5612	LG08	177.99	Chr. 8	24798421	A	A	C	A	C	Accurate
Marker5614	LG08	179.07	Chr. 8	24820724	T	T	C	T	C	Accurate
Marker5609	LG08	180.14	Chr. 8	24765603	G	A	G	A	G	Accurate
Marker5624	LG08	181.76	Chr. 8	25326667	A	A	T	A	T	Accurate
Marker5615	LG08	182.83	Chr. 8	25158367	T	T	C	T	C	Accurate
Marker5618	LG08	183.37	Chr. 8	25276320	A	A	G	A	G	Accurate
Marker5626	LG08	183.37	Chr. 8	25362295	T	T	C	T	C	Accurate
Marker5627	LG08	184.45	Chr. 8	25442777	T	T	C	T	C	Accurate
Marker5628	LG08	185.52	Chr. 8	25446606	A	A	C	A	C	Accurate
Marker5617	LG08	187.14	Chr. 8	25251741	A	A	T	A	T	Accurate
Marker5631	LG08	188.21	Chr. 8	25509926	A	A	G	A	G	Accurate
Marker5633	LG08	189.82	Chr. 8	25641616	G	A	G	A	G	Accurate
Marker5634	LG08	189.82	Chr. 8	25665170	G	A	G	A	G	Accurate
Marker5635	LG08	190.36	Chr. 8	25669454	A	G	A	G	A	Accurate

Marker5640	LG08	191.44	Chr. 8	25909981	A	A	G	A	G	Accurate
Marker5639	LG08	191.97	Chr. 8	25764408	C	C	T	C	T	Accurate
Marker7952	LG08	191.97	Chr. 0	18972115	A	T	A	T	A	Accurate
Marker7955	LG08	191.97	Chr. 0	18993968	A	T	A	T	A	Accurate
Marker7984	LG09	0.00	Chr. 0	21158302	C	C	T	C	T	Accurate
Marker7985	LG09	2.69	Chr. 0	21158502	G	G	A	G	A	Accurate
Marker5645	LG09	5.84	Chr. 9	9755	A	A	G	A	G	Accurate
Marker5653	LG09	5.84	Chr. 9	304478	A	A	T	A	T	Accurate
Marker5652	LG09	6.38	Chr. 9	304448	T	T	C	T	C	Accurate
Marker5654	LG09	7.48	Chr. 9	307837	A	A	G	A	G	Accurate
Marker7983	LG09	8.02	Chr. 0	21076845	T	T	C	T	C	Accurate
Marker7986	LG09	8.02	Chr. 0	21158956	C	C	G	C	G	Accurate
Marker5670	LG09	8.02	Chr. 9	682929	G	A	G	A	G	Accurate
Marker5657	LG09	8.56	Chr. 9	310397	A	A	C	A	C	Accurate
Marker5668	LG09	9.85	Chr. 9	645128	G	A	G	A	G	Accurate
Marker5661	LG09	10.39	Chr. 9	611353	C	C	A	C	A	Accurate
Marker5669	LG09	10.39	Chr. 9	645143	T	T	C	T	C	Accurate
Marker5677	LG09	10.39	Chr. 9	785639	A	G	A	G	A	Accurate
Marker5655	LG09	11.46	Chr. 9	307882	C	C	T	C	T	Accurate
Marker5664	LG09	11.46	Chr. 9	628627	T	T	C	T	C	Accurate
Marker5675	LG09	11.46	Chr. 9	776366	G	T	G	T	G	Accurate
Marker5676	LG09	12.54	Chr. 9	785630	C	A	C	A	C	Accurate
Marker5663	LG09	13.62	Chr. 9	611653	G	G	T	G	T	Accurate
Marker5662	LG09	14.15	Chr. 9	611643	G	G	C	G	C	Accurate
Marker5693	LG09	14.69	Chr. 9	1194218	G	T	G	T	G	Accurate
Marker5726	LG09	14.69	Chr. 9	1952596	C	G	C	G	C	Accurate
Marker5699	LG09	16.30	Chr. 9	1283854	A	C	A	C	A	Accurate
Marker5698	LG09	16.84	Chr. 9	1253822	G	T	G	T	G	Accurate
Marker5717	LG09	16.84	Chr. 9	1812524	G	C	G	C	G	Accurate
Marker5697	LG09	18.99	Chr. 9	1248748	A	G	A	G	A	Accurate
Marker5748	LG09	22.14	Chr. 9	2383616	A	A	G	A	G	Accurate
Marker5721	LG09	23.75	Chr. 9	1877239	A	G	A	G	A	Accurate
Marker5758	LG09	25.37	Chr. 9	2941733	A	G	A	G	A	Accurate
Marker5776	LG09	26.98	Chr. 9	3501665	A	A	G	A	G	Accurate
Marker5771	LG09	27.52	Chr. 9	3445415	A	A	G	A	G	Accurate
Marker5768	LG09	28.06	Chr. 9	3422489	A	A	G	A	G	Accurate
Marker5760	LG09	28.59	Chr. 9	3311418	A	G	A	G	A	Accurate
Marker5787	LG09	28.59	Chr. 9	3748109	A	A	T	A	T	Accurate
Marker5782	LG09	29.67	Chr. 9	3620214	G	G	A	G	A	Accurate
Marker5793	LG09	29.67	Chr. 9	3844692	T	T	C	T	C	Accurate
Marker5773	LG09	31.28	Chr. 9	3487504	T	T	A	T	A	Accurate
Marker5778	LG09	31.28	Chr. 9	3587972	G	G	A	G	A	Accurate
Marker5781	LG09	31.28	Chr. 9	3606957	A	A	G	A	G	Accurate
Marker5796	LG09	31.28	Chr. 9	3918894	A	A	G	A	G	Accurate
Marker5794	LG09	32.36	Chr. 9	3847372	G	G	T	G	T	Accurate
Marker5789	LG09	32.89	Chr. 9	3763020	T	T	C	T	C	Accurate
Marker5805	LG09	33.43	Chr. 9	4013157	C	C	T	C	T	Accurate

Marker5800	LG09	35.05	Chr. 9	3932883	G	G	A	G	A	Accurate
Marker5788	LG09	35.58	Chr. 9	3748987	G	G	A	G	A	Accurate
Marker5785	LG09	36.66	Chr. 9	3726388	T	T	A	T	A	Accurate
Marker5795	LG09	36.66	Chr. 9	3918381	C	C	T	C	T	Accurate
Marker5813	LG09	36.66	Chr. 9	4302482	A	A	G	A	G	Accurate
Marker5807	LG09	38.81	Chr. 9	4191767	T	T	C	T	C	Accurate
Marker5814	LG09	40.96	Chr. 9	4326425	T	T	C	T	C	Accurate
Marker5815	LG09	42.26	Chr. 9	4532750	A	A	G	A	G	Accurate
Marker5827	LG09	43.87	Chr. 9	4994481	C	T	C	T	C	Accurate
Marker5824	LG09	45.83	Chr. 9	4975894	A	C	A	C	A	Accurate
Marker5820	LG09	47.13	Chr. 9	4952770	A	G	A	G	A	Accurate
Marker5830	LG09	48.43	Chr. 9	5588580	G	G	A	G	A	Accurate
Marker5828	LG09	50.26	Chr. 9	5062749	A	G	A	G	A	Accurate
Marker5867	LG09	54.49	Chr. 9	6897002	G	G	T	G	T	Accurate
Marker5866	LG09	56.10	Chr. 9	6871645	G	A	G	A	G	Accurate
Marker5874	LG09	58.25	Chr. 9	6971076	C	C	A	C	A	Accurate
Marker5878	LG09	59.33	Chr. 9	7020156	T	C	T	C	T	Accurate
Marker5888	LG09	59.87	Chr. 9	7509564	C	T	C	T	C	Accurate
Marker5885	LG09	60.40	Chr. 9	7159659	C	C	T	C	T	Accurate
Marker5895	LG09	60.40	Chr. 9	7598288	A	A	T	A	T	Accurate
Marker5906	LG09	60.40	Chr. 9	7787484	C	C	T	C	T	Accurate
Marker5887	LG09	60.94	Chr. 9	7509529	G	C	G	C	G	Accurate
Marker5903	LG09	60.94	Chr. 9	7748845	A	A	G	A	G	Accurate
Marker5909	LG09	60.94	Chr. 9	7822495	A	A	T	A	T	Accurate
Marker5913	LG09	60.94	Chr. 9	7854769	G	G	A	G	A	Accurate
Marker5908	LG09	61.48	Chr. 9	7804914	A	A	G	A	G	Accurate
Marker5898	LG09	62.02	Chr. 9	7723449	T	T	C	T	C	Accurate
Marker5902	LG09	62.02	Chr. 9	7738312	G	G	T	G	T	Accurate
Marker5910	LG09	62.02	Chr. 9	7826476	G	G	T	G	T	Accurate
Marker5894	LG09	63.09	Chr. 9	7535424	C	T	C	T	C	Accurate
Marker5918	LG09	63.09	Chr. 9	7972677	C	C	T	C	T	Accurate
Marker5930	LG09	66.00	Chr. 9	9297485	C	T	C	T	C	Accurate
Marker5937	LG09	66.76	Chr. 9	9379947	G	C	G	C	G	Accurate
Marker5934	LG09	68.60	Chr. 9	9339329	G	A	G	A	G	Accurate
Marker5932	LG09	70.75	Chr. 9	9333932	G	A	G	A	G	Accurate
Marker5936	LG09	70.75	Chr. 9	9339374	C	T	C	T	C	Accurate
Marker5938	LG09	70.75	Chr. 9	9382238	G	A	G	A	G	Accurate
Marker5935	LG09	72.42	Chr. 9	9339353	A	C	A	C	A	Accurate
Marker5954	LG09	73.49	Chr. 9	10079028	G	A	G	A	G	Accurate
Marker5933	LG09	74.57	Chr. 9	9334251	A	G	A	G	N	Uncertain
Marker5943	LG09	75.10	Chr. 9	9816211	G	A	G	A	G	Accurate
Marker5969	LG09	76.72	Chr. 9	10641644	C	T	C	T	C	Accurate
Marker5962	LG09	77.79	Chr. 9	10583933	G	C	G	C	G	Accurate
Marker5950	LG09	78.33	Chr. 9	10031045	C	T	C	T	C	Accurate
Marker5960	LG09	78.87	Chr. 9	10555423	T	C	T	C	T	Accurate
Marker5986	LG09	79.94	Chr. 9	11212679	C	T	C	T	C	Accurate
Marker5963	LG09	80.48	Chr. 9	10594413	G	A	G	A	G	Accurate

Marker5978	LG09	80.48	Chr. 9	10860440	A	G	A	G	A	Accurate
Marker5980	LG09	80.48	Chr. 9	10911984	T	C	T	C	T	Accurate
Marker5979	LG09	81.02	Chr. 9	10868637	C	T	C	T	C	Accurate
Marker5981	LG09	82.10	Chr. 9	10917947	G	A	G	A	G	Accurate
Marker5968	LG09	82.63	Chr. 9	10630414	T	A	T	A	T	Accurate
Marker5967	LG09	83.71	Chr. 9	10630076	G	C	G	C	G	Accurate
Marker5999	LG09	83.71	Chr. 9	11607412	C	T	C	T	C	Accurate
Marker5964	LG09	85.32	Chr. 9	10611418	G	A	G	A	G	Accurate
Marker5975	LG09	85.32	Chr. 9	10818308	C	T	C	T	C	Accurate
Marker5990	LG09	85.86	Chr. 9	11228024	T	C	T	C	T	Accurate
Marker5991	LG09	85.86	Chr. 9	11544405	G	A	G	A	G	Accurate
Marker5998	LG09	85.86	Chr. 9	11607152	G	T	G	T	G	Accurate
Marker6004	LG09	85.86	Chr. 9	11685707	G	T	G	T	G	Accurate
Marker5989	LG09	86.40	Chr. 9	11228016	G	T	G	T	G	Accurate
Marker5996	LG09	87.47	Chr. 9	11584392	C	T	C	T	C	Accurate
Marker6032	LG09	87.47	Chr. 9	12340155	T	G	T	G	T	Accurate
Marker6021	LG09	88.01	Chr. 9	11890220	A	G	A	G	A	Accurate
Marker6035	LG09	88.01	Chr. 9	12389845	A	G	A	G	A	Accurate
Marker5995	LG09	88.55	Chr. 9	11584362	T	C	T	C	T	Accurate
Marker5997	LG09	88.55	Chr. 9	11586374	T	C	T	C	T	Accurate
Marker6000	LG09	88.55	Chr. 9	11607683	C	T	C	T	C	Accurate
Marker6025	LG09	88.55	Chr. 9	11964161	A	G	A	G	A	Accurate
Marker6011	LG09	89.09	Chr. 9	11827087	A	G	A	G	A	Accurate
Marker6030	LG09	89.09	Chr. 9	12006400	G	T	G	T	G	Accurate
Marker6033	LG09	89.09	Chr. 9	12347937	C	G	C	G	C	Accurate
Marker6034	LG09	89.09	Chr. 9	12377575	T	C	T	C	T	Accurate
Marker6037	LG09	89.09	Chr. 9	12404688	A	T	A	T	A	Accurate
Marker5992	LG09	89.62	Chr. 9	11545697	C	T	C	T	C	Accurate
Marker6012	LG09	89.62	Chr. 9	11827101	A	G	A	G	A	Accurate
Marker6029	LG09	89.62	Chr. 9	12006380	C	T	C	T	C	Accurate
Marker6015	LG09	90.16	Chr. 9	11842970	T	G	T	G	T	Accurate
Marker6044	LG09	90.16	Chr. 9	12693057	A	C	A	C	A	Accurate
Marker6002	LG09	90.70	Chr. 9	11647187	T	C	T	C	T	Accurate
Marker6036	LG09	91.77	Chr. 9	12401683	C	T	C	T	C	Accurate
Marker6041	LG09	92.31	Chr. 9	12669281	A	T	A	T	A	Accurate
Marker6048	LG09	92.31	Chr. 9	12737361	C	A	C	A	C	Accurate
Marker6053	LG09	92.31	Chr. 9	12931646	C	T	C	T	C	Accurate
Marker6040	LG09	92.85	Chr. 9	12669100	G	A	G	A	G	Accurate
Marker6045	LG09	92.85	Chr. 9	12699166	G	A	G	A	G	Accurate
Marker6047	LG09	92.85	Chr. 9	12727352	C	G	C	G	C	Accurate
Marker6049	LG09	92.85	Chr. 9	12737860	T	C	T	C	T	Accurate
Marker6006	LG09	93.92	Chr. 9	11724492	T	A	T	A	T	Accurate
Marker5984	LG09	94.46	Chr. 9	11201473	T	A	T	A	T	Accurate
Marker5983	LG09	96.08	Chr. 9	11201247	A	G	A	G	A	Accurate
Marker6050	LG09	96.61	Chr. 9	12748262	T	G	T	G	T	Accurate
Marker6062	LG09	96.61	Chr. 9	14022147	A	G	A	G	A	Accurate
Marker6005	LG09	97.15	Chr. 9	11723737	C	A	C	A	C	Accurate

Marker6031	LG09	97.15	Chr. 9	12019426	T	C	T	C	T	Accurate
Marker6051	LG09	97.15	Chr. 9	12748294	C	T	C	T	C	Accurate
Marker6061	LG09	97.69	Chr. 9	14012740	G	A	G	A	G	Accurate
Marker6067	LG09	98.76	Chr. 9	14295795	T	C	T	C	T	Accurate
Marker6070	LG09	98.76	Chr. 9	14342513	A	G	A	G	A	Accurate
Marker6076	LG09	98.76	Chr. 9	14500385	A	T	A	T	A	Accurate
Marker6071	LG09	99.30	Chr. 9	14355527	C	T	C	T	C	Accurate
Marker6073	LG09	99.30	Chr. 9	14373584	T	C	T	C	T	Accurate
Marker6072	LG09	99.84	Chr. 9	14371424	G	A	G	A	G	Accurate
Marker6068	LG09	100.91	Chr. 9	14330995	T	C	T	C	T	Accurate
Marker6069	LG09	101.45	Chr. 9	14339618	G	T	G	T	G	Accurate
Marker6098	LG09	103.07	Chr. 9	15653083	G	A	G	A	G	Accurate
Marker6077	LG09	103.60	Chr. 9	15135213	G	A	G	A	G	Accurate
Marker6149	LG09	103.60	Chr. 9	16345252	T	A	T	A	T	Accurate
Marker6080	LG09	104.14	Chr. 9	15349163	G	A	G	A	G	Accurate
Marker6078	LG09	104.68	Chr. 9	15294513	A	T	A	T	A	Accurate
Marker6083	LG09	104.68	Chr. 9	15392992	C	T	C	T	C	Accurate
Marker6084	LG09	104.68	Chr. 9	15441055	T	A	T	A	T	Accurate
Marker6090	LG09	104.68	Chr. 9	15498277	A	G	A	G	A	Accurate
Marker6091	LG09	104.68	Chr. 9	15534336	T	C	T	C	T	Accurate
Marker6093	LG09	104.68	Chr. 9	15605242	A	C	A	C	A	Accurate
Marker6110	LG09	104.68	Chr. 9	15667760	G	A	G	A	G	Accurate
Marker6111	LG09	104.68	Chr. 9	15729171	T	C	T	C	T	Accurate
Marker6117	LG09	104.68	Chr. 9	15793233	A	C	A	C	A	Accurate
Marker6121	LG09	104.68	Chr. 9	15844402	C	T	C	T	C	Accurate
Marker6124	LG09	104.68	Chr. 9	15904154	A	G	A	G	A	Accurate
Marker6132	LG09	104.68	Chr. 9	15982343	T	C	T	C	T	Accurate
Marker6138	LG09	104.68	Chr. 9	16080131	A	G	A	G	A	Accurate
Marker6141	LG09	104.68	Chr. 9	16153380	G	A	G	A	G	Accurate
Marker6150	LG09	104.68	Chr. 9	16345255	A	G	A	G	A	Accurate
Marker6173	LG09	104.68	Chr. 9	17056221	G	A	G	A	G	Accurate
Marker6232	LG09	104.68	Chr. 9	18139750	T	C	T	C	T	Accurate
Marker6112	LG09	105.22	Chr. 9	15737596	G	A	G	A	G	Accurate
Marker6122	LG09	105.22	Chr. 9	15844403	T	C	T	C	T	Accurate
Marker6092	LG09	105.75	Chr. 9	15597438	G	A	G	A	G	Accurate
Marker6096	LG09	105.75	Chr. 9	15642797	G	A	G	A	G	Accurate
Marker6109	LG09	105.75	Chr. 9	15667756	G	A	G	A	G	Accurate
Marker6130	LG09	105.75	Chr. 9	15960579	G	A	G	A	G	Accurate
Marker6097	LG09	106.83	Chr. 9	15644902	T	C	T	C	T	Accurate
Marker6205	LG09	107.37	Chr. 9	17322808	T	C	T	C	T	Accurate
Marker6243	LG09	107.90	Chr. 9	18569903	C	A	C	A	C	Accurate
Marker6192	LG09	108.44	Chr. 9	17201197	C	T	C	T	C	Accurate
Marker6222	LG09	108.44	Chr. 9	17467100	T	C	T	C	T	Accurate
Marker6188	LG09	109.52	Chr. 9	17175605	C	T	C	T	C	Accurate
Marker6230	LG09	109.52	Chr. 9	18139188	C	T	C	T	C	Accurate
Marker6181	LG09	110.06	Chr. 9	17111898	G	A	G	A	G	Accurate
Marker6186	LG09	110.06	Chr. 9	17172842	A	T	A	T	A	Accurate

Marker6214	LG09	110.06	Chr. 9	17354792	G	T	G	T	G	Accurate
Marker6226	LG09	110.06	Chr. 9	17504589	G	A	G	A	G	Accurate
Marker6229	LG09	110.06	Chr. 9	17602497	C	T	C	T	C	Accurate
Marker6234	LG09	110.06	Chr. 9	18164757	G	A	G	A	G	Accurate
Marker6236	LG09	110.06	Chr. 9	18192530	T	C	T	Y	T	Uncertain
Marker6244	LG09	110.06	Chr. 9	18929917	G	A	G	A	G	Accurate
Marker6219	LG09	110.59	Chr. 9	17458627	T	G	T	G	T	Accurate
Marker6223	LG09	110.59	Chr. 9	17482062	A	C	A	C	A	Accurate
Marker6155	LG09	111.13	Chr. 9	16849430	C	T	C	T	C	Accurate
Marker6162	LG09	111.13	Chr. 9	16867948	A	G	A	G	A	Accurate
Marker6165	LG09	111.13	Chr. 9	16936775	G	A	G	A	G	Accurate
Marker6177	LG09	111.13	Chr. 9	17070955	T	C	T	C	T	Accurate
Marker6185	LG09	111.13	Chr. 9	17172817	T	C	T	C	T	Accurate
Marker6202	LG09	111.13	Chr. 9	17300502	C	T	C	T	C	Accurate
Marker6204	LG09	111.13	Chr. 9	17320977	A	G	A	G	A	Accurate
Marker6225	LG09	111.13	Chr. 9	17496930	G	A	G	A	G	Accurate
Marker6449	LG09	111.13	Chr. 9	26393552	C	T	C	T	C	Accurate
Marker6450	LG09	111.13	Chr. 9	26416504	T	C	T	C	T	Accurate
Marker6451	LG09	111.13	Chr. 9	26491307	A	C	A	C	A	Accurate
Marker6461	LG09	111.13	Chr. 9	26719922	T	C	T	C	T	Accurate
Marker6217	LG09	112.21	Chr. 9	17413861	T	C	T	C	T	Accurate
Marker7682	LG09	112.74	Chr. 0	880150	G	T	G	T	G	Accurate
Marker6156	LG09	112.74	Chr. 9	16852361	G	A	G	A	G	Accurate
Marker6178	LG09	112.74	Chr. 9	17073540	A	T	A	T	A	Accurate
Marker6183	LG09	112.74	Chr. 9	17153572	C	A	C	A	C	Accurate
Marker6187	LG09	112.74	Chr. 9	17175556	T	A	T	A	T	Accurate
Marker6216	LG09	112.74	Chr. 9	17372175	G	A	G	A	G	Accurate
Marker6221	LG09	112.74	Chr. 9	17464242	C	T	C	T	C	Accurate
Marker6478	LG09	112.74	Chr. 9	27130928	G	T	G	T	G	Accurate
Marker6493	LG09	112.74	Chr. 9	27306903	T	C	T	C	T	Accurate
Marker6506	LG09	112.74	Chr. 9	27447181	A	C	A	C	A	Accurate
Marker6516	LG09	112.74	Chr. 9	27533754	A	G	A	G	A	Accurate
Marker6561	LG09	112.74	Chr. 9	27932081	G	T	G	T	G	Accurate
Marker6575	LG09	112.74	Chr. 9	28021732	T	A	T	A	T	Accurate
Marker6576	LG09	112.74	Chr. 9	28028782	A	G	A	G	A	Accurate
Marker6583	LG09	112.74	Chr. 9	28216227	T	C	T	C	T	Accurate
Marker6588	LG09	112.74	Chr. 9	28273610	A	G	A	G	A	Accurate
Marker6456	LG09	113.28	Chr. 9	26659349	C	A	C	A	C	Accurate
Marker6468	LG09	113.28	Chr. 9	27058512	A	A	G	A	G	Accurate
Marker6485	LG09	113.28	Chr. 9	27184601	A	C	A	C	A	Accurate
Marker6487	LG09	113.28	Chr. 9	27197741	C	T	C	T	C	Accurate
Marker6552	LG09	113.28	Chr. 9	27830791	T	A	T	A	T	Accurate
Marker6577	LG09	113.28	Chr. 9	28028973	G	A	G	A	N	Uncertain
Marker7752	LG09	113.28	Chr. 0	6136987	A	G	A	G	A	Accurate
Marker7679	LG09	113.82	Chr. 0	857811	C	T	C	T	C	Accurate
Marker7828	LG09	113.82	Chr. 0	13533717	G	T	G	T	G	Accurate
Marker6333	LG09	113.82	Chr. 9	22961967	G	G	A	G	A	Accurate

Marker6337	LG09	113.82	Chr. 9	23272849	G	A	G	A	G	Accurate
Marker6350	LG09	113.82	Chr. 9	23652214	T	A	T	A	T	Accurate
Marker6419	LG09	113.82	Chr. 9	25910399	C	C	T	C	T	Accurate
Marker6429	LG09	113.82	Chr. 9	26043702	C	C	G	C	G	Accurate
Marker6430	LG09	113.82	Chr. 9	26046715	G	G	C	G	C	Accurate
Marker6437	LG09	113.82	Chr. 9	26178567	G	G	A	G	A	Accurate
Marker6442	LG09	113.82	Chr. 9	26217619	C	C	T	C	T	Accurate
Marker6465	LG09	113.82	Chr. 9	27014760	C	T	C	T	C	Accurate
Marker6474	LG09	113.82	Chr. 9	27098024	G	G	A	G	A	Accurate
Marker6483	LG09	113.82	Chr. 9	27170261	T	C	T	C	T	Accurate
Marker6489	LG09	113.82	Chr. 9	27228810	C	T	C	T	C	Accurate
Marker6511	LG09	113.82	Chr. 9	27475134	T	A	T	A	T	Accurate
Marker6540	LG09	113.82	Chr. 9	27670290	T	C	T	C	T	Accurate
Marker6541	LG09	113.82	Chr. 9	27700961	C	T	C	T	C	Accurate
Marker6550	LG09	113.82	Chr. 9	27783343	G	A	G	A	G	Accurate
Marker6564	LG09	113.82	Chr. 9	27945622	T	C	T	C	T	Accurate
Marker6568	LG09	113.82	Chr. 9	27985123	A	G	A	G	A	Accurate
Marker6570	LG09	113.82	Chr. 9	27993854	T	A	T	A	T	Accurate
Marker6344	LG09	114.36	Chr. 9	23448878	T	T	C	T	C	Accurate
Marker6418	LG09	114.36	Chr. 9	25905807	C	C	A	C	A	Accurate
Marker6427	LG09	114.36	Chr. 9	26006695	C	C	T	C	T	Accurate
Marker6431	LG09	114.36	Chr. 9	26047393	A	A	C	A	C	Accurate
Marker6448	LG09	114.36	Chr. 9	26335958	A	T	A	T	A	Accurate
Marker6471	LG09	114.36	Chr. 9	27082473	T	T	G	T	G	Accurate
Marker6486	LG09	114.36	Chr. 9	27190310	T	C	T	C	T	Accurate
Marker6510	LG09	114.36	Chr. 9	27474877	G	A	G	A	G	Accurate
Marker6535	LG09	114.36	Chr. 9	27614932	C	A	C	A	C	Accurate
Marker6536	LG09	114.36	Chr. 9	27643479	G	A	G	A	G	Accurate
Marker6548	LG09	114.36	Chr. 9	27782233	A	G	A	G	A	Accurate
Marker6559	LG09	114.36	Chr. 9	27917573	A	G	A	G	A	Accurate
Marker7676	LG09	114.36	Chr. 0	834305	T	G	T	G	T	Accurate
Marker7678	LG09	114.36	Chr. 0	851368	G	A	G	A	G	Accurate
Marker7684	LG09	114.36	Chr. 0	880443	G	A	G	A	G	Accurate
Marker7760	LG09	114.36	Chr. 0	6583475	T	G	T	G	T	Accurate
Marker7758	LG09	115.43	Chr. 0	6567332	G	A	G	A	G	Accurate
Marker6346	LG09	115.43	Chr. 9	23460009	A	A	C	A	C	Accurate
Marker6359	LG09	115.43	Chr. 9	23734692	C	T	C	T	C	Accurate
Marker6417	LG09	115.43	Chr. 9	25875734	T	T	C	T	C	Accurate
Marker6439	LG09	115.43	Chr. 9	26186453	A	A	G	A	G	Accurate
Marker6514	LG09	115.43	Chr. 9	27518398	T	C	T	C	T	Accurate
Marker6555	LG09	115.43	Chr. 9	27868721	G	A	G	A	G	Accurate
Marker6569	LG09	115.43	Chr. 9	27986470	A	T	A	T	A	Accurate
Marker6415	LG09	116.51	Chr. 9	25875354	T	T	A	T	A	Accurate
Marker6424	LG09	116.51	Chr. 9	25941247	G	G	A	G	A	Accurate
Marker6201	LG09	117.05	Chr. 9	17298105	C	G	C	G	C	Accurate
Marker6215	LG09	117.05	Chr. 9	17371248	T	C	T	C	T	Accurate
Marker6368	LG09	117.05	Chr. 9	24026906	A	A	C	A	C	Accurate

Marker6416	LG09	117.05	Chr. 9	25875497	A	A	T	A	T	Accurate
Marker6432	LG09	117.05	Chr. 9	26050447	T	T	C	T	C	Accurate
Marker6433	LG09	117.05	Chr. 9	26151137	C	T	C	T	C	Accurate
Marker6440	LG09	117.05	Chr. 9	26194862	C	C	T	C	T	Accurate
Marker6444	LG09	117.05	Chr. 9	26296950	T	G	T	G	T	Accurate
Marker6466	LG09	117.05	Chr. 9	27022517	A	G	A	G	A	Accurate
Marker6467	LG09	117.05	Chr. 9	27042061	T	C	T	C	T	Accurate
Marker6509	LG09	117.05	Chr. 9	27461230	T	G	T	G	T	Accurate
Marker6529	LG09	117.05	Chr. 9	27573248	G	A	G	A	G	Accurate
Marker6532	LG09	117.05	Chr. 9	27598003	T	G	T	G	T	Accurate
Marker6551	LG09	117.05	Chr. 9	27808516	A	G	A	G	A	Accurate
Marker6556	LG09	117.05	Chr. 9	27887841	G	A	G	A	G	Accurate
Marker7675	LG09	117.05	Chr. 0	822854	G	A	G	A	G	Accurate
Marker7689	LG09	117.05	Chr. 0	987400	T	C	T	C	T	Accurate
Marker7687	LG09	118.12	Chr. 0	987147	A	G	A	G	A	Accurate
Marker7683	LG09	118.12	Chr. 0	880228	G	A	G	A	G	Accurate
Marker6490	LG09	118.12	Chr. 9	27279592	G	A	G	A	G	Accurate
Marker6348	LG09	119.20	Chr. 9	23515953	A	A	C	A	C	Accurate
Marker6351	LG09	119.73	Chr. 9	23705035	C	T	C	T	Y	Uncertain
Marker6370	LG09	120.81	Chr. 9	24031037	A	A	G	A	G	Accurate
Marker6386	LG09	120.81	Chr. 9	24334613	T	T	C	T	C	Accurate
Marker6365	LG09	121.35	Chr. 9	23883849	C	C	A	C	A	Accurate
Marker6366	LG09	121.35	Chr. 9	23899019	G	G	A	G	A	Accurate
Marker6369	LG09	121.88	Chr. 9	24030989	A	A	C	A	C	Accurate
Marker6387	LG09	121.88	Chr. 9	24360497	G	G	A	G	A	Accurate
Marker6354	LG09	122.42	Chr. 9	23721186	A	G	A	G	A	Accurate
Marker6364	LG09	122.42	Chr. 9	23862969	C	C	A	C	A	Accurate
Marker6371	LG09	122.42	Chr. 9	24151202	C	C	T	C	T	Accurate
Marker6377	LG09	122.42	Chr. 9	24235318	C	C	T	C	T	Accurate
Marker6379	LG09	122.42	Chr. 9	24261817	G	G	A	G	A	Accurate
Marker6385	LG09	122.42	Chr. 9	24329724	A	A	G	A	G	Accurate
Marker6389	LG09	122.42	Chr. 9	24376178	T	T	C	T	C	Accurate
Marker6391	LG09	122.42	Chr. 9	24666411	T	T	C	N	C	Uncertain
Marker6394	LG09	122.42	Chr. 9	24696583	G	G	T	G	T	Accurate
Marker6406	LG09	122.42	Chr. 9	24903178	C	T	C	Y	C	Uncertain
Marker6409	LG09	122.42	Chr. 9	24949098	C	C	T	C	T	Accurate
Marker6380	LG09	123.50	Chr. 9	24292054	A	A	G	A	G	Accurate
Marker6388	LG09	123.50	Chr. 9	24360501	G	G	T	G	T	Accurate
Marker6372	LG09	124.04	Chr. 9	24151217	C	C	T	C	T	Accurate
Marker6397	LG09	124.04	Chr. 9	24745769	C	C	T	C	T	Accurate
Marker6400	LG09	124.04	Chr. 9	24844803	T	T	C	T	C	Accurate
Marker6405	LG09	124.04	Chr. 9	24903156	T	C	T	C	T	Accurate
Marker6401	LG09	124.57	Chr. 9	24853405	C	G	C	G	C	Accurate
Marker6398	LG09	125.11	Chr. 9	24745830	C	C	T	C	T	Accurate
Marker6396	LG09	125.65	Chr. 9	24724442	T	T	C	T	C	Accurate
Marker6257	LG09	126.19	Chr. 9	19711327	A	G	A	G	A	Accurate
Marker6258	LG09	126.19	Chr. 9	19735714	G	A	G	A	G	Accurate

Marker6262	LG09	126.19	Chr. 9	19766407	C	A	C	A	C	Accurate
Marker6261	LG09	126.72	Chr. 9	19757736	G	A	G	A	G	Accurate
Marker6256	LG09	127.26	Chr. 9	19708986	A	G	A	G	A	Accurate
Marker6259	LG09	128.34	Chr. 9	19753050	A	G	A	G	A	Accurate
Marker6263	LG09	128.87	Chr. 9	19767655	G	A	G	A	G	Accurate
Marker6268	LG09	129.95	Chr. 9	20736951	C	T	C	T	C	Accurate
Marker6277	LG09	129.95	Chr. 9	21166887	G	A	G	A	G	Accurate
Marker6264	LG09	131.56	Chr. 9	19767697	G	A	G	A	G	Accurate
Marker6317	LG09	133.18	Chr. 9	21989042	G	G	A	G	A	Accurate
Marker6327	LG09	135.33	Chr. 9	22695555	G	G	A	G	A	Accurate
Marker6286	LG09	136.40	Chr. 9	21440658	G	A	G	A	G	Accurate
Marker6294	LG09	136.40	Chr. 9	21737710	G	G	C	G	C	Accurate
Marker6298	LG09	136.40	Chr. 9	21769527	G	G	C	G	C	Accurate
Marker6302	LG09	136.40	Chr. 9	21802232	A	A	G	A	G	Accurate
Marker6308	LG09	136.40	Chr. 9	21854973	T	T	C	T	C	Accurate
Marker6314	LG09	136.40	Chr. 9	21920101	T	T	C	T	C	Accurate
Marker6320	LG09	136.40	Chr. 9	22031458	G	G	A	G	A	Accurate
Marker6324	LG09	136.40	Chr. 9	22058770	C	C	T	C	T	Accurate
Marker6329	LG09	136.40	Chr. 9	22699650	C	T	C	T	C	Accurate
Marker6309	LG09	136.94	Chr. 9	21854982	G	G	A	G	A	Accurate
Marker6316	LG09	136.94	Chr. 9	21978385	G	G	C	G	C	Accurate
Marker6330	LG09	136.94	Chr. 9	22699824	C	C	T	C	T	Accurate
Marker6293	LG09	137.48	Chr. 9	21706402	A	A	T	A	T	Accurate
Marker6322	LG09	137.48	Chr. 9	22045747	G	G	T	G	T	Accurate
Marker6328	LG09	137.48	Chr. 9	22699644	G	G	A	G	A	Accurate
Marker6291	LG09	138.02	Chr. 9	21618745	T	T	C	T	C	Accurate
Marker6287	LG09	139.63	Chr. 9	21453359	G	C	G	C	G	Accurate
Marker6288	LG09	139.63	Chr. 9	21461106	G	A	G	A	G	Accurate
Marker6290	LG09	139.63	Chr. 9	21606041	G	G	C	G	C	Accurate
Marker6276	LG09	140.17	Chr. 9	21133721	T	C	T	C	T	Accurate
Marker6269	LG09	140.71	Chr. 9	20777224	A	G	A	G	A	Accurate
Marker6271	LG09	141.24	Chr. 9	20778334	A	A	T	A	T	Accurate
Marker6273	LG09	141.24	Chr. 9	20808975	C	C	A	C	A	Accurate
Marker6274	LG09	141.24	Chr. 9	21090370	A	A	G	A	G	Accurate
Marker6278	LG09	141.24	Chr. 9	21188908	T	A	T	A	T	Accurate
Marker6281	LG09	141.24	Chr. 9	21249045	C	T	C	T	C	Accurate
Marker6284	LG09	141.24	Chr. 9	21303638	C	T	C	T	C	Accurate
Marker6311	LG09	141.24	Chr. 9	21855480	G	G	A	G	A	Accurate
Marker6280	LG09	141.78	Chr. 9	21245625	A	G	A	G	A	Accurate
Marker6285	LG09	141.78	Chr. 9	21336568	C	T	C	T	C	Accurate
Marker6305	LG09	141.78	Chr. 9	21831681	C	C	T	C	T	Accurate
Marker6307	LG09	141.78	Chr. 9	21854070	A	A	G	A	G	Accurate
Marker6312	LG09	141.78	Chr. 9	21855497	C	C	A	C	A	Accurate
Marker6591	LG09	141.79	Chr. 9	29431640	T	G	T	G	T	Accurate
Marker6596	LG09	142.32	Chr. 9	29500531	T	C	T	C	T	Accurate
Marker6597	LG09	143.08	Chr. 9	29532210	G	G	A	G	A	Accurate
Marker6603	LG09	143.08	Chr. 9	29543852	G	G	A	G	A	Accurate

Marker6589	LG09	143.84	Chr. 9	29419723	C	C	A	C	A	Accurate
Marker6592	LG09	144.38	Chr. 9	29466798	T	C	T	C	T	Accurate
Marker6593	LG09	145.46	Chr. 9	29466825	G	T	G	T	G	Accurate
Marker6595	LG09	145.46	Chr. 9	29497074	C	T	C	T	C	Accurate
Marker6598	LG09	146.53	Chr. 9	29533164	A	A	C	A	C	Accurate
Marker6605	LG09	148.79	Chr. 9	29575770	T	T	A	T	A	Accurate
Marker6607	LG09	148.79	Chr. 9	30192346	A	G	A	G	A	Accurate
Marker6613	LG09	148.79	Chr. 9	30956887	A	A	G	A	G	Accurate
Marker6608	LG09	152.13	Chr. 9	30260292	T	C	T	C	T	Accurate
Marker6609	LG09	152.67	Chr. 9	30260502	G	A	G	A	G	Accurate
Marker6611	LG09	153.75	Chr. 9	30897247	G	A	G	A	G	Accurate
Marker6627	LG09	155.36	Chr. 9	31070364	A	A	T	A	T	Accurate
Marker6629	LG09	155.36	Chr. 9	31076155	T	T	C	T	C	Accurate
Marker6630	LG09	155.90	Chr. 9	31095085	T	T	C	T	C	Accurate
Marker6628	LG09	157.51	Chr. 9	31070554	G	G	A	G	A	Accurate
Marker6610	LG09	158.59	Chr. 9	30896329	A	C	A	C	A	Accurate
Marker6617	LG09	158.59	Chr. 9	31040016	G	A	G	A	G	Accurate
Marker6615	LG09	159.66	Chr. 9	31031352	T	A	T	A	T	Accurate
Marker6626	LG09	159.66	Chr. 9	31043671	T	C	T	C	N	Uncertain
Marker6616	LG09	160.20	Chr. 9	31039845	C	T	C	T	C	Accurate
Marker6632	LG09	160.74	Chr. 9	31097965	T	T	C	T	C	Accurate
Marker6639	LG09	161.28	Chr. 9	31451506	A	A	G	A	G	Accurate
Marker6636	LG09	161.82	Chr. 9	31409648	G	G	A	G	A	Accurate
Marker6638	LG09	162.35	Chr. 9	31450658	T	T	C	T	C	Accurate
Marker6612	LG09	163.43	Chr. 9	30952052	A	T	A	T	A	Accurate
Marker6635	LG09	163.97	Chr. 9	31406144	G	G	A	N	A	Uncertain
Marker6637	LG09	163.97	Chr. 9	31448394	C	C	A	C	A	Accurate
Marker6634	LG09	164.73	Chr. 9	31405906	A	A	T	A	T	Accurate
Marker6641	LG09	164.73	Chr. 9	31684119	A	A	C	A	C	Accurate
Marker6640	LG09	166.88	Chr. 9	31608779	G	G	A	N	A	Uncertain
Marker6643	LG09	167.42	Chr. 9	31708705	C	C	A	C	A	Accurate
Marker6642	LG09	169.03	Chr. 9	31708474	T	T	C	T	C	Accurate
Marker6644	LG09	169.03	Chr. 9	31922776	G	G	A	G	A	Accurate
Marker6658	LG09	171.72	Chr. 9	33011414	T	C	T	C	T	Accurate
Marker6653	LG09	173.34	Chr. 9	32827692	C	G	C	G	C	Accurate
Marker6663	LG09	173.34	Chr. 9	33062807	C	G	C	G	C	Accurate
Marker6666	LG09	173.34	Chr. 9	33119756	T	A	T	A	T	Accurate
Marker6654	LG09	173.88	Chr. 9	32833929	T	C	T	C	T	Accurate
Marker6664	LG09	173.88	Chr. 9	33070066	C	G	C	G	C	Accurate
Marker6675	LG09	174.41	Chr. 9	33659455	C	T	C	T	C	Accurate
Marker6668	LG09	176.56	Chr. 9	33131761	C	T	C	T	C	Accurate
Marker6660	LG09	179.25	Chr. 9	33049936	T	C	T	C	T	Accurate
Marker6672	LG09	179.25	Chr. 9	33530264	G	G	T	G	T	Accurate
Marker6659	LG09	180.33	Chr. 9	33012934	C	C	A	C	A	Accurate
Marker6669	LG09	180.87	Chr. 9	33511764	C	A	C	A	C	Accurate
Marker6686	LG09	181.40	Chr. 9	33970494	G	A	G	A	G	Accurate
Marker6691	LG09	181.40	Chr. 9	34049724	G	T	G	T	G	Accurate

Marker6687	LG09	181.94	Chr. 9	33994400	G	C	G	C	G	Accurate
Marker6693	LG09	182.48	Chr. 9	34128530	G	A	G	A	G	Accurate
Marker6696	LG09	182.48	Chr. 9	34215760	G	C	G	C	G	Accurate
Marker6698	LG09	184.09	Chr. 9	34324113	A	T	A	T	A	Accurate
Marker6710	LG09	184.09	Chr. 9	34900989	A	G	A	G	A	Accurate
Marker6697	LG09	184.63	Chr. 9	34226758	A	T	A	T	A	Accurate
Marker6701	LG09	184.63	Chr. 9	34429030	G	T	G	T	G	Accurate
Marker6702	LG09	184.63	Chr. 9	34443418	A	G	A	G	A	Accurate
Marker6700	LG09	185.71	Chr. 9	34425848	T	G	T	K	T	Uncertain
Marker6690	LG09	186.79	Chr. 9	34049438	A	C	A	C	A	Accurate
Marker6712	LG09	186.79	Chr. 9	34948403	C	T	C	T	C	Accurate
Marker6707	LG09	187.86	Chr. 9	34816931	G	A	G	A	G	Accurate
Marker6708	LG09	191.01	Chr. 9	34851155	C	T	C	T	C	Accurate
Marker6775	LG10	0.54	Chr. 1(	2045372	C	T	C	T	C	Accurate
Marker6783	LG10	0.54	Chr. 1(	2085495	T	C	T	C	T	Accurate
Marker6759	LG10	1.08	Chr. 1(	1712682	A	G	A	G	A	Accurate
Marker6760	LG10	1.08	Chr. 1(	1719126	G	A	G	A	G	Accurate
Marker6761	LG10	1.08	Chr. 1(	1733354	A	C	A	C	A	Accurate
Marker6777	LG10	1.08	Chr. 1(	2066353	C	T	C	T	C	Accurate
Marker6780	LG10	1.08	Chr. 1(	2070696	A	T	A	T	A	Accurate
Marker6782	LG10	1.08	Chr. 1(	2084516	T	G	T	G	T	Accurate
Marker6766	LG10	1.61	Chr. 1(	1885168	A	T	A	T	A	Accurate
Marker6765	LG10	3.23	Chr. 1(	1883297	T	A	T	A	T	Accurate
Marker6762	LG10	4.84	Chr. 1(	1866204	C	G	C	G	C	Accurate
Marker6728	LG10	7.10	Chr. 1(	1139254	G	G	A	G	A	Accurate
Marker6739	LG10	7.10	Chr. 1(	1296972	C	T	C	T	C	Accurate
Marker6719	LG10	7.64	Chr. 1(	1030036	C	T	C	T	C	Accurate
Marker6722	LG10	7.64	Chr. 1(	1072501	T	T	C	T	C	Accurate
Marker6727	LG10	7.64	Chr. 1(	1129845	A	A	T	A	T	Accurate
Marker6730	LG10	7.64	Chr. 1(	1144154	G	G	A	G	A	Accurate
Marker6733	LG10	7.64	Chr. 1(	1268743	A	G	A	G	A	Accurate
Marker6736	LG10	7.64	Chr. 1(	1276218	T	C	T	C	T	Accurate
Marker6744	LG10	7.64	Chr. 1(	1301602	G	A	G	A	G	Accurate
Marker6747	LG10	7.64	Chr. 1(	1322178	A	G	A	G	A	Accurate
Marker6756	LG10	7.64	Chr. 1(	1352815	G	A	G	A	G	Accurate
Marker6726	LG10	8.18	Chr. 1(	1128936	G	G	C	G	C	Accurate
Marker6749	LG10	8.18	Chr. 1(	1322435	T	C	T	C	N	Uncertain
Marker6753	LG10	8.18	Chr. 1(	1336247	C	A	C	A	C	Accurate
Marker6754	LG10	8.18	Chr. 1(	1350101	T	G	T	G	T	Accurate
Marker6741	LG10	8.72	Chr. 1(	1297609	C	T	C	T	C	Accurate
Marker6731	LG10	9.79	Chr. 1(	1166893	G	G	A	G	A	Accurate
Marker6732	LG10	10.33	Chr. 1(	1260636	T	T	C	T	C	Accurate
Marker6716	LG10	13.90	Chr. 1(	202209	A	A	G	A	G	Accurate
Marker6791	LG10	17.57	Chr. 1(	2453895	G	G	A	G	A	Accurate
Marker6787	LG10	18.65	Chr. 1(	2391048	T	T	C	T	C	Accurate
Marker6788	LG10	19.72	Chr. 1(	2400370	C	C	T	C	T	Accurate
Marker6798	LG10	20.80	Chr. 1(	2831343	G	A	G	A	G	Accurate

Marker6801	LG10	20.80	Chr. 1(	2907131	G	G	A	G	A	Accurate
Marker6804	LG10	20.80	Chr. 1(	2968823	G	A	G	A	G	Accurate
Marker6807	LG10	20.80	Chr. 1(	2990839	A	C	A	M	A	Uncertain
Marker6802	LG10	21.34	Chr. 1(	2918184	T	T	A	T	A	Accurate
Marker6803	LG10	21.34	Chr. 1(	2922045	G	G	A	G	A	Accurate
Marker6799	LG10	21.88	Chr. 1(	2831665	G	G	A	G	A	Accurate
Marker6800	LG10	22.42	Chr. 1(	2862155	A	T	A	T	A	Accurate
Marker6808	LG10	24.03	Chr. 1(	3674879	T	C	T	C	T	Accurate
Marker6819	LG10	26.18	Chr. 1(	3760649	G	A	G	A	G	Accurate
Marker6832	LG10	27.26	Chr. 1(	4064377	A	C	A	C	A	Accurate
Marker6829	LG10	29.41	Chr. 1(	4049005	T	C	T	C	T	Accurate
Marker6818	LG10	30.49	Chr. 1(	3750669	A	G	A	G	A	Accurate
Marker6833	LG10	31.02	Chr. 1(	4072117	A	G	A	G	A	Accurate
Marker6831	LG10	31.56	Chr. 1(	4063581	C	T	C	T	C	Accurate
Marker6821	LG10	33.17	Chr. 1(	3828836	A	G	A	G	A	Accurate
Marker6843	LG10	34.25	Chr. 1(	4887194	A	G	A	G	A	Accurate
Marker6845	LG10	34.79	Chr. 1(	4964567	C	T	C	T	C	Accurate
Marker6851	LG10	34.79	Chr. 1(	4996545	A	C	A	C	A	Accurate
Marker6844	LG10	36.51	Chr. 1(	4916547	A	T	A	T	A	Accurate
Marker6838	LG10	39.31	Chr. 1(	4354656	G	A	G	A	G	Accurate
Marker6839	LG10	40.38	Chr. 1(	4357366	G	T	G	T	G	Accurate
Marker6857	LG10	45.93	Chr. 1(	5658711	A	G	A	G	A	Accurate
Marker6888	LG10	57.04	Chr. 1(	13768197	C	T	C	T	C	Accurate
Marker6870	LG10	58.11	Chr. 1(	13613939	A	T	A	T	A	Accurate
Marker6874	LG10	58.11	Chr. 1(	13674173	A	G	A	G	A	Accurate
Marker6866	LG10	59.19	Chr. 1(	13544102	A	G	A	G	A	Accurate
Marker6867	LG10	59.19	Chr. 1(	13548667	C	T	C	T	C	Accurate
Marker6871	LG10	59.19	Chr. 1(	13615856	T	G	T	G	T	Accurate
Marker6872	LG10	59.19	Chr. 1(	13639890	T	C	T	Y	T	Uncertain
Marker6873	LG10	59.19	Chr. 1(	13674081	C	T	C	T	C	Accurate
Marker6877	LG10	59.19	Chr. 1(	13688367	T	A	T	A	T	Accurate
Marker6880	LG10	59.73	Chr. 1(	13707214	T	C	T	C	T	Accurate
Marker6883	LG10	59.73	Chr. 1(	13732182	A	G	A	G	A	Accurate
Marker6881	LG10	60.80	Chr. 1(	13731925	C	T	C	T	C	Accurate
Marker6887	LG10	62.95	Chr. 1(	13765346	A	G	A	G	A	Accurate
Marker6875	LG10	66.12	Chr. 1(	13683086	A	G	A	G	A	Accurate
Marker6876	LG10	67.97	Chr. 1(	13683089	C	T	C	T	C	Accurate
Marker6937	LG10	78.60	Chr. 1(	18106220	G	T	G	T	G	Accurate
Marker6932	LG10	80.75	Chr. 1(	17871116	T	A	T	A	T	Accurate
Marker6939	LG10	81.29	Chr. 1(	18115463	C	T	C	T	C	Accurate
Marker6923	LG10	83.44	Chr. 1(	17563465	G	A	G	A	G	Accurate
Marker6909	LG10	83.98	Chr. 1(	17279516	A	C	A	C	A	Accurate
Marker6911	LG10	83.98	Chr. 1(	17439224	A	G	A	G	A	Accurate
Marker6916	LG10	83.98	Chr. 1(	17518954	T	C	T	C	T	Accurate
Marker6919	LG10	83.98	Chr. 1(	17541469	G	A	G	A	G	Accurate
Marker6913	LG10	84.52	Chr. 1(	17473997	A	G	A	G	A	Accurate
Marker6925	LG10	84.52	Chr. 1(	17565467	G	A	G	A	G	Accurate

Marker6929	LG10	84.52	Chr. 1(	17663143	G	A	G	A	G	Accurate
Marker6924	LG10	85.05	Chr. 1(	17564910	T	C	T	C	T	Accurate
Marker6947	LG10	86.67	Chr. 1(	18537049	T	A	T	A	T	Accurate
Marker6934	LG10	87.74	Chr. 1(	18005264	G	T	G	T	G	Accurate
Marker6942	LG10	89.36	Chr. 1(	18399127	A	C	A	C	A	Accurate
Marker6948	LG10	90.43	Chr. 1(	18553326	C	T	C	T	C	Accurate
Marker6943	LG10	92.69	Chr. 1(	18478448	A	T	A	T	A	Accurate
Marker6956	LG10	97.17	Chr. 1(	19878408	C	T	C	T	C	Accurate
Marker6967	LG10	100.72	Chr. 1(	20078826	T	C	T	C	T	Accurate
Marker6971	LG10	102.87	Chr. 1(	20156867	G	C	G	C	G	Accurate
Marker6972	LG10	102.87	Chr. 1(	20174554	T	A	T	A	T	Accurate
Marker6975	LG10	102.87	Chr. 1(	20197020	A	T	A	T	A	Accurate
Marker6980	LG10	102.87	Chr. 1(	20234928	T	C	T	C	T	Accurate
Marker6965	LG10	103.41	Chr. 1(	20064456	T	C	T	C	T	Accurate
Marker6958	LG10	103.95	Chr. 1(	19905537	A	G	A	G	N	Uncertain
Marker6970	LG10	103.95	Chr. 1(	20105526	C	G	C	G	C	Accurate
Marker6961	LG10	104.49	Chr. 1(	19972258	A	T	A	T	A	Accurate
Marker6966	LG10	104.49	Chr. 1(	20065451	A	G	A	G	A	Accurate
Marker6976	LG10	104.49	Chr. 1(	20209152	T	C	T	C	T	Accurate
Marker6974	LG10	107.29	Chr. 1(	20175075	T	C	T	C	N	Uncertain
Marker6973	LG10	108.36	Chr. 1(	20174841	C	T	C	T	C	Accurate
Marker6994	LG10	116.85	Chr. 1(	22057929	A	C	A	C	A	Accurate
Marker6997	LG10	118.46	Chr. 1(	22070023	G	A	G	A	G	Accurate
Marker6993	LG10	120.07	Chr. 1(	22042019	A	G	A	G	A	Accurate
Marker6992	LG10	121.38	Chr. 1(	22041716	G	A	G	A	G	Accurate
Marker6988	LG10	121.93	Chr. 1(	22020545	T	A	T	A	T	Accurate
Marker6989	LG10	121.93	Chr. 1(	22022492	G	T	G	T	G	Accurate
Marker7008	LG10	122.46	Chr. 1(	22289123	T	C	T	C	T	Accurate
Marker7002	LG10	123.54	Chr. 1(	22246185	T	T	C	T	C	Accurate
Marker7011	LG10	124.61	Chr. 1(	22363749	C	C	A	C	A	Accurate
Marker7013	LG10	125.15	Chr. 1(	22400809	A	C	A	C	A	Accurate
Marker7016	LG10	125.69	Chr. 1(	22430967	T	T	C	T	C	Accurate
Marker7001	LG10	126.77	Chr. 1(	22223558	G	G	C	G	C	Accurate
Marker7003	LG10	127.84	Chr. 1(	22248966	G	G	A	G	A	Accurate
Marker7022	LG10	128.92	Chr. 1(	23057026	C	C	T	C	T	Accurate
Marker7021	LG10	129.45	Chr. 1(	23035313	A	A	G	A	G	Accurate
Marker7024	LG10	130.53	Chr. 1(	23276412	A	A	G	A	G	Accurate
Marker7025	LG10	132.37	Chr. 1(	23452324	C	C	T	C	T	Accurate
Marker7031	LG10	134.21	Chr. 1(	23980840	T	C	T	C	T	Accurate
Marker7034	LG10	135.63	Chr. 1(	24103391	C	T	C	T	C	Accurate
Marker7037	LG10	137.25	Chr. 1(	24141371	A	C	A	C	A	Accurate
Marker7035	LG10	138.32	Chr. 1(	24113012	G	C	G	C	G	Accurate
Marker7038	LG10	140.69	Chr. 1(	24504287	T	G	T	G	T	Accurate
Marker7039	LG10	142.31	Chr. 1(	24607971	C	T	C	T	C	Accurate
Marker7049	LG10	144.57	Chr. 1(	25187140	G	A	G	A	G	Accurate
Marker7043	LG10	146.18	Chr. 1(	24762062	T	T	A	T	A	Accurate
Marker7057	LG10	150.29	Chr. 1(	26547911	G	G	A	G	A	Accurate

Marker7059	LG10	150.29	Chr. 10	26566207	T	T	G	T	G	Accurate
Marker7060	LG10	151.58	Chr. 10	26663293	A	A	G	N	G	Uncertain
Marker7061	LG10	152.12	Chr. 10	26663464	G	G	T	G	T	Accurate
Marker7066	LG10	153.20	Chr. 10	26688803	A	A	C	A	C	Accurate
Marker7067	LG10	154.81	Chr. 10	26689031	G	G	A	G	A	Accurate
Marker7081	LG10	155.89	Chr. 10	26970069	A	G	A	G	A	Accurate
Marker7084	LG10	156.42	Chr. 10	26974434	C	T	C	T	C	Accurate
Marker7063	LG10	156.96	Chr. 10	26678022	T	T	A	T	A	Accurate
Marker7074	LG10	156.96	Chr. 10	26949526	T	A	T	A	T	Accurate
Marker7082	LG10	156.96	Chr. 10	26970185	T	C	T	C	T	Accurate
Marker7091	LG10	159.65	Chr. 10	27060743	A	A	G	A	G	Accurate
Marker7087	LG10	160.19	Chr. 10	27016754	G	A	G	A	G	Accurate
Marker7093	LG10	160.19	Chr. 10	27062569	C	C	T	C	T	Accurate
Marker7095	LG10	160.19	Chr. 10	27102778	C	C	T	C	T	Accurate
Marker7101	LG10	160.19	Chr. 10	27480792	A	T	A	T	A	Accurate
Marker7103	LG10	160.19	Chr. 10	27777487	A	G	A	G	A	Accurate
Marker7669	LG11	0.00	Chr. 11	24282701	A	G	A	G	A	Accurate
Marker7667	LG11	0.54	Chr. 11	24238954	T	C	T	C	T	Accurate
Marker7671	LG11	0.54	Chr. 11	24292896	T	G	T	G	T	Accurate
Marker7668	LG11	1.08	Chr. 11	24276847	A	G	A	G	A	Accurate
Marker7666	LG11	1.61	Chr. 11	24208785	A	G	A	G	A	Accurate
Marker7662	LG11	3.23	Chr. 11	23875152	G	T	G	T	G	Accurate
Marker7661	LG11	4.30	Chr. 11	23828325	A	G	A	G	A	Accurate
Marker7660	LG11	4.84	Chr. 11	23780496	G	C	G	C	G	Accurate
Marker7629	LG11	9.25	Chr. 11	23230005	C	T	C	T	C	Accurate
Marker7628	LG11	9.79	Chr. 11	23229959	A	T	A	T	A	Accurate
Marker7641	LG11	10.86	Chr. 11	23438058	T	C	T	C	T	Accurate
Marker7646	LG11	10.86	Chr. 11	23518427	T	A	T	A	T	Accurate
Marker7654	LG11	11.94	Chr. 11	23642257	T	C	T	C	T	Accurate
Marker7659	LG11	12.48	Chr. 11	23672235	A	C	A	C	A	Accurate
Marker7651	LG11	13.55	Chr. 11	23598864	A	C	A	C	A	Accurate
Marker7649	LG11	15.17	Chr. 11	23571442	T	C	T	C	T	Accurate
Marker7634	LG11	16.24	Chr. 11	23368607	C	T	C	T	C	Accurate
Marker7635	LG11	16.24	Chr. 11	23388452	G	A	G	A	G	Accurate
Marker7636	LG11	16.24	Chr. 11	23397258	T	C	T	C	T	Accurate
Marker7642	LG11	16.24	Chr. 11	23474197	C	T	C	T	C	Accurate
Marker7647	LG11	16.78	Chr. 11	23539644	T	C	T	C	T	Accurate
Marker7640	LG11	18.39	Chr. 11	23426391	G	T	G	T	G	Accurate
Marker7643	LG11	23.79	Chr. 11	23500079	G	T	G	T	G	Accurate
Marker7623	LG11	32.90	Chr. 11	21973390	T	C	T	C	T	Accurate
Marker7620	LG11	33.98	Chr. 11	21859415	T	C	T	C	T	Accurate
Marker7616	LG11	34.73	Chr. 11	21851356	A	C	A	C	A	Accurate
Marker7625	LG11	34.73	Chr. 11	22136681	G	A	G	A	G	Accurate
Marker7621	LG11	35.27	Chr. 11	21861291	A	G	A	G	A	Accurate
Marker7607	LG11	36.35	Chr. 11	21797600	G	A	G	A	G	Accurate
Marker7590	LG11	37.11	Chr. 11	21171254	C	T	C	T	C	Accurate
Marker7591	LG11	37.11	Chr. 11	21186313	C	A	C	A	C	Accurate

Marker7150	LG11	38.19	Chr. 11	2141356	G	A	G	A	G	Accurate
Marker7585	LG11	38.95	Chr. 11	21096698	G	T	G	T	G	Accurate
Marker7148	LG11	39.48	Chr. 11	2134425	G	A	G	A	G	Accurate
Marker7146	LG11	43.90	Chr. 11	1660496	G	T	G	T	G	Accurate
Marker7143	LG11	46.28	Chr. 11	1615944	A	T	A	T	A	Accurate
Marker7142	LG11	48.43	Chr. 11	1436718	G	A	G	A	G	Accurate
Marker7136	LG11	48.97	Chr. 11	1399576	A	G	A	G	A	Accurate
Marker7135	LG11	51.66	Chr. 11	1399057	G	A	G	A	G	Accurate
Marker7129	LG11	53.81	Chr. 11	1247133	T	C	T	C	T	Accurate
Marker7127	LG11	55.42	Chr. 11	1118935	T	C	T	C	T	Accurate
Marker7126	LG11	57.04	Chr. 11	966810	A	T	A	T	A	Accurate
Marker7118	LG11	57.57	Chr. 11	804614	C	T	C	T	C	Accurate
Marker7121	LG11	58.11	Chr. 11	845337	C	T	C	T	C	Accurate
Marker7125	LG11	58.11	Chr. 11	927267	T	C	T	C	T	Accurate
Marker7112	LG11	58.65	Chr. 11	738898	T	C	T	C	T	Accurate
Marker7117	LG11	58.65	Chr. 11	794072	T	A	T	A	T	Accurate
Marker7119	LG11	58.65	Chr. 11	813281	A	G	A	G	A	Accurate
Marker7114	LG11	59.19	Chr. 11	771249	G	A	G	A	N	Uncertain
Marker7120	LG11	59.19	Chr. 11	813300	T	A	T	A	T	Accurate
Marker7123	LG11	59.19	Chr. 11	887771	G	A	G	A	G	Accurate
Marker7107	LG11	60.26	Chr. 11	653436	A	G	A	G	A	Accurate
Marker7966	LG11	60.80	Chr. 0	19991787	C	T	C	T	C	Accurate
Marker7956	LG11	61.88	Chr. 0	19917147	A	G	A	G	A	Accurate
Marker7960	LG11	61.88	Chr. 0	19948808	C	T	C	T	C	Accurate
Marker7965	LG11	61.88	Chr. 0	19991374	T	G	T	G	T	Accurate
Marker7968	LG11	61.88	Chr. 0	20022125	T	C	T	C	T	Accurate
Marker7740	LG11	61.88	Chr. 0	5586078	G	A	G	A	G	Accurate
Marker7290	LG11	61.88	Chr. 11	6981310	G	A	G	A	G	Accurate
Marker7296	LG11	61.88	Chr. 11	7204155	T	G	T	G	T	Accurate
Marker7302	LG11	61.88	Chr. 11	7395155	C	T	C	T	C	Accurate
Marker7309	LG11	61.88	Chr. 11	7558759	A	G	A	G	A	Accurate
Marker7313	LG11	61.88	Chr. 11	7605139	G	A	G	A	G	Accurate
Marker7270	LG11	63.49	Chr. 11	4522526	A	C	A	C	A	Accurate
Marker7274	LG11	64.56	Chr. 11	4781627	T	G	T	G	T	Accurate
Marker7258	LG11	65.64	Chr. 11	4346992	C	T	C	T	C	Accurate
Marker7264	LG11	66.18	Chr. 11	4435577	G	A	G	A	G	Accurate
Marker7269	LG11	66.18	Chr. 11	4522270	G	A	G	A	G	Accurate
Marker7211	LG11	67.25	Chr. 11	3737294	T	C	T	C	T	Accurate
Marker7231	LG11	67.25	Chr. 11	3885176	T	C	T	C	T	Accurate
Marker7237	LG11	67.25	Chr. 11	4064576	C	T	C	T	C	Accurate
Marker7240	LG11	67.25	Chr. 11	4146285	T	C	T	C	T	Accurate
Marker7173	LG11	67.79	Chr. 11	2869804	A	G	A	G	A	Accurate
Marker7177	LG11	67.79	Chr. 11	2919069	C	C	G	C	G	Accurate
Marker7203	LG11	67.79	Chr. 11	3505030	T	T	C	T	C	Accurate
Marker7210	LG11	67.79	Chr. 11	3718323	A	G	A	G	A	Accurate
Marker7221	LG11	67.79	Chr. 11	3748145	T	T	C	T	C	Accurate
Marker7224	LG11	67.79	Chr. 11	3777762	T	T	C	T	C	Accurate

Marker7225	LG11	67.79	Chr. 11	3799484	A	T	A	T	A	Accurate
Marker7226	LG11	67.79	Chr. 11	3803297	C	T	C	T	C	Accurate
Marker7239	LG11	68.87	Chr. 11	4136045	G	A	G	A	G	Accurate
Marker7245	LG11	68.87	Chr. 11	4167022	A	C	A	C	A	Accurate
Marker7246	LG11	68.87	Chr. 11	4191607	T	C	T	C	T	Accurate
Marker7251	LG11	68.87	Chr. 11	4262970	G	A	G	A	G	Accurate
Marker7257	LG11	68.87	Chr. 11	4341112	G	A	G	A	G	Accurate
Marker7263	LG11	68.87	Chr. 11	4432871	A	G	A	G	A	Accurate
Marker7229	LG11	69.40	Chr. 11	3872264	A	T	A	T	A	Accurate
Marker7249	LG11	69.40	Chr. 11	4239882	T	C	T	C	T	Accurate
Marker7262	LG11	69.40	Chr. 11	4419862	A	G	A	G	A	Accurate
Marker7209	LG11	70.48	Chr. 11	3716094	G	A	G	A	G	Accurate
Marker7166	LG11	71.78	Chr. 11	2717473	G	A	G	A	G	Accurate
Marker7165	LG11	72.85	Chr. 11	2717010	T	C	T	C	T	Accurate
Marker7181	LG11	73.39	Chr. 11	3096896	A	T	A	T	A	Accurate
Marker7155	LG11	74.46	Chr. 11	2587038	C	T	C	T	C	Accurate
Marker7151	LG11	75.54	Chr. 11	2570463	T	C	T	C	T	Accurate
Marker7153	LG11	77.15	Chr. 11	2574883	A	T	A	T	A	Accurate
Marker7152	LG11	77.69	Chr. 11	2574860	G	A	G	A	G	Accurate
Marker7160	LG11	78.77	Chr. 11	2695249	G	G	A	G	A	Accurate
Marker7161	LG11	79.30	Chr. 11	2695288	G	G	C	G	C	Accurate
Marker7163	LG11	79.84	Chr. 11	2699831	A	C	A	C	A	Accurate
Marker8016	LG11	81.99	Chr. 0	22646973	G	A	G	A	G	Accurate
Marker8017	LG11	81.99	Chr. 0	22662867	C	G	C	G	C	Accurate
Marker7367	LG11	84.79	Chr. 11	10712216	C	C	A	C	A	Accurate
Marker7355	LG11	85.87	Chr. 11	10133062	A	T	A	T	A	Accurate
Marker7359	LG11	87.48	Chr. 11	10416919	A	A	G	A	G	Accurate
Marker7365	LG11	88.56	Chr. 11	10580535	C	T	C	T	C	Accurate
Marker7334	LG11	90.94	Chr. 11	8927765	C	C	A	C	A	Accurate
Marker7335	LG11	90.94	Chr. 11	8985529	T	T	A	T	A	Accurate
Marker7322	LG11	91.47	Chr. 11	8691285	C	C	T	C	T	Accurate
Marker7326	LG11	92.55	Chr. 11	8760035	C	C	T	C	T	Accurate
Marker7325	LG11	93.09	Chr. 11	8736444	G	G	A	G	A	Accurate
Marker7327	LG11	93.09	Chr. 11	8762750	G	G	T	G	T	Accurate
Marker7331	LG11	93.09	Chr. 11	8898716	T	T	G	T	G	Accurate
Marker7336	LG11	93.09	Chr. 11	9013749	G	G	A	G	A	Accurate
Marker7321	LG11	94.70	Chr. 11	8688440	G	G	A	G	A	Accurate
Marker7424	LG11	101.73	Chr. 11	12008886	C	T	C	T	C	Accurate
Marker7445	LG11	103.35	Chr. 11	12583756	C	C	T	C	T	Accurate
Marker7446	LG11	103.35	Chr. 11	12591725	A	A	G	A	G	Accurate
Marker7452	LG11	103.89	Chr. 11	12701208	T	T	C	T	C	Accurate
Marker7444	LG11	104.96	Chr. 11	12573599	G	G	T	G	T	Accurate
Marker7458	LG11	106.14	Chr. 11	12822500	C	T	C	T	C	Accurate
Marker7447	LG11	106.68	Chr. 11	12592145	G	G	A	G	A	Accurate
Marker7453	LG11	108.40	Chr. 11	12706572	T	T	C	T	C	Accurate
Marker7483	LG11	112.10	Chr. 11	14370203	A	T	A	T	A	Accurate
Marker7484	LG11	112.63	Chr. 11	14370373	T	C	T	C	T	Accurate

Marker7494	LG11	114.25	Chr. 11	14599272	G	G	T	G	T	Accurate
Marker7485	LG11	115.32	Chr. 11	14370406	A	G	A	G	A	Accurate
Marker7491	LG11	115.32	Chr. 11	14389677	A	C	A	C	A	Accurate
Marker7497	LG11	117.70	Chr. 11	14811417	C	C	T	C	T	Accurate
Marker7495	LG11	119.31	Chr. 11	14658879	C	A	C	A	C	Accurate
Marker7496	LG11	121.57	Chr. 11	14807524	A	A	G	A	G	Accurate
Marker7498	LG11	123.29	Chr. 11	14866119	A	A	T	A	T	Accurate
Marker7509	LG11	124.90	Chr. 11	15574673	G	A	G	A	G	Accurate
Marker7530	LG11	125.98	Chr. 11	15832246	C	C	G	C	G	Accurate
Marker7533	LG11	126.52	Chr. 11	15868742	A	A	G	A	G	Accurate
Marker7539	LG11	127.05	Chr. 11	15920522	C	T	C	T	C	Accurate
Marker7528	LG11	127.59	Chr. 11	15819054	G	G	A	G	A	Accurate
Marker7529	LG11	127.59	Chr. 11	15831815	C	C	T	C	T	Accurate
Marker7534	LG11	127.59	Chr. 11	15884639	C	T	C	T	C	Accurate
Marker7544	LG11	127.59	Chr. 11	15982474	C	A	C	A	C	Accurate
Marker7543	LG11	128.13	Chr. 11	15982239	C	T	C	T	C	Accurate
Marker7545	LG11	128.13	Chr. 11	15982525	C	G	C	G	C	Accurate
Marker7503	LG11	129.21	Chr. 11	15447317	T	T	A	T	A	Accurate
Marker7506	LG11	129.74	Chr. 11	15514665	C	C	T	C	T	Accurate
Marker7507	LG11	129.74	Chr. 11	15548663	C	C	T	C	T	Accurate
Marker7508	LG11	129.74	Chr. 11	15556913	G	A	G	A	G	Accurate
Marker7511	LG11	129.74	Chr. 11	15587422	C	C	T	C	T	Accurate
Marker7526	LG11	129.74	Chr. 11	15807760	A	A	T	A	T	Accurate
Marker7515	LG11	130.28	Chr. 11	15602602	T	A	T	A	T	Accurate
Marker7519	LG11	131.05	Chr. 11	15647505	C	T	C	T	C	Accurate
Marker7516	LG11	132.66	Chr. 11	15612084	C	T	C	T	C	Accurate
Marker7563	LG11	137.95	Chr. 11	17004093	C	T	C	T	C	Accurate
Marker7564	LG11	138.51	Chr. 11	17004312	G	A	G	A	G	Accurate
Marker7565	LG11	139.05	Chr. 11	17004340	A	G	A	G	A	Accurate
Marker7560	LG11	140.77	Chr. 11	16987177	G	C	G	C	G	Accurate
Marker7550	LG11	143.03	Chr. 11	16540616	A	C	A	C	A	Accurate
Marker7553	LG11	143.03	Chr. 11	16582151	A	G	A	G	A	Accurate
Marker7555	LG11	143.03	Chr. 11	16611056	C	T	C	T	C	Accurate
Marker7552	LG11	143.56	Chr. 11	16542217	G	T	G	T	G	Accurate
Marker7551	LG11	146.16	Chr. 11	16541091	T	C	T	C	T	Accurate
Marker7559	LG11	148.00	Chr. 11	16982961	C	T	C	T	C	Accurate
Marker7575	LG11	151.04	Chr. 11	17787270	A	T	A	T	A	Accurate
Marker7578	LG11	154.38	Chr. 11	18506691	G	T	G	T	G	Accurate
Marker7580	LG11	157.83	Chr. 11	18524685	A	G	A	G	A	Accurate
Marker1			Chr. 1	190060	G	C	G	C	G	Accurate
Marker2			Chr. 1	190101	A	G	A	G	A	Accurate
Marker6			Chr. 1	349739	A	G	A	G	A	Accurate
Marker17			Chr. 1	2116666	T	G	T	G	N	Uncertain
Marker18			Chr. 1	2117727	T	C	T	C	T	Accurate
Marker24			Chr. 1	2246800	A	A	C	A	C	Accurate
Marker25			Chr. 1	2255963	A	A	T	A	T	Accurate
Marker26			Chr. 1	2255973	A	A	T	A	T	Accurate

Marker29	Chr. 1	2267428	C	T	C	T	C	Accurate
Marker30	Chr. 1	2342636	C	C	T	C	T	Accurate
Marker31	Chr. 1	2342945	A	A	G	A	G	Accurate
Marker32	Chr. 1	2343903	T	T	G	T	G	Accurate
Marker36	Chr. 1	2526827	A	G	A	G	A	Accurate
Marker38	Chr. 1	2533102	T	C	T	C	T	Accurate
Marker39	Chr. 1	2545228	T	G	T	G	T	Accurate
Marker40	Chr. 1	2560613	T	C	T	C	T	Accurate
Marker43	Chr. 1	2653284	C	T	C	T	C	Accurate
Marker45	Chr. 1	2685260	G	A	G	A	G	Accurate
Marker51	Chr. 1	2767658	A	A	G	A	G	Accurate
Marker55	Chr. 1	2811432	C	C	T	C	T	Accurate
Marker56	Chr. 1	2811890	T	T	G	T	G	Accurate
Marker58	Chr. 1	2860498	T	T	C	T	C	Accurate
Marker60	Chr. 1	2900136	T	T	A	T	A	Accurate
Marker63	Chr. 1	2917094	A	A	C	A	C	Accurate
Marker64	Chr. 1	2924096	A	A	G	A	G	Accurate
Marker66	Chr. 1	2948279	T	T	C	T	C	Accurate
Marker69	Chr. 1	2961293	T	T	A	T	A	Accurate
Marker70	Chr. 1	2961294	T	T	A	T	A	Accurate
Marker75	Chr. 1	3212634	T	C	T	C	T	Accurate
Marker79	Chr. 1	3245205	C	T	C	T	N	Uncertain
Marker80	Chr. 1	3245445	C	T	C	T	C	Accurate
Marker81	Chr. 1	3261594	A	C	A	C	A	Accurate
Marker82	Chr. 1	3261628	T	A	T	A	T	Accurate
Marker83	Chr. 1	3261630	G	C	G	C	G	Accurate
Marker85	Chr. 1	3332565	C	T	C	T	N	Uncertain
Marker88	Chr. 1	3384275	C	T	C	T	C	Accurate
Marker89	Chr. 1	3679720	G	A	G	A	G	Accurate
Marker90	Chr. 1	3685928	A	C	A	C	A	Accurate
Marker91	Chr. 1	3807739	C	T	C	T	N	Uncertain
Marker92	Chr. 1	3808041	G	T	G	T	G	Accurate
Marker93	Chr. 1	3809071	G	A	G	A	N	Uncertain
Marker96	Chr. 1	3830267	A	G	A	G	A	Accurate
Marker97	Chr. 1	3832012	T	C	T	C	T	Accurate
Marker99	Chr. 1	3856351	A	G	A	G	A	Accurate
Marker101	Chr. 1	3981012	A	C	A	C	A	Accurate
Marker103	Chr. 1	4090610	T	C	T	C	T	Accurate
Marker108	Chr. 1	4148693	G	G	A	G	A	Accurate
Marker111	Chr. 1	4221304	T	C	T	C	T	Accurate
Marker112	Chr. 1	4317012	T	A	T	A	T	Accurate
Marker113	Chr. 1	4317016	G	A	G	A	G	Accurate
Marker116	Chr. 1	4544372	C	T	C	T	C	Accurate
Marker117	Chr. 1	4561444	A	G	A	G	A	Accurate
Marker121	Chr. 1	4576454	C	T	C	T	C	Accurate
Marker122	Chr. 1	4642469	G	A	G	A	G	Accurate
Marker125	Chr. 1	4794572	T	A	T	A	T	Accurate

Marker126	Chr. 1	4795028	T	A	T	A	T	Accurate
Marker128	Chr. 1	4798242	G	C	G	C	G	Accurate
Marker132	Chr. 1	4833509	G	T	G	T	G	Accurate
Marker144	Chr. 1	6400177	A	G	A	G	A	Accurate
Marker146	Chr. 1	6413293	C	T	C	T	C	Accurate
Marker160	Chr. 1	6644536	T	C	T	C	T	Accurate
Marker163	Chr. 1	6659600	C	T	C	T	C	Accurate
Marker164	Chr. 1	6758864	C	T	C	T	C	Accurate
Marker166	Chr. 1	6776568	T	C	T	C	T	Accurate
Marker173	Chr. 1	7040406	C	C	T	C	T	Accurate
Marker174	Chr. 1	7040438	T	T	C	T	C	Accurate
Marker175	Chr. 1	7043235	G	G	A	G	A	Accurate
Marker177	Chr. 1	7049597	A	A	G	A	G	Accurate
Marker183	Chr. 1	7142015	A	A	T	A	T	Accurate
Marker186	Chr. 1	7196009	A	A	G	A	G	Accurate
Marker187	Chr. 1	7218364	A	A	T	A	T	Accurate
Marker191	Chr. 1	7288761	C	C	T	C	T	Accurate
Marker193	Chr. 1	7292970	T	T	G	T	G	Accurate
Marker196	Chr. 1	7346485	A	A	G	A	G	Accurate
Marker198	Chr. 1	7382497	C	C	T	C	T	Accurate
Marker199	Chr. 1	7382690	A	A	G	A	G	Accurate
Marker201	Chr. 1	7401231	C	C	T	N	T	Uncertain
Marker203	Chr. 1	7402150	C	C	T	C	T	Accurate
Marker205	Chr. 1	7458567	G	G	A	G	A	Accurate
Marker209	Chr. 1	7832961	A	A	G	A	G	Accurate
Marker211	Chr. 1	8016291	A	A	C	A	C	Accurate
Marker215	Chr. 1	8159607	A	A	G	A	G	Accurate
Marker216	Chr. 1	8163766	G	G	A	G	A	Accurate
Marker219	Chr. 1	8422489	T	T	G	T	K	Uncertain
Marker220	Chr. 1	8425889	C	C	T	C	T	Accurate
Marker221	Chr. 1	8444520	G	G	A	G	A	Accurate
Marker222	Chr. 1	8444735	T	T	C	T	C	Accurate
Marker226	Chr. 1	8565544	T	C	T	C	T	Accurate
Marker229	Chr. 1	8788428	G	G	T	G	T	Accurate
Marker230	Chr. 1	8808896	T	A	C	C	C	Inconsistent
Marker231	Chr. 1	8930709	T	C	T	C	T	Accurate
Marker232	Chr. 1	9065390	C	A	C	A	N	Uncertain
Marker233	Chr. 1	9065997	A	A	C	A	C	Accurate
Marker234	Chr. 1	9241290	G	G	T	G	T	Accurate
Marker235	Chr. 1	9247487	C	C	T	C	T	Accurate
Marker236	Chr. 1	9250013	T	T	C	T	C	Accurate
Marker237	Chr. 1	9250014	C	C	T	C	T	Accurate
Marker238	Chr. 1	9275926	A	A	C	A	C	Accurate
Marker239	Chr. 1	9281544	T	T	C	T	C	Accurate
Marker240	Chr. 1	9299109	C	C	T	C	T	Accurate
Marker241	Chr. 1	9299180	T	T	C	T	C	Accurate
Marker242	Chr. 1	9307755	G	G	A	G	A	Accurate

Marker243	Chr. 1	9333210	T	T	C	T	C	Accurate
Marker244	Chr. 1	9348521	G	G	A	G	A	Accurate
Marker245	Chr. 1	9348530	G	G	T	G	T	Accurate
Marker246	Chr. 1	9377375	C	C	T	C	T	Accurate
Marker247	Chr. 1	9377571	A	A	G	A	G	Accurate
Marker248	Chr. 1	9385211	T	T	C	T	C	Accurate
Marker249	Chr. 1	9385538	T	T	G	T	G	Accurate
Marker250	Chr. 1	9391403	C	C	T	C	T	Accurate
Marker251	Chr. 1	9393683	C	C	T	C	T	Accurate
Marker253	Chr. 1	9478717	A	A	C	A	C	Accurate
Marker254	Chr. 1	9481867	T	T	C	T	C	Accurate
Marker259	Chr. 1	10011606	G	G	A	G	A	Accurate
Marker260	Chr. 1	10011631	T	T	C	T	C	Accurate
Marker262	Chr. 1	10234694	C	C	T	C	T	Accurate
Marker263	Chr. 1	10241902	G	G	A	G	A	Accurate
Marker266	Chr. 1	10282453	A	A	G	A	G	Accurate
Marker267	Chr. 1	10302409	T	A	T	A	N	Uncertain
Marker268	Chr. 1	10326634	T	T	A	T	A	Accurate
Marker269	Chr. 1	10342482	A	A	G	A	G	Accurate
Marker270	Chr. 1	10344532	A	A	G	A	G	Accurate
Marker271	Chr. 1	10462247	C	C	A	C	A	Accurate
Marker272	Chr. 1	10462271	T	T	C	T	C	Accurate
Marker274	Chr. 1	10464859	A	A	T	A	T	Accurate
Marker275	Chr. 1	10472715	A	A	G	A	G	Accurate
Marker276	Chr. 1	10530935	T	T	A	T	A	Accurate
Marker279	Chr. 1	10583190	C	A	C	A	C	Accurate
Marker281	Chr. 1	10606482	A	C	A	C	A	Accurate
Marker282	Chr. 1	10666454	T	C	T	C	T	Accurate
Marker283	Chr. 1	10713701	C	T	C	T	C	Accurate
Marker284	Chr. 1	10713997	G	A	G	A	G	Accurate
Marker286	Chr. 1	10737212	G	G	A	G	A	Accurate
Marker287	Chr. 1	10754574	T	T	G	T	G	Accurate
Marker288	Chr. 1	10779973	G	G	A	G	A	Accurate
Marker289	Chr. 1	10779997	C	C	A	C	A	Accurate
Marker290	Chr. 1	10781371	G	A	G	A	G	Accurate
Marker291	Chr. 1	10783745	A	G	A	G	A	Accurate
Marker294	Chr. 1	10810774	G	C	G	C	G	Accurate
Marker297	Chr. 1	10879488	A	T	A	T	A	Accurate
Marker298	Chr. 1	10880023	T	C	T	C	T	Accurate
Marker299	Chr. 1	10964778	G	A	G	A	G	Accurate
Marker300	Chr. 1	10972566	G	C	G	C	G	Accurate
Marker301	Chr. 1	11002012	A	G	A	G	A	Accurate
Marker302	Chr. 1	11006968	C	T	C	T	C	Accurate
Marker304	Chr. 1	11041444	A	G	A	G	A	Accurate
Marker305	Chr. 1	11058284	C	T	C	T	C	Accurate
Marker306	Chr. 1	11079585	T	C	T	C	T	Accurate
Marker307	Chr. 1	11079610	C	G	C	G	C	Accurate

Marker309	Chr. 1	11095339	C	G	C	G	C	Accurate
Marker310	Chr. 1	11102158	T	C	T	C	T	Accurate
Marker313	Chr. 1	11215520	A	T	A	T	A	Accurate
Marker315	Chr. 1	11321799	C	T	C	T	C	Accurate
Marker320	Chr. 1	11492837	C	G	C	G	C	Accurate
Marker321	Chr. 1	11506706	T	C	T	C	T	Accurate
Marker323	Chr. 1	11509858	C	T	C	T	C	Accurate
Marker324	Chr. 1	11513140	T	G	T	G	T	Accurate
Marker325	Chr. 1	11521311	G	A	G	A	G	Accurate
Marker326	Chr. 1	11521347	T	C	T	C	T	Accurate
Marker328	Chr. 1	11539192	T	C	T	C	T	Accurate
Marker331	Chr. 1	11544657	G	A	G	A	G	Accurate
Marker332	Chr. 1	11556833	C	T	C	T	C	Accurate
Marker333	Chr. 1	11717959	G	A	G	A	G	Accurate
Marker334	Chr. 1	11809269	G	A	G	A	G	Accurate
Marker336	Chr. 1	11822909	G	T	G	T	G	Accurate
Marker337	Chr. 1	11823058	G	A	G	A	G	Accurate
Marker338	Chr. 1	11823069	T	C	T	C	T	Accurate
Marker340	Chr. 1	11824606	A	G	A	G	A	Accurate
Marker341	Chr. 1	11828188	T	C	T	C	T	Accurate
Marker342	Chr. 1	11833130	T	C	T	C	T	Accurate
Marker343	Chr. 1	11844071	G	C	G	C	G	Accurate
Marker344	Chr. 1	11844127	T	C	T	C	T	Accurate
Marker345	Chr. 1	11845694	T	C	T	C	T	Accurate
Marker346	Chr. 1	11848399	A	G	A	G	A	Accurate
Marker348	Chr. 1	11865687	T	C	T	C	T	Accurate
Marker349	Chr. 1	11872964	C	G	C	G	C	Accurate
Marker350	Chr. 1	11873611	A	C	A	C	A	Accurate
Marker352	Chr. 1	11877397	C	G	C	G	C	Accurate
Marker354	Chr. 1	11879783	A	G	A	G	A	Accurate
Marker356	Chr. 1	11881184	G	A	G	A	G	Accurate
Marker357	Chr. 1	11905026	G	T	G	T	G	Accurate
Marker358	Chr. 1	11905029	G	A	G	A	G	Accurate
Marker360	Chr. 1	11911638	G	A	G	A	G	Accurate
Marker361	Chr. 1	11911792	A	G	A	G	A	Accurate
Marker362	Chr. 1	11930244	T	G	T	G	T	Accurate
Marker363	Chr. 1	11930276	G	A	G	A	G	Accurate
Marker364	Chr. 1	11933894	T	C	T	C	T	Accurate
Marker366	Chr. 1	11958037	T	C	T	C	T	Accurate
Marker367	Chr. 1	11964375	C	T	C	T	C	Accurate
Marker369	Chr. 1	11980888	A	G	A	G	A	Accurate
Marker371	Chr. 1	11988628	C	T	C	T	C	Accurate
Marker372	Chr. 1	12006255	G	T	G	T	G	Accurate
Marker378	Chr. 1	12082186	C	T	C	T	C	Accurate
Marker381	Chr. 1	12939681	C	T	C	T	C	Accurate
Marker382	Chr. 1	12958706	T	T	G	T	G	Accurate
Marker383	Chr. 1	13005832	T	A	T	A	T	Accurate

Marker384	Chr. 1	13040143	C	T	C	T	C	Accurate
Marker386	Chr. 1	13119226	A	T	A	T	A	Accurate
Marker387	Chr. 1	13121381	A	C	A	C	A	Accurate
Marker389	Chr. 1	13162888	T	C	T	C	T	Accurate
Marker391	Chr. 1	13181219	T	C	T	C	T	Accurate
Marker395	Chr. 1	13367246	T	C	T	C	T	Accurate
Marker397	Chr. 1	13400017	C	G	C	G	C	Accurate
Marker398	Chr. 1	13400330	T	C	T	C	T	Accurate
Marker399	Chr. 1	13401098	A	A	G	A	G	Accurate
Marker401	Chr. 1	13415870	C	T	C	T	C	Accurate
Marker402	Chr. 1	13437969	C	C	T	C	T	Accurate
Marker403	Chr. 1	13438198	C	G	C	G	C	Accurate
Marker404	Chr. 1	13438258	A	G	A	G	A	Accurate
Marker405	Chr. 1	13441657	C	T	C	T	C	Accurate
Marker406	Chr. 1	13443715	C	G	C	G	C	Accurate
Marker407	Chr. 1	13446851	C	G	C	G	C	Accurate
Marker409	Chr. 1	13470441	T	C	T	C	T	Accurate
Marker411	Chr. 1	13474808	G	A	G	A	G	Accurate
Marker414	Chr. 1	13560762	C	C	T	C	T	Accurate
Marker415	Chr. 1	13560806	G	G	A	G	A	Accurate
Marker416	Chr. 1	13609499	C	C	T	C	T	Accurate
Marker417	Chr. 1	13629419	A	C	A	C	A	Accurate
Marker418	Chr. 1	13665188	T	C	T	C	T	Accurate
Marker422	Chr. 1	14187818	G	G	A	G	A	Accurate
Marker423	Chr. 1	14203616	G	G	A	G	A	Accurate
Marker424	Chr. 1	14266918	A	A	G	A	G	Accurate
Marker425	Chr. 1	14267175	A	A	G	A	G	Accurate
Marker426	Chr. 1	14281308	A	A	G	A	G	Accurate
Marker427	Chr. 1	14287000	T	T	C	T	C	Accurate
Marker428	Chr. 1	14287010	A	A	G	A	G	Accurate
Marker429	Chr. 1	14287339	G	G	A	G	A	Accurate
Marker430	Chr. 1	14287515	C	C	T	C	T	Accurate
Marker431	Chr. 1	14295251	T	T	G	T	G	Accurate
Marker432	Chr. 1	14295314	A	A	G	A	G	Accurate
Marker433	Chr. 1	14356298	C	C	T	C	T	Accurate
Marker434	Chr. 1	14356332	T	T	G	T	G	Accurate
Marker435	Chr. 1	14356459	C	C	T	C	T	Accurate
Marker436	Chr. 1	14356514	G	G	A	G	A	Accurate
Marker437	Chr. 1	14367164	A	A	G	A	G	Accurate
Marker438	Chr. 1	14368984	G	G	T	G	T	Accurate
Marker439	Chr. 1	14369060	C	C	T	C	T	Accurate
Marker440	Chr. 1	14373476	A	A	T	A	T	Accurate
Marker441	Chr. 1	14373630	T	T	C	T	C	Accurate
Marker442	Chr. 1	14375026	T	T	C	T	C	Accurate
Marker443	Chr. 1	14375219	A	A	G	A	G	Accurate
Marker444	Chr. 1	14375241	G	G	T	G	T	Accurate
Marker445	Chr. 1	14391567	A	C	A	C	A	Accurate

Marker446	Chr. 1	14391596	T	T	C	T	C	Accurate
Marker447	Chr. 1	14392439	T	T	C	T	C	Accurate
Marker448	Chr. 1	14392507	C	C	A	C	A	Accurate
Marker449	Chr. 1	14392516	T	T	G	T	G	Accurate
Marker450	Chr. 1	14402168	C	C	T	C	T	Accurate
Marker451	Chr. 1	14408598	T	T	C	T	C	Accurate
Marker452	Chr. 1	14408599	G	G	T	G	T	Accurate
Marker454	Chr. 1	14418711	T	T	G	T	G	Accurate
Marker455	Chr. 1	14430550	T	T	C	T	C	Accurate
Marker456	Chr. 1	14430800	C	C	T	C	T	Accurate
Marker457	Chr. 1	14432518	A	A	T	A	T	Accurate
Marker458	Chr. 1	14432526	C	C	T	C	T	Accurate
Marker459	Chr. 1	14435819	G	G	T	G	T	Accurate
Marker460	Chr. 1	14435876	T	T	C	T	C	Accurate
Marker461	Chr. 1	14439809	T	T	C	T	C	Accurate
Marker462	Chr. 1	14452363	A	A	C	A	C	Accurate
Marker463	Chr. 1	14462108	A	A	G	A	G	Accurate
Marker464	Chr. 1	14462528	G	G	A	G	A	Accurate
Marker465	Chr. 1	14489831	A	A	G	A	G	Accurate
Marker466	Chr. 1	14489833	G	G	C	G	C	Accurate
Marker467	Chr. 1	14489969	A	A	G	A	G	Accurate
Marker468	Chr. 1	14540592	C	C	T	C	T	Accurate
Marker469	Chr. 1	14540875	T	T	C	T	C	Accurate
Marker470	Chr. 1	14546394	C	C	T	C	T	Accurate
Marker471	Chr. 1	14546423	T	T	A	N	A	Uncertain
Marker472	Chr. 1	15335413	C	T	C	T	C	Accurate
Marker473	Chr. 1	15532764	C	T	C	T	C	Accurate
Marker475	Chr. 1	15649185	C	C	T	C	T	Accurate
Marker476	Chr. 1	15783043	A	G	A	G	A	Accurate
Marker477	Chr. 1	15787811	T	C	T	C	T	Accurate
Marker478	Chr. 1	15795675	A	C	A	C	A	Accurate
Marker479	Chr. 1	15803325	C	T	C	T	C	Accurate
Marker480	Chr. 1	15840808	C	G	C	G	C	Accurate
Marker481	Chr. 1	15842725	T	C	T	C	T	Accurate
Marker482	Chr. 1	15874469	G	A	G	A	G	Accurate
Marker483	Chr. 1	15974403	T	C	T	C	T	Accurate
Marker485	Chr. 1	16853774	G	A	G	A	G	Accurate
Marker488	Chr. 1	17169513	G	G	A	G	A	Accurate
Marker489	Chr. 1	17170090	T	T	C	T	C	Accurate
Marker490	Chr. 1	17187193	C	C	T	C	T	Accurate
Marker491	Chr. 1	17206057	A	G	A	G	A	Accurate
Marker492	Chr. 1	17240508	C	A	C	A	C	Accurate
Marker493	Chr. 1	17240764	T	C	T	C	T	Accurate
Marker494	Chr. 1	17251267	T	A	T	A	T	Accurate
Marker496	Chr. 1	17277120	A	A	G	A	G	Accurate
Marker497	Chr. 1	17298639	T	T	C	T	C	Accurate
Marker498	Chr. 1	17302910	C	C	T	C	T	Accurate

Marker499	Chr. 1	17303421	T	T	C	T	C	Accurate
Marker500	Chr. 1	17324977	A	A	G	A	G	Accurate
Marker501	Chr. 1	17341017	C	C	T	C	T	Accurate
Marker502	Chr. 1	17355898	A	A	G	A	G	Accurate
Marker503	Chr. 1	17358957	C	C	T	C	T	Accurate
Marker504	Chr. 1	17358959	G	G	A	G	A	Accurate
Marker505	Chr. 1	17361724	C	C	G	C	G	Accurate
Marker506	Chr. 1	17369345	C	C	T	C	T	Accurate
Marker507	Chr. 1	17372065	T	T	C	T	C	Accurate
Marker508	Chr. 1	17376508	C	C	G	C	G	Accurate
Marker509	Chr. 1	17378677	T	T	C	T	C	Accurate
Marker510	Chr. 1	17379020	G	G	A	G	A	Accurate
Marker511	Chr. 1	17384216	A	A	G	A	G	Accurate
Marker512	Chr. 1	17399773	A	A	G	A	G	Accurate
Marker513	Chr. 1	17411931	C	C	T	C	T	Accurate
Marker514	Chr. 1	17413422	T	T	C	T	C	Accurate
Marker515	Chr. 1	17413488	A	A	G	A	G	Accurate
Marker516	Chr. 1	17413705	A	A	G	A	G	Accurate
Marker517	Chr. 1	17434476	T	T	A	T	A	Accurate
Marker518	Chr. 1	17434477	T	T	G	T	G	Accurate
Marker519	Chr. 1	17434478	C	C	A	C	A	Accurate
Marker520	Chr. 1	17452458	G	G	A	G	A	Accurate
Marker521	Chr. 1	17456695	C	C	T	C	T	Accurate
Marker522	Chr. 1	17477991	A	A	C	A	C	Accurate
Marker523	Chr. 1	17497982	A	G	A	G	A	Accurate
Marker525	Chr. 1	17538521	G	A	G	A	G	Accurate
Marker527	Chr. 1	17547485	A	G	A	G	A	Accurate
Marker528	Chr. 1	17562799	T	C	T	C	T	Accurate
Marker534	Chr. 1	17633269	T	C	T	C	T	Accurate
Marker535	Chr. 1	17649639	G	A	G	A	G	Accurate
Marker536	Chr. 1	17649657	T	C	T	C	T	Accurate
Marker538	Chr. 1	17683298	A	G	A	G	A	Accurate
Marker539	Chr. 1	17691801	T	C	T	C	T	Accurate
Marker540	Chr. 1	17697574	T	A	T	A	T	Accurate
Marker541	Chr. 1	17703034	T	T	C	T	C	Accurate
Marker542	Chr. 1	17953188	C	C	G	C	G	Accurate
Marker543	Chr. 1	18072690	G	A	G	A	G	Accurate
Marker544	Chr. 1	18090307	G	G	T	G	T	Accurate
Marker545	Chr. 1	18093546	A	C	A	C	A	Accurate
Marker546	Chr. 1	18141866	T	T	C	T	C	Accurate
Marker547	Chr. 1	18145296	T	T	C	T	C	Accurate
Marker548	Chr. 1	18200302	C	C	T	C	T	Accurate
Marker549	Chr. 1	18200502	A	A	G	A	G	Accurate
Marker550	Chr. 1	18200819	A	A	G	A	G	Accurate
Marker551	Chr. 1	18209006	C	C	T	C	T	Accurate
Marker553	Chr. 1	18233725	G	G	A	G	A	Accurate
Marker555	Chr. 1	18255386	C	C	A	C	A	Accurate

Marker556	Chr. 1	18255772	A	A	G	A	G	Accurate
Marker557	Chr. 1	18255967	A	A	G	N	G	Uncertain
Marker559	Chr. 1	18276051	T	C	T	C	T	Accurate
Marker560	Chr. 1	18336055	C	T	C	T	C	Accurate
Marker562	Chr. 1	18369986	C	T	C	T	C	Accurate
Marker563	Chr. 1	18497321	C	C	T	C	T	Accurate
Marker564	Chr. 1	18599419	A	G	A	G	A	Accurate
Marker565	Chr. 1	18603641	T	A	T	A	T	Accurate
Marker566	Chr. 1	18603837	G	A	G	A	G	Accurate
Marker568	Chr. 1	18626174	T	T	A	T	A	Accurate
Marker569	Chr. 1	18630793	C	C	G	N	G	Uncertain
Marker570	Chr. 1	18630900	G	G	A	G	A	Accurate
Marker571	Chr. 1	18660917	T	T	C	N	C	Uncertain
Marker572	Chr. 1	18660951	A	A	C	N	C	Uncertain
Marker573	Chr. 1	18661164	G	G	T	G	T	Accurate
Marker574	Chr. 1	18690861	G	G	A	G	A	Accurate
Marker575	Chr. 1	18699745	C	C	G	C	G	Accurate
Marker577	Chr. 1	18830662	G	G	A	G	A	Accurate
Marker578	Chr. 1	18860754	G	G	A	G	A	Accurate
Marker579	Chr. 1	18903629	G	G	A	G	A	Accurate
Marker585	Chr. 1	19761216	A	T	A	T	A	Accurate
Marker586	Chr. 1	19761377	T	C	T	C	T	Accurate
Marker588	Chr. 1	20142159	T	T	C	T	C	Accurate
Marker589	Chr. 1	20169658	G	G	A	G	A	Accurate
Marker590	Chr. 1	20185915	C	C	T	C	T	Accurate
Marker592	Chr. 1	20218960	C	C	T	C	T	Accurate
Marker593	Chr. 1	20255677	C	C	T	C	T	Accurate
Marker594	Chr. 1	20258735	T	T	C	T	C	Accurate
Marker595	Chr. 1	20303548	G	G	A	G	A	Accurate
Marker596	Chr. 1	20312467	A	A	G	A	G	Accurate
Marker597	Chr. 1	20330355	A	A	T	A	T	Accurate
Marker598	Chr. 1	20433408	G	G	A	R	A	Uncertain
Marker599	Chr. 1	20455392	C	C	T	C	T	Accurate
Marker600	Chr. 1	20471325	A	A	G	A	G	Accurate
Marker601	Chr. 1	20487608	A	A	C	A	C	Accurate
Marker602	Chr. 1	20511607	T	T	C	T	C	Accurate
Marker603	Chr. 1	20751870	A	A	G	A	G	Accurate
Marker606	Chr. 1	20785639	A	A	G	A	G	Accurate
Marker607	Chr. 1	20795997	T	T	C	T	C	Accurate
Marker608	Chr. 1	20796199	G	G	A	G	A	Accurate
Marker609	Chr. 1	20797434	C	C	T	C	T	Accurate
Marker610	Chr. 1	20797435	T	T	A	T	A	Accurate
Marker611	Chr. 1	20815145	C	C	T	C	T	Accurate
Marker612	Chr. 1	20829908	G	A	G	A	G	Accurate
Marker613	Chr. 1	21166518	T	T	C	T	C	Accurate
Marker615	Chr. 1	21316533	T	A	T	A	T	Accurate
Marker616	Chr. 1	21316696	G	G	C	G	C	Accurate

Marker619	Chr. 1	21413290	G	A	G	A	G	Accurate
Marker621	Chr. 1	21415629	A	T	A	T	A	Accurate
Marker622	Chr. 1	21415798	A	G	A	G	A	Accurate
Marker623	Chr. 1	21415805	A	G	A	G	A	Accurate
Marker624	Chr. 1	21415843	C	T	C	T	C	Accurate
Marker625	Chr. 1	21435904	A	T	A	T	A	Accurate
Marker627	Chr. 1	21456831	C	T	C	T	C	Accurate
Marker628	Chr. 1	21461708	T	A	T	A	T	Accurate
Marker631	Chr. 1	21492527	A	G	A	G	A	Accurate
Marker632	Chr. 1	21527784	C	T	C	T	C	Accurate
Marker633	Chr. 1	21569421	A	G	A	G	A	Accurate
Marker635	Chr. 1	21581166	A	C	A	C	A	Accurate
Marker636	Chr. 1	21603891	A	G	A	G	A	Accurate
Marker637	Chr. 1	21610780	G	T	G	T	G	Accurate
Marker638	Chr. 1	21659672	C	T	C	T	C	Accurate
Marker641	Chr. 1	21835983	G	A	G	A	G	Accurate
Marker642	Chr. 1	21874271	T	T	C	T	C	Accurate
Marker643	Chr. 1	21880788	T	T	A	T	A	Accurate
Marker646	Chr. 1	21931579	A	A	G	A	G	Accurate
Marker648	Chr. 1	21944541	G	G	A	G	A	Accurate
Marker649	Chr. 1	21944559	A	A	G	A	G	Accurate
Marker652	Chr. 1	21956731	C	C	T	C	T	Accurate
Marker654	Chr. 1	22013275	T	T	C	T	C	Accurate
Marker655	Chr. 1	22238803	A	A	G	A	G	Accurate
Marker661	Chr. 1	22287799	T	T	C	T	C	Accurate
Marker665	Chr. 1	22323886	C	C	T	C	T	Accurate
Marker666	Chr. 1	22325497	C	C	T	C	T	Accurate
Marker667	Chr. 1	22384211	T	T	A	T	A	Accurate
Marker671	Chr. 1	22401919	T	T	C	T	C	Accurate
Marker673	Chr. 1	22419033	C	C	T	C	T	Accurate
Marker677	Chr. 1	22514635	C	C	T	C	T	Accurate
Marker681	Chr. 1	22624565	G	G	T	G	T	Accurate
Marker682	Chr. 1	22625301	C	C	T	C	T	Accurate
Marker683	Chr. 1	22625350	C	C	T	C	Y	Uncertain
Marker686	Chr. 1	22667811	G	G	A	G	A	Accurate
Marker688	Chr. 1	22682231	G	G	A	G	A	Accurate
Marker696	Chr. 1	22918333	A	A	G	A	G	Accurate
Marker698	Chr. 1	23066098	G	A	G	A	G	Accurate
Marker701	Chr. 1	23697248	T	A	T	A	T	Accurate
Marker702	Chr. 1	23697259	A	G	A	G	A	Accurate
Marker704	Chr. 1	23725565	A	G	A	G	A	Accurate
Marker706	Chr. 1	23753907	T	C	T	C	T	Accurate
Marker707	Chr. 1	23756495	T	C	T	C	T	Accurate
Marker709	Chr. 1	24377466	C	C	T	C	T	Accurate
Marker710	Chr. 1	24380575	T	T	C	T	C	Accurate
Marker711	Chr. 1	24407488	C	C	A	C	A	Accurate
Marker714	Chr. 1	24480450	G	G	T	G	T	Accurate

Marker715	Chr. 1	24482849	G	G	A	G	A	Accurate
Marker716	Chr. 1	24527564	T	T	C	T	C	Accurate
Marker717	Chr. 1	24579328	T	C	T	W	A	Inconsistent
Marker718	Chr. 1	25179542	A	A	G	A	G	Accurate
Marker719	Chr. 1	25182274	T	T	C	T	C	Accurate
Marker722	Chr. 1	25206069	T	C	T	C	T	Accurate
Marker728	Chr. 1	25507482	G	A	G	A	G	Accurate
Marker729	Chr. 1	25534746	G	A	G	A	G	Accurate
Marker731	Chr. 1	25551105	G	T	G	T	G	Accurate
Marker735	Chr. 1	25858874	A	G	A	G	A	Accurate
Marker738	Chr. 1	25906231	T	C	T	C	T	Accurate
Marker742	Chr. 1	26000327	C	G	C	G	C	Accurate
Marker743	Chr. 1	26132724	G	G	A	G	A	Accurate
Marker745	Chr. 1	26175838	C	A	C	A	C	Accurate
Marker748	Chr. 1	26436594	C	T	C	T	C	Accurate
Marker749	Chr. 1	26438194	G	A	G	A	G	Accurate
Marker751	Chr. 1	26451766	C	T	C	T	C	Accurate
Marker752	Chr. 1	26453160	T	C	T	C	T	Accurate
Marker753	Chr. 1	26458820	A	T	A	T	A	Accurate
Marker754	Chr. 1	26481944	T	C	T	C	T	Accurate
Marker755	Chr. 1	26580553	A	G	A	G	A	Accurate
Marker756	Chr. 1	26584242	T	G	T	G	T	Accurate
Marker757	Chr. 1	26601076	A	G	A	G	A	Accurate
Marker759	Chr. 1	26605053	G	A	G	A	G	Accurate
Marker760	Chr. 1	26605056	C	T	C	T	C	Accurate
Marker764	Chr. 1	26917447	G	A	G	A	G	Accurate
Marker765	Chr. 1	26936774	G	C	G	C	G	Accurate
Marker766	Chr. 1	27103499	C	A	C	A	C	Accurate
Marker769	Chr. 1	27197556	G	A	G	A	G	Accurate
Marker771	Chr. 1	27313856	T	C	T	C	T	Accurate
Marker772	Chr. 1	27326199	C	T	C	T	C	Accurate
Marker773	Chr. 1	27326201	A	G	A	G	A	Accurate
Marker774	Chr. 1	27326221	C	T	C	T	C	Accurate
Marker775	Chr. 1	27341337	C	T	C	T	C	Accurate
Marker776	Chr. 1	27351688	G	T	G	T	G	Accurate
Marker777	Chr. 1	27377176	G	A	G	A	G	Accurate
Marker778	Chr. 1	27393376	G	A	G	A	G	Accurate
Marker779	Chr. 1	27460357	T	T	C	T	C	Accurate
Marker781	Chr. 1	27478265	C	C	T	C	T	Accurate
Marker782	Chr. 1	27487870	T	T	C	T	C	Accurate
Marker783	Chr. 1	27489613	A	A	G	A	G	Accurate
Marker784	Chr. 1	27915817	C	A	C	A	C	Accurate
Marker785	Chr. 1	27933300	A	G	A	G	A	Accurate
Marker787	Chr. 1	28045285	T	T	G	T	G	Accurate
Marker788	Chr. 1	28046805	C	C	A	C	A	Accurate
Marker789	Chr. 1	28056605	T	T	C	T	C	Accurate
Marker790	Chr. 1	28140156	C	C	A	C	A	Accurate

Marker791	Chr. 1	28152601	G	G	T	N	T	Uncertain
Marker792	Chr. 1	28194835	G	G	A	G	A	Accurate
Marker793	Chr. 1	28211362	G	G	A	G	A	Accurate
Marker794	Chr. 1	28223665	C	C	T	C	T	Accurate
Marker795	Chr. 1	28255801	A	A	T	A	T	Accurate
Marker797	Chr. 1	28260153	T	T	C	T	C	Accurate
Marker798	Chr. 1	28266618	A	A	T	A	T	Accurate
Marker799	Chr. 1	28385267	T	A	T	A	T	Accurate
Marker800	Chr. 1	28391992	A	G	A	G	A	Accurate
Marker801	Chr. 1	28405652	G	A	G	A	G	Accurate
Marker802	Chr. 1	28411151	A	T	A	T	A	Accurate
Marker803	Chr. 1	28446252	C	T	C	T	C	Accurate
Marker804	Chr. 1	28486694	C	T	C	T	C	Accurate
Marker805	Chr. 1	28510941	A	G	A	G	A	Accurate
Marker806	Chr. 1	28546201	T	C	T	C	T	Accurate
Marker807	Chr. 1	28547295	G	A	G	A	G	Accurate
Marker808	Chr. 1	28557624	T	C	T	C	T	Accurate
Marker809	Chr. 1	28558063	G	A	G	A	G	Accurate
Marker811	Chr. 1	28632245	A	G	A	G	N	Uncertain
Marker812	Chr. 1	28650122	A	T	A	T	A	Accurate
Marker813	Chr. 1	28710663	T	A	T	A	T	Accurate
Marker814	Chr. 1	28782910	T	C	T	C	T	Accurate
Marker815	Chr. 1	28782960	A	G	A	G	A	Accurate
Marker817	Chr. 1	28863676	T	C	T	C	T	Accurate
Marker818	Chr. 1	28912703	G	T	G	T	G	Accurate
Marker819	Chr. 1	28913101	G	T	G	T	G	Accurate
Marker820	Chr. 1	28913270	T	A	T	A	T	Accurate
Marker821	Chr. 1	28927605	A	G	A	G	A	Accurate
Marker822	Chr. 1	28939444	T	C	T	C	T	Accurate
Marker823	Chr. 1	28946272	A	A	G	A	G	Accurate
Marker825	Chr. 1	29162239	C	C	T	C	T	Accurate
Marker826	Chr. 1	29162526	T	T	A	T	A	Accurate
Marker827	Chr. 1	29270860	C	T	C	T	C	Accurate
Marker828	Chr. 1	29271104	C	A	C	A	C	Accurate
Marker829	Chr. 1	29567941	T	T	C	T	C	Accurate
Marker832	Chr. 1	29685065	A	G	A	G	A	Accurate
Marker833	Chr. 1	29697484	T	C	T	C	T	Accurate
Marker834	Chr. 1	29697506	A	T	A	T	A	Accurate
Marker835	Chr. 1	29710628	G	A	G	A	G	Accurate
Marker837	Chr. 1	29725425	T	C	T	C	T	Accurate
Marker838	Chr. 1	29747082	A	G	A	G	A	Accurate
Marker839	Chr. 1	29778850	T	T	G	T	G	Accurate
Marker840	Chr. 1	29781747	G	G	T	G	T	Accurate
Marker841	Chr. 1	29795334	G	G	A	G	A	Accurate
Marker842	Chr. 1	29893249	C	C	T	C	T	Accurate
Marker845	Chr. 1	29926101	C	A	C	A	C	Accurate
Marker846	Chr. 1	29926847	C	T	C	T	C	Accurate

Marker847	Chr. 1	29927032	A	G	A	G	A	Accurate
Marker850	Chr. 1	30054955	C	G	C	G	C	Accurate
Marker851	Chr. 1	30065178	G	T	G	T	G	Accurate
Marker852	Chr. 1	30094278	C	T	C	T	C	Accurate
Marker853	Chr. 1	30127359	G	T	G	T	G	Accurate
Marker854	Chr. 1	30131377	A	G	A	G	A	Accurate
Marker855	Chr. 1	30131386	C	T	C	T	C	Accurate
Marker856	Chr. 1	30276646	G	C	G	C	G	Accurate
Marker858	Chr. 1	30392193	A	C	A	C	A	Accurate
Marker859	Chr. 1	30434949	T	C	T	C	T	Accurate
Marker861	Chr. 1	30486350	A	T	A	T	A	Accurate
Marker862	Chr. 1	30517412	G	A	G	A	G	Accurate
Marker863	Chr. 1	30555414	T	C	T	C	T	Accurate
Marker864	Chr. 1	30614229	G	A	G	A	G	Accurate
Marker865	Chr. 1	30641822	T	A	T	A	T	Accurate
Marker866	Chr. 1	30643296	T	C	T	C	T	Accurate
Marker867	Chr. 1	30660776	G	A	G	A	G	Accurate
Marker868	Chr. 1	30666726	T	C	T	C	T	Accurate
Marker869	Chr. 1	30671277	A	G	A	G	A	Accurate
Marker870	Chr. 1	30673954	A	G	A	G	A	Accurate
Marker871	Chr. 1	30674313	A	G	A	G	A	Accurate
Marker872	Chr. 1	30725086	C	T	C	T	C	Accurate
Marker873	Chr. 1	30748004	T	A	T	A	T	Accurate
Marker874	Chr. 1	30755505	T	A	T	A	T	Accurate
Marker875	Chr. 1	30775945	T	C	T	C	T	Accurate
Marker876	Chr. 1	30815593	T	A	T	A	T	Accurate
Marker878	Chr. 1	30893759	A	C	A	C	A	Accurate
Marker879	Chr. 1	30920555	C	C	A	C	A	Accurate
Marker880	Chr. 1	30932809	G	A	G	A	G	Accurate
Marker881	Chr. 1	30959533	G	A	G	A	G	Accurate
Marker882	Chr. 1	30977218	T	C	T	C	T	Accurate
Marker883	Chr. 1	31013103	A	G	A	G	N	Uncertain
Marker884	Chr. 1	31013112	T	C	T	C	T	Accurate
Marker885	Chr. 1	31038634	G	T	G	T	N	Uncertain
Marker886	Chr. 1	31046836	G	A	G	A	G	Accurate
Marker887	Chr. 1	31127158	A	G	A	G	A	Accurate
Marker888	Chr. 1	31178159	G	A	G	A	G	Accurate
Marker889	Chr. 1	31262463	C	C	T	C	T	Accurate
Marker890	Chr. 1	31262710	G	G	C	G	C	Accurate
Marker891	Chr. 1	31281799	C	C	T	C	T	Accurate
Marker892	Chr. 1	31294585	T	T	C	T	C	Accurate
Marker893	Chr. 1	31305827	A	A	G	A	G	Accurate
Marker894	Chr. 1	31325596	C	C	A	C	A	Accurate
Marker895	Chr. 1	31474092	A	G	A	G	A	Accurate
Marker896	Chr. 1	31474343	C	T	C	T	C	Accurate
Marker897	Chr. 1	31474485	G	A	G	A	G	Accurate
Marker898	Chr. 1	31479797	T	C	T	C	T	Accurate

Marker899	Chr. 1	31485637	G	A	G	A	G	Accurate
Marker900	Chr. 1	31503055	G	T	G	T	G	Accurate
Marker901	Chr. 1	31507152	A	C	A	C	A	Accurate
Marker902	Chr. 1	31620783	A	G	A	G	A	Accurate
Marker903	Chr. 1	31730739	C	G	C	G	C	Accurate
Marker904	Chr. 1	31783099	G	G	C	G	C	Accurate
Marker905	Chr. 1	31784399	T	T	C	T	C	Accurate
Marker906	Chr. 1	31804131	A	G	A	G	A	Accurate
Marker907	Chr. 1	31885667	T	C	T	C	T	Accurate
Marker908	Chr. 1	31887334	C	G	C	G	C	Accurate
Marker909	Chr. 1	31891539	A	G	A	G	A	Accurate
Marker910	Chr. 1	31924120	T	G	T	G	T	Accurate
Marker911	Chr. 1	31924321	A	C	A	C	A	Accurate
Marker912	Chr. 1	31965644	A	A	G	A	G	Accurate
Marker913	Chr. 1	31984460	G	G	T	G	T	Accurate
Marker914	Chr. 1	31984851	A	A	G	A	G	Accurate
Marker915	Chr. 1	31984960	A	A	C	A	C	Accurate
Marker916	Chr. 1	31984962	C	C	A	C	A	Accurate
Marker917	Chr. 1	31989763	A	A	C	A	C	Accurate
Marker918	Chr. 1	31996562	G	G	T	G	T	Accurate
Marker919	Chr. 1	31996585	C	C	T	C	T	Accurate
Marker920	Chr. 1	32007833	G	G	A	G	A	Accurate
Marker921	Chr. 1	32023536	C	C	A	C	A	Accurate
Marker922	Chr. 1	32039992	G	G	A	G	A	Accurate
Marker923	Chr. 1	32040021	C	C	T	C	T	Accurate
Marker925	Chr. 1	32205728	T	T	G	T	G	Accurate
Marker926	Chr. 1	32236823	C	C	T	C	T	Accurate
Marker927	Chr. 1	32236850	G	G	A	G	A	Accurate
Marker931	Chr. 1	32392223	G	G	A	G	A	Accurate
Marker932	Chr. 1	32479727	A	A	G	A	G	Accurate
Marker933	Chr. 1	32531231	A	A	T	A	T	Accurate
Marker934	Chr. 1	32541031	T	T	A	T	A	Accurate
Marker935	Chr. 1	32565735	A	A	G	A	G	Accurate
Marker936	Chr. 1	32565933	C	C	T	C	T	Accurate
Marker937	Chr. 1	32632147	G	G	A	G	A	Accurate
Marker938	Chr. 1	32632457	A	A	G	A	G	Accurate
Marker939	Chr. 1	32640288	T	T	A	N	A	Uncertain
Marker940	Chr. 1	32647880	A	T	A	T	A	Accurate
Marker941	Chr. 1	32647896	A	C	A	C	A	Accurate
Marker942	Chr. 1	32648053	T	A	T	A	T	Accurate
Marker943	Chr. 1	32678935	A	T	A	T	A	Accurate
Marker944	Chr. 1	32681537	A	A	G	A	G	Accurate
Marker945	Chr. 1	32681850	A	A	T	A	T	Accurate
Marker946	Chr. 1	32691937	A	A	G	A	G	Accurate
Marker947	Chr. 1	32697712	A	A	C	A	C	Accurate
Marker948	Chr. 1	32709637	T	G	T	G	T	Accurate
Marker950	Chr. 1	32714135	T	T	G	T	G	Accurate

Marker951	Chr. 1	33034298	A	A	T	N	T	Uncertain
Marker952	Chr. 1	33035045	A	A	C	N	C	Uncertain
Marker953	Chr. 1	33035068	G	G	A	G	A	Accurate
Marker954	Chr. 1	33038266	A	A	G	A	G	Accurate
Marker955	Chr. 1	33038429	T	T	C	T	C	Accurate
Marker956	Chr. 1	33043967	C	T	C	T	C	Accurate
Marker957	Chr. 1	33133048	G	A	G	A	G	Accurate
Marker958	Chr. 1	33133069	G	A	G	A	G	Accurate
Marker959	Chr. 1	33138274	T	C	T	C	T	Accurate
Marker960	Chr. 1	33146647	T	C	T	C	T	Accurate
Marker961	Chr. 1	33154356	C	T	C	T	C	Accurate
Marker964	Chr. 1	33309184	T	T	A	T	A	Accurate
Marker965	Chr. 1	33347782	C	C	A	C	A	Accurate
Marker966	Chr. 1	33409635	C	A	C	A	C	Accurate
Marker967	Chr. 1	33426423	C	A	C	A	C	Accurate
Marker968	Chr. 1	33440217	T	C	T	C	T	Accurate
Marker969	Chr. 1	33451705	G	A	G	A	G	Accurate
Marker970	Chr. 1	33453755	G	T	G	T	G	Accurate
Marker971	Chr. 1	33574523	C	T	C	T	C	Accurate
Marker972	Chr. 1	33678304	A	A	T	A	T	Accurate
Marker973	Chr. 1	33678502	T	T	C	T	C	Accurate
Marker974	Chr. 1	33700288	A	A	T	A	T	Accurate
Marker976	Chr. 1	33734855	A	C	A	C	N	Uncertain
Marker977	Chr. 1	33736146	G	A	G	A	G	Accurate
Marker978	Chr. 1	33747773	G	T	G	T	G	Accurate
Marker979	Chr. 1	33775660	G	G	C	G	C	Accurate
Marker981	Chr. 1	33775687	A	A	T	A	T	Accurate
Marker987	Chr. 2	57329	C	C	G	C	G	Accurate
Marker990	Chr. 2	80814	G	G	A	G	A	Accurate
Marker992	Chr. 2	83862	A	A	T	A	W	Uncertain
Marker995	Chr. 2	122251	C	C	T	C	T	Accurate
Marker1000	Chr. 2	321378	T	T	G	T	G	Accurate
Marker1001	Chr. 2	321547	G	G	A	N	A	Uncertain
Marker1007	Chr. 2	399211	C	T	C	T	C	Accurate
Marker1012	Chr. 2	456887	C	T	C	T	C	Accurate
Marker1013	Chr. 2	462763	G	A	G	A	G	Accurate
Marker1018	Chr. 2	667918	G	T	G	T	G	Accurate
Marker1019	Chr. 2	673553	G	T	G	T	G	Accurate
Marker1021	Chr. 2	779278	T	C	T	C	T	Accurate
Marker1023	Chr. 2	879956	C	C	A	C	A	Accurate
Marker1024	Chr. 2	952777	G	G	A	G	A	Accurate
Marker1025	Chr. 2	959390	C	C	G	C	G	Accurate
Marker1029	Chr. 2	1037527	T	T	A	T	A	Accurate
Marker1035	Chr. 2	2145399	G	G	A	G	A	Accurate
Marker1037	Chr. 2	2160916	G	G	A	G	A	Accurate
Marker1041	Chr. 2	2379837	C	G	C	G	C	Accurate
Marker1043	Chr. 2	2423974	G	G	T	G	T	Accurate

Marker1047	Chr. 2	2628782	C	C	A	C	A	Accurate
Marker1056	Chr. 2	3312820	C	C	G	C	G	Accurate
Marker1058	Chr. 2	3316155	G	G	A	G	A	Accurate
Marker1060	Chr. 2	3378523	G	G	A	G	A	Accurate
Marker1063	Chr. 2	4599998	C	C	G	C	G	Accurate
Marker1064	Chr. 2	4717691	G	G	T	G	T	Accurate
Marker1065	Chr. 2	4717940	C	C	T	C	T	Accurate
Marker1066	Chr. 2	4748660	A	A	G	A	G	Accurate
Marker1067	Chr. 2	4748837	T	T	C	T	C	Accurate
Marker1068	Chr. 2	4782861	G	G	A	G	A	Accurate
Marker1069	Chr. 2	4782862	A	A	G	A	G	Accurate
Marker1070	Chr. 2	4783064	T	T	A	T	A	Accurate
Marker1072	Chr. 2	4858873	T	T	C	T	C	Accurate
Marker1073	Chr. 2	4859022	G	G	A	G	A	Accurate
Marker1074	Chr. 2	4870349	C	C	G	C	G	Accurate
Marker1077	Chr. 2	5345338	A	A	G	A	G	Accurate
Marker1080	Chr. 2	5611569	G	G	T	G	T	Accurate
Marker1081	Chr. 2	5612552	A	A	G	A	G	Accurate
Marker1082	Chr. 2	5612565	T	T	G	T	G	Accurate
Marker1083	Chr. 2	5615613	C	C	G	C	G	Accurate
Marker1084	Chr. 2	5725800	C	C	T	C	T	Accurate
Marker1085	Chr. 2	5737730	T	T	C	T	C	Accurate
Marker1088	Chr. 2	5813788	A	T	A	T	A	Accurate
Marker1089	Chr. 2	6014634	G	G	A	G	A	Accurate
Marker1091	Chr. 2	6063168	A	T	A	T	A	Accurate
Marker1095	Chr. 2	6243475	T	T	C	T	C	Accurate
Marker1096	Chr. 2	6243489	A	A	G	A	G	Accurate
Marker1097	Chr. 2	7445498	C	C	A	C	A	Accurate
Marker1099	Chr. 2	7498945	A	A	C	A	C	Accurate
Marker1104	Chr. 2	7878192	A	G	A	G	A	Accurate
Marker1109	Chr. 2	8812829	A	C	A	C	A	Accurate
Marker1110	Chr. 2	8828081	T	A	T	A	T	Accurate
Marker1111	Chr. 2	8857408	T	C	T	C	T	Accurate
Marker1112	Chr. 2	8857527	G	A	G	A	G	Accurate
Marker1113	Chr. 2	8868175	A	G	A	G	A	Accurate
Marker1118	Chr. 2	10027789	T	C	T	C	T	Accurate
Marker1123	Chr. 2	10522396	C	T	C	T	C	Accurate
Marker1124	Chr. 2	10522413	T	C	T	C	T	Accurate
Marker1125	Chr. 2	10654525	T	T	C	T	C	Accurate
Marker1128	Chr. 2	10669989	C	C	T	C	T	Accurate
Marker1129	Chr. 2	10684835	G	G	T	G	T	Accurate
Marker1130	Chr. 2	10724053	A	T	A	T	A	Accurate
Marker1134	Chr. 2	10911344	A	A	G	A	G	Accurate
Marker1137	Chr. 2	10934572	A	A	G	A	G	Accurate
Marker1138	Chr. 2	10934591	T	T	C	T	C	Accurate
Marker1139	Chr. 2	10934633	T	T	C	T	C	Accurate
Marker1140	Chr. 2	10935427	T	T	C	T	C	Accurate

Marker1141	Chr. 2	10935484	A	A	G	A	G	Accurate
Marker1142	Chr. 2	10951970	T	T	C	T	C	Accurate
Marker1145	Chr. 2	10975616	A	A	G	A	G	Accurate
Marker1146	Chr. 2	10978254	T	T	C	T	C	Accurate
Marker1147	Chr. 2	10980612	G	G	T	G	T	Accurate
Marker1148	Chr. 2	10983943	C	C	A	C	A	Accurate
Marker1150	Chr. 2	11009283	T	T	G	T	G	Accurate
Marker1151	Chr. 2	11020006	C	C	T	C	T	Accurate
Marker1153	Chr. 2	11073172	G	G	C	G	C	Accurate
Marker1155	Chr. 2	11083955	A	A	G	A	G	Accurate
Marker1156	Chr. 2	11084078	C	C	T	C	T	Accurate
Marker1157	Chr. 2	11084122	G	G	A	G	A	Accurate
Marker1158	Chr. 2	11096526	T	T	C	T	C	Accurate
Marker1159	Chr. 2	11096705	T	T	G	T	G	Accurate
Marker1161	Chr. 2	11103341	G	G	A	G	A	Accurate
Marker1162	Chr. 2	11103537	T	T	C	T	C	Accurate
Marker1164	Chr. 2	11109876	A	A	G	A	G	Accurate
Marker1165	Chr. 2	11110136	C	C	T	C	T	Accurate
Marker1166	Chr. 2	11110193	C	C	T	C	T	Accurate
Marker1167	Chr. 2	11110537	A	A	C	A	C	Accurate
Marker1168	Chr. 2	11120282	G	G	T	G	T	Accurate
Marker1176	Chr. 2	11162532	A	A	T	A	T	Accurate
Marker1180	Chr. 2	11184330	G	G	A	G	A	Accurate
Marker1182	Chr. 2	11194256	A	A	G	A	G	Accurate
Marker1183	Chr. 2	11194292	T	T	A	T	A	Accurate
Marker1184	Chr. 2	11217213	C	C	T	C	T	Accurate
Marker1185	Chr. 2	11221425	T	T	C	T	C	Accurate
Marker1189	Chr. 2	11239453	C	C	A	C	A	Accurate
Marker1190	Chr. 2	11337621	A	G	A	G	A	Accurate
Marker1194	Chr. 2	11407868	T	C	T	C	T	Accurate
Marker1196	Chr. 2	11429299	G	G	A	G	A	Accurate
Marker1199	Chr. 2	11435973	A	A	G	A	G	Accurate
Marker1200	Chr. 2	11436035	A	A	G	A	G	Accurate
Marker1201	Chr. 2	11436056	G	G	T	G	T	Accurate
Marker1202	Chr. 2	11436253	C	C	T	C	T	Accurate
Marker1203	Chr. 2	11436296	G	G	C	G	C	Accurate
Marker1212	Chr. 2	11565550	G	G	A	G	A	Accurate
Marker1214	Chr. 2	11566007	T	T	C	T	C	Accurate
Marker1217	Chr. 2	11995420	G	A	G	A	G	Accurate
Marker1218	Chr. 2	12318340	A	A	G	A	G	Accurate
Marker1219	Chr. 2	12332131	A	A	G	A	G	Accurate
Marker1225	Chr. 2	12376234	C	C	T	C	T	Accurate
Marker1226	Chr. 2	12385701	G	G	A	G	A	Accurate
Marker1227	Chr. 2	12409651	G	G	A	G	A	Accurate
Marker1228	Chr. 2	12409833	G	G	C	G	C	Accurate
Marker1229	Chr. 2	12413132	C	C	T	C	T	Accurate
Marker1230	Chr. 2	12415845	A	A	G	A	G	Accurate

Marker1231	Chr. 2	12426969	T	T	C	T	C	Accurate
Marker1232	Chr. 2	12430101	G	G	A	G	A	Accurate
Marker1233	Chr. 2	12434199	C	C	A	C	A	Accurate
Marker1234	Chr. 2	12469741	G	G	A	G	A	Accurate
Marker1235	Chr. 2	12469830	T	T	C	T	C	Accurate
Marker1236	Chr. 2	12505216	G	G	C	G	C	Accurate
Marker1237	Chr. 2	12506226	T	T	A	T	A	Accurate
Marker1238	Chr. 2	12511431	A	A	G	A	G	Accurate
Marker1239	Chr. 2	12516747	C	C	G	C	G	Accurate
Marker1240	Chr. 2	12527042	G	G	C	G	C	Accurate
Marker1241	Chr. 2	12531813	C	C	T	C	T	Accurate
Marker1242	Chr. 2	12545551	C	C	A	C	A	Accurate
Marker1243	Chr. 2	12583724	A	A	G	A	G	Accurate
Marker1244	Chr. 2	12583974	A	A	C	A	C	Accurate
Marker1245	Chr. 2	12584726	G	G	A	G	A	Accurate
Marker1247	Chr. 2	12634377	C	C	T	C	T	Accurate
Marker1248	Chr. 2	12634581	A	A	C	A	C	Accurate
Marker1249	Chr. 2	12634638	T	T	G	T	G	Accurate
Marker1250	Chr. 2	12634866	A	A	C	A	C	Accurate
Marker1251	Chr. 2	12634894	T	T	A	T	A	Accurate
Marker1254	Chr. 2	14034710	A	A	G	A	G	Accurate
Marker1256	Chr. 2	14124512	A	A	G	A	G	Accurate
Marker1258	Chr. 2	14718905	A	G	A	G	N	Uncertain
Marker1261	Chr. 2	14810320	A	T	A	T	A	Accurate
Marker1262	Chr. 2	14810468	T	C	T	C	T	Accurate
Marker1263	Chr. 2	14815321	A	G	A	G	A	Accurate
Marker1267	Chr. 2	14861615	G	C	G	C	G	Accurate
Marker1268	Chr. 2	14867556	C	T	C	T	C	Accurate
Marker1269	Chr. 2	14867564	A	G	A	G	A	Accurate
Marker1270	Chr. 2	14867786	G	A	G	A	G	Accurate
Marker1271	Chr. 2	14867806	T	C	T	C	T	Accurate
Marker1272	Chr. 2	14896905	A	C	A	C	A	Accurate
Marker1273	Chr. 2	14897470	T	C	T	C	T	Accurate
Marker1274	Chr. 2	14902612	G	T	G	T	G	Accurate
Marker1275	Chr. 2	14902644	T	A	T	A	T	Accurate
Marker1276	Chr. 2	14909570	G	A	G	A	G	Accurate
Marker1277	Chr. 2	14915308	A	G	A	G	N	Uncertain
Marker1278	Chr. 2	15718209	C	C	G	C	G	Accurate
Marker1279	Chr. 2	15722081	C	A	C	A	C	Accurate
Marker1281	Chr. 2	16168911	C	C	T	C	T	Accurate
Marker1282	Chr. 2	16168956	A	A	G	A	G	Accurate
Marker1283	Chr. 2	16177493	G	G	A	G	A	Accurate
Marker1284	Chr. 2	16193334	G	G	A	G	A	Accurate
Marker1287	Chr. 2	16223175	T	T	C	T	C	Accurate
Marker1290	Chr. 2	17247862	G	G	A	G	A	Accurate
Marker1291	Chr. 2	17248027	C	C	T	C	T	Accurate
Marker1292	Chr. 2	17248043	G	G	T	G	T	Accurate

Marker1293	Chr. 2	17248068	C	C	T	C	T	Accurate
Marker1294	Chr. 2	17376162	A	A	G	A	G	Accurate
Marker1296	Chr. 2	17391834	C	C	A	C	A	Accurate
Marker1300	Chr. 2	17490461	G	T	G	K	G	Uncertain
Marker1301	Chr. 2	17565953	A	C	A	A	R	Inconsistent
Marker1304	Chr. 2	17743097	T	T	C	T	C	Accurate
Marker1307	Chr. 2	17890112	T	T	A	N	A	Uncertain
Marker1310	Chr. 2	17890324	C	C	T	N	T	Uncertain
Marker1313	Chr. 2	17946852	C	T	C	T	C	Accurate
Marker1314	Chr. 2	17947004	T	G	A	C	C	Inconsistent
Marker1319	Chr. 2	18103191	A	G	A	G	A	Accurate
Marker1321	Chr. 2	18181874	C	A	C	A	C	Accurate
Marker1322	Chr. 2	18555450	C	T	C	T	C	Accurate
Marker1324	Chr. 2	18632202	A	A	G	A	G	Accurate
Marker1325	Chr. 2	18632573	A	A	G	A	G	Accurate
Marker1327	Chr. 2	18664589	T	T	C	T	C	Accurate
Marker1328	Chr. 2	18664861	A	A	G	A	G	Accurate
Marker1329	Chr. 2	18665849	G	G	A	G	A	Accurate
Marker1330	Chr. 2	18695792	G	G	T	N	T	Uncertain
Marker1332	Chr. 2	18697438	C	C	T	C	Y	Uncertain
Marker1335	Chr. 2	18733160	T	T	C	T	C	Accurate
Marker1336	Chr. 2	18738783	A	A	G	A	G	Accurate
Marker1337	Chr. 2	18738949	A	A	T	A	T	Accurate
Marker1339	Chr. 2	18745998	T	T	C	Y	C	Uncertain
Marker1340	Chr. 2	18746024	A	A	G	A	G	Accurate
Marker1342	Chr. 2	18778936	C	T	C	T	C	Accurate
Marker1343	Chr. 2	18778958	T	C	T	C	T	Accurate
Marker1344	Chr. 2	18785254	T	C	T	C	T	Accurate
Marker1346	Chr. 2	18813403	G	A	G	A	G	Accurate
Marker1348	Chr. 2	18820885	G	A	G	A	G	Accurate
Marker1350	Chr. 2	18859180	T	C	T	C	T	Accurate
Marker1353	Chr. 2	18894264	T	C	T	C	T	Accurate
Marker1355	Chr. 2	18946150	T	C	T	C	T	Accurate
Marker1356	Chr. 2	18990006	T	C	T	C	T	Accurate
Marker1357	Chr. 2	18990238	T	C	T	C	T	Accurate
Marker1361	Chr. 2	19020446	T	T	A	T	A	Accurate
Marker1363	Chr. 2	19088869	G	T	G	T	G	Accurate
Marker1365	Chr. 2	19090135	C	G	C	G	C	Accurate
Marker1367	Chr. 2	19125750	C	T	C	T	C	Accurate
Marker1369	Chr. 2	19153148	C	A	C	A	C	Accurate
Marker1371	Chr. 2	19176379	G	G	A	G	A	Accurate
Marker1374	Chr. 2	19195468	G	G	A	G	A	Accurate
Marker1376	Chr. 2	19201553	T	T	A	T	A	Accurate
Marker1378	Chr. 2	19217042	A	A	G	A	G	Accurate
Marker1383	Chr. 2	19277365	T	T	C	T	C	Accurate
Marker1384	Chr. 2	19277655	C	C	A	C	A	Accurate
Marker1385	Chr. 2	19292571	G	A	G	A	G	Accurate

Marker1386	Chr. 2	19322155	C	T	C	T	C	Accurate
Marker1391	Chr. 2	19426944	A	G	A	G	A	Accurate
Marker1393	Chr. 2	19440905	A	C	A	C	A	Accurate
Marker1394	Chr. 2	19448123	T	C	T	C	T	Accurate
Marker1396	Chr. 2	19469716	C	T	C	T	C	Accurate
Marker1397	Chr. 2	19479595	C	T	C	T	C	Accurate
Marker1402	Chr. 2	19521589	T	C	T	C	T	Accurate
Marker1404	Chr. 2	19525225	C	T	C	T	C	Accurate
Marker1406	Chr. 2	19656893	T	T	G	G	G	Inconsistent
Marker1408	Chr. 2	19842649	G	G	A	G	A	Accurate
Marker1413	Chr. 2	23069349	G	A	G	A	G	Accurate
Marker1414	Chr. 2	23083429	G	A	G	A	G	Accurate
Marker1415	Chr. 2	23088345	T	G	T	G	T	Accurate
Marker1416	Chr. 2	23113851	A	G	A	G	A	Accurate
Marker1417	Chr. 2	23120399	G	A	G	A	G	Accurate
Marker1418	Chr. 2	23125468	T	G	T	G	T	Accurate
Marker1419	Chr. 2	23165843	T	C	T	C	T	Accurate
Marker1420	Chr. 2	23201417	G	A	G	A	G	Accurate
Marker1421	Chr. 2	23218579	C	A	C	A	C	Accurate
Marker1422	Chr. 2	23241235	T	A	T	A	T	Accurate
Marker1423	Chr. 2	23241502	G	A	G	A	G	Accurate
Marker1424	Chr. 2	23253841	G	A	G	A	G	Accurate
Marker1428	Chr. 2	23287860	A	C	A	C	A	Accurate
Marker1429	Chr. 2	23288082	C	T	C	T	C	Accurate
Marker1432	Chr. 2	23296298	T	C	T	C	T	Accurate
Marker1433	Chr. 2	23319658	A	G	A	G	A	Accurate
Marker1434	Chr. 2	23337990	T	A	T	A	T	Accurate
Marker1436	Chr. 2	23347819	C	T	C	T	C	Accurate
Marker1437	Chr. 2	23368824	T	C	T	C	T	Accurate
Marker1438	Chr. 2	23370115	A	G	A	G	A	Accurate
Marker1439	Chr. 2	23370139	G	A	G	A	G	Accurate
Marker1440	Chr. 2	23379050	G	A	G	A	G	Accurate
Marker1443	Chr. 2	23421439	C	T	C	T	C	Accurate
Marker1444	Chr. 2	23437917	G	A	G	A	G	Accurate
Marker1445	Chr. 2	23474781	C	T	C	T	C	Accurate
Marker1446	Chr. 2	23475080	T	G	T	G	T	Accurate
Marker1447	Chr. 2	23480736	G	A	G	A	G	Accurate
Marker1448	Chr. 2	23480748	T	G	T	G	T	Accurate
Marker1449	Chr. 2	23480749	T	A	T	A	T	Accurate
Marker1450	Chr. 2	23484905	A	G	A	G	A	Accurate
Marker1451	Chr. 2	23488700	A	T	A	T	A	Accurate
Marker1454	Chr. 2	23497323	C	T	C	T	C	Accurate
Marker1455	Chr. 2	23527902	A	G	A	G	A	Accurate
Marker1457	Chr. 2	23530181	C	T	C	T	C	Accurate
Marker1458	Chr. 2	23531464	T	A	T	A	T	Accurate
Marker1459	Chr. 2	23563744	C	T	C	T	C	Accurate
Marker1460	Chr. 2	23563764	G	A	G	A	G	Accurate

Marker1461	Chr. 2	23573267	C	T	C	T	C	Accurate
Marker1462	Chr. 2	23573284	T	C	T	C	T	Accurate
Marker1463	Chr. 2	23573319	C	T	C	T	C	Accurate
Marker1465	Chr. 2	23578181	G	A	G	A	G	Accurate
Marker1466	Chr. 2	23585694	G	A	G	A	G	Accurate
Marker1467	Chr. 2	23585902	A	C	A	C	N	Uncertain
Marker1468	Chr. 2	23586087	G	A	G	A	G	Accurate
Marker1469	Chr. 2	23586113	T	C	T	C	T	Accurate
Marker1470	Chr. 2	23697742	G	A	G	A	G	Accurate
Marker1471	Chr. 2	23699031	A	G	A	G	A	Accurate
Marker1473	Chr. 2	23699684	C	T	C	T	C	Accurate
Marker1474	Chr. 2	23707535	C	T	C	T	C	Accurate
Marker1475	Chr. 2	23765823	A	T	A	T	A	Accurate
Marker1476	Chr. 2	23766002	C	T	C	T	C	Accurate
Marker1477	Chr. 2	23772050	T	C	T	C	T	Accurate
Marker1479	Chr. 2	23778410	G	A	G	A	G	Accurate
Marker1480	Chr. 2	23805867	T	T	C	T	C	Accurate
Marker1481	Chr. 2	23850241	T	T	C	T	C	Accurate
Marker1483	Chr. 2	23892363	C	C	T	C	T	Accurate
Marker1484	Chr. 2	23937547	T	T	A	T	A	Accurate
Marker1485	Chr. 2	23945152	A	A	G	A	G	Accurate
Marker1486	Chr. 2	23950250	T	T	C	T	C	Accurate
Marker1487	Chr. 2	23955143	G	G	A	G	A	Accurate
Marker1488	Chr. 2	23959862	A	A	G	A	G	Accurate
Marker1489	Chr. 2	23972766	C	T	C	T	C	Accurate
Marker1490	Chr. 2	23972935	C	C	T	C	T	Accurate
Marker1491	Chr. 2	24016027	G	A	G	A	G	Accurate
Marker1494	Chr. 2	24180509	G	A	G	A	G	Accurate
Marker1496	Chr. 2	24216516	C	A	C	A	C	Accurate
Marker1497	Chr. 2	24231714	C	T	C	T	C	Accurate
Marker1501	Chr. 2	24254600	T	C	T	C	T	Accurate
Marker1502	Chr. 2	24254630	G	C	G	C	G	Accurate
Marker1503	Chr. 2	24254671	A	G	A	G	A	Accurate
Marker1505	Chr. 2	24333656	T	C	T	C	T	Accurate
Marker1506	Chr. 2	24333699	T	C	T	C	T	Accurate
Marker1507	Chr. 2	24373688	A	G	A	G	A	Accurate
Marker1509	Chr. 2	24381670	T	A	T	A	T	Accurate
Marker1510	Chr. 2	24390492	T	C	T	C	T	Accurate
Marker1511	Chr. 2	24390552	T	C	T	C	T	Accurate
Marker1513	Chr. 2	24398391	G	A	G	A	G	Accurate
Marker1520	Chr. 2	24567978	A	T	A	T	A	Accurate
Marker1521	Chr. 2	24568115	A	G	A	G	A	Accurate
Marker1522	Chr. 2	24578328	A	G	A	G	N	Uncertain
Marker1524	Chr. 2	24679821	C	C	A	C	A	Accurate
Marker1525	Chr. 2	24679863	G	A	G	A	G	Accurate
Marker1527	Chr. 2	24739442	C	T	C	T	C	Accurate
Marker1529	Chr. 2	24798442	G	A	G	A	G	Accurate

Marker1530	Chr. 2	24799911	A	A	G	A	G	Accurate
Marker1531	Chr. 2	24800229	G	G	A	G	A	Accurate
Marker1532	Chr. 2	24800291	G	G	A	G	A	Accurate
Marker1533	Chr. 2	24800464	T	T	G	T	G	Accurate
Marker1535	Chr. 2	24842609	C	T	C	T	C	Accurate
Marker1536	Chr. 2	24849227	G	C	G	C	G	Accurate
Marker1537	Chr. 2	24849780	A	G	A	G	A	Accurate
Marker1538	Chr. 2	24875363	T	A	T	A	T	Accurate
Marker1539	Chr. 2	24875890	C	T	C	T	C	Accurate
Marker1548	Chr. 2	24917047	G	A	G	A	G	Accurate
Marker1549	Chr. 2	25002724	T	A	T	A	T	Accurate
Marker1550	Chr. 2	25069669	T	C	T	C	T	Accurate
Marker1551	Chr. 2	25069744	T	C	T	C	T	Accurate
Marker1552	Chr. 2	25076135	C	T	C	T	C	Accurate
Marker1555	Chr. 2	25090365	G	A	G	A	G	Accurate
Marker1556	Chr. 2	25093462	G	A	G	A	G	Accurate
Marker1557	Chr. 2	25100902	G	A	G	A	G	Accurate
Marker1558	Chr. 2	25102328	G	C	G	C	G	Accurate
Marker1561	Chr. 2	25343020	C	T	C	T	C	Accurate
Marker1564	Chr. 2	25348325	T	C	T	C	T	Accurate
Marker1566	Chr. 2	25430619	G	T	G	T	G	Accurate
Marker1568	Chr. 2	25594212	A	A	G	A	G	Accurate
Marker1569	Chr. 2	25613450	T	T	C	T	C	Accurate
Marker1570	Chr. 2	25704185	G	A	G	A	G	Accurate
Marker1571	Chr. 2	25704197	T	C	T	C	T	Accurate
Marker1573	Chr. 2	25777539	T	C	T	C	T	Accurate
Marker1576	Chr. 2	25911619	G	A	G	A	G	Accurate
Marker1577	Chr. 2	25937114	C	A	C	A	C	Accurate
Marker1578	Chr. 2	26287668	A	C	A	C	A	Accurate
Marker1579	Chr. 2	26287910	G	A	G	A	G	Accurate
Marker1582	Chr. 2	26310456	A	G	A	G	A	Accurate
Marker1583	Chr. 2	26313076	C	T	C	T	C	Accurate
Marker1584	Chr. 2	26313104	A	G	A	G	A	Accurate
Marker1586	Chr. 2	26313280	G	C	G	C	G	Accurate
Marker1587	Chr. 2	26313526	A	T	A	T	A	Accurate
Marker1588	Chr. 2	26473592	A	C	A	C	A	Accurate
Marker1589	Chr. 2	26474072	A	T	A	T	A	Accurate
Marker1590	Chr. 2	26474167	G	C	G	C	G	Accurate
Marker1591	Chr. 2	26474188	G	A	G	A	G	Accurate
Marker1594	Chr. 2	26573160	A	G	A	G	A	Accurate
Marker1597	Chr. 2	26876699	T	C	T	C	T	Accurate
Marker1598	Chr. 2	26876930	C	T	C	T	C	Accurate
Marker1600	Chr. 2	27182024	G	G	C	G	C	Accurate
Marker1601	Chr. 2	27218349	A	A	G	A	G	Accurate
Marker1602	Chr. 2	27673070	G	G	A	G	A	Accurate
Marker1603	Chr. 2	27742926	T	T	A	T	A	Accurate
Marker1604	Chr. 2	27800331	G	G	T	G	T	Accurate

Marker1605	Chr. 2	27808008	C	C	G	C	G	Accurate
Marker1606	Chr. 2	27883052	G	G	A	G	A	Accurate
Marker1607	Chr. 2	27890151	G	G	A	G	A	Accurate
Marker1608	Chr. 2	27890164	T	T	G	T	G	Accurate
Marker1609	Chr. 2	27908612	C	C	A	C	A	Accurate
Marker1610	Chr. 2	27908613	A	A	T	A	T	Accurate
Marker1611	Chr. 2	28002681	A	A	G	A	G	Accurate
Marker1612	Chr. 2	28240529	T	T	C	T	C	Accurate
Marker1613	Chr. 2	28276519	A	A	G	A	G	Accurate
Marker1614	Chr. 2	28309590	A	A	G	A	G	Accurate
Marker1615	Chr. 2	28318808	T	T	C	T	C	Accurate
Marker1616	Chr. 2	28328468	C	C	A	C	A	Accurate
Marker1617	Chr. 2	28354910	T	T	C	T	C	Accurate
Marker1618	Chr. 2	28355309	C	C	T	C	T	Accurate
Marker1619	Chr. 2	28464917	G	G	A	G	A	Accurate
Marker1620	Chr. 2	28465160	G	G	A	G	A	Accurate
Marker1621	Chr. 2	28520139	T	T	C	T	C	Accurate
Marker1622	Chr. 2	28580911	G	A	G	A	G	Accurate
Marker1623	Chr. 2	28580963	G	C	G	C	G	Accurate
Marker1626	Chr. 2	28587181	C	T	C	T	C	Accurate
Marker1627	Chr. 2	28587226	G	T	G	T	G	Accurate
Marker1628	Chr. 2	28686409	G	G	A	G	A	Accurate
Marker1629	Chr. 2	28701114	C	C	T	N	T	Uncertain
Marker1630	Chr. 2	28764219	G	G	C	G	C	Accurate
Marker1631	Chr. 2	28764222	A	A	G	A	G	Accurate
Marker1632	Chr. 2	28771211	T	T	C	T	C	Accurate
Marker1633	Chr. 2	28771235	T	T	C	T	C	Accurate
Marker1634	Chr. 2	28818667	T	T	G	T	G	Accurate
Marker1635	Chr. 2	28853360	A	T	A	T	A	Accurate
Marker1636	Chr. 2	28854405	T	A	T	A	T	Accurate
Marker1637	Chr. 2	28863982	G	A	G	A	G	Accurate
Marker1638	Chr. 2	28864194	T	G	T	G	T	Accurate
Marker1639	Chr. 2	28866085	T	C	T	C	T	Accurate
Marker1640	Chr. 2	28866311	A	G	A	G	A	Accurate
Marker1641	Chr. 2	28938822	C	A	C	A	C	Accurate
Marker1642	Chr. 2	28953491	T	T	G	T	G	Accurate
Marker1643	Chr. 2	28989067	G	A	G	A	G	Accurate
Marker1644	Chr. 2	28989086	A	G	A	G	A	Accurate
Marker1645	Chr. 2	28992832	T	G	T	G	T	Accurate
Marker1646	Chr. 2	28992856	G	A	G	A	G	Accurate
Marker1647	Chr. 2	29006617	C	T	C	T	C	Accurate
Marker1648	Chr. 2	29025603	T	G	T	G	T	Accurate
Marker1649	Chr. 2	29035622	G	T	G	T	G	Accurate
Marker1650	Chr. 2	29035657	C	T	C	T	C	Accurate
Marker1652	Chr. 2	29062206	T	T	G	T	G	Accurate
Marker1653	Chr. 2	29062232	A	A	G	A	G	Accurate
Marker1655	Chr. 2	29113054	C	C	T	C	T	Accurate

Marker1657	Chr. 2	29174888	A	G	A	G	A	Accurate
Marker1658	Chr. 2	29195322	T	T	G	T	G	Accurate
Marker1659	Chr. 2	29240879	T	T	A	T	A	Accurate
Marker1660	Chr. 2	29414449	G	A	G	A	G	Accurate
Marker1661	Chr. 2	29415204	T	C	T	C	T	Accurate
Marker1662	Chr. 2	29443860	C	C	T	C	T	Accurate
Marker1663	Chr. 2	29443908	G	G	A	G	A	Accurate
Marker1664	Chr. 2	29457199	C	C	A	C	A	Accurate
Marker1665	Chr. 2	29457255	C	C	T	C	T	Accurate
Marker1666	Chr. 2	29463941	T	T	C	T	C	Accurate
Marker1667	Chr. 2	29464244	T	T	C	N	C	Uncertain
Marker1668	Chr. 2	29474619	C	C	T	C	T	Accurate
Marker1669	Chr. 2	29480230	G	G	A	G	A	Accurate
Marker1670	Chr. 2	29489375	G	G	C	G	C	Accurate
Marker1671	Chr. 2	29489377	C	C	T	C	T	Accurate
Marker1672	Chr. 2	29509165	T	T	C	T	C	Accurate
Marker1673	Chr. 2	29513853	G	G	T	G	T	Accurate
Marker1674	Chr. 2	29534720	T	T	G	T	A	Uncertain
Marker1675	Chr. 2	29540218	C	G	C	G	C	Accurate
Marker1676	Chr. 2	29545223	A	A	G	A	G	Accurate
Marker1677	Chr. 2	29546844	C	C	T	C	T	Accurate
Marker1678	Chr. 2	29560423	C	G	C	G	C	Accurate
Marker1679	Chr. 2	29565073	T	C	T	C	T	Accurate
Marker1680	Chr. 2	29565293	T	G	T	G	T	Accurate
Marker1681	Chr. 2	29567035	A	G	A	G	A	Accurate
Marker1682	Chr. 2	29567065	A	G	A	G	A	Accurate
Marker1683	Chr. 2	29567067	A	C	A	C	A	Accurate
Marker1684	Chr. 2	29568800	A	C	A	C	A	Accurate
Marker1685	Chr. 2	29568808	A	G	A	G	A	Accurate
Marker1686	Chr. 2	29583204	C	C	A	C	A	Accurate
Marker1687	Chr. 2	29606175	T	C	T	C	T	Accurate
Marker1688	Chr. 2	29611740	A	G	A	G	A	Accurate
Marker1689	Chr. 2	29611798	A	T	A	T	A	Accurate
Marker1690	Chr. 2	29619953	C	T	C	T	C	Accurate
Marker1691	Chr. 2	29619968	T	A	T	A	T	Accurate
Marker1692	Chr. 2	29622417	T	C	T	C	T	Accurate
Marker1693	Chr. 2	29630689	A	C	A	C	A	Accurate
Marker1695	Chr. 2	29657257	G	G	A	G	A	Accurate
Marker1696	Chr. 2	29657326	A	A	G	N	G	Uncertain
Marker1697	Chr. 2	29659004	A	A	T	A	T	Accurate
Marker1698	Chr. 2	29714938	C	C	T	C	T	Accurate
Marker1699	Chr. 2	29715094	A	A	C	A	C	Accurate
Marker1700	Chr. 2	29756444	G	G	T	G	T	Accurate
Marker1702	Chr. 2	29840517	A	T	A	T	A	Accurate
Marker1705	Chr. 2	29958111	A	G	A	G	A	Accurate
Marker1710	Chr. 2	30037048	G	C	G	C	G	Accurate
Marker1712	Chr. 2	30071285	G	G	A	G	A	Accurate

Marker1713	Chr. 2	30071787	A	A	T	A	T	Accurate
Marker1714	Chr. 2	30080888	T	T	C	T	C	Accurate
Marker1715	Chr. 2	30106139	G	A	G	A	G	Accurate
Marker1716	Chr. 2	30108571	T	A	T	A	T	Accurate
Marker1717	Chr. 2	30139705	A	A	G	A	G	Accurate
Marker1718	Chr. 2	30139831	C	C	T	C	T	Accurate
Marker1719	Chr. 2	30164366	C	C	A	C	A	Accurate
Marker1720	Chr. 2	30164424	G	G	A	G	A	Accurate
Marker1721	Chr. 2	30169665	C	C	T	C	T	Accurate
Marker1722	Chr. 2	30170861	A	A	G	A	G	Accurate
Marker1723	Chr. 2	30171027	T	T	C	T	C	Accurate
Marker1724	Chr. 2	30188565	C	T	C	T	C	Accurate
Marker1725	Chr. 2	30195261	C	C	T	C	T	Accurate
Marker1726	Chr. 2	30225725	C	T	C	T	C	Accurate
Marker1727	Chr. 2	30227157	T	A	T	A	T	Accurate
Marker1729	Chr. 2	30227884	C	T	C	T	C	Accurate
Marker1730	Chr. 2	30227891	T	G	T	G	T	Accurate
Marker1731	Chr. 2	30237513	A	A	G	A	G	Accurate
Marker1732	Chr. 2	30239393	C	C	G	C	G	Accurate
Marker1733	Chr. 2	30241212	A	A	G	A	G	Accurate
Marker1737	Chr. 2	30394242	G	G	A	G	A	Accurate
Marker1738	Chr. 2	30400449	G	G	A	G	A	Accurate
Marker1739	Chr. 2	30408821	G	G	C	G	C	Accurate
Marker1740	Chr. 2	30456874	G	G	A	G	A	Accurate
Marker1741	Chr. 2	30465990	G	G	A	G	A	Accurate
Marker1742	Chr. 2	30487435	C	C	T	C	T	Accurate
Marker1743	Chr. 2	30497025	A	A	T	A	T	Accurate
Marker1744	Chr. 2	30506789	A	A	G	N	G	Uncertain
Marker1745	Chr. 2	30508515	G	G	A	G	A	Accurate
Marker1746	Chr. 2	30726859	T	G	T	G	T	Accurate
Marker1747	Chr. 2	30957621	C	T	C	T	C	Accurate
Marker1748	Chr. 2	30959181	T	C	T	C	T	Accurate
Marker1749	Chr. 2	30988259	A	G	A	G	A	Accurate
Marker1750	Chr. 2	30994491	C	T	C	T	C	Accurate
Marker1751	Chr. 2	30994630	A	G	A	G	A	Accurate
Marker1752	Chr. 2	31015268	C	C	T	C	T	Accurate
Marker1753	Chr. 2	31046770	A	G	A	G	A	Accurate
Marker1754	Chr. 2	31048012	A	G	A	G	A	Accurate
Marker1755	Chr. 2	31056300	G	C	G	C	G	Accurate
Marker1756	Chr. 2	31058984	T	G	T	G	T	Accurate
Marker1757	Chr. 2	31072990	A	C	A	C	A	Accurate
Marker1758	Chr. 2	31074019	C	T	C	T	C	Accurate
Marker1759	Chr. 2	31127066	A	G	A	G	A	Accurate
Marker1760	Chr. 2	31131008	G	A	G	A	G	Accurate
Marker1761	Chr. 2	31131066	A	G	A	G	A	Accurate
Marker1762	Chr. 2	31145943	A	G	A	G	A	Accurate
Marker1763	Chr. 2	31165839	G	C	G	C	G	Accurate

Marker1764	Chr. 2	31195946	G	A	G	A	G	Accurate
Marker1765	Chr. 2	31195977	C	T	C	T	N	Uncertain
Marker1766	Chr. 2	31288446	G	G	A	G	A	Accurate
Marker1768	Chr. 2	31484362	C	C	G	C	G	Accurate
Marker1770	Chr. 2	31574847	C	A	C	A	C	Accurate
Marker1771	Chr. 2	31579206	C	C	T	C	T	Accurate
Marker1772	Chr. 2	31599456	A	A	G	A	G	Accurate
Marker1773	Chr. 2	31922903	A	A	G	A	G	Accurate
Marker1774	Chr. 2	31923097	C	C	T	C	T	Accurate
Marker1775	Chr. 2	31929047	G	G	A	G	A	Accurate
Marker1776	Chr. 2	31940105	G	G	T	G	T	Accurate
Marker1777	Chr. 2	31946922	A	A	C	A	C	Accurate
Marker1778	Chr. 2	31947329	A	A	G	A	G	Accurate
Marker1779	Chr. 2	31968794	C	C	A	C	A	Accurate
Marker1780	Chr. 2	31970306	G	G	T	G	T	Accurate
Marker1781	Chr. 2	31986115	A	A	C	A	C	Accurate
Marker1782	Chr. 2	32215670	C	C	T	C	T	Accurate
Marker1783	Chr. 2	32226289	C	C	T	C	T	Accurate
Marker1784	Chr. 2	32226295	C	C	A	C	A	Accurate
Marker1787	Chr. 2	32365050	T	T	G	T	G	Accurate
Marker1789	Chr. 2	32395850	C	T	C	T	C	Accurate
Marker1793	Chr. 2	32404492	T	T	C	T	C	Accurate
Marker1795	Chr. 2	32538582	A	G	A	G	A	Accurate
Marker1796	Chr. 2	32788071	A	G	A	G	A	Accurate
Marker1798	Chr. 2	32858254	C	A	C	A	C	Accurate
Marker1801	Chr. 2	32995025	T	C	T	C	T	Accurate
Marker1805	Chr. 2	33091300	G	G	T	G	T	Accurate
Marker1806	Chr. 2	33091313	T	T	C	T	C	Accurate
Marker1807	Chr. 2	33203335	T	T	G	T	G	Accurate
Marker1808	Chr. 2	33255292	C	T	C	T	C	Accurate
Marker1809	Chr. 2	33258427	A	G	A	G	A	Accurate
Marker1810	Chr. 2	33949568	T	C	T	C	T	Accurate
Marker1814	Chr. 2	34261227	G	C	G	C	G	Accurate
Marker1816	Chr. 2	34298199	T	C	T	C	T	Accurate
Marker1824	Chr. 3	2745711	T	T	C	T	C	Accurate
Marker1828	Chr. 3	2784253	A	A	C	A	C	Accurate
Marker1829	Chr. 3	2937123	T	T	C	T	C	Accurate
Marker1831	Chr. 3	2975403	G	G	A	G	A	Accurate
Marker1832	Chr. 3	2982525	A	A	G	A	G	Accurate
Marker1833	Chr. 3	3009952	T	T	A	T	A	Accurate
Marker1834	Chr. 3	3032908	C	C	G	C	G	Accurate
Marker1835	Chr. 3	3172478	T	T	C	N	C	Uncertain
Marker1836	Chr. 3	3174902	A	C	A	C	A	Accurate
Marker1843	Chr. 3	3388578	C	C	A	C	A	Accurate
Marker1846	Chr. 3	3707754	C	T	C	T	C	Accurate
Marker1851	Chr. 3	3899429	G	T	G	T	G	Accurate
Marker1858	Chr. 3	4402779	C	T	C	T	C	Accurate

Marker1861	Chr. 3	4649905	A	G	A	G	A	Accurate
Marker1862	Chr. 3	4650145	G	A	G	A	G	Accurate
Marker1864	Chr. 3	4670711	G	A	G	A	G	Accurate
Marker1866	Chr. 3	4708582	T	C	T	C	T	Accurate
Marker1867	Chr. 3	4743025	T	C	T	C	T	Accurate
Marker1868	Chr. 3	4743199	T	C	T	C	T	Accurate
Marker1869	Chr. 3	4754288	A	C	A	M	A	Uncertain
Marker1871	Chr. 3	4763091	A	T	A	T	A	Accurate
Marker1881	Chr. 3	5062301	C	T	C	T	C	Accurate
Marker1882	Chr. 3	5062302	T	A	T	A	T	Accurate
Marker1886	Chr. 3	5104823	T	C	T	C	T	Accurate
Marker1889	Chr. 3	5129660	A	T	A	T	A	Accurate
Marker1892	Chr. 3	5142580	G	A	G	A	G	Accurate
Marker1893	Chr. 3	5166527	G	A	G	A	G	Accurate
Marker1895	Chr. 3	5172949	T	G	T	G	T	Accurate
Marker1896	Chr. 3	5173178	A	G	A	G	A	Accurate
Marker1899	Chr. 3	5185969	A	G	A	G	A	Accurate
Marker1902	Chr. 3	5229179	T	G	T	G	T	Accurate
Marker1903	Chr. 3	5246924	T	C	T	C	T	Accurate
Marker1904	Chr. 3	5247112	A	T	A	T	A	Accurate
Marker1906	Chr. 3	5335601	A	A	G	A	G	Accurate
Marker1908	Chr. 3	5476139	C	T	C	T	C	Accurate
Marker1909	Chr. 3	5476372	G	A	G	A	G	Accurate
Marker1914	Chr. 3	5670898	T	C	T	C	T	Accurate
Marker1916	Chr. 3	5680917	G	T	G	T	G	Accurate
Marker1917	Chr. 3	5686151	T	T	C	T	C	Accurate
Marker1918	Chr. 3	5711423	T	A	T	A	T	Accurate
Marker1920	Chr. 3	5770489	G	G	C	G	C	Accurate
Marker1921	Chr. 3	5812919	T	C	T	C	T	Accurate
Marker1922	Chr. 3	5812968	G	A	G	A	G	Accurate
Marker1925	Chr. 3	5880575	A	G	A	G	A	Accurate
Marker1927	Chr. 3	5901630	T	C	T	C	T	Accurate
Marker1929	Chr. 3	6095421	C	C	T	C	T	Accurate
Marker1932	Chr. 3	6736576	C	C	T	C	T	Accurate
Marker1934	Chr. 3	6935795	G	G	T	G	T	Accurate
Marker1935	Chr. 3	6973501	C	C	T	C	T	Accurate
Marker1937	Chr. 3	7019899	G	G	A	G	A	Accurate
Marker1942	Chr. 3	7081564	C	C	G	C	G	Accurate
Marker1945	Chr. 3	7175357	G	A	G	A	G	Accurate
Marker1947	Chr. 3	7201608	C	T	C	T	C	Accurate
Marker1948	Chr. 3	7201774	A	G	A	G	A	Accurate
Marker1952	Chr. 3	7236277	A	C	A	C	A	Accurate
Marker1954	Chr. 3	7269354	G	A	G	A	G	Accurate
Marker1956	Chr. 3	7451909	C	T	C	T	C	Accurate
Marker1957	Chr. 3	7465957	G	A	G	A	G	Accurate
Marker1958	Chr. 3	7472867	G	A	G	A	G	Accurate
Marker1960	Chr. 3	7484194	G	T	G	T	G	Accurate

Marker1961	Chr. 3	7490065	C	G	C	G	C	Accurate
Marker1965	Chr. 3	7608146	G	G	A	G	A	Accurate
Marker1968	Chr. 3	7644160	T	T	C	T	C	Accurate
Marker1969	Chr. 3	7961890	A	A	G	A	G	Accurate
Marker1970	Chr. 3	7988072	C	C	G	C	G	Accurate
Marker1971	Chr. 3	7988367	T	T	C	T	C	Accurate
Marker1973	Chr. 3	8137632	C	C	T	C	T	Accurate
Marker1974	Chr. 3	8302654	G	G	A	G	A	Accurate
Marker1975	Chr. 3	8335694	A	A	G	A	G	Accurate
Marker1976	Chr. 3	8359949	A	A	G	A	G	Accurate
Marker1978	Chr. 3	8372421	A	A	C	A	C	Accurate
Marker1980	Chr. 3	8584826	A	A	G	R	G	Uncertain
Marker1982	Chr. 3	8591961	T	T	C	T	C	Accurate
Marker1984	Chr. 3	8658433	C	C	A	C	A	Accurate
Marker1985	Chr. 3	8658614	T	T	C	T	C	Accurate
Marker1986	Chr. 3	8690736	G	G	T	G	T	Accurate
Marker1987	Chr. 3	8695300	C	C	T	C	T	Accurate
Marker1988	Chr. 3	8700924	C	C	A	C	A	Accurate
Marker1989	Chr. 3	8700932	A	A	G	A	G	Accurate
Marker1990	Chr. 3	8704519	T	T	C	T	C	Accurate
Marker1991	Chr. 3	8706278	A	A	G	A	G	Accurate
Marker1993	Chr. 3	8746802	G	G	A	G	A	Accurate
Marker1995	Chr. 3	9312679	C	C	T	C	T	Accurate
Marker1996	Chr. 3	9316135	C	C	T	C	T	Accurate
Marker1997	Chr. 3	9364794	C	C	A	C	A	Accurate
Marker1998	Chr. 3	9377501	T	T	G	T	G	Accurate
Marker1999	Chr. 3	9388822	G	G	A	G	A	Accurate
Marker2000	Chr. 3	9415283	T	T	C	T	C	Accurate
Marker2001	Chr. 3	9447250	T	T	C	T	C	Accurate
Marker2002	Chr. 3	9448991	T	T	C	T	C	Accurate
Marker2003	Chr. 3	9461752	G	G	A	G	A	Accurate
Marker2004	Chr. 3	9462681	G	G	A	G	A	Accurate
Marker2005	Chr. 3	9468543	G	G	A	G	A	Accurate
Marker2006	Chr. 3	9468545	T	T	G	T	G	Accurate
Marker2007	Chr. 3	9472768	G	G	A	G	A	Accurate
Marker2008	Chr. 3	9508673	T	T	C	T	C	Accurate
Marker2009	Chr. 3	9508851	G	G	T	G	T	Accurate
Marker2011	Chr. 3	9594250	A	A	G	A	G	Accurate
Marker2012	Chr. 3	9597745	A	A	G	A	G	Accurate
Marker2014	Chr. 3	9672879	A	A	G	A	G	Accurate
Marker2020	Chr. 3	9833419	G	A	G	A	G	Accurate
Marker2021	Chr. 3	9833533	T	A	T	A	T	Accurate
Marker2022	Chr. 3	9973589	G	A	G	A	G	Accurate
Marker2023	Chr. 3	9992086	T	G	T	G	T	Accurate
Marker2024	Chr. 3	10000040	A	G	A	G	A	Accurate
Marker2025	Chr. 3	10001724	A	G	A	G	A	Accurate
Marker2027	Chr. 3	10055845	A	A	T	A	T	Accurate

Marker2028	Chr. 3	10056500	G	G	T	G	T	Accurate
Marker2030	Chr. 3	10066845	A	A	G	A	G	Accurate
Marker2031	Chr. 3	10067121	A	A	G	A	G	Accurate
Marker2032	Chr. 3	10082637	G	G	C	N	C	Uncertain
Marker2033	Chr. 3	10084608	A	A	G	A	G	Accurate
Marker2034	Chr. 3	10098257	C	C	T	C	T	Accurate
Marker2035	Chr. 3	10112735	A	G	A	G	A	Accurate
Marker2036	Chr. 3	10118128	G	T	G	T	G	Accurate
Marker2037	Chr. 3	10121423	T	C	T	C	T	Accurate
Marker2038	Chr. 3	10141543	A	G	A	G	A	Accurate
Marker2039	Chr. 3	10145697	G	A	G	A	G	Accurate
Marker2040	Chr. 3	10158984	A	G	A	G	A	Accurate
Marker2041	Chr. 3	10170030	G	A	G	A	G	Accurate
Marker2042	Chr. 3	10201334	G	T	G	T	G	Accurate
Marker2043	Chr. 3	10201335	G	C	G	C	G	Accurate
Marker2044	Chr. 3	10201336	A	C	A	C	A	Accurate
Marker2045	Chr. 3	10222361	G	A	G	A	G	Accurate
Marker2046	Chr. 3	10389367	C	T	C	T	C	Accurate
Marker2047	Chr. 3	10392768	C	A	C	A	C	Accurate
Marker2050	Chr. 3	10468389	T	C	T	C	T	Accurate
Marker2051	Chr. 3	10604550	T	C	T	C	T	Accurate
Marker2058	Chr. 3	12940694	T	C	T	C	T	Accurate
Marker2064	Chr. 3	18501409	G	G	T	G	T	Accurate
Marker2067	Chr. 3	18506884	T	T	C	T	C	Accurate
Marker2068	Chr. 3	18522880	A	A	G	A	G	Accurate
Marker2069	Chr. 3	18522885	G	G	A	G	A	Accurate
Marker2071	Chr. 3	18525709	T	T	C	T	C	Accurate
Marker2072	Chr. 3	18526657	G	G	T	G	T	Accurate
Marker2073	Chr. 3	18532456	T	T	C	T	C	Accurate
Marker2074	Chr. 3	18532472	A	A	G	A	G	Accurate
Marker2077	Chr. 3	18536718	A	A	T	A	T	Accurate
Marker2078	Chr. 3	18536844	A	A	C	A	C	Accurate
Marker2082	Chr. 3	18565880	A	A	T	A	T	Accurate
Marker2087	Chr. 3	18600175	C	T	C	T	C	Accurate
Marker2089	Chr. 3	18951987	C	T	C	T	C	Accurate
Marker2090	Chr. 3	18959066	G	C	G	C	G	Accurate
Marker2091	Chr. 3	19205515	C	T	C	T	C	Accurate
Marker2094	Chr. 3	19235588	A	C	A	C	A	Accurate
Marker2095	Chr. 3	19237373	C	T	C	T	C	Accurate
Marker2096	Chr. 3	19252835	T	T	C	T	C	Accurate
Marker2097	Chr. 3	19281909	C	T	C	T	C	Accurate
Marker2098	Chr. 3	19281917	T	C	T	C	T	Accurate
Marker2106	Chr. 3	19982488	T	G	T	G	T	Accurate
Marker2107	Chr. 3	20304466	G	A	G	A	G	Accurate
Marker2108	Chr. 3	20305942	A	T	A	T	A	Accurate
Marker2109	Chr. 3	20305943	T	G	T	G	T	Accurate
Marker2111	Chr. 3	20478337	T	G	T	G	T	Accurate

Marker2113	Chr. 3	20480034	G	A	G	R	G	Uncertain
Marker2114	Chr. 3	20480054	A	G	A	G	A	Accurate
Marker2116	Chr. 3	20500636	A	G	A	G	A	Accurate
Marker2117	Chr. 3	20503506	T	C	T	C	T	Accurate
Marker2118	Chr. 3	20508477	G	A	G	A	G	Accurate
Marker2120	Chr. 3	20521472	G	A	G	A	G	Accurate
Marker2121	Chr. 3	20563327	C	T	C	T	C	Accurate
Marker2125	Chr. 3	20638091	T	C	T	C	T	Accurate
Marker2127	Chr. 3	20656339	A	G	A	G	A	Accurate
Marker2131	Chr. 3	20765270	A	G	A	G	A	Accurate
Marker2132	Chr. 3	20771477	G	T	G	T	G	Accurate
Marker2133	Chr. 3	20789506	G	A	G	A	G	Accurate
Marker2134	Chr. 3	20795651	C	T	C	T	C	Accurate
Marker2137	Chr. 3	20966114	G	A	G	A	G	Accurate
Marker2140	Chr. 3	21180384	G	A	G	A	G	Accurate
Marker2142	Chr. 3	21203179	A	G	A	G	A	Accurate
Marker2144	Chr. 3	21252935	C	T	C	T	C	Accurate
Marker2145	Chr. 3	21258374	T	C	T	C	T	Accurate
Marker2147	Chr. 3	21339345	T	T	A	T	A	Accurate
Marker2148	Chr. 3	21339355	A	A	G	A	G	Accurate
Marker2149	Chr. 3	21339415	C	C	A	C	A	Accurate
Marker2153	Chr. 3	21538900	G	A	G	A	G	Accurate
Marker2155	Chr. 3	21543185	C	T	C	T	C	Accurate
Marker2162	Chr. 3	22601032	G	A	G	A	G	Accurate
Marker2167	Chr. 3	22680754	G	T	G	T	G	Accurate
Marker2174	Chr. 3	23410564	T	T	A	T	A	Accurate
Marker2176	Chr. 3	23456944	T	T	C	T	C	Accurate
Marker2177	Chr. 3	23456953	C	C	T	C	T	Accurate
Marker2180	Chr. 3	23499879	T	T	C	T	C	Accurate
Marker2182	Chr. 3	24947753	A	A	C	A	C	Accurate
Marker2185	Chr. 3	25643314	A	A	G	A	G	Accurate
Marker2186	Chr. 3	25643325	G	G	T	G	T	Accurate
Marker2187	Chr. 3	25643500	A	A	T	A	T	Accurate
Marker2188	Chr. 3	25659902	C	C	T	C	T	Accurate
Marker2190	Chr. 3	25838574	T	T	A	T	A	Accurate
Marker2191	Chr. 3	25842084	A	A	T	A	T	Accurate
Marker2192	Chr. 3	25842795	T	T	A	T	A	Accurate
Marker2193	Chr. 3	25866946	A	A	G	A	G	Accurate
Marker2195	Chr. 3	25969773	T	G	T	G	T	Accurate
Marker2197	Chr. 3	25971507	A	C	A	C	A	Accurate
Marker2201	Chr. 3	26044135	G	A	G	A	G	Accurate
Marker2204	Chr. 3	26067967	T	G	T	G	T	Accurate
Marker2205	Chr. 3	26068799	G	T	G	T	G	Accurate
Marker2215	Chr. 3	26470499	T	C	T	C	T	Accurate
Marker2218	Chr. 3	26834087	C	T	C	T	C	Accurate
Marker2219	Chr. 3	26848699	T	C	T	C	T	Accurate
Marker2220	Chr. 3	26863224	T	A	T	A	T	Accurate

Marker2221	Chr. 3	26904153	C	C	A	N	A	Uncertain
Marker2222	Chr. 3	27083547	C	A	C	A	C	Accurate
Marker2224	Chr. 3	27085509	T	A	T	A	T	Accurate
Marker2230	Chr. 3	27128233	G	A	G	A	G	Accurate
Marker2231	Chr. 3	27129705	T	C	T	C	T	Accurate
Marker2236	Chr. 3	27174312	A	T	A	T	A	Accurate
Marker2241	Chr. 3	27593423	T	T	A	T	A	Accurate
Marker2243	Chr. 3	28180989	G	G	A	G	A	Accurate
Marker2245	Chr. 3	28287772	C	T	C	T	C	Accurate
Marker2247	Chr. 3	28291513	C	A	C	A	C	Accurate
Marker2249	Chr. 3	28318207	T	C	T	C	T	Accurate
Marker2251	Chr. 3	28332232	G	C	G	C	G	Accurate
Marker2253	Chr. 3	28342123	A	G	A	G	A	Accurate
Marker2254	Chr. 3	28369813	A	G	A	G	A	Accurate
Marker2261	Chr. 3	28454234	T	C	T	C	T	Accurate
Marker2264	Chr. 3	28499666	A	C	A	C	A	Accurate
Marker2265	Chr. 3	28539945	A	G	A	G	A	Accurate
Marker2268	Chr. 3	28589903	C	A	C	A	C	Accurate
Marker2274	Chr. 4	160616	G	G	A	G	A	Accurate
Marker2278	Chr. 4	164490	A	A	G	A	G	Accurate
Marker2279	Chr. 4	164777	G	G	T	G	T	Accurate
Marker2280	Chr. 4	179952	G	G	A	G	A	Accurate
Marker2281	Chr. 4	196854	A	A	C	A	C	Accurate
Marker2282	Chr. 4	196879	T	T	G	T	G	Accurate
Marker2283	Chr. 4	204748	A	A	G	A	G	Accurate
Marker2284	Chr. 4	208617	T	T	C	T	C	Accurate
Marker2286	Chr. 4	209327	T	T	C	T	C	Accurate
Marker2288	Chr. 4	229334	T	T	A	T	A	Accurate
Marker2290	Chr. 4	278121	A	A	G	A	G	Accurate
Marker2291	Chr. 4	281677	C	C	T	C	T	Accurate
Marker2293	Chr. 4	300699	C	C	A	C	A	Accurate
Marker2296	Chr. 4	330336	T	T	C	T	C	Accurate
Marker2297	Chr. 4	330475	T	T	G	T	G	Accurate
Marker2298	Chr. 4	358175	T	T	G	T	G	Accurate
Marker2299	Chr. 4	361027	C	C	T	C	T	Accurate
Marker2300	Chr. 4	409282	C	C	T	C	T	Accurate
Marker2301	Chr. 4	426111	G	G	A	G	A	Accurate
Marker2302	Chr. 4	429664	A	A	G	A	G	Accurate
Marker2303	Chr. 4	429851	A	A	G	A	G	Accurate
Marker2304	Chr. 4	430123	C	C	T	C	T	Accurate
Marker2305	Chr. 4	430548	T	T	C	T	C	Accurate
Marker2307	Chr. 4	450339	T	T	C	T	C	Accurate
Marker2308	Chr. 4	450534	C	C	T	C	T	Accurate
Marker2310	Chr. 4	454019	G	G	A	G	A	Accurate
Marker2311	Chr. 4	455727	T	T	G	T	G	Accurate
Marker2314	Chr. 4	554392	G	G	A	G	A	Accurate
Marker2315	Chr. 4	557991	A	A	G	A	G	Accurate

Marker2316	Chr. 4	558199	T	T	C	T	C	Accurate
Marker2317	Chr. 4	558204	G	G	A	G	A	Accurate
Marker2318	Chr. 4	558218	A	A	G	A	G	Accurate
Marker2321	Chr. 4	562418	T	T	C	T	C	Accurate
Marker2322	Chr. 4	562567	T	T	G	T	G	Accurate
Marker2323	Chr. 4	565632	C	C	T	C	T	Accurate
Marker2332	Chr. 4	2457333	A	G	A	G	A	Accurate
Marker2337	Chr. 4	3143467	T	C	T	C	T	Accurate
Marker2338	Chr. 4	3189879	A	G	A	G	A	Accurate
Marker2339	Chr. 4	3189919	G	A	G	A	G	Accurate
Marker2342	Chr. 4	3197202	C	A	C	A	C	Accurate
Marker2344	Chr. 4	3197496	A	G	A	G	A	Accurate
Marker2346	Chr. 4	3199064	G	A	G	A	G	Accurate
Marker2349	Chr. 4	3237519	T	C	T	C	T	Accurate
Marker2350	Chr. 4	3237546	A	G	A	G	A	Accurate
Marker2352	Chr. 4	3243966	T	G	T	G	T	Accurate
Marker2358	Chr. 4	3298819	G	A	G	A	G	Accurate
Marker2359	Chr. 4	3298822	G	T	G	K	G	Uncertain
Marker2360	Chr. 4	3305782	G	A	G	A	G	Accurate
Marker2361	Chr. 4	3308955	G	A	G	A	G	Accurate
Marker2363	Chr. 4	3330773	G	A	G	A	G	Accurate
Marker2366	Chr. 4	3396084	A	G	A	G	A	Accurate
Marker2367	Chr. 4	3418811	T	C	T	C	T	Accurate
Marker2371	Chr. 4	3820663	G	A	G	A	G	Accurate
Marker2373	Chr. 4	4615898	T	G	T	G	T	Accurate
Marker2377	Chr. 4	4674645	G	A	G	A	G	Accurate
Marker2378	Chr. 4	4686339	G	A	G	A	G	Accurate
Marker2379	Chr. 4	4695308	C	A	C	A	C	Accurate
Marker2380	Chr. 4	4695462	T	C	T	C	T	Accurate
Marker2385	Chr. 4	4773577	C	G	C	G	C	Accurate
Marker2387	Chr. 4	4817230	A	G	A	G	A	Accurate
Marker2389	Chr. 4	4830461	T	G	T	G	T	Accurate
Marker2390	Chr. 4	4830667	C	T	C	T	C	Accurate
Marker2393	Chr. 4	5627574	A	C	A	C	A	Accurate
Marker2394	Chr. 4	5627948	A	G	A	G	A	Accurate
Marker2395	Chr. 4	5629864	G	A	G	R	G	Uncertain
Marker2397	Chr. 4	5653168	G	A	G	A	G	Accurate
Marker2398	Chr. 4	5667655	T	C	T	C	T	Accurate
Marker2400	Chr. 4	5712339	A	T	A	T	A	Accurate
Marker2401	Chr. 4	5734069	A	A	C	A	G	Inconsistent
Marker2402	Chr. 4	5736473	C	T	C	T	C	Accurate
Marker2411	Chr. 4	5812014	C	T	C	T	C	Accurate
Marker2415	Chr. 4	7518627	T	C	T	C	T	Accurate
Marker2416	Chr. 4	7547462	T	C	T	C	T	Accurate
Marker2418	Chr. 4	7567289	T	A	T	A	T	Accurate
Marker2419	Chr. 4	7567497	G	A	G	A	G	Accurate
Marker2420	Chr. 4	7573254	A	G	A	G	A	Accurate

Marker2423	Chr. 4	7680060	G	A	G	A	G	Accurate
Marker2424	Chr. 4	7681606	G	A	G	A	G	Accurate
Marker2425	Chr. 4	7681636	C	T	C	T	C	Accurate
Marker2427	Chr. 4	7682305	T	C	T	C	T	Accurate
Marker2429	Chr. 4	7707335	T	C	T	C	T	Accurate
Marker2430	Chr. 4	7707382	A	C	A	C	A	Accurate
Marker2437	Chr. 4	7821562	A	G	A	G	A	Accurate
Marker2454	Chr. 4	7920494	C	T	C	T	C	Accurate
Marker2456	Chr. 4	7968848	A	G	A	R	A	Uncertain
Marker2457	Chr. 4	7968886	A	G	A	R	A	Uncertain
Marker2458	Chr. 4	8016869	A	G	A	G	A	Accurate
Marker2460	Chr. 4	8027310	C	A	C	A	C	Accurate
Marker2462	Chr. 4	8029062	T	C	T	C	T	Accurate
Marker2464	Chr. 4	8073317	T	C	T	C	T	Accurate
Marker2465	Chr. 4	8074813	T	C	T	C	T	Accurate
Marker2468	Chr. 4	8079507	A	G	A	G	A	Accurate
Marker2480	Chr. 4	9269207	A	A	G	A	G	Accurate
Marker2481	Chr. 4	9269212	A	A	G	A	G	Accurate
Marker2482	Chr. 4	9269237	A	A	G	A	G	Accurate
Marker2483	Chr. 4	9269265	A	A	G	A	G	Accurate
Marker2484	Chr. 4	9276648	G	G	A	G	A	Accurate
Marker2485	Chr. 4	9278740	T	T	C	T	C	Accurate
Marker2486	Chr. 4	9278745	A	A	G	A	G	Accurate
Marker2487	Chr. 4	9296201	G	G	C	G	C	Accurate
Marker2488	Chr. 4	9296348	T	T	A	T	A	Accurate
Marker2489	Chr. 4	9304135	T	T	C	T	C	Accurate
Marker2490	Chr. 4	9355535	T	T	C	T	C	Accurate
Marker2491	Chr. 4	9358109	T	T	C	T	C	Accurate
Marker2494	Chr. 4	9651075	G	A	G	A	G	Accurate
Marker2495	Chr. 4	9656250	C	T	C	T	C	Accurate
Marker2496	Chr. 4	9656433	A	G	A	G	A	Accurate
Marker2500	Chr. 4	9824082	G	A	G	A	G	Accurate
Marker2502	Chr. 4	9842501	T	G	T	G	T	Accurate
Marker2503	Chr. 4	9849553	A	C	A	C	A	Accurate
Marker2505	Chr. 4	9868023	T	G	T	G	T	Accurate
Marker2507	Chr. 4	9873756	T	T	C	T	C	Accurate
Marker2509	Chr. 4	10036612	A	A	T	A	T	Accurate
Marker2510	Chr. 4	10036623	T	T	G	T	G	Accurate
Marker2511	Chr. 4	10051791	G	G	A	G	A	Accurate
Marker2512	Chr. 4	10059208	T	T	C	T	C	Accurate
Marker2514	Chr. 4	10173843	G	G	A	G	A	Accurate
Marker2516	Chr. 4	10191329	T	T	C	T	C	Accurate
Marker2517	Chr. 4	10198281	T	T	C	T	C	Accurate
Marker2519	Chr. 4	11905288	A	C	A	C	A	Accurate
Marker2520	Chr. 4	11945538	G	A	G	A	G	Accurate
Marker2524	Chr. 4	12070933	A	T	A	T	A	Accurate
Marker2525	Chr. 4	12074626	A	C	A	C	A	Accurate

Marker2528	Chr. 4	12105851	T	C	T	C	T	Accurate
Marker2529	Chr. 4	12105886	C	T	C	T	C	Accurate
Marker2532	Chr. 4	12123504	T	C	T	C	T	Accurate
Marker2535	Chr. 4	12170794	A	G	A	G	A	Accurate
Marker2537	Chr. 4	12229375	G	C	G	C	G	Accurate
Marker2542	Chr. 4	12422088	A	A	G	A	G	Accurate
Marker2545	Chr. 4	12997481	A	G	A	G	A	Accurate
Marker2551	Chr. 4	13695317	A	G	A	G	A	Accurate
Marker2552	Chr. 4	13697007	C	T	C	T	C	Accurate
Marker2553	Chr. 4	13710961	C	A	C	A	C	Accurate
Marker2555	Chr. 4	14141648	T	C	T	C	T	Accurate
Marker2566	Chr. 4	15345161	C	C	T	C	T	Accurate
Marker2571	Chr. 4	15564681	G	G	A	G	A	Accurate
Marker2573	Chr. 4	15564784	T	T	C	T	C	Accurate
Marker2577	Chr. 4	15587198	C	C	T	C	T	Accurate
Marker2578	Chr. 4	15696392	C	C	T	N	T	Uncertain
Marker2581	Chr. 4	15978920	A	A	T	A	T	Accurate
Marker2583	Chr. 4	15984139	A	A	C	A	C	Accurate
Marker2584	Chr. 4	16262209	G	G	A	G	A	Accurate
Marker2585	Chr. 4	16262210	A	A	G	A	G	Accurate
Marker2586	Chr. 4	16262714	A	A	C	A	C	Accurate
Marker2587	Chr. 4	16262716	G	G	A	G	A	Accurate
Marker2594	Chr. 4	16294771	G	G	A	G	A	Accurate
Marker2596	Chr. 4	16295229	A	A	G	A	G	Accurate
Marker2605	Chr. 4	17640628	C	C	A	C	A	Accurate
Marker2606	Chr. 4	17640646	T	T	C	T	C	Accurate
Marker2607	Chr. 4	17648271	A	A	C	A	C	Accurate
Marker2609	Chr. 4	17649706	A	A	T	A	T	Accurate
Marker2612	Chr. 4	18144949	C	C	A	C	A	Accurate
Marker2613	Chr. 4	18144950	A	A	G	A	G	Accurate
Marker2614	Chr. 4	18147370	G	T	G	T	G	Accurate
Marker2615	Chr. 4	18147385	A	C	A	C	A	Accurate
Marker2619	Chr. 4	18409333	C	C	T	C	T	Accurate
Marker2620	Chr. 4	18409579	T	T	C	T	C	Accurate
Marker2621	Chr. 4	18409598	G	G	C	G	C	Accurate
Marker2623	Chr. 4	18409807	T	T	C	T	C	Accurate
Marker2624	Chr. 4	18412508	A	A	G	A	G	Accurate
Marker2626	Chr. 4	18509950	G	G	T	G	T	Accurate
Marker2631	Chr. 4	18685626	A	A	G	A	G	Accurate
Marker2632	Chr. 4	18692168	A	T	A	T	A	Accurate
Marker2634	Chr. 4	18754825	T	A	T	A	T	Accurate
Marker2636	Chr. 4	18758999	C	T	C	T	C	Accurate
Marker2638	Chr. 4	18788300	C	G	C	G	C	Accurate
Marker2639	Chr. 4	18799677	G	A	G	A	G	Accurate
Marker2641	Chr. 4	18808948	T	C	T	C	T	Accurate
Marker2642	Chr. 4	18809141	G	A	G	A	G	Accurate
Marker2646	Chr. 4	19061418	C	C	G	C	G	Accurate

Marker2649	Chr. 4	19217711	G	G	A	G	A	Accurate
Marker2650	Chr. 4	19222087	C	C	A	C	A	Accurate
Marker2655	Chr. 4	19308415	C	C	T	N	T	Uncertain
Marker2662	Chr. 4	19653293	A	A	G	A	G	Accurate
Marker2664	Chr. 4	19901228	T	T	A	T	A	Accurate
Marker2665	Chr. 4	19901421	T	T	C	T	C	Accurate
Marker2666	Chr. 4	19921519	A	A	C	A	C	Accurate
Marker2667	Chr. 4	19930849	T	T	G	T	G	Accurate
Marker2668	Chr. 4	19932438	A	A	G	A	G	Accurate
Marker2669	Chr. 4	19938307	C	C	T	C	T	Accurate
Marker2670	Chr. 4	20006201	G	G	A	G	A	Accurate
Marker2671	Chr. 4	20020382	G	G	A	G	A	Accurate
Marker2672	Chr. 4	20020439	C	C	T	C	T	Accurate
Marker2673	Chr. 4	20040012	A	A	T	A	T	Accurate
Marker2674	Chr. 4	20040033	T	T	G	T	G	Accurate
Marker2675	Chr. 4	20092152	C	C	T	C	T	Accurate
Marker2676	Chr. 4	20093812	C	C	A	C	A	Accurate
Marker2677	Chr. 4	20430671	G	A	G	A	G	Accurate
Marker2679	Chr. 4	20760931	C	C	T	C	T	Accurate
Marker2681	Chr. 4	20775667	T	T	C	N	C	Uncertain
Marker2684	Chr. 4	20904917	C	C	T	C	T	Accurate
Marker2687	Chr. 4	21851410	C	T	C	T	N	Uncertain
Marker2688	Chr. 4	21989239	T	T	C	T	C	Accurate
Marker2689	Chr. 4	22088935	T	A	T	A	T	Accurate
Marker2692	Chr. 4	22120756	T	T	C	T	C	Accurate
Marker2693	Chr. 4	22120774	A	A	G	A	G	Accurate
Marker2694	Chr. 4	22136827	C	C	T	C	T	Accurate
Marker2695	Chr. 4	22138065	T	T	G	T	G	Accurate
Marker2696	Chr. 4	22211588	T	T	A	N	A	Uncertain
Marker2698	Chr. 4	22226391	C	C	T	C	T	Accurate
Marker2699	Chr. 4	22241181	G	G	A	G	A	Accurate
Marker2703	Chr. 4	23122119	C	T	C	T	C	Accurate
Marker2704	Chr. 4	23122306	T	G	T	G	T	Accurate
Marker2709	Chr. 4	23650126	G	G	T	N	T	Uncertain
Marker2711	Chr. 4	23683705	G	G	C	G	C	Accurate
Marker2712	Chr. 4	23683722	A	A	G	A	G	Accurate
Marker2713	Chr. 4	23714478	G	G	A	G	A	Accurate
Marker2714	Chr. 4	23724773	C	C	T	C	T	Accurate
Marker2715	Chr. 4	23726298	T	T	C	T	C	Accurate
Marker2719	Chr. 4	23821340	C	C	A	C	A	Accurate
Marker2721	Chr. 4	23837105	C	C	T	C	T	Accurate
Marker2723	Chr. 4	23848571	G	A	G	A	N	Uncertain
Marker2729	Chr. 5	400981	T	T	C	T	C	Accurate
Marker2735	Chr. 5	421324	A	A	G	A	G	Accurate
Marker2739	Chr. 5	661773	T	C	T	C	T	Accurate
Marker2740	Chr. 5	669247	T	C	T	C	T	Accurate
Marker2741	Chr. 5	711433	G	A	G	A	G	Accurate

Marker2742	Chr. 5	1000493	C	C	T	C	T	Accurate
Marker2743	Chr. 5	1000506	G	G	A	G	A	Accurate
Marker2744	Chr. 5	1005857	A	A	G	A	G	Accurate
Marker2754	Chr. 5	1313738	G	A	G	A	G	Accurate
Marker2755	Chr. 5	1315478	A	G	A	G	A	Accurate
Marker2759	Chr. 5	1340551	A	G	A	G	A	Accurate
Marker2760	Chr. 5	1346558	A	G	A	G	A	Accurate
Marker2761	Chr. 5	1346600	T	C	T	C	T	Accurate
Marker2763	Chr. 5	1355086	A	G	A	G	A	Accurate
Marker2769	Chr. 5	1449118	G	A	G	A	G	Accurate
Marker2770	Chr. 5	1459437	G	T	G	T	G	Accurate
Marker2771	Chr. 5	1461502	C	T	C	T	C	Accurate
Marker2774	Chr. 5	1495920	C	G	C	G	C	Accurate
Marker2777	Chr. 5	1649977	C	C	G	C	G	Accurate
Marker2778	Chr. 5	1650589	C	C	T	C	Y	Uncertain
Marker2779	Chr. 5	1650861	A	A	C	N	C	Uncertain
Marker2785	Chr. 5	1652994	C	C	T	N	T	Uncertain
Marker2786	Chr. 5	1676635	C	T	C	T	C	Accurate
Marker2787	Chr. 5	1676661	A	C	A	C	A	Accurate
Marker2789	Chr. 5	1723800	A	G	A	G	N	Uncertain
Marker2791	Chr. 5	1765734	A	A	G	A	G	Accurate
Marker2796	Chr. 5	1877713	A	T	A	T	A	Accurate
Marker2797	Chr. 5	1883517	A	T	A	T	A	Accurate
Marker2800	Chr. 5	1940096	T	C	T	C	T	Accurate
Marker2801	Chr. 5	2418175	C	C	T	C	T	Accurate
Marker2802	Chr. 5	2418214	G	G	A	G	A	Accurate
Marker2803	Chr. 5	2418332	G	G	A	G	A	Accurate
Marker2807	Chr. 5	2618946	A	C	A	C	A	Accurate
Marker2808	Chr. 5	2621406	A	G	A	G	A	Accurate
Marker2809	Chr. 5	2623508	A	T	A	T	A	Accurate
Marker2813	Chr. 5	2930524	G	A	G	A	G	Accurate
Marker2818	Chr. 5	3564894	A	G	A	G	A	Accurate
Marker2820	Chr. 5	3792833	G	C	G	C	N	Uncertain
Marker2822	Chr. 5	3846709	T	C	T	C	T	Accurate
Marker2823	Chr. 5	4567444	G	A	G	A	G	Accurate
Marker2826	Chr. 5	4567694	C	T	C	T	C	Accurate
Marker2828	Chr. 5	4567802	T	C	T	C	T	Accurate
Marker2829	Chr. 5	4950466	A	C	A	C	A	Accurate
Marker2835	Chr. 5	5053430	T	A	T	A	T	Accurate
Marker2844	Chr. 5	5152867	T	C	T	C	T	Accurate
Marker2849	Chr. 5	5182868	A	G	A	G	A	Accurate
Marker2853	Chr. 5	5455770	A	A	G	N	G	Uncertain
Marker2854	Chr. 5	5552068	A	A	G	A	G	Accurate
Marker2855	Chr. 5	5566722	C	C	T	C	T	Accurate
Marker2857	Chr. 5	5575079	T	A	T	A	T	Accurate
Marker2858	Chr. 5	5575362	G	A	G	A	G	Accurate
Marker2860	Chr. 5	5587725	A	G	A	G	A	Accurate

Marker2861	Chr. 5	5806536	T	C	T	C	T	Accurate
Marker2862	Chr. 5	5831826	T	C	T	C	T	Accurate
Marker2863	Chr. 5	5832914	C	A	C	A	C	Accurate
Marker2872	Chr. 5	8518588	C	C	T	C	T	Accurate
Marker2873	Chr. 5	8714610	C	T	C	T	C	Accurate
Marker2874	Chr. 5	8714614	T	G	T	G	T	Accurate
Marker2878	Chr. 5	8786645	G	G	A	N	A	Uncertain
Marker2885	Chr. 5	8855440	G	A	G	A	G	Accurate
Marker2886	Chr. 5	8855442	C	A	C	A	C	Accurate
Marker2888	Chr. 5	9243579	G	A	G	A	G	Accurate
Marker2889	Chr. 5	9243799	G	G	T	G	T	Accurate
Marker2890	Chr. 5	9263549	T	A	T	A	T	Accurate
Marker2891	Chr. 5	9335138	C	T	C	T	C	Accurate
Marker2892	Chr. 5	9478183	C	T	C	T	N	Uncertain
Marker2894	Chr. 5	9839466	G	A	G	A	G	Accurate
Marker2895	Chr. 5	9839673	T	G	T	G	T	Accurate
Marker2896	Chr. 5	9858632	A	G	A	G	A	Accurate
Marker2897	Chr. 5	9863249	G	A	G	A	G	Accurate
Marker2900	Chr. 5	9878080	G	G	T	G	T	Accurate
Marker2901	Chr. 5	9890376	G	A	G	A	G	Accurate
Marker2902	Chr. 5	9891977	C	T	C	T	C	Accurate
Marker2905	Chr. 5	9898808	C	G	C	G	C	Accurate
Marker2906	Chr. 5	9898810	A	C	A	C	A	Accurate
Marker2911	Chr. 5	9906923	G	A	G	A	G	Accurate
Marker2913	Chr. 5	10203444	G	A	G	A	G	Accurate
Marker2915	Chr. 5	10294398	C	C	T	C	T	Accurate
Marker2916	Chr. 5	10300368	C	C	T	C	T	Accurate
Marker2920	Chr. 5	10910840	T	T	C	T	C	Accurate
Marker2921	Chr. 5	10910862	T	T	C	T	C	Accurate
Marker2922	Chr. 5	10914406	C	C	T	C	T	Accurate
Marker2923	Chr. 5	10914594	T	T	C	T	C	Accurate
Marker2924	Chr. 5	10948125	C	A	C	A	C	Accurate
Marker2925	Chr. 5	10949136	T	C	T	C	T	Accurate
Marker2927	Chr. 5	11017195	C	T	C	T	C	Accurate
Marker2928	Chr. 5	11025292	A	C	A	C	A	Accurate
Marker2929	Chr. 5	11027172	G	A	G	A	G	Accurate
Marker2930	Chr. 5	11027217	T	C	T	C	T	Accurate
Marker2931	Chr. 5	11031407	C	T	C	T	C	Accurate
Marker2932	Chr. 5	11081732	T	C	T	C	T	Accurate
Marker2933	Chr. 5	11212903	T	G	T	G	T	Accurate
Marker2934	Chr. 5	11230757	G	A	G	A	G	Accurate
Marker2936	Chr. 5	11269621	T	T	A	T	A	Accurate
Marker2937	Chr. 5	11347503	T	T	C	T	C	Accurate
Marker2938	Chr. 5	11347795	G	G	A	G	A	Accurate
Marker2940	Chr. 5	11348882	A	A	G	A	G	Accurate
Marker2944	Chr. 5	11373880	G	G	T	G	T	Accurate
Marker2945	Chr. 5	11388950	C	C	T	C	T	Accurate

Marker2947	Chr. 5	11397064	G	T	G	T	G	Accurate
Marker2948	Chr. 5	11399498	C	T	C	T	C	Accurate
Marker2950	Chr. 5	11413237	G	G	A	G	A	Accurate
Marker2951	Chr. 5	11477633	A	G	A	G	A	Accurate
Marker2954	Chr. 5	11530761	C	A	C	A	C	Accurate
Marker2955	Chr. 5	11554368	G	A	G	A	G	Accurate
Marker2959	Chr. 5	11622665	C	C	T	C	T	Accurate
Marker2960	Chr. 5	11622786	A	A	G	A	G	Accurate
Marker2961	Chr. 5	11623012	A	A	T	A	T	Accurate
Marker2962	Chr. 5	11623238	T	T	C	T	C	Accurate
Marker2963	Chr. 5	11626850	T	T	A	T	A	Accurate
Marker2964	Chr. 5	11626950	T	T	G	T	G	Accurate
Marker2966	Chr. 5	11650896	C	C	T	C	T	Accurate
Marker2968	Chr. 5	11661053	C	C	T	C	T	Accurate
Marker2969	Chr. 5	11663383	G	G	A	G	A	Accurate
Marker2970	Chr. 5	11689425	C	C	A	C	A	Accurate
Marker2972	Chr. 5	11691526	A	A	T	A	T	Accurate
Marker2973	Chr. 5	11719762	A	C	A	C	A	Accurate
Marker2974	Chr. 5	11721204	T	T	C	N	C	Uncertain
Marker2976	Chr. 5	11883633	C	G	C	G	N	Uncertain
Marker2977	Chr. 5	11914923	T	T	C	T	C	Accurate
Marker2979	Chr. 5	12219870	T	A	T	A	T	Accurate
Marker2983	Chr. 5	12723352	G	T	G	T	G	Accurate
Marker2984	Chr. 5	12723360	T	C	T	C	T	Accurate
Marker2985	Chr. 5	12725292	C	A	C	A	C	Accurate
Marker2987	Chr. 5	12814560	G	A	G	A	G	Accurate
Marker2988	Chr. 5	12816921	A	C	A	C	A	Accurate
Marker2989	Chr. 5	12817054	G	A	G	A	G	Accurate
Marker2991	Chr. 5	12860311	T	T	C	T	C	Accurate
Marker2992	Chr. 5	12866558	C	T	C	T	C	Accurate
Marker2993	Chr. 5	12872912	C	C	T	C	T	Accurate
Marker2994	Chr. 5	12877191	G	G	A	G	A	Accurate
Marker2995	Chr. 5	12888120	C	C	T	C	T	Accurate
Marker2996	Chr. 5	12906885	C	C	T	C	T	Accurate
Marker2997	Chr. 5	12906920	A	A	G	A	G	Accurate
Marker3000	Chr. 5	13321122	G	A	G	A	G	Accurate
Marker3001	Chr. 5	13345580	C	T	C	T	C	Accurate
Marker3002	Chr. 5	13351299	T	C	T	C	T	Accurate
Marker3007	Chr. 5	15292422	G	A	G	A	G	Accurate
Marker3013	Chr. 5	15455894	G	A	G	A	G	Accurate
Marker3014	Chr. 5	15455916	C	T	C	T	C	Accurate
Marker3017	Chr. 5	15630986	G	A	G	A	G	Accurate
Marker3018	Chr. 5	15631159	T	T	C	T	C	Accurate
Marker3019	Chr. 5	15631205	C	C	A	C	A	Accurate
Marker3022	Chr. 5	15979072	T	T	C	T	C	Accurate
Marker3024	Chr. 5	16099903	T	T	C	N	C	Uncertain
Marker3025	Chr. 5	16099904	G	G	A	N	A	Uncertain

Marker3026	Chr. 5	16115072	T	T	A	T	A	Accurate
Marker3027	Chr. 5	16527392	C	C	A	C	A	Accurate
Marker3033	Chr. 5	16620285	G	G	A	G	A	Accurate
Marker3038	Chr. 5	16680441	T	T	G	T	G	Accurate
Marker3039	Chr. 5	16695864	G	G	A	G	A	Accurate
Marker3041	Chr. 5	16696553	G	G	A	G	A	Accurate
Marker3042	Chr. 5	16696580	A	A	G	A	G	Accurate
Marker3043	Chr. 5	16700930	A	A	G	A	G	Accurate
Marker3046	Chr. 5	16804882	C	T	C	T	C	Accurate
Marker3047	Chr. 5	16807890	C	A	C	A	C	Accurate
Marker3051	Chr. 5	16819724	C	T	C	T	C	Accurate
Marker3052	Chr. 5	16819839	C	T	C	T	C	Accurate
Marker3056	Chr. 5	16860405	C	C	T	C	Y	Uncertain
Marker3058	Chr. 5	16903885	T	G	T	G	T	Accurate
Marker3060	Chr. 5	17648074	A	G	A	G	A	Accurate
Marker3061	Chr. 5	17648188	C	T	C	T	C	Accurate
Marker3063	Chr. 5	19767327	T	C	T	C	T	Accurate
Marker3064	Chr. 5	20134749	C	T	C	T	C	Accurate
Marker3067	Chr. 5	20322340	C	T	C	T	C	Accurate
Marker3069	Chr. 5	20361325	T	C	T	C	T	Accurate
Marker3070	Chr. 5	20373077	C	T	C	T	C	Accurate
Marker3071	Chr. 5	20373252	C	A	C	A	C	Accurate
Marker3074	Chr. 5	20439211	T	C	T	C	T	Accurate
Marker3075	Chr. 5	20458439	G	A	G	A	G	Accurate
Marker3077	Chr. 5	20538003	T	G	T	G	T	Accurate
Marker3080	Chr. 5	20586791	A	T	A	T	A	Accurate
Marker3082	Chr. 5	21001265	A	T	A	T	A	Accurate
Marker3083	Chr. 5	21014908	T	C	T	C	T	Accurate
Marker3085	Chr. 5	21025448	A	T	A	T	A	Accurate
Marker3086	Chr. 5	21025478	C	T	C	T	C	Accurate
Marker3087	Chr. 5	21025523	T	C	T	C	T	Accurate
Marker3094	Chr. 5	21076865	G	C	G	C	G	Accurate
Marker3103	Chr. 5	21218977	C	T	C	T	C	Accurate
Marker3105	Chr. 5	21239204	G	A	G	A	G	Accurate
Marker3106	Chr. 5	21239500	T	C	T	C	T	Accurate
Marker3107	Chr. 5	21239607	G	A	G	A	G	Accurate
Marker3108	Chr. 5	21261840	G	A	G	A	G	Accurate
Marker3110	Chr. 5	21267749	C	T	C	T	C	Accurate
Marker3121	Chr. 5	21454511	T	C	T	C	N	Uncertain
Marker3122	Chr. 5	21454746	T	C	T	Y	T	Uncertain
Marker3124	Chr. 5	21504703	T	C	T	C	T	Accurate
Marker3125	Chr. 5	21563425	A	C	A	C	A	Accurate
Marker3127	Chr. 5	21569020	G	A	G	A	G	Accurate
Marker3128	Chr. 5	21569086	T	C	T	C	T	Accurate
Marker3131	Chr. 5	21650829	T	C	T	C	T	Accurate
Marker3132	Chr. 5	21699651	A	G	A	G	A	Accurate
Marker3135	Chr. 5	21746064	T	C	T	C	T	Accurate

Marker3136	Chr. 5	21747349	T	C	T	C	T	Accurate
Marker3137	Chr. 5	21748711	C	T	C	T	C	Accurate
Marker3138	Chr. 5	21748735	T	C	T	C	T	Accurate
Marker3139	Chr. 5	21753028	T	C	T	C	T	Accurate
Marker3140	Chr. 5	21896141	A	C	A	C	A	Accurate
Marker3141	Chr. 5	22243019	G	A	G	A	G	Accurate
Marker3142	Chr. 5	22247191	A	G	A	G	A	Accurate
Marker3143	Chr. 5	22257102	C	G	C	G	C	Accurate
Marker3144	Chr. 5	22257104	T	C	T	C	T	Accurate
Marker3145	Chr. 5	22300421	T	C	T	C	T	Accurate
Marker3147	Chr. 5	22317510	G	A	G	A	G	Accurate
Marker3148	Chr. 5	22317517	C	T	C	T	C	Accurate
Marker3149	Chr. 5	22318966	G	A	G	A	G	Accurate
Marker3150	Chr. 5	22318990	A	G	A	G	A	Accurate
Marker3153	Chr. 5	22352754	G	A	G	A	G	Accurate
Marker3155	Chr. 5	22375834	G	C	G	C	G	Accurate
Marker3156	Chr. 5	22586422	G	G	C	C	S	Inconsistent
Marker3158	Chr. 5	22708686	C	T	C	T	C	Accurate
Marker3159	Chr. 5	22710345	A	C	A	C	A	Accurate
Marker3161	Chr. 5	22719197	C	T	C	T	C	Accurate
Marker3162	Chr. 5	22743630	C	T	C	T	C	Accurate
Marker3163	Chr. 5	22743893	C	T	C	T	C	Accurate
Marker3170	Chr. 5	22968397	A	C	A	C	A	Accurate
Marker3173	Chr. 5	23216682	A	C	A	C	A	Accurate
Marker3174	Chr. 5	23249819	T	C	T	C	T	Accurate
Marker3182	Chr. 5	23498570	A	A	G	A	G	Accurate
Marker3183	Chr. 5	23555296	G	C	G	C	G	Accurate
Marker3184	Chr. 5	23555556	C	T	C	T	C	Accurate
Marker3185	Chr. 5	24611236	T	T	C	T	C	Accurate
Marker3186	Chr. 5	24611507	C	C	A	C	A	Accurate
Marker3187	Chr. 5	24625699	T	T	C	T	C	Accurate
Marker3188	Chr. 5	24625716	C	C	G	C	G	Accurate
Marker3189	Chr. 5	24625959	A	A	C	A	C	Accurate
Marker3190	Chr. 5	24630014	G	G	T	G	T	Accurate
Marker3191	Chr. 5	24630715	C	C	T	C	T	Accurate
Marker3196	Chr. 5	24780748	T	T	A	T	A	Accurate
Marker3198	Chr. 5	24809413	C	A	C	A	C	Accurate
Marker3199	Chr. 5	24832803	A	T	A	T	A	Accurate
Marker3200	Chr. 5	24843371	G	A	G	A	G	Accurate
Marker3201	Chr. 5	24886421	A	G	A	G	A	Accurate
Marker3202	Chr. 5	24900425	C	T	C	T	C	Accurate
Marker3203	Chr. 5	24900445	C	T	C	T	C	Accurate
Marker3204	Chr. 5	24900651	G	A	G	A	G	Accurate
Marker3205	Chr. 5	24900662	C	T	C	T	C	Accurate
Marker3206	Chr. 5	24928426	T	C	T	C	T	Accurate
Marker3208	Chr. 5	25072851	A	G	A	G	A	Accurate
Marker3209	Chr. 5	25088549	G	A	G	A	G	Accurate

Marker3210	Chr. 5	25449028	G	G	C	G	C	Accurate
Marker3213	Chr. 5	25767675	C	T	C	T	C	Accurate
Marker3214	Chr. 5	25768429	A	G	A	G	A	Accurate
Marker3215	Chr. 5	25768451	G	A	G	A	G	Accurate
Marker3218	Chr. 5	25847379	C	T	C	T	C	Accurate
Marker3221	Chr. 5	26018684	A	A	G	A	G	Accurate
Marker3222	Chr. 5	26019733	T	T	C	T	C	Accurate
Marker3226	Chr. 5	26123270	T	T	C	T	C	Accurate
Marker3229	Chr. 5	26159487	A	A	G	A	G	Accurate
Marker3230	Chr. 5	26159778	A	A	G	A	G	Accurate
Marker3231	Chr. 5	26159790	G	G	A	G	A	Accurate
Marker3232	Chr. 5	26159800	C	C	T	C	T	Accurate
Marker3233	Chr. 5	26162366	T	T	A	T	A	Accurate
Marker3234	Chr. 5	26169391	C	C	T	C	T	Accurate
Marker3235	Chr. 5	26169396	C	C	T	C	T	Accurate
Marker3236	Chr. 5	26174080	T	T	A	T	A	Accurate
Marker3239	Chr. 5	26217825	C	C	T	C	T	Accurate
Marker3240	Chr. 5	26334781	C	C	A	C	A	Accurate
Marker3241	Chr. 5	26334805	C	C	A	C	A	Accurate
Marker3242	Chr. 5	26350942	A	A	T	A	T	Accurate
Marker3243	Chr. 5	26372563	A	A	G	A	G	Accurate
Marker3247	Chr. 5	26373958	G	G	A	G	A	Accurate
Marker3248	Chr. 5	26373975	G	G	T	G	T	Accurate
Marker3249	Chr. 5	26374009	G	G	A	G	A	Accurate
Marker3251	Chr. 5	26420254	T	T	C	T	C	Accurate
Marker3252	Chr. 5	26421814	T	T	C	T	C	Accurate
Marker3253	Chr. 5	26428391	C	C	T	C	T	Accurate
Marker3255	Chr. 5	26444962	C	C	T	C	T	Accurate
Marker3258	Chr. 5	26452797	A	A	T	A	T	Accurate
Marker3259	Chr. 5	26454619	A	A	T	A	T	Accurate
Marker3260	Chr. 5	26476648	C	A	C	A	C	Accurate
Marker3265	Chr. 5	26668090	A	A	G	A	G	Accurate
Marker3266	Chr. 5	26671113	C	C	T	C	T	Accurate
Marker3267	Chr. 5	26687908	G	G	A	G	A	Accurate
Marker3268	Chr. 5	26711093	T	T	C	T	C	Accurate
Marker3269	Chr. 5	26762225	C	C	T	C	T	Accurate
Marker3270	Chr. 5	26762481	C	C	T	C	T	Accurate
Marker3273	Chr. 5	26797133	C	C	T	C	T	Accurate
Marker3276	Chr. 5	28285690	T	C	T	C	T	Accurate
Marker3277	Chr. 5	28396866	T	T	C	T	C	Accurate
Marker3278	Chr. 5	28505114	G	G	A	G	A	Accurate
Marker3279	Chr. 5	28532765	A	A	G	A	G	Accurate
Marker3280	Chr. 5	28558101	C	G	C	G	C	Accurate
Marker3281	Chr. 5	28577120	A	A	G	A	G	Accurate
Marker3282	Chr. 5	28577396	G	G	A	G	A	Accurate
Marker3283	Chr. 5	28577456	A	A	G	A	G	Accurate
Marker3284	Chr. 5	28581270	C	C	T	C	T	Accurate

Marker3285	Chr. 5	28582829	A	A	G	A	G	Accurate
Marker3286	Chr. 5	28658845	A	G	A	G	A	Accurate
Marker3288	Chr. 5	28680225	T	C	T	C	T	Accurate
Marker3295	Chr. 5	29592664	T	T	C	T	C	Accurate
Marker3296	Chr. 5	29592672	C	C	T	C	T	Accurate
Marker3302	Chr. 5	29610375	G	G	A	G	A	Accurate
Marker3303	Chr. 5	29612206	T	A	T	A	T	Accurate
Marker3312	Chr. 5	32248325	A	A	G	A	G	Accurate
Marker3314	Chr. 5	32323090	T	T	C	T	C	Accurate
Marker3316	Chr. 5	32431084	T	T	C	T	C	Accurate
Marker3318	Chr. 5	32442267	A	A	G	A	G	Accurate
Marker3319	Chr. 5	32442313	A	A	G	A	G	Accurate
Marker3321	Chr. 5	32483191	G	G	T	G	T	Accurate
Marker3323	Chr. 5	32642509	T	G	T	G	T	Accurate
Marker3324	Chr. 5	32649897	A	G	A	G	A	Accurate
Marker3327	Chr. 5	32805059	G	T	G	T	G	Accurate
Marker3328	Chr. 5	32805060	A	T	A	T	A	Accurate
Marker3329	Chr. 5	32808191	G	A	G	A	G	Accurate
Marker3330	Chr. 5	32808557	T	A	T	A	T	Accurate
Marker3333	Chr. 5	32994235	T	T	A	T	A	Accurate
Marker3334	Chr. 5	32994471	T	T	C	T	C	Accurate
Marker3335	Chr. 5	33009659	T	T	A	T	A	Accurate
Marker3336	Chr. 5	33012766	C	C	A	C	A	Accurate
Marker3337	Chr. 5	33013050	T	T	C	T	C	Accurate
Marker3338	Chr. 5	33013085	A	A	G	A	G	Accurate
Marker3341	Chr. 5	33033670	A	A	C	A	C	Accurate
Marker3344	Chr. 5	33054576	G	G	A	G	A	Accurate
Marker3345	Chr. 5	33054605	C	C	T	C	T	Accurate
Marker3346	Chr. 5	33054752	T	T	C	T	C	Accurate
Marker3347	Chr. 5	33054793	T	T	C	T	C	Accurate
Marker3349	Chr. 5	33083775	T	T	C	T	C	Accurate
Marker3350	Chr. 5	33085398	T	T	C	T	C	Accurate
Marker3354	Chr. 5	33161052	G	G	T	G	T	Accurate
Marker3361	Chr. 5	33325768	A	A	C	A	C	Accurate
Marker3362	Chr. 5	33338934	C	C	T	C	T	Accurate
Marker3364	Chr. 5	33413717	G	G	A	G	A	Accurate
Marker3366	Chr. 5	33527791	C	T	C	T	C	Accurate
Marker3367	Chr. 5	33527824	T	A	T	A	T	Accurate
Marker3368	Chr. 5	33527840	T	G	T	G	T	Accurate
Marker3369	Chr. 5	33533163	A	G	A	G	N	Uncertain
Marker3371	Chr. 5	33545675	G	A	G	A	G	Accurate
Marker3376	Chr. 5	33710528	C	T	C	T	C	Accurate
Marker3377	Chr. 5	33710545	C	T	C	T	C	Accurate
Marker3378	Chr. 5	33711405	A	G	A	G	A	Accurate
Marker3382	Chr. 6	166875	T	T	A	T	A	Accurate
Marker3384	Chr. 6	310613	G	A	G	A	G	Accurate
Marker3385	Chr. 6	607220	T	T	G	T	G	Accurate

Marker3386	Chr. 6	634435	T	T	A	T	A	Accurate
Marker3387	Chr. 6	644436	T	T	C	T	C	Accurate
Marker3389	Chr. 6	657296	C	C	T	C	T	Accurate
Marker3391	Chr. 6	715944	T	T	A	T	A	Accurate
Marker3392	Chr. 6	816150	A	T	A	T	A	Accurate
Marker3393	Chr. 6	816174	T	C	T	C	T	Accurate
Marker3399	Chr. 6	849911	G	A	G	A	G	Accurate
Marker3400	Chr. 6	947210	A	A	G	A	G	Accurate
Marker3402	Chr. 6	1189978	A	A	G	A	G	Accurate
Marker3403	Chr. 6	1222949	C	C	T	C	T	Accurate
Marker3404	Chr. 6	1227356	T	T	A	T	A	Accurate
Marker3405	Chr. 6	1231912	G	G	A	G	A	Accurate
Marker3406	Chr. 6	1243765	T	T	G	T	G	Accurate
Marker3407	Chr. 6	1311353	A	C	A	C	A	Accurate
Marker3408	Chr. 6	1552219	G	G	C	G	C	Accurate
Marker3409	Chr. 6	1553416	A	A	G	A	G	Accurate
Marker3410	Chr. 6	1573813	C	T	C	T	C	Accurate
Marker3413	Chr. 6	1625394	T	T	C	T	C	Accurate
Marker3414	Chr. 6	1645751	T	T	C	T	C	Accurate
Marker3415	Chr. 6	1645755	A	A	G	A	G	Accurate
Marker3416	Chr. 6	1645770	C	C	T	C	T	Accurate
Marker3417	Chr. 6	1659200	G	G	A	G	A	Accurate
Marker3418	Chr. 6	1682234	C	C	A	C	A	Accurate
Marker3419	Chr. 6	1699746	T	T	A	T	A	Accurate
Marker3420	Chr. 6	1706374	A	A	G	A	G	Accurate
Marker3421	Chr. 6	1707705	C	C	G	C	G	Accurate
Marker3422	Chr. 6	1719867	G	G	A	G	A	Accurate
Marker3423	Chr. 6	1829115	G	G	A	G	A	Accurate
Marker3424	Chr. 6	1830430	T	T	C	T	C	Accurate
Marker3425	Chr. 6	1830968	C	C	T	C	T	Accurate
Marker3426	Chr. 6	1833108	C	C	T	C	T	Accurate
Marker3427	Chr. 6	1834121	T	T	C	T	C	Accurate
Marker3428	Chr. 6	1858718	T	T	G	T	G	Accurate
Marker3429	Chr. 6	1863100	T	T	A	T	A	Accurate
Marker3430	Chr. 6	1884742	G	G	T	G	T	Accurate
Marker3431	Chr. 6	1895351	A	A	G	A	G	Accurate
Marker3432	Chr. 6	1918252	C	C	G	C	G	Accurate
Marker3434	Chr. 6	1919152	C	C	A	C	A	Accurate
Marker3435	Chr. 6	1924502	C	C	T	C	T	Accurate
Marker3436	Chr. 6	1936042	A	A	G	A	G	Accurate
Marker3437	Chr. 6	1938442	G	G	A	G	A	Accurate
Marker3438	Chr. 6	1947260	C	C	T	C	T	Accurate
Marker3439	Chr. 6	1976569	C	C	T	C	T	Accurate
Marker3441	Chr. 6	1985562	C	C	T	N	T	Uncertain
Marker3442	Chr. 6	2000815	A	A	G	A	G	Accurate
Marker3443	Chr. 6	2003307	G	G	A	G	A	Accurate
Marker3444	Chr. 6	2003347	C	C	T	C	T	Accurate

Marker3445	Chr. 6	2003499	A	A	G	A	G	Accurate
Marker3447	Chr. 6	2410903	G	A	G	A	G	Accurate
Marker3448	Chr. 6	2467558	T	A	T	A	T	Accurate
Marker3449	Chr. 6	2570082	A	A	G	A	G	Accurate
Marker3450	Chr. 6	2655037	A	G	A	G	A	Accurate
Marker3451	Chr. 6	2684775	T	C	T	C	T	Accurate
Marker3452	Chr. 6	2800459	G	A	G	A	G	Accurate
Marker3453	Chr. 6	2813334	A	G	A	G	A	Accurate
Marker3455	Chr. 6	2816885	A	C	A	C	A	Accurate
Marker3456	Chr. 6	2832624	T	C	T	C	T	Accurate
Marker3457	Chr. 6	2841879	T	C	T	C	T	Accurate
Marker3458	Chr. 6	3306158	C	T	C	T	C	Accurate
Marker3460	Chr. 6	3337854	G	A	G	A	G	Accurate
Marker3461	Chr. 6	3347464	C	G	C	G	C	Accurate
Marker3464	Chr. 6	3858258	A	A	C	A	C	Accurate
Marker3466	Chr. 6	3906184	G	G	A	G	A	Accurate
Marker3467	Chr. 6	3906185	A	A	G	A	G	Accurate
Marker3468	Chr. 6	3906194	A	A	G	A	G	Accurate
Marker3470	Chr. 6	4092379	T	T	C	T	C	Accurate
Marker3472	Chr. 6	4377703	C	C	G	C	G	Accurate
Marker3473	Chr. 6	4380141	G	G	C	G	C	Accurate
Marker3475	Chr. 6	4387929	G	G	A	G	A	Accurate
Marker3476	Chr. 6	4388561	T	T	C	T	C	Accurate
Marker3478	Chr. 6	4403714	C	C	G	C	G	Accurate
Marker3481	Chr. 6	4404257	C	C	T	C	T	Accurate
Marker3482	Chr. 6	4405257	T	T	A	T	A	Accurate
Marker3483	Chr. 6	4405261	A	A	G	A	G	Accurate
Marker3484	Chr. 6	4405613	C	C	G	C	G	Accurate
Marker3487	Chr. 6	4607610	T	T	C	T	C	Accurate
Marker3494	Chr. 6	4703902	T	T	A	T	A	Accurate
Marker3496	Chr. 6	4762619	G	G	A	G	A	Accurate
Marker3498	Chr. 6	4782631	A	A	G	A	G	Accurate
Marker3500	Chr. 6	4832304	C	C	T	C	T	Accurate
Marker3503	Chr. 6	4956962	T	C	T	C	T	Accurate
Marker3504	Chr. 6	4956967	G	C	G	C	G	Accurate
Marker3506	Chr. 6	4959631	T	C	T	C	T	Accurate
Marker3507	Chr. 6	4959632	A	G	A	G	A	Accurate
Marker3508	Chr. 6	4999628	T	C	T	C	T	Accurate
Marker3512	Chr. 6	5071163	T	T	C	T	C	Accurate
Marker3517	Chr. 6	5080715	T	T	G	T	G	Accurate
Marker3518	Chr. 6	5085271	T	T	G	T	G	Accurate
Marker3521	Chr. 6	5805721	G	A	G	A	G	Accurate
Marker3522	Chr. 6	5826951	A	T	A	T	A	Accurate
Marker3525	Chr. 6	6396048	A	A	G	A	G	Accurate
Marker3526	Chr. 6	6396363	C	C	T	C	T	Accurate
Marker3527	Chr. 6	6400039	G	G	C	G	C	Accurate
Marker3528	Chr. 6	6406040	A	A	G	A	G	Accurate

Marker3529	Chr. 6	6406449	A	A	G	A	G	Accurate
Marker3530	Chr. 6	6407320	C	C	T	C	T	Accurate
Marker3531	Chr. 6	6407372	A	A	G	A	G	Accurate
Marker3532	Chr. 6	6424518	G	G	A	G	A	Accurate
Marker3533	Chr. 6	6424728	A	A	G	N	G	Uncertain
Marker3534	Chr. 6	6428943	T	T	C	T	C	Accurate
Marker3537	Chr. 6	6516657	G	A	G	R	G	Uncertain
Marker3538	Chr. 6	6518597	G	A	G	A	G	Accurate
Marker3539	Chr. 6	6518825	A	G	A	G	A	Accurate
Marker3540	Chr. 6	6518858	C	T	C	T	C	Accurate
Marker3541	Chr. 6	6527714	C	T	C	T	C	Accurate
Marker3542	Chr. 6	6527749	G	A	G	A	G	Accurate
Marker3545	Chr. 6	6680322	G	A	G	A	G	Accurate
Marker3546	Chr. 6	6683083	T	C	T	C	T	Accurate
Marker3547	Chr. 6	6690829	T	C	T	C	T	Accurate
Marker3548	Chr. 6	6742168	G	A	G	A	G	Accurate
Marker3549	Chr. 6	6742948	G	A	G	A	G	Accurate
Marker3550	Chr. 6	6806779	G	A	G	A	G	Accurate
Marker3551	Chr. 6	6815478	A	G	A	G	A	Accurate
Marker3552	Chr. 6	6835146	C	T	C	T	C	Accurate
Marker3553	Chr. 6	6835384	A	G	A	G	A	Accurate
Marker3554	Chr. 6	6864975	C	T	C	T	C	Accurate
Marker3555	Chr. 6	6870313	A	G	A	G	A	Accurate
Marker3557	Chr. 6	6901699	G	A	G	A	G	Accurate
Marker3558	Chr. 6	6903223	C	T	C	T	C	Accurate
Marker3559	Chr. 6	6956146	A	T	A	T	A	Accurate
Marker3560	Chr. 6	6958189	T	C	T	C	T	Accurate
Marker3561	Chr. 6	6980218	T	C	T	C	T	Accurate
Marker3564	Chr. 6	7011245	C	T	C	T	C	Accurate
Marker3565	Chr. 6	7125862	T	C	T	C	T	Accurate
Marker3568	Chr. 6	7129751	C	A	C	A	C	Accurate
Marker3573	Chr. 6	7297624	C	T	C	T	C	Accurate
Marker3574	Chr. 6	7297630	A	G	A	G	A	Accurate
Marker3575	Chr. 6	7300055	C	G	C	G	C	Accurate
Marker3579	Chr. 6	7464315	C	C	T	C	T	Accurate
Marker3580	Chr. 6	7468696	T	T	G	T	G	Accurate
Marker3581	Chr. 6	7634107	T	A	T	A	T	Accurate
Marker3586	Chr. 6	8294619	G	T	G	T	G	Accurate
Marker3591	Chr. 6	8664118	C	T	C	T	C	Accurate
Marker3592	Chr. 6	8697964	C	T	C	T	C	Accurate
Marker3593	Chr. 6	8717275	A	G	A	G	A	Accurate
Marker3594	Chr. 6	8717276	A	G	A	G	A	Accurate
Marker3595	Chr. 6	8752522	G	T	G	T	G	Accurate
Marker3596	Chr. 6	8766782	T	C	T	C	T	Accurate
Marker3597	Chr. 6	8766813	C	T	C	T	C	Accurate
Marker3600	Chr. 6	8798535	C	A	C	A	C	Accurate
Marker3601	Chr. 6	8798591	G	T	G	T	G	Accurate

Marker3602	Chr. 6	8942529	C	T	C	T	C	Accurate
Marker3604	Chr. 6	9019742	C	C	T	C	T	Accurate
Marker3606	Chr. 6	9075141	G	G	A	G	A	Accurate
Marker3608	Chr. 6	9129794	A	G	A	G	A	Accurate
Marker3609	Chr. 6	9137026	T	T	G	T	G	Accurate
Marker3613	Chr. 6	9161558	C	T	C	T	C	Accurate
Marker3614	Chr. 6	9279956	G	C	G	C	G	Accurate
Marker3615	Chr. 6	9288811	A	G	A	G	A	Accurate
Marker3617	Chr. 6	9308537	G	A	G	A	G	Accurate
Marker3618	Chr. 6	9308731	C	T	C	T	C	Accurate
Marker3620	Chr. 6	9328916	C	T	C	T	C	Accurate
Marker3622	Chr. 6	9421914	T	T	C	T	C	Accurate
Marker3629	Chr. 6	9470104	C	C	T	C	T	Accurate
Marker3630	Chr. 6	9475534	G	G	A	G	A	Accurate
Marker3631	Chr. 6	9479235	A	A	G	A	G	Accurate
Marker3633	Chr. 6	9508338	T	G	T	G	T	Accurate
Marker3636	Chr. 6	9573222	T	C	T	C	T	Accurate
Marker3637	Chr. 6	9578110	G	G	T	G	T	Accurate
Marker3639	Chr. 6	9583164	C	A	C	A	C	Accurate
Marker3641	Chr. 6	9641609	C	C	G	C	G	Accurate
Marker3642	Chr. 6	9644237	C	T	C	T	C	Accurate
Marker3643	Chr. 6	9660431	G	T	G	T	G	Accurate
Marker3644	Chr. 6	9736333	C	A	C	A	C	Accurate
Marker3645	Chr. 6	9807459	T	C	T	C	T	Accurate
Marker3647	Chr. 6	9824104	C	A	C	A	C	Accurate
Marker3648	Chr. 6	9830277	T	C	T	C	T	Accurate
Marker3649	Chr. 6	9830449	G	A	G	A	G	Accurate
Marker3650	Chr. 6	9836422	C	T	C	T	C	Accurate
Marker3651	Chr. 6	9850822	G	C	G	C	G	Accurate
Marker3652	Chr. 6	9859147	C	T	C	T	C	Accurate
Marker3653	Chr. 6	9859154	G	T	G	T	G	Accurate
Marker3654	Chr. 6	9859187	C	T	C	T	C	Accurate
Marker3656	Chr. 6	9901710	G	A	G	A	G	Accurate
Marker3657	Chr. 6	9913588	C	T	C	T	C	Accurate
Marker3658	Chr. 6	9931632	A	G	A	G	A	Accurate
Marker3659	Chr. 6	9949640	G	A	G	A	G	Accurate
Marker3661	Chr. 6	9988201	C	T	C	T	C	Accurate
Marker3662	Chr. 6	9994144	C	T	C	T	C	Accurate
Marker3663	Chr. 6	10024379	A	G	A	G	A	Accurate
Marker3664	Chr. 6	10039260	T	C	T	C	T	Accurate
Marker3667	Chr. 6	10064159	G	T	G	T	G	Accurate
Marker3668	Chr. 6	10079736	G	A	G	A	G	Accurate
Marker3669	Chr. 6	10232286	A	A	T	A	T	Accurate
Marker3675	Chr. 6	10514797	T	T	C	T	Y	Uncertain
Marker3680	Chr. 6	10561187	A	G	A	G	A	Accurate
Marker3681	Chr. 6	10561218	T	C	T	C	T	Accurate
Marker3682	Chr. 6	10574232	C	T	C	T	C	Accurate

Marker3683	Chr. 6	10575014	C	T	C	T	C	Accurate
Marker3685	Chr. 6	10592350	A	G	A	G	A	Accurate
Marker3687	Chr. 6	10631305	C	G	C	G	C	Accurate
Marker3688	Chr. 6	10632446	A	G	A	G	A	Accurate
Marker3690	Chr. 6	10637736	G	A	G	A	G	Accurate
Marker3691	Chr. 6	10637976	A	C	A	C	A	Accurate
Marker3692	Chr. 6	10643346	A	C	A	C	A	Accurate
Marker3693	Chr. 6	10643369	T	A	T	A	T	Accurate
Marker3694	Chr. 6	10643400	T	C	T	C	T	Accurate
Marker3696	Chr. 6	10672797	C	T	C	Y	C	Uncertain
Marker3702	Chr. 6	10863067	C	C	T	C	T	Accurate
Marker3703	Chr. 6	10863083	C	C	T	C	T	Accurate
Marker3706	Chr. 6	11138648	T	C	T	C	T	Accurate
Marker3709	Chr. 6	11288083	C	A	C	A	C	Accurate
Marker3710	Chr. 6	11306435	T	G	T	G	T	Accurate
Marker3711	Chr. 6	11338078	T	G	T	G	T	Accurate
Marker3712	Chr. 6	11354832	A	G	A	G	A	Accurate
Marker3713	Chr. 6	11354887	C	A	C	A	C	Accurate
Marker3714	Chr. 6	11359313	G	A	G	A	G	Accurate
Marker3716	Chr. 6	11437165	T	C	T	Y	T	Uncertain
Marker3717	Chr. 6	11437419	G	A	G	A	G	Accurate
Marker3718	Chr. 6	11437439	C	T	C	T	C	Accurate
Marker3719	Chr. 6	11437593	G	A	G	A	G	Accurate
Marker3720	Chr. 6	11437763	A	G	A	G	A	Accurate
Marker3721	Chr. 6	11437780	T	C	T	C	T	Accurate
Marker3722	Chr. 6	11444438	A	G	A	G	A	Accurate
Marker3723	Chr. 6	11451129	G	A	G	A	G	Accurate
Marker3724	Chr. 6	11453829	C	A	C	A	C	Accurate
Marker3725	Chr. 6	11464463	A	T	A	T	A	Accurate
Marker3726	Chr. 6	11475769	C	T	C	T	C	Accurate
Marker3727	Chr. 6	11476840	A	G	A	G	A	Accurate
Marker3728	Chr. 6	11477095	G	G	A	G	A	Accurate
Marker3731	Chr. 6	11510532	C	T	C	T	C	Accurate
Marker3732	Chr. 6	11511919	G	A	G	A	G	Accurate
Marker3733	Chr. 6	11519458	T	C	T	C	T	Accurate
Marker3736	Chr. 6	11584432	G	A	G	A	G	Accurate
Marker3737	Chr. 6	11584584	G	A	G	A	G	Accurate
Marker3738	Chr. 6	11612241	T	C	T	C	T	Accurate
Marker3739	Chr. 6	11612474	T	C	T	C	T	Accurate
Marker3740	Chr. 6	11612477	T	C	T	C	T	Accurate
Marker3741	Chr. 6	11624826	A	G	A	G	A	Accurate
Marker3742	Chr. 6	11635357	A	G	A	G	A	Accurate
Marker3747	Chr. 6	11702305	C	T	C	T	C	Accurate
Marker3748	Chr. 6	11735300	A	T	A	T	A	Accurate
Marker3750	Chr. 6	11741629	C	T	C	Y	C	Uncertain
Marker3751	Chr. 6	11819612	T	C	T	C	T	Accurate
Marker3752	Chr. 6	11819647	T	C	T	C	T	Accurate

Marker3753	Chr. 6	11944966	T	T	G	G	G	Inconsistent
Marker3755	Chr. 6	11967049	A	C	A	C	A	Accurate
Marker3757	Chr. 6	12047745	C	T	C	T	C	Accurate
Marker3758	Chr. 6	12052467	A	G	A	G	A	Accurate
Marker3759	Chr. 6	12053798	G	A	G	A	G	Accurate
Marker3760	Chr. 6	12054032	A	G	A	G	A	Accurate
Marker3762	Chr. 6	12085405	C	C	G	C	G	Accurate
Marker3763	Chr. 6	12092281	G	G	A	G	A	Accurate
Marker3764	Chr. 6	12098734	A	A	G	A	G	Accurate
Marker3766	Chr. 6	12099034	G	G	A	G	A	Accurate
Marker3769	Chr. 6	12239973	T	T	C	T	C	Accurate
Marker3770	Chr. 6	12244683	A	A	G	A	G	Accurate
Marker3771	Chr. 6	12257501	G	G	A	G	A	Accurate
Marker3773	Chr. 6	12275812	C	T	C	T	C	Accurate
Marker3774	Chr. 6	12276053	A	T	A	T	A	Accurate
Marker3775	Chr. 6	12281865	T	C	T	C	T	Accurate
Marker3776	Chr. 6	12295154	T	C	T	C	T	Accurate
Marker3778	Chr. 6	12643206	C	T	C	T	C	Accurate
Marker3779	Chr. 6	12655743	T	C	T	C	T	Accurate
Marker3781	Chr. 6	12655984	C	G	C	G	C	Accurate
Marker3783	Chr. 6	12656191	C	T	C	T	C	Accurate
Marker3784	Chr. 6	12659113	A	G	A	G	R	Uncertain
Marker3788	Chr. 6	12700168	G	C	G	C	G	Accurate
Marker3789	Chr. 6	12700176	G	A	G	A	G	Accurate
Marker3790	Chr. 6	12739072	T	C	T	C	T	Accurate
Marker3791	Chr. 6	12805038	G	C	G	C	G	Accurate
Marker3792	Chr. 6	12811474	C	T	C	T	N	Uncertain
Marker3793	Chr. 6	12818100	T	T	A	T	A	Accurate
Marker3795	Chr. 6	12965198	C	T	C	T	C	Accurate
Marker3796	Chr. 6	12966029	G	A	G	A	G	Accurate
Marker3798	Chr. 6	13000185	G	A	G	A	G	Accurate
Marker3799	Chr. 6	13015261	T	G	T	G	T	Accurate
Marker3800	Chr. 6	13015522	A	G	A	G	A	Accurate
Marker3801	Chr. 6	13033684	T	C	T	C	T	Accurate
Marker3802	Chr. 6	13040595	G	C	G	C	G	Accurate
Marker3803	Chr. 6	13040849	C	T	C	T	C	Accurate
Marker3804	Chr. 6	13050125	G	A	G	A	G	Accurate
Marker3805	Chr. 6	13088920	T	C	T	C	T	Accurate
Marker3806	Chr. 6	13090152	C	G	C	G	C	Accurate
Marker3808	Chr. 6	14717398	G	G	A	G	A	Accurate
Marker3809	Chr. 6	14717579	A	A	G	A	G	Accurate
Marker3810	Chr. 6	14817743	T	T	C	C	C	Inconsistent
Marker3815	Chr. 6	15364926	T	T	C	T	C	Accurate
Marker3816	Chr. 6	15629880	A	A	G	A	G	Accurate
Marker3817	Chr. 6	15651624	T	T	C	T	C	Accurate
Marker3818	Chr. 6	15657155	T	T	G	T	G	Accurate
Marker3819	Chr. 6	15848571	T	T	C	T	C	Accurate

Marker3820	Chr. 6	15898118	C	C	T	C	T	Accurate
Marker3822	Chr. 6	16075709	C	C	G	C	G	Accurate
Marker3823	Chr. 6	16128367	A	A	G	A	G	Accurate
Marker3824	Chr. 6	16144104	A	A	G	A	G	Accurate
Marker3825	Chr. 6	16171231	C	C	T	C	T	Accurate
Marker3826	Chr. 6	16275526	C	C	T	C	T	Accurate
Marker3827	Chr. 6	16333004	G	G	A	G	A	Accurate
Marker3828	Chr. 6	16339883	T	T	C	T	C	Accurate
Marker3829	Chr. 6	16618555	A	A	T	A	T	Accurate
Marker3830	Chr. 6	16669992	G	G	T	G	T	Accurate
Marker3832	Chr. 6	16698604	A	T	A	T	A	Accurate
Marker3833	Chr. 6	16727959	T	T	C	T	C	Accurate
Marker3835	Chr. 6	16787553	A	A	G	A	G	Accurate
Marker3837	Chr. 6	16824840	T	T	G	T	G	Accurate
Marker3838	Chr. 6	16830138	G	G	T	G	T	Accurate
Marker3840	Chr. 6	16859716	T	T	C	T	C	Accurate
Marker3841	Chr. 6	16873753	A	A	G	A	G	Accurate
Marker3842	Chr. 6	16873767	A	A	G	A	G	Accurate
Marker3843	Chr. 6	16874005	T	T	C	T	Y	Uncertain
Marker3846	Chr. 6	17582240	A	A	G	A	G	Accurate
Marker3847	Chr. 6	17586855	G	G	T	G	T	Accurate
Marker3848	Chr. 6	17619891	G	G	A	G	A	Accurate
Marker3849	Chr. 6	17620387	T	T	C	T	C	Accurate
Marker3850	Chr. 6	17622323	A	A	C	A	C	Accurate
Marker3851	Chr. 6	17624840	T	T	G	T	G	Accurate
Marker3852	Chr. 6	17626190	G	G	A	G	A	Accurate
Marker3853	Chr. 6	17653735	G	G	C	G	C	Accurate
Marker3854	Chr. 6	17786728	A	A	G	A	G	Accurate
Marker3855	Chr. 6	17822642	T	T	C	T	C	Accurate
Marker3857	Chr. 6	17993049	C	C	A	C	A	Accurate
Marker3858	Chr. 6	18052044	A	A	G	A	G	Accurate
Marker3859	Chr. 6	18054944	A	A	G	A	G	Accurate
Marker3860	Chr. 6	18141995	G	G	C	G	C	Accurate
Marker3861	Chr. 6	18147285	T	T	C	T	C	Accurate
Marker3863	Chr. 6	18188927	T	T	C	T	C	Accurate
Marker3864	Chr. 6	18198301	T	T	A	T	A	Accurate
Marker3865	Chr. 6	18213154	C	C	T	C	T	Accurate
Marker3866	Chr. 6	18232052	G	G	T	G	T	Accurate
Marker3867	Chr. 6	18273224	G	A	G	A	G	Accurate
Marker3868	Chr. 6	18304336	C	C	T	C	T	Accurate
Marker3869	Chr. 6	18304360	A	A	G	A	G	Accurate
Marker3870	Chr. 6	18307919	G	G	A	G	A	Accurate
Marker3871	Chr. 6	18314166	G	G	A	G	A	Accurate
Marker3872	Chr. 6	18317928	C	C	T	C	T	Accurate
Marker3873	Chr. 6	18339671	A	A	G	A	G	Accurate
Marker3874	Chr. 6	18382851	T	T	C	T	C	Accurate
Marker3875	Chr. 6	18383471	A	A	G	A	G	Accurate

Marker3876	Chr. 6	18383744	C	C	T	C	T	Accurate
Marker3877	Chr. 6	18383951	G	G	A	G	A	Accurate
Marker3880	Chr. 6	18758121	T	T	C	N	C	Uncertain
Marker3882	Chr. 6	18763552	A	A	C	A	C	Accurate
Marker3887	Chr. 6	19844969	G	G	T	G	T	Accurate
Marker3888	Chr. 6	19926452	A	A	G	A	G	Accurate
Marker3891	Chr. 6	20439652	T	T	C	T	C	Accurate
Marker3892	Chr. 6	20520778	A	A	G	A	G	Accurate
Marker3893	Chr. 6	20543642	G	G	A	G	A	Accurate
Marker3894	Chr. 6	20543860	G	G	A	G	A	Accurate
Marker3896	Chr. 6	20558914	A	A	G	A	G	Accurate
Marker3898	Chr. 6	20695978	G	G	A	G	A	Accurate
Marker3899	Chr. 6	20696002	A	A	G	A	G	Accurate
Marker3900	Chr. 6	20721151	A	A	G	A	G	Accurate
Marker3901	Chr. 6	20721161	T	T	C	T	C	Accurate
Marker3902	Chr. 6	20724046	A	A	C	A	C	Accurate
Marker3903	Chr. 6	20749277	T	T	G	T	G	Accurate
Marker3904	Chr. 6	20759129	C	C	T	C	T	Accurate
Marker3906	Chr. 6	21200574	T	T	C	T	C	Accurate
Marker3907	Chr. 6	21202320	C	C	T	C	T	Accurate
Marker3908	Chr. 6	21210547	G	G	A	G	A	Accurate
Marker3909	Chr. 6	21355451	C	T	C	T	C	Accurate
Marker3911	Chr. 6	21415083	G	A	G	A	G	Accurate
Marker3913	Chr. 6	21462078	T	G	T	G	T	Accurate
Marker3914	Chr. 6	21463426	A	G	A	G	A	Accurate
Marker3915	Chr. 6	21469788	C	T	C	T	N	Uncertain
Marker3916	Chr. 6	21475803	A	C	A	C	A	Accurate
Marker3920	Chr. 6	21500668	A	G	A	G	A	Accurate
Marker3921	Chr. 6	21531919	G	A	G	A	G	Accurate
Marker3924	Chr. 6	21633187	C	T	C	T	C	Accurate
Marker3925	Chr. 6	21633387	C	A	C	A	C	Accurate
Marker3929	Chr. 6	21665103	A	C	A	C	A	Accurate
Marker3930	Chr. 6	21737418	G	T	G	T	G	Accurate
Marker3932	Chr. 6	21740861	T	C	T	C	T	Accurate
Marker3935	Chr. 6	21785730	A	A	G	N	G	Uncertain
Marker3937	Chr. 6	21809102	C	T	C	T	C	Accurate
Marker3942	Chr. 6	21871979	C	C	G	C	G	Accurate
Marker3943	Chr. 6	21923954	C	C	T	C	T	Accurate
Marker3944	Chr. 6	21923995	G	G	T	G	T	Accurate
Marker3946	Chr. 6	21948804	G	G	A	G	A	Accurate
Marker3948	Chr. 6	21971289	G	G	T	G	T	Accurate
Marker3951	Chr. 6	22197598	T	T	A	T	A	Accurate
Marker3952	Chr. 6	22197732	T	C	T	C	T	Accurate
Marker3953	Chr. 6	22203482	C	T	C	T	C	Accurate
Marker3955	Chr. 6	22325183	C	C	G	C	G	Accurate
Marker3956	Chr. 6	22325214	G	G	A	G	A	Accurate
Marker3957	Chr. 6	22325483	G	G	C	G	C	Accurate

Marker3958	Chr. 6	22332941	T	T	C	T	C	Accurate
Marker3959	Chr. 6	22332944	T	T	C	T	C	Accurate
Marker3960	Chr. 6	22368449	A	C	A	C	A	Accurate
Marker3962	Chr. 6	22522417	C	C	G	C	G	Accurate
Marker3963	Chr. 6	22522436	G	G	A	G	A	Accurate
Marker3964	Chr. 6	22522437	C	C	A	C	A	Accurate
Marker3965	Chr. 6	22529964	C	C	A	C	A	Accurate
Marker3967	Chr. 6	22534730	A	T	A	T	A	Accurate
Marker3968	Chr. 6	22534964	A	C	A	C	A	Accurate
Marker3969	Chr. 6	22538236	G	G	A	G	A	Accurate
Marker3970	Chr. 6	22629438	A	A	T	A	T	Accurate
Marker3971	Chr. 6	22758648	T	A	T	A	T	Accurate
Marker3972	Chr. 6	22825574	G	T	G	T	G	Accurate
Marker3974	Chr. 6	22828587	A	A	G	A	G	Accurate
Marker3976	Chr. 6	22923425	C	C	T	C	T	Accurate
Marker3977	Chr. 6	22923462	C	C	T	C	T	Accurate
Marker3978	Chr. 6	22923596	G	G	T	G	T	Accurate
Marker3980	Chr. 6	22958264	C	C	T	C	T	Accurate
Marker3981	Chr. 6	23120389	G	G	T	G	T	Accurate
Marker3982	Chr. 6	23121431	A	A	G	A	G	Accurate
Marker3985	Chr. 6	23150291	G	G	A	G	A	Accurate
Marker3986	Chr. 6	23308114	T	T	A	T	A	Accurate
Marker3987	Chr. 6	23337129	C	C	T	C	T	Accurate
Marker3991	Chr. 6	23446505	C	T	C	T	C	Accurate
Marker3996	Chr. 6	23760522	G	G	A	G	A	Accurate
Marker3998	Chr. 6	23794107	G	T	G	T	G	Accurate
Marker3999	Chr. 6	23808617	G	A	G	A	G	Accurate
Marker4000	Chr. 6	23884266	T	T	C	T	C	Accurate
Marker4001	Chr. 6	23906993	C	A	C	A	C	Accurate
Marker4002	Chr. 6	23921372	G	A	G	A	G	Accurate
Marker4003	Chr. 6	23938184	C	T	C	T	C	Accurate
Marker4006	Chr. 6	24052156	G	G	T	G	T	Accurate
Marker4009	Chr. 6	24182836	G	G	A	G	A	Accurate
Marker4010	Chr. 6	24197769	T	C	T	C	T	Accurate
Marker4011	Chr. 6	24248600	T	T	C	T	C	Accurate
Marker4014	Chr. 6	24285970	T	C	T	C	T	Accurate
Marker4018	Chr. 6	24337471	G	A	G	A	G	Accurate
Marker4019	Chr. 6	24393172	G	T	G	T	G	Accurate
Marker4020	Chr. 6	24397464	T	C	T	C	T	Accurate
Marker4022	Chr. 6	24438775	G	G	A	G	A	Accurate
Marker4024	Chr. 6	24474702	A	A	G	N	G	Uncertain
Marker4025	Chr. 6	24475020	A	A	G	A	G	Accurate
Marker4026	Chr. 6	24559357	G	A	G	A	N	Uncertain
Marker4027	Chr. 6	24559381	A	G	A	G	N	Uncertain
Marker4028	Chr. 6	24831629	C	C	T	C	T	Accurate
Marker4029	Chr. 6	24880994	C	C	T	C	T	Accurate
Marker4030	Chr. 6	24897716	G	G	T	G	T	Accurate

Marker4033	Chr. 6	25153220	C	C	T	C	T	Accurate
Marker4034	Chr. 6	25189594	C	C	T	C	T	Accurate
Marker4035	Chr. 6	25190283	A	G	A	G	A	Accurate
Marker4039	Chr. 6	25204019	G	G	A	G	A	Accurate
Marker4040	Chr. 6	25213350	T	T	C	T	C	Accurate
Marker4041	Chr. 6	25213548	T	T	G	T	G	Accurate
Marker4042	Chr. 6	25350191	G	A	G	A	G	Accurate
Marker4043	Chr. 6	25350192	T	C	T	C	T	Accurate
Marker4045	Chr. 6	25766071	C	C	T	N	T	Uncertain
Marker4046	Chr. 6	25766754	A	A	T	A	T	Accurate
Marker4049	Chr. 6	25816770	T	T	C	T	C	Accurate
Marker4051	Chr. 6	25832154	A	A	G	A	G	Accurate
Marker4052	Chr. 6	25832165	G	G	A	G	A	Accurate
Marker4053	Chr. 6	25832175	T	T	C	T	C	Accurate
Marker4054	Chr. 6	25832199	A	A	G	A	G	Accurate
Marker4056	Chr. 6	25832332	C	C	T	N	T	Uncertain
Marker4061	Chr. 6	25848051	G	G	C	G	C	Accurate
Marker4062	Chr. 6	25848072	C	C	T	C	T	Accurate
Marker4063	Chr. 6	25848077	A	A	G	A	G	Accurate
Marker4064	Chr. 6	25858916	C	C	T	C	T	Accurate
Marker4065	Chr. 6	25858923	T	T	C	T	C	Accurate
Marker4066	Chr. 6	25859119	C	C	T	C	T	Accurate
Marker4068	Chr. 6	25859767	G	G	T	G	T	Accurate
Marker4070	Chr. 6	25873782	A	A	T	A	T	Accurate
Marker4072	Chr. 6	25874840	G	G	A	G	A	Accurate
Marker4073	Chr. 6	25875008	G	G	A	G	A	Accurate
Marker4074	Chr. 6	25876935	A	A	T	A	T	Accurate
Marker4077	Chr. 6	25877191	C	C	T	C	T	Accurate
Marker4079	Chr. 6	25882216	G	G	C	G	C	Accurate
Marker4080	Chr. 6	25884911	A	A	C	A	C	Accurate
Marker4081	Chr. 6	25885210	C	C	A	C	A	Accurate
Marker4082	Chr. 6	25886549	C	C	G	C	G	Accurate
Marker4083	Chr. 6	25886565	T	T	A	T	A	Accurate
Marker4086	Chr. 6	25900072	C	C	A	C	A	Accurate
Marker4087	Chr. 6	25900101	T	T	C	T	C	Accurate
Marker4088	Chr. 6	25900534	A	A	G	A	G	Accurate
Marker4090	Chr. 6	25900847	C	C	T	C	T	Accurate
Marker4091	Chr. 6	25908504	T	T	C	T	C	Accurate
Marker4092	Chr. 6	25908696	A	A	G	A	G	Accurate
Marker4093	Chr. 6	25908809	G	G	T	G	T	Accurate
Marker4094	Chr. 6	25909016	C	C	T	C	T	Accurate
Marker4095	Chr. 6	25911966	T	T	C	T	C	Accurate
Marker4096	Chr. 6	25915108	T	T	G	T	G	Accurate
Marker4097	Chr. 6	25915143	G	G	T	G	T	Accurate
Marker4099	Chr. 6	25915323	G	G	C	G	C	Accurate
Marker4100	Chr. 6	25922654	T	T	C	T	C	Accurate
Marker4101	Chr. 6	25927817	C	C	T	C	T	Accurate

Marker4104	Chr. 6	25933255	G	G	T	G	T	Accurate
Marker4105	Chr. 6	25933445	A	A	G	A	G	Accurate
Marker4106	Chr. 6	25933447	G	G	A	G	A	Accurate
Marker4107	Chr. 6	25939365	T	T	C	T	C	Accurate
Marker4108	Chr. 6	25939370	G	G	A	G	A	Accurate
Marker4109	Chr. 6	25939567	T	T	C	T	C	Accurate
Marker4110	Chr. 6	25939585	G	G	A	G	A	Accurate
Marker4111	Chr. 6	25939629	C	C	T	C	T	Accurate
Marker4112	Chr. 6	25945470	G	G	T	G	T	Accurate
Marker4113	Chr. 6	25945676	A	A	G	A	G	Accurate
Marker4114	Chr. 6	25947944	T	T	A	T	A	Accurate
Marker4117	Chr. 6	25954017	A	A	G	A	G	Accurate
Marker4118	Chr. 6	25954237	A	A	G	A	G	Accurate
Marker4119	Chr. 6	25962395	G	G	A	G	A	Accurate
Marker4120	Chr. 6	25962423	G	G	A	G	A	Accurate
Marker4121	Chr. 6	25968319	G	G	A	G	A	Accurate
Marker4122	Chr. 6	25979003	C	C	T	C	T	Accurate
Marker4123	Chr. 6	25979048	A	A	G	A	G	Accurate
Marker4125	Chr. 6	25996230	C	C	T	C	T	Accurate
Marker4126	Chr. 6	25996233	C	C	T	C	T	Accurate
Marker4127	Chr. 6	25996387	C	C	T	C	T	Accurate
Marker4128	Chr. 6	25996457	T	T	C	T	C	Accurate
Marker4130	Chr. 6	26003856	A	A	C	A	C	Accurate
Marker4131	Chr. 6	26004112	C	C	T	C	T	Accurate
Marker4132	Chr. 6	26004114	T	T	A	T	A	Accurate
Marker4133	Chr. 6	26004204	T	T	A	T	A	Accurate
Marker4135	Chr. 6	26008406	T	T	A	T	A	Accurate
Marker4137	Chr. 6	26008664	T	T	A	T	A	Accurate
Marker4138	Chr. 6	26009345	T	T	C	T	C	Accurate
Marker4139	Chr. 6	26010002	A	A	T	A	T	Accurate
Marker4141	Chr. 6	26015721	A	A	G	A	G	Accurate
Marker4142	Chr. 6	26020562	C	C	T	C	T	Accurate
Marker4143	Chr. 6	26023306	C	C	T	C	T	Accurate
Marker4144	Chr. 6	26023331	T	T	C	T	C	Accurate
Marker4145	Chr. 6	26023547	A	A	T	A	T	Accurate
Marker4146	Chr. 6	26023583	C	C	T	C	T	Accurate
Marker4147	Chr. 6	26023592	G	G	A	G	A	Accurate
Marker4148	Chr. 6	26023627	G	G	A	G	A	Accurate
Marker4149	Chr. 6	26023639	T	T	C	T	C	Accurate
Marker4150	Chr. 6	26023641	G	G	A	G	A	Accurate
Marker4151	Chr. 6	26023683	A	A	T	A	T	Accurate
Marker4152	Chr. 6	26023901	T	T	C	T	C	Accurate
Marker4153	Chr. 6	26023914	T	T	C	T	C	Accurate
Marker4154	Chr. 6	26023936	G	G	A	G	A	Accurate
Marker4156	Chr. 6	26051090	C	C	T	C	T	Accurate
Marker4158	Chr. 6	26060616	A	A	G	A	G	Accurate
Marker4160	Chr. 6	26064589	C	C	T	C	T	Accurate

Marker4161	Chr. 6	26064608	C	C	T	C	T	Accurate
Marker4162	Chr. 6	26064617	A	A	T	A	T	Accurate
Marker4163	Chr. 6	26064760	A	A	G	A	G	Accurate
Marker4165	Chr. 6	26064784	T	T	G	T	G	Accurate
Marker4166	Chr. 6	26066118	G	G	A	G	A	Accurate
Marker4167	Chr. 6	26066141	T	T	C	T	C	Accurate
Marker4168	Chr. 6	26073981	G	G	A	G	A	Accurate
Marker4169	Chr. 6	26074004	A	A	G	A	G	Accurate
Marker4170	Chr. 6	26074018	G	G	A	G	A	Accurate
Marker4177	Chr. 6	26088313	T	T	G	T	G	Accurate
Marker4182	Chr. 6	26111499	T	T	C	T	C	Accurate
Marker4183	Chr. 6	26111516	T	T	C	T	C	Accurate
Marker4184	Chr. 6	26111523	T	T	C	T	C	Accurate
Marker4185	Chr. 6	26111649	A	A	T	A	T	Accurate
Marker4186	Chr. 6	26112064	G	G	T	G	T	Accurate
Marker4187	Chr. 6	26123640	G	G	A	G	A	Accurate
Marker4189	Chr. 6	26123814	A	A	G	A	G	Accurate
Marker4190	Chr. 6	26131201	T	T	C	T	C	Accurate
Marker4193	Chr. 6	26174025	C	C	T	C	T	Accurate
Marker4196	Chr. 6	26198231	G	G	A	G	A	Accurate
Marker4197	Chr. 6	26198233	A	A	T	A	T	Accurate
Marker4198	Chr. 6	26198362	G	G	A	G	A	Accurate
Marker4199	Chr. 6	26198375	A	A	C	A	C	Accurate
Marker4200	Chr. 6	26199386	T	T	C	T	C	Accurate
Marker4201	Chr. 6	26199401	T	T	C	N	C	Uncertain
Marker4203	Chr. 6	26202946	A	A	T	A	T	Accurate
Marker4204	Chr. 6	26202947	C	C	G	C	G	Accurate
Marker4205	Chr. 6	26202982	C	C	T	C	T	Accurate
Marker4207	Chr. 6	26208182	A	A	G	A	G	Accurate
Marker4208	Chr. 6	26210763	A	A	G	A	G	Accurate
Marker4209	Chr. 6	26210866	T	T	C	T	C	Accurate
Marker4210	Chr. 6	26211921	A	A	G	A	G	Accurate
Marker4211	Chr. 6	26211936	A	A	G	A	G	Accurate
Marker4212	Chr. 6	26227995	A	A	G	A	G	Accurate
Marker4213	Chr. 6	26228156	G	G	A	G	A	Accurate
Marker4214	Chr. 6	26228160	T	T	C	T	C	Accurate
Marker4215	Chr. 6	26228162	G	G	A	G	A	Accurate
Marker4217	Chr. 6	26229622	C	C	T	C	T	Accurate
Marker4218	Chr. 6	26229643	A	A	G	A	G	Accurate
Marker4219	Chr. 6	26229645	A	A	G	A	G	Accurate
Marker4220	Chr. 6	26229905	C	C	A	C	A	Accurate
Marker4221	Chr. 6	26231481	T	T	G	T	G	Accurate
Marker4223	Chr. 6	26236428	C	C	A	C	A	Accurate
Marker4224	Chr. 6	26236432	G	G	A	G	A	Accurate
Marker4225	Chr. 6	26236435	G	G	A	G	A	Accurate
Marker4226	Chr. 6	26236616	C	C	T	C	T	Accurate
Marker4227	Chr. 6	26236618	A	A	G	A	G	Accurate

Marker4229	Chr. 6	26248949	T	T	C	T	C	Accurate
Marker4230	Chr. 6	26251886	T	T	C	T	C	Accurate
Marker4231	Chr. 6	26251910	A	A	G	A	G	Accurate
Marker4232	Chr. 6	26253361	T	T	C	T	C	Accurate
Marker4233	Chr. 6	26261572	G	G	A	G	A	Accurate
Marker4234	Chr. 6	26261578	T	T	C	T	C	Accurate
Marker4235	Chr. 6	26261582	A	A	G	A	G	Accurate
Marker4236	Chr. 6	26261588	C	C	A	C	A	Accurate
Marker4237	Chr. 6	26261590	C	C	T	C	T	Accurate
Marker4243	Chr. 6	26304264	T	T	C	T	C	Accurate
Marker4244	Chr. 6	26304270	G	G	A	G	A	Accurate
Marker4245	Chr. 6	26304296	T	T	G	T	G	Accurate
Marker4246	Chr. 6	26304525	A	A	C	A	C	Accurate
Marker4247	Chr. 6	26304549	T	T	A	T	A	Accurate
Marker4248	Chr. 6	26307524	A	A	T	A	T	Accurate
Marker4249	Chr. 6	26307557	G	G	C	G	C	Accurate
Marker4250	Chr. 6	26311408	A	A	G	A	G	Accurate
Marker4251	Chr. 6	26311604	G	G	A	G	A	Accurate
Marker4252	Chr. 6	26313637	C	C	A	C	A	Accurate
Marker4253	Chr. 6	26313662	A	A	G	A	G	Accurate
Marker4254	Chr. 6	26313807	A	A	G	A	G	Accurate
Marker4256	Chr. 6	26324465	T	T	C	T	C	Accurate
Marker4259	Chr. 6	26335875	T	T	G	T	G	Accurate
Marker4260	Chr. 6	26335881	C	C	T	C	T	Accurate
Marker4261	Chr. 6	26348781	C	C	G	C	G	Accurate
Marker4262	Chr. 6	26349021	C	C	G	C	G	Accurate
Marker4263	Chr. 6	26349506	A	A	G	A	G	Accurate
Marker4264	Chr. 6	26349672	C	C	G	C	G	Accurate
Marker4265	Chr. 6	26349681	G	G	A	G	A	Accurate
Marker4267	Chr. 6	26354475	C	C	T	C	T	Accurate
Marker4270	Chr. 6	26380146	G	G	A	G	R	Uncertain
Marker4271	Chr. 6	26380153	G	G	A	G	R	Uncertain
Marker4272	Chr. 6	26380395	G	G	T	G	T	Accurate
Marker4276	Chr. 6	26407830	G	G	T	G	T	Accurate
Marker4277	Chr. 6	26418630	G	G	A	G	A	Accurate
Marker4283	Chr. 6	26425611	C	C	T	C	T	Accurate
Marker4284	Chr. 6	26425628	C	C	A	C	A	Accurate
Marker4285	Chr. 6	26425642	C	C	A	C	A	Accurate
Marker4286	Chr. 6	26425814	C	C	T	C	T	Accurate
Marker4290	Chr. 6	26451127	G	G	T	G	T	Accurate
Marker4291	Chr. 6	26451133	A	A	T	A	T	Accurate
Marker4292	Chr. 6	26451274	A	A	G	A	G	Accurate
Marker4293	Chr. 6	26451301	G	G	A	G	A	Accurate
Marker4294	Chr. 6	26466051	C	C	A	C	A	Accurate
Marker4295	Chr. 6	26466256	G	G	T	G	T	Accurate
Marker4296	Chr. 6	26473676	T	T	G	T	G	Accurate
Marker4297	Chr. 6	26473684	T	T	C	T	C	Accurate

Marker4298	Chr. 6	26473929	T	T	A	T	A	Accurate
Marker4299	Chr. 6	26476382	G	G	T	G	T	Accurate
Marker4300	Chr. 6	26476383	A	A	T	A	T	Accurate
Marker4301	Chr. 6	26476390	C	C	T	C	T	Accurate
Marker4302	Chr. 6	26476533	G	G	A	G	A	Accurate
Marker4303	Chr. 6	26476593	G	G	A	G	A	Accurate
Marker4304	Chr. 6	26479409	G	G	A	G	A	Accurate
Marker4307	Chr. 6	26487564	T	T	G	N	G	Uncertain
Marker4308	Chr. 6	26487576	A	A	G	N	G	Uncertain
Marker4309	Chr. 6	26487801	A	A	G	A	G	Accurate
Marker4310	Chr. 6	26487841	T	T	A	T	A	Accurate
Marker4312	Chr. 6	26491049	G	G	A	G	A	Accurate
Marker4313	Chr. 6	26491055	C	C	T	C	T	Accurate
Marker4314	Chr. 6	26491056	A	A	T	A	T	Accurate
Marker4315	Chr. 6	26491309	C	C	T	C	T	Accurate
Marker4316	Chr. 6	26491315	A	A	G	A	G	Accurate
Marker4317	Chr. 6	26495437	C	C	T	C	T	Accurate
Marker4319	Chr. 6	26505013	G	G	A	G	A	Accurate
Marker4320	Chr. 6	26505056	T	T	C	T	C	Accurate
Marker4321	Chr. 6	26512694	G	G	A	G	A	Accurate
Marker4323	Chr. 6	26512719	A	A	G	A	G	Accurate
Marker4324	Chr. 6	26512765	A	A	T	A	T	Accurate
Marker4325	Chr. 6	26516916	G	G	A	G	A	Accurate
Marker4328	Chr. 6	26517342	T	T	C	T	C	Accurate
Marker4329	Chr. 6	26517363	A	A	G	A	G	Accurate
Marker4330	Chr. 6	26517377	C	C	T	C	T	Accurate
Marker4331	Chr. 6	26517524	A	A	C	A	C	Accurate
Marker4332	Chr. 6	26518226	A	A	G	A	G	Accurate
Marker4333	Chr. 6	26518231	T	T	C	T	C	Accurate
Marker4334	Chr. 6	26520429	G	G	A	G	A	Accurate
Marker4335	Chr. 6	26520477	C	C	T	C	T	Accurate
Marker4337	Chr. 6	26524341	T	T	G	T	G	Accurate
Marker4338	Chr. 6	26524367	G	G	A	G	A	Accurate
Marker4339	Chr. 6	26524395	C	C	T	C	T	Accurate
Marker4340	Chr. 6	26529748	T	T	C	T	C	Accurate
Marker4341	Chr. 6	26530069	A	A	T	A	T	Accurate
Marker4343	Chr. 6	26531358	G	G	A	G	A	Accurate
Marker4345	Chr. 6	26553226	G	G	T	G	T	Accurate
Marker4347	Chr. 6	26553255	G	G	A	G	A	Accurate
Marker4348	Chr. 6	26553421	C	C	T	C	T	Accurate
Marker4351	Chr. 6	26555381	C	C	G	C	G	Accurate
Marker4356	Chr. 6	26565605	A	A	C	A	C	Accurate
Marker4357	Chr. 6	26565618	G	G	A	G	A	Accurate
Marker4358	Chr. 6	26565787	C	C	A	C	A	Accurate
Marker4360	Chr. 6	26569000	T	T	C	T	C	Accurate
Marker4361	Chr. 6	26571462	C	C	T	C	T	Accurate
Marker4362	Chr. 6	26571465	G	G	A	G	A	Accurate

Marker4363	Chr. 6	26571613	A	A	G	A	G	Accurate
Marker4364	Chr. 6	26571659	A	A	G	A	G	Accurate
Marker4365	Chr. 6	26573097	T	T	A	T	A	Accurate
Marker4366	Chr. 6	26573118	C	C	T	C	T	Accurate
Marker4377	Chr. 6	26583222	A	A	G	A	G	Accurate
Marker4378	Chr. 6	26583228	T	T	C	T	C	Accurate
Marker4379	Chr. 6	26583230	G	G	A	G	A	Accurate
Marker4380	Chr. 6	26583263	T	T	G	T	G	Accurate
Marker4381	Chr. 6	26583267	G	G	A	G	A	Accurate
Marker4382	Chr. 6	26583269	C	C	T	C	T	Accurate
Marker4383	Chr. 6	26583270	C	C	A	C	A	Accurate
Marker4384	Chr. 6	26583389	T	T	C	T	C	Accurate
Marker4385	Chr. 6	26583400	G	G	T	G	T	Accurate
Marker4386	Chr. 6	26583426	C	C	T	C	T	Accurate
Marker4387	Chr. 6	26583469	T	T	C	T	C	Accurate
Marker4388	Chr. 6	26583643	A	A	T	A	T	Accurate
Marker4389	Chr. 6	26583680	A	A	G	A	G	Accurate
Marker4390	Chr. 6	26597814	G	G	T	G	T	Accurate
Marker4391	Chr. 6	26597984	T	T	C	T	C	Accurate
Marker4392	Chr. 6	26597993	T	T	C	T	C	Accurate
Marker4395	Chr. 6	26599439	A	A	G	A	G	Accurate
Marker4396	Chr. 6	26600431	T	T	A	T	A	Accurate
Marker4397	Chr. 6	26602808	T	T	G	T	G	Accurate
Marker4398	Chr. 6	26602810	G	G	C	G	C	Accurate
Marker4402	Chr. 6	26606495	T	T	A	T	A	Accurate
Marker4403	Chr. 6	26606686	A	A	G	A	G	Accurate
Marker4404	Chr. 6	26606712	T	T	C	T	C	Accurate
Marker4405	Chr. 6	26606717	G	G	A	G	A	Accurate
Marker4406	Chr. 6	26606740	G	G	A	G	A	Accurate
Marker4407	Chr. 6	26608736	A	A	C	A	C	Accurate
Marker4408	Chr. 6	26608960	C	C	T	C	T	Accurate
Marker4409	Chr. 6	26612229	G	G	A	G	A	Accurate
Marker4410	Chr. 6	26612265	T	T	C	T	C	Accurate
Marker4411	Chr. 6	26617800	A	A	C	A	C	Accurate
Marker4412	Chr. 6	26617841	T	T	G	T	G	Accurate
Marker4413	Chr. 6	26617980	C	C	T	C	T	Accurate
Marker4414	Chr. 6	26618005	C	C	T	C	T	Accurate
Marker4416	Chr. 6	26621204	C	C	T	C	T	Accurate
Marker4417	Chr. 6	26621224	G	G	C	G	C	Accurate
Marker4418	Chr. 6	26621237	G	G	A	G	A	Accurate
Marker4420	Chr. 6	26651886	T	T	G	T	G	Accurate
Marker4421	Chr. 6	26654996	C	C	A	C	A	Accurate
Marker4423	Chr. 6	26658580	G	G	A	G	A	Accurate
Marker4426	Chr. 6	26659382	A	A	C	A	C	Accurate
Marker4433	Chr. 6	26700208	G	G	A	G	A	Accurate
Marker4434	Chr. 6	26700384	A	A	G	A	G	Accurate
Marker4435	Chr. 6	26721782	A	A	T	A	T	Accurate

Marker4436	Chr. 6	26723874	G	G	C	G	C	Accurate
Marker4437	Chr. 6	26724372	A	A	G	A	G	Accurate
Marker4438	Chr. 6	26724441	A	A	G	A	G	Accurate
Marker4439	Chr. 6	26724602	G	G	A	G	A	Accurate
Marker4440	Chr. 6	26724616	G	G	A	G	A	Accurate
Marker4441	Chr. 6	26724649	T	T	G	T	G	Accurate
Marker4442	Chr. 6	26724703	C	C	T	C	T	Accurate
Marker4443	Chr. 6	26733538	A	A	G	A	G	Accurate
Marker4444	Chr. 6	26733782	G	G	A	G	A	Accurate
Marker4445	Chr. 6	26736764	C	C	T	C	T	Accurate
Marker4446	Chr. 6	26736777	G	G	A	G	A	Accurate
Marker4450	Chr. 6	26758536	A	A	G	A	G	Accurate
Marker4451	Chr. 6	26758547	A	A	G	A	G	Accurate
Marker4453	Chr. 6	26759523	G	G	A	G	A	Accurate
Marker4454	Chr. 6	26759547	G	G	A	N	A	Uncertain
Marker4455	Chr. 6	26759549	A	A	G	N	G	Uncertain
Marker4456	Chr. 6	26777745	G	G	C	G	C	Accurate
Marker4457	Chr. 6	26834664	T	T	C	T	C	Accurate
Marker4459	Chr. 6	26847922	T	T	C	T	C	Accurate
Marker4460	Chr. 6	26848202	C	C	T	C	T	Accurate
Marker4463	Chr. 7	154295	A	A	T	A	T	Accurate
Marker4464	Chr. 7	158695	A	G	A	G	A	Accurate
Marker4466	Chr. 7	170972	T	A	T	A	T	Accurate
Marker4468	Chr. 7	246355	C	T	C	T	C	Accurate
Marker4469	Chr. 7	261199	T	G	T	G	T	Accurate
Marker4471	Chr. 7	309253	T	C	T	C	T	Accurate
Marker4472	Chr. 7	309706	G	A	G	A	G	Accurate
Marker4473	Chr. 7	315874	A	G	A	G	A	Accurate
Marker4474	Chr. 7	589032	A	G	A	G	A	Accurate
Marker4475	Chr. 7	671300	A	T	A	T	A	Accurate
Marker4476	Chr. 7	737239	T	G	T	G	T	Accurate
Marker4477	Chr. 7	740267	A	C	A	C	A	Accurate
Marker4478	Chr. 7	740542	A	G	A	G	A	Accurate
Marker4480	Chr. 7	783556	G	G	A	G	A	Accurate
Marker4481	Chr. 7	844818	C	C	A	C	A	Accurate
Marker4482	Chr. 7	912555	G	G	A	G	A	Accurate
Marker4483	Chr. 7	1100681	A	T	A	T	A	Accurate
Marker4484	Chr. 7	1100738	A	G	A	G	A	Accurate
Marker4485	Chr. 7	1103759	A	T	A	T	A	Accurate
Marker4486	Chr. 7	1284659	A	A	G	A	G	Accurate
Marker4487	Chr. 7	1320074	T	T	G	T	G	Accurate
Marker4488	Chr. 7	1320724	T	T	C	T	C	Accurate
Marker4489	Chr. 7	1324944	G	G	A	G	A	Accurate
Marker4496	Chr. 7	1417939	C	A	C	A	C	Accurate
Marker4500	Chr. 7	1763373	T	A	T	A	T	Accurate
Marker4502	Chr. 7	1784213	G	A	G	A	G	Accurate
Marker4503	Chr. 7	1784329	C	T	C	T	C	Accurate

Marker4505	Chr. 7	1792013	T	C	T	C	T	Accurate
Marker4506	Chr. 7	1851809	C	T	C	T	C	Accurate
Marker4507	Chr. 7	1851827	A	T	A	T	A	Accurate
Marker4508	Chr. 7	1860712	C	T	C	T	C	Accurate
Marker4509	Chr. 7	1860981	A	G	A	G	A	Accurate
Marker4510	Chr. 7	1862740	T	C	T	C	T	Accurate
Marker4511	Chr. 7	1969104	A	C	A	C	A	Accurate
Marker4513	Chr. 7	1982424	A	G	A	G	A	Accurate
Marker4515	Chr. 7	1995213	G	A	G	A	G	Accurate
Marker4516	Chr. 7	2021417	T	C	T	C	T	Accurate
Marker4521	Chr. 7	2305550	T	C	T	C	T	Accurate
Marker4522	Chr. 7	2305801	G	A	G	A	G	Accurate
Marker4523	Chr. 7	2324653	A	G	A	G	A	Accurate
Marker4526	Chr. 7	2335010	G	A	T	A	G	Inconsistent
Marker4531	Chr. 7	2564167	G	A	G	A	G	Accurate
Marker4532	Chr. 7	2564169	G	A	G	A	G	Accurate
Marker4533	Chr. 7	2775892	C	T	C	T	C	Accurate
Marker4534	Chr. 7	2970008	C	C	T	N	T	Uncertain
Marker4536	Chr. 7	3142094	C	C	T	C	T	Accurate
Marker4537	Chr. 7	3154923	A	A	T	A	T	Accurate
Marker4538	Chr. 7	3266074	A	A	T	A	T	Accurate
Marker4541	Chr. 7	3305386	C	C	T	N	T	Uncertain
Marker4544	Chr. 7	3325294	C	C	T	C	T	Accurate
Marker4545	Chr. 7	3325384	A	A	T	A	T	Accurate
Marker4546	Chr. 7	3334412	G	G	A	G	A	Accurate
Marker4547	Chr. 7	3347076	T	T	A	T	A	Accurate
Marker4550	Chr. 7	3422291	G	G	A	G	A	Accurate
Marker4551	Chr. 7	3422292	G	G	A	G	A	Accurate
Marker4552	Chr. 7	3535681	T	T	C	T	C	Accurate
Marker4553	Chr. 7	3537501	T	T	C	T	C	Accurate
Marker4554	Chr. 7	3550108	T	T	C	T	C	Accurate
Marker4555	Chr. 7	3580499	C	C	T	C	T	Accurate
Marker4556	Chr. 7	3671783	A	A	C	A	C	Accurate
Marker4557	Chr. 7	3692379	C	C	T	C	T	Accurate
Marker4558	Chr. 7	3709252	A	A	G	A	G	Accurate
Marker4559	Chr. 7	3710546	T	T	C	T	C	Accurate
Marker4561	Chr. 7	3960343	A	G	A	G	A	Accurate
Marker4562	Chr. 7	3967513	T	A	T	A	T	Accurate
Marker4563	Chr. 7	3979403	C	C	A	C	A	Accurate
Marker4564	Chr. 7	4014304	C	G	C	G	C	Accurate
Marker4567	Chr. 7	4172978	G	G	T	G	T	Accurate
Marker4568	Chr. 7	4172991	G	G	A	G	A	Accurate
Marker4569	Chr. 7	4173035	A	A	T	A	T	Accurate
Marker4570	Chr. 7	4222122	G	A	G	A	G	Accurate
Marker4574	Chr. 7	4361740	G	G	A	G	A	Accurate
Marker4577	Chr. 7	4593959	G	G	A	G	A	Accurate
Marker4579	Chr. 7	4671762	C	T	C	T	C	Accurate

Marker4583	Chr. 7	5247481	C	T	C	T	C	Accurate
Marker4584	Chr. 7	5345798	A	A	G	A	G	Accurate
Marker4586	Chr. 7	5389795	G	G	A	G	A	Accurate
Marker4588	Chr. 7	5430300	T	T	C	T	C	Accurate
Marker4591	Chr. 7	5489634	T	T	C	T	C	Accurate
Marker4592	Chr. 7	5531097	T	T	A	T	A	Accurate
Marker4594	Chr. 7	5737127	A	G	A	G	A	Accurate
Marker4596	Chr. 7	5754673	A	G	A	G	A	Accurate
Marker4597	Chr. 7	5846174	T	G	T	G	T	Accurate
Marker4598	Chr. 7	5910879	G	A	G	A	G	Accurate
Marker4601	Chr. 7	5947089	C	T	C	T	C	Accurate
Marker4603	Chr. 7	6977416	C	C	T	C	T	Accurate
Marker4605	Chr. 7	7800921	T	C	T	C	T	Accurate
Marker4607	Chr. 7	7882637	C	T	C	T	C	Accurate
Marker4608	Chr. 7	7890044	A	G	A	G	A	Accurate
Marker4609	Chr. 7	7902312	A	C	A	C	A	Accurate
Marker4610	Chr. 7	7904495	T	A	T	A	T	Accurate
Marker4612	Chr. 7	7954021	G	C	G	C	G	Accurate
Marker4614	Chr. 7	8101564	G	G	A	G	A	Accurate
Marker4616	Chr. 7	8114983	T	T	G	T	G	Accurate
Marker4618	Chr. 7	8179506	C	C	T	C	T	Accurate
Marker4619	Chr. 7	8196908	G	G	A	G	A	Accurate
Marker4622	Chr. 7	8669615	C	T	C	T	C	Accurate
Marker4623	Chr. 7	8673444	C	T	C	T	C	Accurate
Marker4627	Chr. 7	8837192	A	A	T	A	T	Accurate
Marker4628	Chr. 7	8837223	T	T	C	T	C	Accurate
Marker4629	Chr. 7	8837377	A	A	C	A	C	Accurate
Marker4630	Chr. 7	8844895	A	A	G	A	G	Accurate
Marker4632	Chr. 7	8847851	T	T	C	T	C	Accurate
Marker4633	Chr. 7	8866586	C	G	C	G	C	Accurate
Marker4634	Chr. 7	8866917	T	C	T	C	T	Accurate
Marker4635	Chr. 7	8901966	T	A	T	A	T	Accurate
Marker4636	Chr. 7	8902353	T	C	T	C	T	Accurate
Marker4637	Chr. 7	8906706	C	A	C	A	C	Accurate
Marker4640	Chr. 7	11266817	C	C	T	C	T	Accurate
Marker4658	Chr. 7	19851341	A	T	A	T	A	Accurate
Marker4659	Chr. 7	19968107	G	T	G	T	G	Accurate
Marker4660	Chr. 7	19970905	T	C	T	C	T	Accurate
Marker4663	Chr. 7	19999039	A	G	A	G	A	Accurate
Marker4665	Chr. 7	20018796	G	A	G	A	G	Accurate
Marker4666	Chr. 7	20021933	A	G	A	G	A	Accurate
Marker4667	Chr. 7	20021944	A	G	A	G	A	Accurate
Marker4668	Chr. 7	20021981	A	G	A	G	N	Uncertain
Marker4670	Chr. 7	20026287	C	G	C	G	C	Accurate
Marker4673	Chr. 7	20043419	G	A	G	A	G	Accurate
Marker4676	Chr. 7	20107409	G	A	G	A	G	Accurate
Marker4680	Chr. 7	20449706	C	T	C	T	C	Accurate

Marker4681	Chr. 7	20551440	T	C	T	C	T	Accurate
Marker4682	Chr. 7	20554843	G	A	G	A	G	Accurate
Marker4683	Chr. 7	20574878	A	G	A	G	A	Accurate
Marker4686	Chr. 7	20628403	A	G	A	G	A	Accurate
Marker4687	Chr. 7	20676598	A	G	A	G	A	Accurate
Marker4691	Chr. 7	21761550	G	G	A	G	A	Accurate
Marker4692	Chr. 7	21777660	G	G	T	G	T	Accurate
Marker4693	Chr. 7	21779090	G	G	C	G	C	Accurate
Marker4694	Chr. 7	21822677	C	C	T	C	T	Accurate
Marker4695	Chr. 7	21828500	C	C	A	C	A	Accurate
Marker4696	Chr. 7	21943244	T	T	A	T	A	Accurate
Marker4697	Chr. 7	21943440	T	T	G	T	G	Accurate
Marker4698	Chr. 7	21954878	T	T	A	T	A	Accurate
Marker4699	Chr. 7	21970575	A	A	G	A	G	Accurate
Marker4700	Chr. 7	21985961	A	A	G	A	G	Accurate
Marker4701	Chr. 7	22000653	A	A	G	A	G	Accurate
Marker4704	Chr. 7	22015374	C	T	C	T	C	Accurate
Marker4705	Chr. 7	22039050	A	C	A	C	A	Accurate
Marker4706	Chr. 7	22066541	T	T	C	T	C	Accurate
Marker4707	Chr. 7	22066547	C	C	A	C	A	Accurate
Marker4708	Chr. 7	22080624	C	C	T	C	T	Accurate
Marker4709	Chr. 7	22096317	G	G	A	G	A	Accurate
Marker4710	Chr. 7	22099320	T	G	T	G	T	Accurate
Marker4711	Chr. 7	22128220	A	T	A	T	A	Accurate
Marker4712	Chr. 7	22161589	C	T	C	T	C	Accurate
Marker4714	Chr. 7	22210623	A	G	A	G	A	Accurate
Marker4715	Chr. 7	22220350	A	G	A	G	A	Accurate
Marker4716	Chr. 7	22222864	G	A	G	A	G	Accurate
Marker4717	Chr. 7	22223063	G	A	G	A	G	Accurate
Marker4718	Chr. 7	22246851	G	A	G	A	G	Accurate
Marker4719	Chr. 7	22250547	A	G	A	G	A	Accurate
Marker4720	Chr. 7	22252077	A	G	A	G	A	Accurate
Marker4721	Chr. 7	22255289	A	G	A	G	A	Accurate
Marker4723	Chr. 7	22334046	A	C	A	C	A	Accurate
Marker4724	Chr. 7	22349728	A	G	A	G	A	Accurate
Marker4725	Chr. 7	22390781	T	C	T	C	T	Accurate
Marker4726	Chr. 7	22398973	C	T	C	T	C	Accurate
Marker4727	Chr. 7	22418204	A	A	G	A	G	Accurate
Marker4728	Chr. 7	22427217	G	G	C	G	C	Accurate
Marker4729	Chr. 7	22440446	T	T	C	T	C	Accurate
Marker4730	Chr. 7	22454120	G	G	A	G	A	Accurate
Marker4731	Chr. 7	22471440	T	T	C	T	C	Accurate
Marker4732	Chr. 7	22733801	T	T	C	T	C	Accurate
Marker4733	Chr. 7	22757296	A	A	G	A	G	Accurate
Marker4734	Chr. 7	22757646	T	T	C	T	C	Accurate
Marker4735	Chr. 7	22757835	G	G	T	G	T	Accurate
Marker4736	Chr. 7	22758243	C	C	T	C	T	Accurate

Marker4737	Chr. 7	22790848	A	A	G	A	G	Accurate
Marker4738	Chr. 7	22893958	A	T	A	T	A	Accurate
Marker4739	Chr. 7	23054611	G	A	G	A	G	Accurate
Marker4740	Chr. 7	23054904	T	T	C	T	C	Accurate
Marker4741	Chr. 7	23073347	G	G	A	G	A	Accurate
Marker4742	Chr. 7	23073531	G	G	T	G	T	Accurate
Marker4743	Chr. 7	23073770	A	A	G	A	G	Accurate
Marker4744	Chr. 7	23075881	A	A	G	A	G	Accurate
Marker4746	Chr. 7	23079514	G	A	G	A	G	Accurate
Marker4747	Chr. 7	23083058	A	A	G	A	G	Accurate
Marker4748	Chr. 7	23108242	C	C	A	C	A	Accurate
Marker4749	Chr. 7	23108361	T	T	G	T	G	Accurate
Marker4750	Chr. 7	23113177	A	A	C	A	C	Accurate
Marker4751	Chr. 7	23116790	A	A	G	A	G	Accurate
Marker4752	Chr. 7	23122920	G	G	A	G	A	Accurate
Marker4753	Chr. 7	23134919	G	G	A	G	A	Accurate
Marker4754	Chr. 7	23174072	C	A	C	A	C	Accurate
Marker4755	Chr. 7	23174485	G	A	G	A	G	Accurate
Marker4756	Chr. 7	23181324	T	C	T	C	T	Accurate
Marker4757	Chr. 7	23233664	C	C	T	C	T	Accurate
Marker4758	Chr. 7	23641920	C	T	C	T	C	Accurate
Marker4759	Chr. 7	23643662	G	T	G	T	G	Accurate
Marker4760	Chr. 7	23653811	C	T	C	T	C	Accurate
Marker4761	Chr. 7	23659880	A	G	A	G	N	Uncertain
Marker4762	Chr. 7	23663524	T	G	T	G	T	Accurate
Marker4763	Chr. 7	23681317	A	G	A	G	N	Uncertain
Marker4764	Chr. 7	23687556	T	C	T	C	T	Accurate
Marker4765	Chr. 7	23687783	A	C	A	C	A	Accurate
Marker4766	Chr. 7	23693729	A	G	A	G	N	Uncertain
Marker4767	Chr. 7	23881671	C	T	C	T	C	Accurate
Marker4768	Chr. 7	23884466	T	C	T	C	T	Accurate
Marker4769	Chr. 7	23886762	A	G	A	G	A	Accurate
Marker4770	Chr. 7	23901570	A	G	A	G	A	Accurate
Marker4771	Chr. 7	23905289	G	T	G	T	G	Accurate
Marker4772	Chr. 7	23922155	A	G	A	G	A	Accurate
Marker4773	Chr. 7	23931246	G	T	G	T	G	Accurate
Marker4774	Chr. 7	23948146	T	C	T	C	N	Uncertain
Marker4775	Chr. 7	24192365	A	C	A	C	A	Accurate
Marker4776	Chr. 7	24192608	C	A	C	A	C	Accurate
Marker4777	Chr. 7	24204240	A	T	A	T	A	Accurate
Marker4778	Chr. 7	24252246	C	T	C	T	C	Accurate
Marker4787	Chr. 7	24431772	A	G	A	G	A	Accurate
Marker4789	Chr. 7	24433494	A	G	A	G	A	Accurate
Marker4791	Chr. 7	24440074	C	T	C	T	C	Accurate
Marker4794	Chr. 7	24483675	G	T	G	T	N	Uncertain
Marker4796	Chr. 7	24563966	G	A	G	A	G	Accurate
Marker4797	Chr. 7	24564280	A	G	A	G	A	Accurate

Marker4799	Chr. 7	24636973	C	T	C	T	C	Accurate
Marker4800	Chr. 7	24823431	T	G	T	G	T	Accurate
Marker4801	Chr. 7	24852140	T	A	T	A	T	Accurate
Marker4802	Chr. 7	24852398	G	A	G	A	G	Accurate
Marker4803	Chr. 7	24991034	T	C	T	C	T	Accurate
Marker4804	Chr. 7	24991046	G	A	G	A	G	Accurate
Marker4805	Chr. 7	24991049	G	C	G	C	G	Accurate
Marker4806	Chr. 7	24999743	T	C	T	C	T	Accurate
Marker4807	Chr. 7	25001454	T	C	T	C	T	Accurate
Marker4808	Chr. 7	25015884	T	T	C	T	C	Accurate
Marker4809	Chr. 7	25016143	G	G	A	G	A	Accurate
Marker4810	Chr. 7	25020215	C	C	T	N	T	Uncertain
Marker4811	Chr. 7	25037116	G	A	G	A	G	Accurate
Marker4814	Chr. 7	25510930	T	C	T	C	T	Accurate
Marker4815	Chr. 7	25510936	C	T	C	T	C	Accurate
Marker4816	Chr. 7	25679487	C	C	T	C	T	Accurate
Marker4817	Chr. 7	25682267	G	G	A	G	A	Accurate
Marker4818	Chr. 7	25695208	C	C	T	C	T	Accurate
Marker4819	Chr. 7	25700599	T	T	C	T	C	Accurate
Marker4821	Chr. 7	25709020	G	G	T	G	T	Accurate
Marker4822	Chr. 7	25722716	T	T	A	T	A	Accurate
Marker4823	Chr. 7	25753054	A	A	T	A	T	Accurate
Marker4824	Chr. 7	25753244	G	G	A	G	A	Accurate
Marker4825	Chr. 7	25764649	T	T	C	T	C	Accurate
Marker4826	Chr. 7	25817198	G	A	G	A	G	Accurate
Marker4827	Chr. 7	25817696	T	C	T	C	T	Accurate
Marker4828	Chr. 7	25823218	A	T	A	T	A	Accurate
Marker4830	Chr. 7	25866531	T	T	C	T	C	Accurate
Marker4834	Chr. 7	25968997	C	T	C	T	C	Accurate
Marker4835	Chr. 7	25974147	T	G	T	G	T	Accurate
Marker4836	Chr. 7	25978739	C	T	C	T	C	Accurate
Marker4837	Chr. 7	25981776	T	C	T	C	T	Accurate
Marker4838	Chr. 7	26038409	T	C	T	C	T	Accurate
Marker4839	Chr. 7	26061734	T	C	T	C	T	Accurate
Marker4840	Chr. 7	26083286	A	T	A	T	A	Accurate
Marker4841	Chr. 7	26099756	C	G	C	G	C	Accurate
Marker4842	Chr. 7	26110002	C	A	C	A	C	Accurate
Marker4845	Chr. 7	26138475	G	A	G	A	G	Accurate
Marker4846	Chr. 7	26138511	T	A	T	A	T	Accurate
Marker4847	Chr. 7	26138553	A	C	A	C	A	Accurate
Marker4848	Chr. 7	26138578	A	C	A	C	A	Accurate
Marker4849	Chr. 7	26138827	A	C	A	C	A	Accurate
Marker4850	Chr. 7	26147295	A	G	A	G	A	Accurate
Marker4851	Chr. 7	26167642	C	T	C	T	C	Accurate
Marker4852	Chr. 7	26176251	T	G	T	G	N	Uncertain
Marker4854	Chr. 7	26372940	C	T	C	T	C	Accurate
Marker4855	Chr. 7	26381321	T	G	T	G	T	Accurate

Marker4856	Chr. 7	26385397	C	T	C	T	C	Accurate
Marker4858	Chr. 7	26429250	A	G	A	G	A	Accurate
Marker4859	Chr. 7	26435707	A	G	A	G	A	Accurate
Marker4862	Chr. 7	26531102	T	C	T	C	T	Accurate
Marker4864	Chr. 7	26658362	G	G	A	G	R	Uncertain
Marker4865	Chr. 7	26658416	A	A	G	A	G	Accurate
Marker4866	Chr. 7	26658666	G	G	A	G	A	Accurate
Marker4867	Chr. 7	26658912	G	G	A	G	A	Accurate
Marker4868	Chr. 7	26680351	C	T	C	T	C	Accurate
Marker4869	Chr. 7	26683554	A	G	A	G	A	Accurate
Marker4872	Chr. 7	26854941	T	C	T	C	T	Accurate
Marker4873	Chr. 7	26903318	A	A	C	A	C	Accurate
Marker4874	Chr. 7	26904078	A	A	C	A	C	Accurate
Marker4875	Chr. 7	26935152	C	C	T	C	T	Accurate
Marker4876	Chr. 7	26939021	A	A	T	A	T	Accurate
Marker4877	Chr. 7	26949143	T	T	C	T	C	Accurate
Marker4878	Chr. 7	26952211	G	G	A	G	A	Accurate
Marker4882	Chr. 7	27482991	T	T	C	T	C	Accurate
Marker4883	Chr. 7	27587845	A	A	G	A	G	Accurate
Marker4884	Chr. 7	27600543	C	C	G	C	G	Accurate
Marker4885	Chr. 7	27610929	G	G	A	G	A	Accurate
Marker4886	Chr. 7	27670339	A	A	G	A	G	Accurate
Marker4888	Chr. 7	27727920	G	G	A	G	A	Accurate
Marker4889	Chr. 7	27728242	C	C	A	C	A	Accurate
Marker4891	Chr. 7	27742740	G	G	T	G	T	Accurate
Marker4892	Chr. 7	27858690	A	A	G	A	G	Accurate
Marker4893	Chr. 7	27858923	C	C	T	C	T	Accurate
Marker4894	Chr. 7	27860845	G	G	A	G	A	Accurate
Marker4895	Chr. 7	27862049	T	T	G	T	G	Accurate
Marker4896	Chr. 7	27871706	T	T	C	T	C	Accurate
Marker4897	Chr. 7	27871745	G	G	A	G	A	Accurate
Marker4898	Chr. 7	27877746	G	G	T	G	T	Accurate
Marker4899	Chr. 7	27878350	T	T	A	N	A	Uncertain
Marker4904	Chr. 7	27892967	A	A	T	A	T	Accurate
Marker4905	Chr. 7	28013570	T	T	G	T	G	Accurate
Marker4906	Chr. 7	28058525	T	A	T	A	T	Accurate
Marker4909	Chr. 7	28064234	G	T	G	T	G	Accurate
Marker4912	Chr. 7	28068213	A	C	A	C	A	Accurate
Marker4913	Chr. 7	28068688	T	G	T	G	T	Accurate
Marker4914	Chr. 7	28086858	G	T	G	T	G	Accurate
Marker4915	Chr. 7	28087322	T	A	T	A	T	Accurate
Marker4916	Chr. 7	28094513	G	A	G	A	G	Accurate
Marker4917	Chr. 7	28116943	G	A	G	A	G	Accurate
Marker4919	Chr. 7	28139175	C	C	T	C	T	Accurate
Marker4920	Chr. 7	28155520	T	G	T	G	T	Accurate
Marker4921	Chr. 7	28155590	T	A	T	A	T	Accurate
Marker4922	Chr. 7	28190521	T	T	G	T	G	Accurate

Marker4924	Chr. 7	28251449	C	T	C	T	C	Accurate
Marker4925	Chr. 7	28259041	G	A	G	A	G	Accurate
Marker4926	Chr. 7	28264567	A	G	A	G	A	Accurate
Marker4927	Chr. 7	28336799	C	C	T	C	T	Accurate
Marker4928	Chr. 7	28401512	T	T	C	T	C	Accurate
Marker4929	Chr. 7	28404166	G	G	A	G	A	Accurate
Marker4933	Chr. 7	28689443	G	G	A	G	A	Accurate
Marker4937	Chr. 7	28761424	C	C	A	C	A	Accurate
Marker4938	Chr. 7	28767262	T	T	A	T	A	Accurate
Marker4939	Chr. 7	28783381	A	A	C	A	C	Accurate
Marker4940	Chr. 7	28797777	T	T	C	T	C	Accurate
Marker4941	Chr. 7	28846524	T	T	C	T	C	Accurate
Marker4943	Chr. 7	29068607	A	G	A	G	A	Accurate
Marker4944	Chr. 7	29070156	C	T	C	T	C	Accurate
Marker4945	Chr. 7	29262978	A	G	A	G	A	Accurate
Marker4947	Chr. 7	29267971	G	T	G	T	G	Accurate
Marker4949	Chr. 7	29323226	C	C	A	C	A	Accurate
Marker4950	Chr. 7	29334174	A	T	A	T	N	Uncertain
Marker4951	Chr. 7	29334175	A	T	A	T	A	Accurate
Marker4953	Chr. 7	29382166	T	T	G	T	G	Accurate
Marker4955	Chr. 7	29540297	C	G	C	G	C	Accurate
Marker4958	Chr. 7	29598752	G	T	G	T	G	Accurate
Marker4962	Chr. 7	29806754	G	G	A	G	A	Accurate
Marker4963	Chr. 7	29899669	A	G	A	G	A	Accurate
Marker4967	Chr. 7	30080421	A	T	A	T	A	Accurate
Marker4968	Chr. 7	30324567	C	C	T	C	T	Accurate
Marker4969	Chr. 7	30324616	G	G	A	G	A	Accurate
Marker4970	Chr. 7	30325534	T	T	C	N	C	Uncertain
Marker4971	Chr. 7	30325549	G	G	A	N	A	Uncertain
Marker4972	Chr. 7	30330832	G	G	A	G	A	Accurate
Marker4974	Chr. 7	30351967	T	T	A	T	A	Accurate
Marker4975	Chr. 7	30351992	C	C	A	C	A	Accurate
Marker4976	Chr. 7	30360251	G	G	A	G	A	Accurate
Marker4977	Chr. 7	30365884	G	G	A	G	A	Accurate
Marker4978	Chr. 7	30392123	A	A	G	A	G	Accurate
Marker4979	Chr. 7	30408584	T	T	A	T	A	Accurate
Marker4980	Chr. 7	30421289	A	A	G	A	G	Accurate
Marker4981	Chr. 7	30481541	A	G	A	G	A	Accurate
Marker4982	Chr. 7	30481737	G	C	G	C	G	Accurate
Marker4983	Chr. 7	30496089	T	T	A	T	A	Accurate
Marker4985	Chr. 7	30525568	C	A	C	A	C	Accurate
Marker4986	Chr. 7	30525569	G	A	G	A	G	Accurate
Marker4989	Chr. 7	30655067	C	C	G	C	G	Accurate
Marker4990	Chr. 7	30656172	A	A	G	A	G	Accurate
Marker4991	Chr. 7	30725978	A	A	G	A	G	Accurate
Marker4992	Chr. 7	30747651	T	C	T	C	T	Accurate
Marker4997	Chr. 7	30795626	T	C	T	C	N	Uncertain

Marker4999	Chr. 7	30797714	T	C	T	C	T	Accurate
Marker5000	Chr. 7	30797747	C	T	C	T	C	Accurate
Marker5001	Chr. 7	30873531	A	A	T	A	T	Accurate
Marker5002	Chr. 7	31048497	C	C	T	C	T	Accurate
Marker5003	Chr. 7	31077604	A	A	G	A	G	Accurate
Marker5004	Chr. 7	31077635	C	C	G	C	G	Accurate
Marker5005	Chr. 7	31107750	T	T	C	T	C	Accurate
Marker5006	Chr. 7	31107971	A	A	G	A	G	Accurate
Marker5007	Chr. 7	31111774	C	C	G	C	G	Accurate
Marker5009	Chr. 7	31191431	A	G	A	G	A	Accurate
Marker5010	Chr. 7	31208816	C	T	C	T	C	Accurate
Marker5012	Chr. 7	31278243	T	A	T	A	T	Accurate
Marker5014	Chr. 8	62245	G	T	G	T	G	Accurate
Marker5017	Chr. 8	96174	G	A	G	A	G	Accurate
Marker5021	Chr. 8	1563837	A	A	G	A	G	Accurate
Marker5022	Chr. 8	1575312	A	A	G	A	G	Accurate
Marker5023	Chr. 8	2573322	C	C	T	C	T	Accurate
Marker5024	Chr. 8	2578077	G	G	T	G	T	Accurate
Marker5025	Chr. 8	2586825	C	C	T	C	T	Accurate
Marker5026	Chr. 8	2612521	C	C	T	C	T	Accurate
Marker5027	Chr. 8	2612533	T	T	G	T	G	Accurate
Marker5028	Chr. 8	3145613	A	G	A	G	A	Accurate
Marker5029	Chr. 8	3149256	G	A	G	A	G	Accurate
Marker5031	Chr. 8	3174891	G	A	G	A	G	Accurate
Marker5032	Chr. 8	3175137	A	G	A	G	A	Accurate
Marker5033	Chr. 8	3175434	G	A	G	A	G	Accurate
Marker5034	Chr. 8	3175517	T	C	T	C	T	Accurate
Marker5035	Chr. 8	3194141	A	T	A	T	A	Accurate
Marker5036	Chr. 8	3226591	A	G	A	G	A	Accurate
Marker5037	Chr. 8	3227022	A	T	A	T	A	Accurate
Marker5039	Chr. 8	3241681	T	C	T	C	T	Accurate
Marker5041	Chr. 8	3251783	C	A	C	A	C	Accurate
Marker5042	Chr. 8	3278244	G	C	G	C	G	Accurate
Marker5044	Chr. 8	3278270	T	C	T	C	T	Accurate
Marker5045	Chr. 8	3278406	T	A	T	A	T	Accurate
Marker5046	Chr. 8	3330020	C	A	C	A	C	Accurate
Marker5048	Chr. 8	3565216	G	A	G	A	G	Accurate
Marker5049	Chr. 8	3647523	G	C	G	C	G	Accurate
Marker5057	Chr. 8	5046994	T	C	T	C	T	Accurate
Marker5058	Chr. 8	5047007	T	C	T	C	T	Accurate
Marker5059	Chr. 8	5057953	T	C	T	C	T	Accurate
Marker5061	Chr. 8	5073331	C	T	C	T	C	Accurate
Marker5064	Chr. 8	5094096	C	T	C	T	C	Accurate
Marker5065	Chr. 8	5094388	A	G	A	G	A	Accurate
Marker5068	Chr. 8	5101529	C	A	C	A	C	Accurate
Marker5072	Chr. 8	5162912	C	C	T	C	T	Accurate
Marker5073	Chr. 8	5180142	T	T	C	T	C	Accurate

Marker5074	Chr. 8	5201086	G	A	G	A	G	Accurate
Marker5075	Chr. 8	5227293	A	G	A	G	A	Accurate
Marker5076	Chr. 8	5227296	A	T	A	T	A	Accurate
Marker5077	Chr. 8	5233931	A	T	A	T	A	Accurate
Marker5080	Chr. 8	5235828	A	C	A	C	A	Accurate
Marker5082	Chr. 8	5235881	T	C	T	C	T	Accurate
Marker5086	Chr. 8	5240260	A	G	A	G	A	Accurate
Marker5088	Chr. 8	5240347	G	A	G	A	G	Accurate
Marker5090	Chr. 8	5240361	G	A	G	A	G	Accurate
Marker5092	Chr. 8	5245238	G	C	G	C	G	Accurate
Marker5093	Chr. 8	5256422	A	A	C	A	C	Accurate
Marker5097	Chr. 8	5294878	A	G	A	G	A	Accurate
Marker5098	Chr. 8	5294885	C	T	C	T	C	Accurate
Marker5105	Chr. 8	5682004	A	A	T	A	T	Accurate
Marker5106	Chr. 8	5709284	T	T	C	T	C	Accurate
Marker5107	Chr. 8	5709315	T	T	C	T	C	Accurate
Marker5108	Chr. 8	5709316	C	C	A	C	A	Accurate
Marker5109	Chr. 8	5709347	G	G	T	G	T	Accurate
Marker5110	Chr. 8	5709607	T	T	C	T	C	Accurate
Marker5111	Chr. 8	5711274	G	G	A	G	A	Accurate
Marker5112	Chr. 8	5711464	G	G	A	G	A	Accurate
Marker5114	Chr. 8	5721476	G	G	A	G	A	Accurate
Marker5115	Chr. 8	5721503	C	C	G	C	G	Accurate
Marker5117	Chr. 8	5724239	C	C	A	C	A	Accurate
Marker5119	Chr. 8	5850417	G	G	A	G	A	Accurate
Marker5120	Chr. 8	5858309	T	T	G	T	G	Accurate
Marker5124	Chr. 8	5895202	A	A	G	A	G	Accurate
Marker5125	Chr. 8	5895235	G	G	A	G	A	Accurate
Marker5127	Chr. 8	5908097	C	C	T	C	T	Accurate
Marker5130	Chr. 8	6003904	A	A	G	A	G	Accurate
Marker5131	Chr. 8	6003954	C	C	T	C	T	Accurate
Marker5132	Chr. 8	6058157	G	G	T	G	T	Accurate
Marker5133	Chr. 8	6074689	C	C	A	C	A	Accurate
Marker5141	Chr. 8	8806299	T	T	C	T	C	Accurate
Marker5142	Chr. 8	8806562	T	T	C	T	C	Accurate
Marker5143	Chr. 8	8806606	G	G	A	G	A	Accurate
Marker5144	Chr. 8	8843931	G	G	A	G	A	Accurate
Marker5145	Chr. 8	8848545	A	A	G	A	G	Accurate
Marker5147	Chr. 8	8871589	G	G	A	G	A	Accurate
Marker5148	Chr. 8	8882618	C	C	T	C	T	Accurate
Marker5151	Chr. 8	8902412	T	T	C	T	C	Accurate
Marker5153	Chr. 8	8909786	G	G	A	G	A	Accurate
Marker5155	Chr. 8	8911857	T	T	C	T	C	Accurate
Marker5156	Chr. 8	8912129	A	A	C	A	C	Accurate
Marker5157	Chr. 8	8914481	T	T	G	T	G	Accurate
Marker5159	Chr. 8	8940355	C	C	G	C	G	Accurate
Marker5160	Chr. 8	8955173	G	G	C	G	C	Accurate

Marker5163	Chr. 8	9002681	T	C	T	C	T	Accurate
Marker5164	Chr. 8	9002842	G	A	G	A	G	Accurate
Marker5166	Chr. 8	9021839	C	T	C	T	C	Accurate
Marker5167	Chr. 8	9030760	C	T	C	T	C	Accurate
Marker5170	Chr. 8	9044457	T	G	T	G	T	Accurate
Marker5171	Chr. 8	9056201	G	T	G	T	G	Accurate
Marker5172	Chr. 8	9058838	T	A	T	A	T	Accurate
Marker5175	Chr. 8	9089804	C	A	C	A	C	Accurate
Marker5177	Chr. 8	9097384	C	T	C	T	C	Accurate
Marker5179	Chr. 8	9112965	A	G	A	G	A	Accurate
Marker5184	Chr. 8	9146389	T	C	T	C	T	Accurate
Marker5185	Chr. 8	9146929	C	T	C	T	C	Accurate
Marker5186	Chr. 8	9146985	G	C	G	C	G	Accurate
Marker5192	Chr. 8	9450724	G	G	A	G	A	Accurate
Marker5193	Chr. 8	9450738	C	C	G	C	G	Accurate
Marker5194	Chr. 8	9450794	G	G	A	G	A	Accurate
Marker5197	Chr. 8	9628777	T	T	A	N	A	Uncertain
Marker5203	Chr. 8	9689307	A	A	G	A	G	Accurate
Marker5206	Chr. 8	9731631	G	G	T	G	T	Accurate
Marker5209	Chr. 8	9748332	G	G	A	G	A	Accurate
Marker5210	Chr. 8	9748384	G	G	T	G	T	Accurate
Marker5211	Chr. 8	9758235	A	A	G	A	G	Accurate
Marker5212	Chr. 8	9761244	C	C	A	C	A	Accurate
Marker5214	Chr. 8	9798315	G	G	T	G	T	Accurate
Marker5216	Chr. 8	9834075	G	G	A	G	A	Accurate
Marker5217	Chr. 8	9846216	A	A	G	A	G	Accurate
Marker5218	Chr. 8	9858771	G	G	A	G	A	Accurate
Marker5221	Chr. 8	10012839	A	A	G	A	G	Accurate
Marker5223	Chr. 8	10028619	G	G	A	G	A	Accurate
Marker5224	Chr. 8	10028628	G	G	T	G	T	Accurate
Marker5227	Chr. 8	10051259	G	G	A	G	A	Accurate
Marker5228	Chr. 8	10056345	A	A	G	A	G	Accurate
Marker5229	Chr. 8	10057191	T	T	C	T	C	Accurate
Marker5230	Chr. 8	10058454	C	C	G	C	G	Accurate
Marker5231	Chr. 8	10068081	T	T	C	T	C	Accurate
Marker5232	Chr. 8	10068098	T	T	G	T	K	Uncertain
Marker5235	Chr. 8	10075356	C	C	A	C	A	Accurate
Marker5236	Chr. 8	10075694	C	C	T	C	T	Accurate
Marker5237	Chr. 8	10080951	T	T	C	T	C	Accurate
Marker5240	Chr. 8	10239884	G	G	A	G	A	Accurate
Marker5241	Chr. 8	10242813	A	A	G	A	G	Accurate
Marker5246	Chr. 8	10670851	C	C	T	C	T	Accurate
Marker5248	Chr. 8	10703830	C	T	C	T	C	Accurate
Marker5249	Chr. 8	10723114	C	T	C	T	C	Accurate
Marker5250	Chr. 8	10723151	T	C	T	C	T	Accurate
Marker5251	Chr. 8	10764987	A	G	A	G	A	Accurate
Marker5252	Chr. 8	10765599	A	G	A	G	A	Accurate

Marker5254	Chr. 8	10785559	G	A	G	A	G	Accurate
Marker5255	Chr. 8	10785569	G	A	G	A	G	Accurate
Marker5259	Chr. 8	11088047	A	G	A	G	A	Accurate
Marker5260	Chr. 8	11088050	C	T	C	T	C	Accurate
Marker5261	Chr. 8	11093688	C	T	C	T	C	Accurate
Marker5262	Chr. 8	11093985	G	C	G	C	G	Accurate
Marker5263	Chr. 8	11108432	G	A	G	A	G	Accurate
Marker5264	Chr. 8	11595257	C	T	C	T	C	Accurate
Marker5265	Chr. 8	11605105	A	T	A	T	A	Accurate
Marker5267	Chr. 8	11737542	C	T	C	T	C	Accurate
Marker5269	Chr. 8	11848062	A	G	A	G	A	Accurate
Marker5270	Chr. 8	11848072	C	T	C	T	C	Accurate
Marker5271	Chr. 8	11848130	A	G	A	G	A	Accurate
Marker5272	Chr. 8	11848309	G	T	G	T	G	Accurate
Marker5273	Chr. 8	11878799	C	A	C	A	C	Accurate
Marker5274	Chr. 8	11880159	G	A	G	A	G	Accurate
Marker5275	Chr. 8	11900741	G	A	G	A	G	Accurate
Marker5278	Chr. 8	12102568	A	A	G	A	G	Accurate
Marker5280	Chr. 8	12134221	C	C	T	C	T	Accurate
Marker5281	Chr. 8	12135223	G	G	T	G	T	Accurate
Marker5283	Chr. 8	12162542	T	T	C	T	C	Accurate
Marker5287	Chr. 8	12230072	A	A	G	A	G	Accurate
Marker5288	Chr. 8	12272287	C	C	T	C	T	Accurate
Marker5290	Chr. 8	12349774	A	A	G	A	G	Accurate
Marker5293	Chr. 8	12415039	C	C	T	C	T	Accurate
Marker5294	Chr. 8	12625816	T	C	T	C	T	Accurate
Marker5295	Chr. 8	12649029	G	A	G	A	G	Accurate
Marker5298	Chr. 8	12706973	G	A	G	A	G	Accurate
Marker5299	Chr. 8	12739695	A	G	A	R	A	Uncertain
Marker5300	Chr. 8	12754252	G	C	G	C	G	Accurate
Marker5302	Chr. 8	12771000	A	G	A	G	A	Accurate
Marker5305	Chr. 8	12869134	A	A	T	A	W	Uncertain
Marker5306	Chr. 8	12869137	G	G	A	G	R	Uncertain
Marker5310	Chr. 8	12896279	T	T	C	T	C	Accurate
Marker5312	Chr. 8	12927107	A	A	G	A	G	Accurate
Marker5313	Chr. 8	12972750	T	T	C	T	C	Accurate
Marker5314	Chr. 8	12978781	G	G	A	G	A	Accurate
Marker5319	Chr. 8	13020845	C	C	T	C	T	Accurate
Marker5320	Chr. 8	13030457	T	T	A	T	A	Accurate
Marker5327	Chr. 8	13088642	G	G	A	G	A	Accurate
Marker5328	Chr. 8	13090942	A	A	G	A	G	Accurate
Marker5331	Chr. 8	13126976	A	A	G	A	G	Accurate
Marker5333	Chr. 8	13144885	A	A	T	N	T	Uncertain
Marker5335	Chr. 8	13154857	C	C	T	C	T	Accurate
Marker5336	Chr. 8	13155055	A	A	G	A	G	Accurate
Marker5338	Chr. 8	13158194	A	A	G	A	G	Accurate
Marker5339	Chr. 8	13158250	C	C	T	C	T	Accurate

Marker5340	Chr. 8	13264887	C	C	A	C	A	Accurate
Marker5341	Chr. 8	13293732	A	A	C	A	C	Accurate
Marker5348	Chr. 8	13348626	A	A	G	A	G	Accurate
Marker5349	Chr. 8	13357175	A	A	G	A	G	Accurate
Marker5350	Chr. 8	13374471	A	A	G	A	G	Accurate
Marker5358	Chr. 8	14310390	T	G	T	G	T	Accurate
Marker5359	Chr. 8	14313001	C	C	T	C	T	Accurate
Marker5362	Chr. 8	14320866	T	C	T	C	T	Accurate
Marker5366	Chr. 8	14382877	A	G	A	G	A	Accurate
Marker5369	Chr. 8	14419214	T	C	T	C	T	Accurate
Marker5370	Chr. 8	14419462	T	C	T	C	T	Accurate
Marker5371	Chr. 8	14421163	T	C	T	C	T	Accurate
Marker5372	Chr. 8	14421197	C	T	C	T	C	Accurate
Marker5378	Chr. 8	14500557	A	T	A	T	A	Accurate
Marker5379	Chr. 8	14582563	T	T	C	T	C	Accurate
Marker5381	Chr. 8	14873641	C	C	T	C	T	Accurate
Marker5382	Chr. 8	14873681	T	T	C	T	C	Accurate
Marker5383	Chr. 8	14894673	A	A	G	A	G	Accurate
Marker5384	Chr. 8	14895798	G	G	A	G	A	Accurate
Marker5387	Chr. 8	15682611	T	C	T	C	T	Accurate
Marker5388	Chr. 8	15682676	A	G	A	G	A	Accurate
Marker5390	Chr. 8	15705511	C	T	C	T	C	Accurate
Marker5391	Chr. 8	15717780	G	T	G	T	G	Accurate
Marker5393	Chr. 8	15737124	G	A	G	A	G	Accurate
Marker5398	Chr. 8	16422382	G	A	G	A	G	Accurate
Marker5399	Chr. 8	16455399	A	G	A	G	A	Accurate
Marker5407	Chr. 8	16608782	C	T	C	T	C	Accurate
Marker5408	Chr. 8	16610027	G	A	G	A	G	Accurate
Marker5412	Chr. 8	16691380	A	T	A	T	A	Accurate
Marker5413	Chr. 8	16739708	A	G	A	G	A	Accurate
Marker5415	Chr. 8	16751269	A	C	A	C	A	Accurate
Marker5422	Chr. 8	17150967	A	G	A	G	A	Accurate
Marker5424	Chr. 8	17233022	C	T	C	T	C	Accurate
Marker5426	Chr. 8	17320886	A	T	A	T	A	Accurate
Marker5427	Chr. 8	17320892	G	A	G	A	G	Accurate
Marker5428	Chr. 8	17348164	C	A	C	A	C	Accurate
Marker5430	Chr. 8	17426738	C	T	C	T	C	Accurate
Marker5433	Chr. 8	17458432	G	G	C	G	C	Accurate
Marker5434	Chr. 8	17464489	A	A	G	A	G	Accurate
Marker5435	Chr. 8	17464618	T	T	C	T	C	Accurate
Marker5438	Chr. 8	17481083	C	T	C	T	C	Accurate
Marker5439	Chr. 8	17481985	T	G	T	G	T	Accurate
Marker5444	Chr. 8	17483396	T	C	T	C	T	Accurate
Marker5448	Chr. 8	17742679	A	G	A	G	A	Accurate
Marker5454	Chr. 8	19162979	A	A	C	A	C	Accurate
Marker5455	Chr. 8	19219920	G	A	G	A	G	Accurate
Marker5457	Chr. 8	19733407	C	T	C	T	C	Accurate

Marker5460	Chr. 8	19760026	T	C	T	C	T	Accurate
Marker5462	Chr. 8	19762156	C	A	C	A	C	Accurate
Marker5464	Chr. 8	19802493	G	G	T	G	T	Accurate
Marker5466	Chr. 8	19815734	G	A	G	A	G	Accurate
Marker5472	Chr. 8	19884646	T	G	T	G	T	Accurate
Marker5475	Chr. 8	20076214	A	G	A	G	A	Accurate
Marker5478	Chr. 8	20198214	A	A	G	N	G	Uncertain
Marker5479	Chr. 8	20198391	T	T	A	T	A	Accurate
Marker5480	Chr. 8	20243520	C	A	C	A	C	Accurate
Marker5482	Chr. 8	20279076	C	C	T	C	T	Accurate
Marker5484	Chr. 8	20349749	G	T	G	T	G	Accurate
Marker5486	Chr. 8	20563148	T	C	T	C	T	Accurate
Marker5496	Chr. 8	21536950	T	C	T	C	T	Accurate
Marker5497	Chr. 8	21537217	C	T	C	T	C	Accurate
Marker5498	Chr. 8	21555734	A	G	A	G	A	Accurate
Marker5500	Chr. 8	21574576	G	A	G	A	G	Accurate
Marker5501	Chr. 8	21649352	A	G	A	G	A	Accurate
Marker5509	Chr. 8	22223235	C	C	G	C	G	Accurate
Marker5510	Chr. 8	22227427	T	T	C	T	C	Accurate
Marker5511	Chr. 8	22358074	A	A	G	A	G	Accurate
Marker5514	Chr. 8	22433553	G	T	G	T	G	Accurate
Marker5517	Chr. 8	22573402	T	G	T	G	T	Accurate
Marker5518	Chr. 8	22592938	T	C	T	C	T	Accurate
Marker5521	Chr. 8	22592948	T	A	T	C	C	Inconsistent
Marker5523	Chr. 8	22623558	T	A	T	A	T	Accurate
Marker5524	Chr. 8	22623859	A	G	A	G	A	Accurate
Marker5529	Chr. 8	22819214	C	T	C	T	C	Accurate
Marker5533	Chr. 8	23137790	T	T	C	T	C	Accurate
Marker5534	Chr. 8	23137936	C	C	T	C	T	Accurate
Marker5535	Chr. 8	23138790	G	G	A	G	A	Accurate
Marker5537	Chr. 8	23162409	G	G	A	G	A	Accurate
Marker5538	Chr. 8	23184363	A	A	G	A	G	Accurate
Marker5542	Chr. 8	23203907	T	T	C	T	C	Accurate
Marker5544	Chr. 8	23210277	A	A	G	N	G	Uncertain
Marker5545	Chr. 8	23212547	G	G	A	G	A	Accurate
Marker5547	Chr. 8	23212759	C	C	T	C	T	Accurate
Marker5549	Chr. 8	23223480	C	C	T	C	T	Accurate
Marker5550	Chr. 8	23235899	A	A	G	A	G	Accurate
Marker5555	Chr. 8	23391562	G	G	A	G	A	Accurate
Marker5562	Chr. 8	23634928	A	T	A	T	A	Accurate
Marker5563	Chr. 8	23654134	C	T	C	T	C	Accurate
Marker5565	Chr. 8	23749533	T	A	T	A	T	Accurate
Marker5569	Chr. 8	23914042	T	T	A	T	A	Accurate
Marker5570	Chr. 8	23914044	G	G	A	G	A	Accurate
Marker5571	Chr. 8	23938717	C	C	T	C	T	Accurate
Marker5573	Chr. 8	23984208	A	A	T	A	T	Accurate
Marker5577	Chr. 8	24148705	T	T	G	T	G	Accurate

Marker5579	Chr. 8	24172813	T	T	A	T	A	Accurate
Marker5580	Chr. 8	24173247	C	C	T	C	T	Accurate
Marker5581	Chr. 8	24173256	C	C	T	C	T	Accurate
Marker5584	Chr. 8	24349025	A	G	A	G	A	Accurate
Marker5588	Chr. 8	24368369	T	T	C	T	C	Accurate
Marker5589	Chr. 8	24372379	C	C	T	C	T	Accurate
Marker5590	Chr. 8	24372615	T	T	G	T	G	Accurate
Marker5591	Chr. 8	24379101	T	T	G	T	G	Accurate
Marker5593	Chr. 8	24383045	G	G	A	G	A	Accurate
Marker5594	Chr. 8	24383340	A	A	G	A	G	Accurate
Marker5595	Chr. 8	24437151	A	G	A	G	A	Accurate
Marker5598	Chr. 8	24475066	A	A	T	N	T	Uncertain
Marker5599	Chr. 8	24475511	T	T	A	T	A	Accurate
Marker5606	Chr. 8	24677857	T	T	C	N	C	Uncertain
Marker5610	Chr. 8	24768218	T	C	T	C	T	Accurate
Marker5613	Chr. 8	24820478	C	C	T	C	T	Accurate
Marker5616	Chr. 8	25247280	T	T	C	T	C	Accurate
Marker5619	Chr. 8	25288198	A	A	G	N	G	Uncertain
Marker5620	Chr. 8	25288204	A	A	G	N	G	Uncertain
Marker5621	Chr. 8	25321532	G	G	T	G	T	Accurate
Marker5622	Chr. 8	25323278	A	A	T	A	T	Accurate
Marker5623	Chr. 8	25323306	T	T	G	T	G	Accurate
Marker5625	Chr. 8	25329745	T	T	C	T	C	Accurate
Marker5629	Chr. 8	25465295	C	C	T	C	T	Accurate
Marker5630	Chr. 8	25470801	A	A	C	A	C	Accurate
Marker5632	Chr. 8	25524627	A	A	T	A	T	Accurate
Marker5636	Chr. 8	25693337	T	T	C	T	C	Accurate
Marker5637	Chr. 8	25762121	G	G	T	G	T	Accurate
Marker5638	Chr. 8	25762150	A	A	G	A	G	Accurate
Marker5641	Chr. 8	25947502	C	C	T	C	T	Accurate
Marker5642	Chr. 8	25953223	A	A	G	N	G	Uncertain
Marker5643	Chr. 9	5915	T	T	C	N	C	Uncertain
Marker5644	Chr. 9	6659	A	T	A	T	N	Uncertain
Marker5646	Chr. 9	173076	T	C	T	C	T	Accurate
Marker5647	Chr. 9	211179	A	A	G	A	G	Accurate
Marker5648	Chr. 9	216369	G	G	A	G	A	Accurate
Marker5649	Chr. 9	230683	T	T	C	T	C	Accurate
Marker5650	Chr. 9	239083	A	A	G	A	G	Accurate
Marker5651	Chr. 9	289398	G	G	A	G	A	Accurate
Marker5656	Chr. 9	308152	G	G	A	G	A	Accurate
Marker5658	Chr. 9	311200	T	T	C	T	C	Accurate
Marker5660	Chr. 9	602370	A	A	G	A	G	Accurate
Marker5672	Chr. 9	767636	T	C	T	C	T	Accurate
Marker5673	Chr. 9	767669	C	A	C	A	C	Accurate
Marker5679	Chr. 9	822408	A	C	A	C	A	Accurate
Marker5680	Chr. 9	822453	C	T	C	T	C	Accurate
Marker5681	Chr. 9	822454	C	T	C	T	C	Accurate

Marker5682	Chr. 9	822610	G	A	G	A	G	Accurate
Marker5683	Chr. 9	1096118	A	G	A	G	A	Accurate
Marker5684	Chr. 9	1106878	C	G	C	G	C	Accurate
Marker5685	Chr. 9	1116669	T	C	T	C	T	Accurate
Marker5686	Chr. 9	1134510	C	T	C	T	C	Accurate
Marker5687	Chr. 9	1143877	T	C	T	C	T	Accurate
Marker5689	Chr. 9	1178069	T	C	T	C	T	Accurate
Marker5690	Chr. 9	1187382	A	C	A	C	A	Accurate
Marker5691	Chr. 9	1191616	A	G	A	G	A	Accurate
Marker5692	Chr. 9	1191925	G	A	G	A	G	Accurate
Marker5694	Chr. 9	1198013	T	A	T	A	T	Accurate
Marker5695	Chr. 9	1203077	A	C	A	C	A	Accurate
Marker5696	Chr. 9	1211963	T	A	T	A	T	Accurate
Marker5700	Chr. 9	1283910	C	A	C	A	C	Accurate
Marker5701	Chr. 9	1298896	A	G	A	G	A	Accurate
Marker5703	Chr. 9	1531083	T	C	T	C	T	Accurate
Marker5705	Chr. 9	1544813	G	C	G	C	G	Accurate
Marker5706	Chr. 9	1617346	G	A	G	A	G	Accurate
Marker5707	Chr. 9	1617952	G	T	G	T	G	Accurate
Marker5708	Chr. 9	1633355	A	G	A	G	A	Accurate
Marker5710	Chr. 9	1645513	G	A	G	A	G	Accurate
Marker5711	Chr. 9	1658645	T	C	T	C	T	Accurate
Marker5712	Chr. 9	1664308	T	A	T	A	T	Accurate
Marker5713	Chr. 9	1692826	T	C	T	C	T	Accurate
Marker5714	Chr. 9	1754557	A	T	A	T	A	Accurate
Marker5715	Chr. 9	1754774	G	T	G	T	G	Accurate
Marker5716	Chr. 9	1754794	T	C	T	C	T	Accurate
Marker5718	Chr. 9	1826253	A	T	A	T	A	Accurate
Marker5720	Chr. 9	1868568	G	C	G	C	G	Accurate
Marker5722	Chr. 9	1903507	A	G	A	G	A	Accurate
Marker5723	Chr. 9	1924816	T	C	T	C	T	Accurate
Marker5724	Chr. 9	1924818	C	G	C	G	C	Accurate
Marker5725	Chr. 9	1925757	G	C	G	C	G	Accurate
Marker5727	Chr. 9	1968417	A	G	A	G	A	Accurate
Marker5728	Chr. 9	1989505	C	T	C	T	C	Accurate
Marker5729	Chr. 9	2020194	A	G	A	G	A	Accurate
Marker5730	Chr. 9	2022109	A	T	A	T	A	Accurate
Marker5731	Chr. 9	2022375	T	C	T	C	T	Accurate
Marker5732	Chr. 9	2056090	T	G	T	G	T	Accurate
Marker5733	Chr. 9	2063876	G	T	G	T	G	Accurate
Marker5734	Chr. 9	2064960	T	C	T	C	N	Uncertain
Marker5735	Chr. 9	2064962	T	G	T	G	N	Uncertain
Marker5736	Chr. 9	2065207	T	G	T	G	T	Accurate
Marker5739	Chr. 9	2268441	T	T	A	T	A	Accurate
Marker5740	Chr. 9	2282013	C	C	A	C	A	Accurate
Marker5741	Chr. 9	2284100	T	T	G	N	G	Uncertain
Marker5746	Chr. 9	2299441	G	G	A	G	A	Accurate

Marker5747	Chr. 9	2370699	A	A	T	A	T	Accurate
Marker5749	Chr. 9	2498857	A	A	G	A	G	Accurate
Marker5751	Chr. 9	2742451	C	C	T	C	T	Accurate
Marker5752	Chr. 9	2755655	T	T	C	T	C	Accurate
Marker5753	Chr. 9	2755703	C	C	T	C	T	Accurate
Marker5754	Chr. 9	2939428	C	T	C	T	C	Accurate
Marker5755	Chr. 9	2940684	G	T	G	T	G	Accurate
Marker5756	Chr. 9	2940707	G	A	G	A	G	Accurate
Marker5757	Chr. 9	2940724	C	T	C	T	C	Accurate
Marker5759	Chr. 9	2963164	G	T	G	T	G	Accurate
Marker5761	Chr. 9	3312447	T	C	T	C	T	Accurate
Marker5762	Chr. 9	3393229	A	A	G	A	G	Accurate
Marker5763	Chr. 9	3393435	A	A	T	A	T	Accurate
Marker5764	Chr. 9	3397153	C	C	G	C	G	Accurate
Marker5765	Chr. 9	3397322	C	C	A	C	A	Accurate
Marker5766	Chr. 9	3397352	A	A	C	A	C	Accurate
Marker5767	Chr. 9	3397586	G	G	A	G	A	Accurate
Marker5770	Chr. 9	3434854	A	A	G	A	G	Accurate
Marker5772	Chr. 9	3448723	G	G	T	G	T	Accurate
Marker5775	Chr. 9	3489559	A	A	G	A	G	Accurate
Marker5779	Chr. 9	3591247	A	A	T	A	T	Accurate
Marker5780	Chr. 9	3592588	A	A	G	A	G	Accurate
Marker5783	Chr. 9	3636983	G	G	A	G	A	Accurate
Marker5784	Chr. 9	3666105	C	C	T	C	T	Accurate
Marker5786	Chr. 9	3728381	G	G	A	G	A	Accurate
Marker5791	Chr. 9	3821921	C	C	G	C	G	Accurate
Marker5792	Chr. 9	3840899	G	G	T	G	T	Accurate
Marker5797	Chr. 9	3919708	C	C	T	C	T	Accurate
Marker5798	Chr. 9	3932851	C	C	A	C	A	Accurate
Marker5799	Chr. 9	3932868	T	T	C	T	C	Accurate
Marker5801	Chr. 9	3932886	A	A	G	A	G	Accurate
Marker5803	Chr. 9	4000145	A	A	G	A	G	Accurate
Marker5808	Chr. 9	4214944	C	C	T	C	T	Accurate
Marker5809	Chr. 9	4245529	C	C	T	C	T	Accurate
Marker5811	Chr. 9	4292751	C	C	T	C	T	Accurate
Marker5812	Chr. 9	4292933	T	T	C	T	C	Accurate
Marker5816	Chr. 9	4772631	C	C	T	C	T	Accurate
Marker5817	Chr. 9	4775922	A	T	A	T	A	Accurate
Marker5818	Chr. 9	4797339	A	T	A	T	A	Accurate
Marker5819	Chr. 9	4852629	G	T	G	T	G	Accurate
Marker5821	Chr. 9	4960739	G	G	A	G	A	Accurate
Marker5822	Chr. 9	4963441	G	C	G	C	G	Accurate
Marker5823	Chr. 9	4963442	C	T	C	T	C	Accurate
Marker5825	Chr. 9	4984257	G	A	G	A	G	Accurate
Marker5826	Chr. 9	4991326	A	C	A	C	A	Accurate
Marker5831	Chr. 9	5596643	C	C	A	C	A	Accurate
Marker5832	Chr. 9	5649677	C	T	C	Y	C	Uncertain

Marker5833	Chr. 9	5651185	G	A	G	A	G	Accurate
Marker5834	Chr. 9	5864051	C	C	T	N	T	Uncertain
Marker5835	Chr. 9	5875762	C	T	C	T	C	Accurate
Marker5836	Chr. 9	5918233	G	A	G	A	G	Accurate
Marker5837	Chr. 9	6060043	C	C	T	N	T	Uncertain
Marker5838	Chr. 9	6060591	A	A	G	A	G	Accurate
Marker5839	Chr. 9	6062263	C	C	A	C	A	Accurate
Marker5840	Chr. 9	6062284	G	G	A	G	A	Accurate
Marker5841	Chr. 9	6062804	T	T	A	T	A	Accurate
Marker5842	Chr. 9	6066187	C	T	C	T	C	Accurate
Marker5843	Chr. 9	6105294	T	T	C	T	C	Accurate
Marker5845	Chr. 9	6111434	G	G	T	G	T	Accurate
Marker5846	Chr. 9	6124451	T	T	C	T	C	Accurate
Marker5847	Chr. 9	6124696	C	C	G	C	G	Accurate
Marker5848	Chr. 9	6128193	G	G	T	G	T	Accurate
Marker5849	Chr. 9	6129604	C	C	T	C	T	Accurate
Marker5851	Chr. 9	6145597	T	T	A	T	A	Accurate
Marker5852	Chr. 9	6201109	A	A	T	A	T	Accurate
Marker5853	Chr. 9	6264652	G	G	A	G	A	Accurate
Marker5854	Chr. 9	6266749	A	A	C	A	C	Accurate
Marker5856	Chr. 9	6317141	T	C	T	C	T	Accurate
Marker5857	Chr. 9	6348451	A	G	A	G	A	Accurate
Marker5858	Chr. 9	6350734	G	C	G	C	G	Accurate
Marker5859	Chr. 9	6354188	C	T	C	T	C	Accurate
Marker5860	Chr. 9	6361153	T	C	T	C	T	Accurate
Marker5861	Chr. 9	6361544	A	G	A	G	A	Accurate
Marker5862	Chr. 9	6497511	C	A	C	A	C	Accurate
Marker5863	Chr. 9	6503228	A	A	C	A	C	Accurate
Marker5864	Chr. 9	6512808	T	T	C	T	C	Accurate
Marker5868	Chr. 9	6897675	G	G	A	G	A	Accurate
Marker5869	Chr. 9	6908643	C	C	T	C	T	Accurate
Marker5870	Chr. 9	6908929	A	A	G	A	G	Accurate
Marker5871	Chr. 9	6927249	A	A	G	A	G	Accurate
Marker5872	Chr. 9	6935396	T	T	G	T	G	Accurate
Marker5873	Chr. 9	6935672	C	C	A	C	A	Accurate
Marker5875	Chr. 9	7013294	A	T	A	W	A	Uncertain
Marker5876	Chr. 9	7013309	A	G	A	R	A	Uncertain
Marker5877	Chr. 9	7019602	C	T	C	T	C	Accurate
Marker5879	Chr. 9	7026067	C	T	C	T	C	Accurate
Marker5881	Chr. 9	7108956	C	C	T	C	T	Accurate
Marker5882	Chr. 9	7110898	C	C	T	C	T	Accurate
Marker5883	Chr. 9	7140089	T	T	G	T	G	Accurate
Marker5884	Chr. 9	7140422	C	C	T	C	T	Accurate
Marker5886	Chr. 9	7506387	C	C	A	C	A	Accurate
Marker5892	Chr. 9	7531602	C	T	C	T	C	Accurate
Marker5896	Chr. 9	7723134	A	A	G	A	G	Accurate
Marker5897	Chr. 9	7723172	G	G	A	G	A	Accurate

Marker5899	Chr. 9	7725750	A	A	T	A	T	Accurate
Marker5901	Chr. 9	7728311	C	C	A	N	A	Uncertain
Marker5904	Chr. 9	7764238	A	A	G	A	G	Accurate
Marker5907	Chr. 9	7788579	G	G	T	G	T	Accurate
Marker5911	Chr. 9	7831249	T	T	G	T	G	Accurate
Marker5912	Chr. 9	7831357	C	C	A	C	A	Accurate
Marker5914	Chr. 9	7858132	A	A	G	N	G	Uncertain
Marker5915	Chr. 9	7918071	C	C	A	C	A	Accurate
Marker5916	Chr. 9	7939882	T	T	A	T	A	Accurate
Marker5917	Chr. 9	7966216	T	T	A	T	A	Accurate
Marker5920	Chr. 9	8333087	T	T	C	T	C	Accurate
Marker5921	Chr. 9	8347943	T	T	G	T	G	Accurate
Marker5922	Chr. 9	8369726	C	C	T	C	T	Accurate
Marker5923	Chr. 9	8476317	G	G	T	G	T	Accurate
Marker5925	Chr. 9	8490612	C	C	A	C	A	Accurate
Marker5926	Chr. 9	8490832	A	A	G	N	G	Uncertain
Marker5927	Chr. 9	8494834	G	G	A	G	A	Accurate
Marker5928	Chr. 9	8496228	G	G	A	G	A	Accurate
Marker5931	Chr. 9	9308513	A	T	A	T	A	Accurate
Marker5939	Chr. 9	9418773	C	T	C	T	C	Accurate
Marker5941	Chr. 9	9665908	C	T	C	T	C	Accurate
Marker5942	Chr. 9	9665912	T	A	T	A	T	Accurate
Marker5949	Chr. 9	9950936	C	T	C	T	C	Accurate
Marker5952	Chr. 9	10056252	A	G	A	G	A	Accurate
Marker5953	Chr. 9	10076243	C	A	C	A	C	Accurate
Marker5955	Chr. 9	10409998	C	T	C	T	C	Accurate
Marker5961	Chr. 9	10562261	A	G	A	G	A	Accurate
Marker5966	Chr. 9	10629539	C	T	C	T	C	Accurate
Marker5972	Chr. 9	10657308	G	A	T	A	A	Inconsistent
Marker5976	Chr. 9	10837358	C	C	A	C	A	Accurate
Marker5987	Chr. 9	11227885	T	C	T	C	T	Accurate
Marker5988	Chr. 9	11227896	A	G	A	G	A	Accurate
Marker5993	Chr. 9	11570934	G	A	G	A	G	Accurate
Marker6001	Chr. 9	11615621	C	T	C	T	C	Accurate
Marker6003	Chr. 9	11648637	C	T	C	T	C	Accurate
Marker6007	Chr. 9	11726711	C	G	C	G	C	Accurate
Marker6008	Chr. 9	11813328	C	T	C	T	C	Accurate
Marker6009	Chr. 9	11813337	C	T	C	T	C	Accurate
Marker6010	Chr. 9	11826867	A	C	A	C	A	Accurate
Marker6013	Chr. 9	11831171	G	A	G	A	G	Accurate
Marker6014	Chr. 9	11840572	C	A	C	A	C	Accurate
Marker6016	Chr. 9	11851262	A	G	A	G	A	Accurate
Marker6017	Chr. 9	11877025	C	T	C	T	C	Accurate
Marker6018	Chr. 9	11887491	A	G	A	G	A	Accurate
Marker6020	Chr. 9	11887650	G	A	G	A	G	Accurate
Marker6023	Chr. 9	11956378	G	A	G	A	G	Accurate
Marker6024	Chr. 9	11956537	A	G	A	G	A	Accurate

Marker6026	Chr. 9	11975773	A	G	A	G	A	Accurate
Marker6027	Chr. 9	11983548	C	T	C	T	C	Accurate
Marker6028	Chr. 9	12004443	G	A	G	A	G	Accurate
Marker6039	Chr. 9	12624373	T	G	T	G	T	Accurate
Marker6042	Chr. 9	12671114	C	T	C	T	C	Accurate
Marker6043	Chr. 9	12691071	G	A	G	A	G	Accurate
Marker6046	Chr. 9	12721053	G	T	G	K	G	Uncertain
Marker6052	Chr. 9	12748954	A	G	A	G	A	Accurate
Marker6054	Chr. 9	13221067	C	A	C	A	C	Accurate
Marker6055	Chr. 9	13425311	C	T	C	T	C	Accurate
Marker6056	Chr. 9	13588394	G	A	G	A	N	Uncertain
Marker6063	Chr. 9	14058844	T	C	T	C	T	Accurate
Marker6065	Chr. 9	14265537	A	G	A	G	A	Accurate
Marker6066	Chr. 9	14267882	G	A	G	A	G	Accurate
Marker6075	Chr. 9	14495130	A	G	A	G	A	Accurate
Marker6079	Chr. 9	15317651	C	G	C	G	C	Accurate
Marker6081	Chr. 9	15386782	G	A	G	A	G	Accurate
Marker6082	Chr. 9	15390819	T	C	T	C	T	Accurate
Marker6085	Chr. 9	15461807	T	C	T	C	T	Accurate
Marker6086	Chr. 9	15461815	G	A	G	A	G	Accurate
Marker6087	Chr. 9	15479636	A	C	A	C	A	Accurate
Marker6088	Chr. 9	15479910	T	C	T	C	T	Accurate
Marker6089	Chr. 9	15498268	A	G	A	G	A	Accurate
Marker6094	Chr. 9	15614857	C	T	C	T	C	Accurate
Marker6095	Chr. 9	15614901	G	A	G	A	G	Accurate
Marker6099	Chr. 9	15655408	C	T	C	T	C	Accurate
Marker6100	Chr. 9	15655425	T	C	T	C	T	Accurate
Marker6101	Chr. 9	15657219	A	G	A	G	A	Accurate
Marker6102	Chr. 9	15657235	T	C	T	C	T	Accurate
Marker6103	Chr. 9	15657729	A	A	C	A	C	Accurate
Marker6107	Chr. 9	15667529	T	C	T	C	T	Accurate
Marker6108	Chr. 9	15667732	G	C	G	C	G	Accurate
Marker6113	Chr. 9	15738258	C	T	C	T	C	Accurate
Marker6114	Chr. 9	15771854	A	G	A	R	A	Uncertain
Marker6115	Chr. 9	15774459	A	G	A	G	A	Accurate
Marker6118	Chr. 9	15805493	G	A	G	A	G	Accurate
Marker6119	Chr. 9	15819642	A	G	A	G	A	Accurate
Marker6120	Chr. 9	15833321	C	G	C	G	C	Accurate
Marker6123	Chr. 9	15860986	T	C	T	C	T	Accurate
Marker6126	Chr. 9	15920506	A	G	A	G	N	Uncertain
Marker6133	Chr. 9	15982391	A	T	A	T	A	Accurate
Marker6136	Chr. 9	16047191	T	C	T	C	T	Accurate
Marker6137	Chr. 9	16080100	A	A	G	N	G	Uncertain
Marker6139	Chr. 9	16125597	T	C	T	C	T	Accurate
Marker6140	Chr. 9	16130838	T	A	T	A	T	Accurate
Marker6142	Chr. 9	16213000	A	G	A	G	R	Uncertain
Marker6144	Chr. 9	16225370	A	T	A	T	A	Accurate

Marker6147	Chr. 9	16315754	T	C	T	C	T	Accurate
Marker6148	Chr. 9	16315986	A	G	A	G	A	Accurate
Marker6157	Chr. 9	16864528	G	A	G	A	G	Accurate
Marker6158	Chr. 9	16864552	C	G	C	G	C	Accurate
Marker6159	Chr. 9	16867536	T	C	T	C	T	Accurate
Marker6160	Chr. 9	16867570	A	G	A	G	A	Accurate
Marker6161	Chr. 9	16867636	T	C	T	C	T	Accurate
Marker6163	Chr. 9	16897786	C	T	C	T	C	Accurate
Marker6164	Chr. 9	16908557	G	A	G	A	G	Accurate
Marker6166	Chr. 9	16946595	T	C	T	C	T	Accurate
Marker6167	Chr. 9	16952333	C	T	C	T	C	Accurate
Marker6168	Chr. 9	17015216	G	T	G	T	G	Accurate
Marker6172	Chr. 9	17056205	A	G	A	G	A	Accurate
Marker6174	Chr. 9	17064326	T	C	T	C	T	Accurate
Marker6175	Chr. 9	17064389	G	A	G	A	G	Accurate
Marker6176	Chr. 9	17069950	C	T	C	T	C	Accurate
Marker6179	Chr. 9	17086258	G	A	G	A	G	Accurate
Marker6180	Chr. 9	17088571	T	C	T	C	T	Accurate
Marker6182	Chr. 9	17122294	T	C	T	C	T	Accurate
Marker6184	Chr. 9	17172448	C	T	C	T	C	Accurate
Marker6189	Chr. 9	17179884	A	C	A	C	A	Accurate
Marker6190	Chr. 9	17190491	G	A	G	A	G	Accurate
Marker6191	Chr. 9	17195480	G	A	G	A	G	Accurate
Marker6193	Chr. 9	17215841	G	A	G	A	G	Accurate
Marker6194	Chr. 9	17216432	C	T	C	T	C	Accurate
Marker6195	Chr. 9	17216472	T	G	T	G	T	Accurate
Marker6196	Chr. 9	17242229	T	C	T	C	T	Accurate
Marker6197	Chr. 9	17249805	C	T	C	T	C	Accurate
Marker6198	Chr. 9	17264965	T	C	T	C	T	Accurate
Marker6199	Chr. 9	17265138	G	A	G	A	G	Accurate
Marker6200	Chr. 9	17292425	T	C	T	C	T	Accurate
Marker6203	Chr. 9	17320747	G	A	G	A	G	Accurate
Marker6206	Chr. 9	17322839	G	A	G	A	G	Accurate
Marker6207	Chr. 9	17323604	C	T	C	T	C	Accurate
Marker6211	Chr. 9	17349695	T	C	T	C	T	Accurate
Marker6212	Chr. 9	17352293	T	G	T	G	T	Accurate
Marker6213	Chr. 9	17352318	C	G	C	G	C	Accurate
Marker6218	Chr. 9	17433571	C	T	C	T	C	Accurate
Marker6220	Chr. 9	17462305	T	C	T	C	T	Accurate
Marker6227	Chr. 9	17508449	T	G	T	G	T	Accurate
Marker6228	Chr. 9	17516966	C	A	C	A	C	Accurate
Marker6231	Chr. 9	18139737	T	C	T	C	T	Accurate
Marker6233	Chr. 9	18146838	T	A	T	A	T	Accurate
Marker6235	Chr. 9	18178544	G	A	G	A	G	Accurate
Marker6237	Chr. 9	18206488	A	G	A	G	A	Accurate
Marker6238	Chr. 9	18207790	G	A	G	A	G	Accurate
Marker6239	Chr. 9	18216501	G	T	G	T	G	Accurate

Marker6240	Chr. 9	18220174	T	C	T	C	T	Accurate
Marker6241	Chr. 9	18290228	C	T	C	T	C	Accurate
Marker6242	Chr. 9	18336192	C	G	C	G	C	Accurate
Marker6245	Chr. 9	18930152	T	A	T	A	T	Accurate
Marker6247	Chr. 9	19102487	C	T	C	T	C	Accurate
Marker6248	Chr. 9	19179252	G	A	G	A	G	Accurate
Marker6249	Chr. 9	19193187	G	A	G	A	G	Accurate
Marker6251	Chr. 9	19315729	G	A	G	A	G	Accurate
Marker6252	Chr. 9	19650929	G	T	G	T	G	Accurate
Marker6253	Chr. 9	19653386	G	A	G	A	G	Accurate
Marker6254	Chr. 9	19705139	C	T	C	T	C	Accurate
Marker6255	Chr. 9	19705165	T	C	T	C	T	Accurate
Marker6260	Chr. 9	19755828	C	T	C	T	C	Accurate
Marker6265	Chr. 9	19770410	G	A	G	A	G	Accurate
Marker6266	Chr. 9	19770511	C	T	C	T	C	Accurate
Marker6267	Chr. 9	20679441	A	A	G	A	G	Accurate
Marker6270	Chr. 9	20778161	T	T	C	T	C	Accurate
Marker6272	Chr. 9	20789934	T	T	C	T	C	Accurate
Marker6279	Chr. 9	21226263	A	T	A	T	A	Accurate
Marker6282	Chr. 9	21292707	G	T	G	T	G	Accurate
Marker6283	Chr. 9	21292964	T	C	T	C	T	Accurate
Marker6295	Chr. 9	21744168	A	T	A	A	G	Inconsistent
Marker6299	Chr. 9	21789360	G	G	T	G	T	Accurate
Marker6300	Chr. 9	21798733	T	T	C	T	C	Accurate
Marker6301	Chr. 9	21802045	A	A	T	A	T	Accurate
Marker6303	Chr. 9	21807828	T	T	A	T	A	Accurate
Marker6304	Chr. 9	21811814	A	A	C	A	C	Accurate
Marker6306	Chr. 9	21853494	A	A	G	A	G	Accurate
Marker6310	Chr. 9	21855275	C	C	T	C	T	Accurate
Marker6313	Chr. 9	21913419	G	G	A	G	A	Accurate
Marker6315	Chr. 9	21970353	A	A	G	A	G	Accurate
Marker6318	Chr. 9	22001219	T	T	A	T	A	Accurate
Marker6319	Chr. 9	22029968	T	T	A	T	A	Accurate
Marker6323	Chr. 9	22052335	G	G	A	G	A	Accurate
Marker6325	Chr. 9	22685091	G	G	A	G	A	Accurate
Marker6326	Chr. 9	22694640	C	C	T	C	T	Accurate
Marker6331	Chr. 9	22722315	C	C	T	C	T	Accurate
Marker6334	Chr. 9	22981772	T	T	A	T	A	Accurate
Marker6338	Chr. 9	23276437	G	G	A	G	A	Accurate
Marker6339	Chr. 9	23378369	G	A	G	A	G	Accurate
Marker6340	Chr. 9	23438371	A	A	G	A	G	Accurate
Marker6342	Chr. 9	23446953	T	T	C	T	C	Accurate
Marker6343	Chr. 9	23447171	C	C	T	C	T	Accurate
Marker6345	Chr. 9	23452824	T	T	C	T	C	Accurate
Marker6352	Chr. 9	23705560	G	A	G	A	G	Accurate
Marker6353	Chr. 9	23708369	G	A	G	A	G	Accurate
Marker6355	Chr. 9	23724478	C	T	C	T	C	Accurate

Marker6356	Chr. 9	23729420	C	T	C	T	C	Accurate
Marker6357	Chr. 9	23729421	C	T	C	T	C	Accurate
Marker6358	Chr. 9	23732293	T	G	T	G	T	Accurate
Marker6360	Chr. 9	23741776	G	A	G	A	G	Accurate
Marker6361	Chr. 9	23767212	C	C	G	C	G	Accurate
Marker6362	Chr. 9	23767235	G	G	A	G	A	Accurate
Marker6363	Chr. 9	23859154	T	T	C	T	C	Accurate
Marker6367	Chr. 9	23943440	C	C	G	C	G	Accurate
Marker6373	Chr. 9	24168005	C	C	T	C	T	Accurate
Marker6374	Chr. 9	24183556	G	G	A	G	A	Accurate
Marker6375	Chr. 9	24193864	C	C	T	C	T	Accurate
Marker6378	Chr. 9	24238949	G	G	A	G	A	Accurate
Marker6381	Chr. 9	24300221	T	T	A	T	A	Accurate
Marker6382	Chr. 9	24300496	C	C	T	C	T	Accurate
Marker6383	Chr. 9	24311809	C	C	T	C	T	Accurate
Marker6384	Chr. 9	24312098	A	A	G	A	G	Accurate
Marker6392	Chr. 9	24692101	A	A	G	A	G	Accurate
Marker6395	Chr. 9	24702404	C	C	T	C	T	Accurate
Marker6399	Chr. 9	24792703	C	C	T	C	T	Accurate
Marker6402	Chr. 9	24870037	C	T	C	T	C	Accurate
Marker6403	Chr. 9	24898851	G	A	G	A	G	Accurate
Marker6404	Chr. 9	24898852	A	G	A	G	A	Accurate
Marker6407	Chr. 9	24904488	C	A	C	A	C	Accurate
Marker6410	Chr. 9	25114198	A	G	A	G	A	Accurate
Marker6411	Chr. 9	25244265	G	A	G	A	G	Accurate
Marker6412	Chr. 9	25355850	C	T	C	T	C	Accurate
Marker6413	Chr. 9	25555855	C	C	T	C	T	Accurate
Marker6414	Chr. 9	25611614	T	T	C	T	C	Accurate
Marker6421	Chr. 9	25933840	C	C	T	C	T	Accurate
Marker6422	Chr. 9	25934114	G	G	A	G	A	Accurate
Marker6423	Chr. 9	25941047	T	T	G	T	G	Accurate
Marker6428	Chr. 9	26037278	T	T	A	T	A	Accurate
Marker6436	Chr. 9	26157257	T	A	T	A	T	Accurate
Marker6438	Chr. 9	26182016	C	C	T	C	T	Accurate
Marker6441	Chr. 9	26202232	C	C	T	N	T	Uncertain
Marker6443	Chr. 9	26257089	A	T	A	T	A	Accurate
Marker6445	Chr. 9	26303639	C	T	C	T	C	Accurate
Marker6447	Chr. 9	26322360	C	T	C	T	C	Accurate
Marker6452	Chr. 9	26503707	C	T	C	Y	C	Uncertain
Marker6453	Chr. 9	26619487	G	A	G	A	G	Accurate
Marker6455	Chr. 9	26650753	C	G	C	G	C	Accurate
Marker6458	Chr. 9	26662193	G	A	G	A	G	Accurate
Marker6459	Chr. 9	26711686	T	G	T	G	T	Accurate
Marker6460	Chr. 9	26714393	T	C	T	C	T	Accurate
Marker6462	Chr. 9	26730771	T	C	T	C	T	Accurate
Marker6463	Chr. 9	26741521	T	C	T	C	T	Accurate
Marker6469	Chr. 9	27059158	A	A	C	A	C	Accurate

Marker6470	Chr. 9	27077911	T	T	C	T	C	Accurate
Marker6472	Chr. 9	27092489	C	C	A	C	A	Accurate
Marker6473	Chr. 9	27097240	A	A	G	A	G	Accurate
Marker6475	Chr. 9	27098219	T	T	C	T	C	Accurate
Marker6476	Chr. 9	27124972	G	G	A	G	A	Accurate
Marker6477	Chr. 9	27124982	T	T	C	T	C	Accurate
Marker6479	Chr. 9	27132715	A	G	A	G	A	Accurate
Marker6480	Chr. 9	27150620	A	G	A	G	A	Accurate
Marker6482	Chr. 9	27169068	A	G	A	G	A	Accurate
Marker6484	Chr. 9	27182941	C	T	C	T	C	Accurate
Marker6488	Chr. 9	27198241	T	C	T	C	T	Accurate
Marker6494	Chr. 9	27378419	C	T	C	T	C	Accurate
Marker6495	Chr. 9	27400255	T	C	T	C	T	Accurate
Marker6496	Chr. 9	27410822	G	T	G	T	G	Accurate
Marker6497	Chr. 9	27410858	T	G	T	G	T	Accurate
Marker6498	Chr. 9	27417756	G	T	G	T	G	Accurate
Marker6499	Chr. 9	27417763	G	A	G	A	G	Accurate
Marker6500	Chr. 9	27425596	C	T	C	T	C	Accurate
Marker6501	Chr. 9	27446727	A	G	A	G	A	Accurate
Marker6502	Chr. 9	27446763	G	T	G	T	G	Accurate
Marker6503	Chr. 9	27446885	T	C	T	C	T	Accurate
Marker6504	Chr. 9	27446938	A	G	A	G	A	Accurate
Marker6505	Chr. 9	27446982	T	C	T	C	T	Accurate
Marker6507	Chr. 9	27447238	A	G	A	G	A	Accurate
Marker6508	Chr. 9	27447551	A	T	A	T	A	Accurate
Marker6513	Chr. 9	27518332	G	A	G	A	G	Accurate
Marker6515	Chr. 9	27518593	C	A	C	A	C	Accurate
Marker6517	Chr. 9	27533842	G	A	G	A	G	Accurate
Marker6518	Chr. 9	27533905	T	C	T	C	T	Accurate
Marker6519	Chr. 9	27540551	T	C	T	C	T	Accurate
Marker6520	Chr. 9	27540605	G	A	G	A	G	Accurate
Marker6521	Chr. 9	27540767	G	C	G	C	G	Accurate
Marker6522	Chr. 9	27540783	T	C	T	C	T	Accurate
Marker6523	Chr. 9	27540858	G	A	G	A	G	Accurate
Marker6524	Chr. 9	27540889	A	G	A	G	A	Accurate
Marker6525	Chr. 9	27551229	C	T	C	T	C	Accurate
Marker6526	Chr. 9	27558180	G	A	G	A	G	Accurate
Marker6528	Chr. 9	27559797	C	T	C	T	C	Accurate
Marker6530	Chr. 9	27580565	G	T	G	T	G	Accurate
Marker6531	Chr. 9	27582826	T	C	T	C	T	Accurate
Marker6533	Chr. 9	27605985	C	T	C	T	C	Accurate
Marker6534	Chr. 9	27614772	T	C	T	C	T	Accurate
Marker6537	Chr. 9	27643497	C	T	C	T	C	Accurate
Marker6538	Chr. 9	27655841	A	C	A	C	A	Accurate
Marker6539	Chr. 9	27663986	G	A	G	A	G	Accurate
Marker6542	Chr. 9	27749619	T	C	T	C	T	Accurate
Marker6543	Chr. 9	27749631	A	G	A	G	A	Accurate

Marker6544	Chr. 9	27749843	T	C	T	C	T	Accurate
Marker6545	Chr. 9	27761593	T	G	T	G	T	Accurate
Marker6546	Chr. 9	27777072	A	G	A	G	A	Accurate
Marker6547	Chr. 9	27778265	G	A	G	A	G	Accurate
Marker6553	Chr. 9	27845347	G	A	G	A	G	Accurate
Marker6557	Chr. 9	27887876	G	C	G	C	G	Accurate
Marker6558	Chr. 9	27899837	T	C	T	C	T	Accurate
Marker6560	Chr. 9	27932055	C	T	C	T	C	Accurate
Marker6562	Chr. 9	27934116	C	T	C	T	C	Accurate
Marker6566	Chr. 9	27951362	A	G	A	G	A	Accurate
Marker6567	Chr. 9	27951669	A	G	A	G	A	Accurate
Marker6571	Chr. 9	28001759	G	A	G	A	G	Accurate
Marker6572	Chr. 9	28002888	T	C	T	C	T	Accurate
Marker6573	Chr. 9	28002889	T	C	T	C	T	Accurate
Marker6574	Chr. 9	28003058	A	G	A	G	A	Accurate
Marker6578	Chr. 9	28141284	C	T	C	T	C	Accurate
Marker6579	Chr. 9	28147960	T	C	T	C	T	Accurate
Marker6580	Chr. 9	28148205	C	T	C	T	C	Accurate
Marker6582	Chr. 9	28170623	G	A	G	A	G	Accurate
Marker6584	Chr. 9	28231483	A	G	A	G	A	Accurate
Marker6585	Chr. 9	28235365	A	G	A	G	A	Accurate
Marker6590	Chr. 9	29419730	T	T	C	T	C	Accurate
Marker6594	Chr. 9	29486621	G	A	G	A	G	Accurate
Marker6601	Chr. 9	29533955	G	A	G	A	G	Accurate
Marker6604	Chr. 9	29558395	A	A	G	A	G	Accurate
Marker6606	Chr. 9	29582597	G	G	A	G	A	Accurate
Marker6614	Chr. 9	31000702	G	G	A	G	A	Accurate
Marker6618	Chr. 9	31042762	T	C	T	C	T	Accurate
Marker6619	Chr. 9	31042855	G	A	G	A	G	Accurate
Marker6620	Chr. 9	31042860	G	A	G	A	G	Accurate
Marker6621	Chr. 9	31042870	T	C	T	C	T	Accurate
Marker6631	Chr. 9	31097819	G	G	A	G	A	Accurate
Marker6633	Chr. 9	31102843	A	A	G	N	G	Uncertain
Marker6645	Chr. 9	32026324	G	A	G	A	G	Accurate
Marker6646	Chr. 9	32039911	C	G	C	G	C	Accurate
Marker6647	Chr. 9	32304685	T	C	T	C	T	Accurate
Marker6648	Chr. 9	32320376	G	A	G	A	G	Accurate
Marker6649	Chr. 9	32320652	A	G	A	G	A	Accurate
Marker6650	Chr. 9	32770337	C	T	C	T	C	Accurate
Marker6651	Chr. 9	32809834	A	C	A	C	A	Accurate
Marker6652	Chr. 9	32809874	A	G	A	G	A	Accurate
Marker6656	Chr. 9	32982325	T	A	T	A	T	Accurate
Marker6657	Chr. 9	32997882	C	C	T	C	T	Accurate
Marker6665	Chr. 9	33078757	G	A	G	A	G	Accurate
Marker6667	Chr. 9	33119999	T	G	T	G	T	Accurate
Marker6670	Chr. 9	33520151	C	C	A	C	A	Accurate
Marker6674	Chr. 9	33627070	A	C	A	C	A	Accurate

Marker6676	Chr. 9	33667676	C	A	C	A	C	Accurate
Marker6677	Chr. 9	33808339	C	A	C	A	C	Accurate
Marker6678	Chr. 9	33808729	G	A	G	A	G	Accurate
Marker6681	Chr. 9	33928536	G	A	G	A	G	Accurate
Marker6683	Chr. 9	33941743	G	C	G	C	G	Accurate
Marker6685	Chr. 9	33970465	T	C	T	C	T	Accurate
Marker6688	Chr. 9	34007080	G	T	G	T	G	Accurate
Marker6689	Chr. 9	34049290	C	T	C	T	C	Accurate
Marker6692	Chr. 9	34112669	G	A	G	A	G	Accurate
Marker6694	Chr. 9	34186458	A	G	A	G	A	Accurate
Marker6695	Chr. 9	34209755	G	A	G	A	G	Accurate
Marker6699	Chr. 9	34381331	A	C	A	C	A	Accurate
Marker6703	Chr. 9	34475533	C	A	C	A	C	Accurate
Marker6704	Chr. 9	34703803	C	T	C	T	C	Accurate
Marker6705	Chr. 9	34763289	T	C	T	C	T	Accurate
Marker6709	Chr. 9	34892665	T	C	T	C	T	Accurate
Marker6711	Chr. 9	34923330	A	G	A	G	A	Accurate
Marker6713	Chr. 9	34956310	T	C	T	C	T	Accurate
Marker6720	Chr. 1(	1051066	A	G	A	G	A	Accurate
Marker6721	Chr. 1(	1057893	T	G	T	G	T	Accurate
Marker6723	Chr. 1(	1082828	T	C	T	C	T	Accurate
Marker6724	Chr. 1(	1083106	G	T	G	T	G	Accurate
Marker6725	Chr. 1(	1121873	G	G	A	G	A	Accurate
Marker6729	Chr. 1(	1143972	G	G	T	G	T	Accurate
Marker6734	Chr. 1(	1272471	G	A	G	A	G	Accurate
Marker6737	Chr. 1(	1296804	A	G	A	G	A	Accurate
Marker6738	Chr. 1(	1296821	A	C	A	C	A	Accurate
Marker6740	Chr. 1(	1297366	A	G	A	G	A	Accurate
Marker6742	Chr. 1(	1298079	A	G	A	G	A	Accurate
Marker6743	Chr. 1(	1298098	T	C	T	C	T	Accurate
Marker6745	Chr. 1(	1301945	C	T	C	T	C	Accurate
Marker6746	Chr. 1(	1312697	A	T	A	T	A	Accurate
Marker6748	Chr. 1(	1322395	C	T	C	T	C	Accurate
Marker6750	Chr. 1(	1328501	G	A	G	A	G	Accurate
Marker6751	Chr. 1(	1328540	T	C	T	C	T	Accurate
Marker6752	Chr. 1(	1332759	A	T	A	T	A	Accurate
Marker6755	Chr. 1(	1352365	T	C	T	C	T	Accurate
Marker6757	Chr. 1(	1361535	A	G	A	G	A	Accurate
Marker6763	Chr. 1(	1874821	A	G	A	G	A	Accurate
Marker6764	Chr. 1(	1875539	C	T	C	T	C	Accurate
Marker6769	Chr. 1(	1961744	T	C	T	C	T	Accurate
Marker6770	Chr. 1(	1961748	A	G	A	G	A	Accurate
Marker6773	Chr. 1(	2034012	C	A	C	A	C	Accurate
Marker6778	Chr. 1(	2067580	A	T	A	T	A	Accurate
Marker6779	Chr. 1(	2067581	C	T	C	T	C	Accurate
Marker6781	Chr. 1(	2081802	C	T	C	T	C	Accurate
Marker6785	Chr. 1(	2362441	G	G	A	G	A	Accurate

Marker6786	Chr. 1(	2390993	G	G	A	G	A	Accurate
Marker6790	Chr. 1(	2441170	A	A	G	A	G	Accurate
Marker6796	Chr. 1(	2580221	A	A	T	A	T	Accurate
Marker6797	Chr. 1(	2831314	G	A	G	A	G	Accurate
Marker6805	Chr. 1(	2978301	A	G	A	G	A	Accurate
Marker6806	Chr. 1(	2978587	C	A	C	A	C	Accurate
Marker6809	Chr. 1(	3688665	A	G	A	G	A	Accurate
Marker6810	Chr. 1(	3728879	C	T	C	T	C	Accurate
Marker6811	Chr. 1(	3729155	G	A	G	A	G	Accurate
Marker6812	Chr. 1(	3729158	A	G	A	G	A	Accurate
Marker6813	Chr. 1(	3729186	T	G	T	G	T	Accurate
Marker6814	Chr. 1(	3729199	T	A	T	A	T	Accurate
Marker6815	Chr. 1(	3750462	G	T	G	T	G	Accurate
Marker6816	Chr. 1(	3750639	T	A	T	A	T	Accurate
Marker6817	Chr. 1(	3750664	A	C	A	C	A	Accurate
Marker6820	Chr. 1(	3788928	A	T	A	T	A	Accurate
Marker6825	Chr. 1(	4029267	G	A	G	A	G	Accurate
Marker6830	Chr. 1(	4050022	T	C	T	C	T	Accurate
Marker6834	Chr. 1(	4079280	T	C	T	C	T	Accurate
Marker6835	Chr. 1(	4079281	C	T	C	T	C	Accurate
Marker6840	Chr. 1(	4882048	T	C	T	C	T	Accurate
Marker6841	Chr. 1(	4885796	A	T	A	T	A	Accurate
Marker6842	Chr. 1(	4885946	A	G	A	G	A	Accurate
Marker6846	Chr. 1(	4970113	C	T	C	T	C	Accurate
Marker6848	Chr. 1(	4991847	T	C	T	C	T	Accurate
Marker6849	Chr. 1(	4995664	T	C	T	C	T	Accurate
Marker6850	Chr. 1(	4995958	A	T	A	T	A	Accurate
Marker6855	Chr. 1(	5656697	C	G	C	G	C	Accurate
Marker6856	Chr. 1(	5656833	C	T	C	T	C	Accurate
Marker6862	Chr. 1(	8771731	A	A	C	N	C	Uncertain
Marker6864	Chr. 1(	12196792	A	T	A	N	T	Inconsistent
Marker6868	Chr. 1(	13590769	C	T	C	T	C	Accurate
Marker6869	Chr. 1(	13600619	T	G	T	G	T	Accurate
Marker6882	Chr. 1(	13732168	G	A	G	A	G	Accurate
Marker6884	Chr. 1(	13732489	G	A	G	A	G	Accurate
Marker6885	Chr. 1(	13732507	A	G	A	G	A	Accurate
Marker6886	Chr. 1(	13744678	A	G	A	G	A	Accurate
Marker6889	Chr. 1(	13769648	G	T	G	T	G	Accurate
Marker6890	Chr. 1(	14418362	C	C	T	C	T	Accurate
Marker6891	Chr. 1(	14455475	A	A	G	A	G	Accurate
Marker6892	Chr. 1(	14456512	A	A	G	A	G	Accurate
Marker6893	Chr. 1(	14457116	A	A	G	A	G	Accurate
Marker6894	Chr. 1(	14463242	C	C	A	C	A	Accurate
Marker6897	Chr. 1(	14483492	C	C	T	C	T	Accurate
Marker6898	Chr. 1(	14500777	C	C	T	C	T	Accurate
Marker6899	Chr. 1(	14511563	G	G	A	G	A	Accurate
Marker6900	Chr. 1(	14513672	G	G	A	G	A	Accurate

Marker6901	Chr. 10	14535608	C	C	A	C	A	Accurate
Marker6904	Chr. 10	16801671	T	C	T	C	T	Accurate
Marker6905	Chr. 10	17273302	G	A	G	A	G	Accurate
Marker6910	Chr. 10	17331321	C	G	C	G	C	Accurate
Marker6912	Chr. 10	17448081	C	T	C	T	C	Accurate
Marker6914	Chr. 10	17478065	A	C	A	C	A	Accurate
Marker6915	Chr. 10	17518173	G	T	G	T	G	Accurate
Marker6917	Chr. 10	17535598	G	A	G	A	G	Accurate
Marker6918	Chr. 10	17541413	T	C	T	C	T	Accurate
Marker6920	Chr. 10	17545668	C	T	C	T	C	Accurate
Marker6921	Chr. 10	17545700	A	G	A	G	A	Accurate
Marker6922	Chr. 10	17562319	C	A	C	A	C	Accurate
Marker6926	Chr. 10	17567979	T	C	T	C	T	Accurate
Marker6927	Chr. 10	17567989	A	G	A	G	A	Accurate
Marker6928	Chr. 10	17568009	T	C	T	C	T	Accurate
Marker6930	Chr. 10	17786938	G	A	G	A	G	Accurate
Marker6931	Chr. 10	17791256	G	A	G	A	G	Accurate
Marker6933	Chr. 10	17874022	G	A	G	A	G	Accurate
Marker6935	Chr. 10	18103179	A	G	A	G	A	Accurate
Marker6936	Chr. 10	18104132	G	T	G	T	G	Accurate
Marker6938	Chr. 10	18106739	A	T	A	T	A	Accurate
Marker6940	Chr. 10	18290094	A	G	A	G	A	Accurate
Marker6941	Chr. 10	18378285	A	G	A	G	A	Accurate
Marker6944	Chr. 10	18503947	C	T	C	T	C	Accurate
Marker6945	Chr. 10	18535055	G	A	G	A	G	Accurate
Marker6946	Chr. 10	18535427	G	A	G	A	G	Accurate
Marker6950	Chr. 10	18564831	C	T	C	T	C	Accurate
Marker6951	Chr. 10	18575821	C	T	C	T	C	Accurate
Marker6953	Chr. 10	19519393	T	C	T	C	T	Accurate
Marker6955	Chr. 10	19876182	C	T	C	T	C	Accurate
Marker6957	Chr. 10	19878665	G	T	G	T	G	Accurate
Marker6959	Chr. 10	19916163	C	T	C	T	C	Accurate
Marker6962	Chr. 10	19996660	A	C	A	C	A	Accurate
Marker6963	Chr. 10	20060326	G	C	G	C	G	Accurate
Marker6964	Chr. 10	20064184	A	G	A	G	A	Accurate
Marker6968	Chr. 10	20087348	T	A	T	A	T	Accurate
Marker6969	Chr. 10	20087612	G	A	G	A	G	Accurate
Marker6977	Chr. 10	20229405	A	C	A	C	A	Accurate
Marker6978	Chr. 10	20229568	C	G	C	G	C	Accurate
Marker6979	Chr. 10	20232613	A	G	A	G	A	Accurate
Marker6984	Chr. 10	20244337	T	C	T	C	T	Accurate
Marker6985	Chr. 10	21989369	A	T	A	T	A	Accurate
Marker6986	Chr. 10	21990378	A	G	A	G	A	Accurate
Marker6987	Chr. 10	21990577	A	A	C	A	C	Accurate
Marker6990	Chr. 10	22030751	G	T	G	T	G	Accurate
Marker6991	Chr. 10	22041640	G	T	G	T	G	Accurate
Marker6998	Chr. 10	22194247	T	T	A	T	A	Accurate

Marker6999	Chr. 1(	22194252	G	G	T	G	T	Accurate
Marker7000	Chr. 1(	22195113	T	T	C	T	C	Accurate
Marker7004	Chr. 1(	22259526	G	T	G	T	G	Accurate
Marker7006	Chr. 1(	22264112	C	A	C	A	C	Accurate
Marker7007	Chr. 1(	22264264	C	T	C	T	C	Accurate
Marker7009	Chr. 1(	22345539	T	T	C	T	C	Accurate
Marker7010	Chr. 1(	22352763	A	A	T	A	T	Accurate
Marker7012	Chr. 1(	22381981	G	G	C	G	C	Accurate
Marker7014	Chr. 1(	22416637	C	T	C	T	C	Accurate
Marker7015	Chr. 1(	22430948	C	C	T	C	T	Accurate
Marker7017	Chr. 1(	22442469	T	C	T	C	T	Accurate
Marker7018	Chr. 1(	22442471	C	A	C	A	C	Accurate
Marker7019	Chr. 1(	22442722	C	T	C	T	C	Accurate
Marker7020	Chr. 1(	22442770	G	A	G	A	G	Accurate
Marker7023	Chr. 1(	23143869	G	G	C	G	C	Accurate
Marker7026	Chr. 1(	23504408	C	C	G	C	G	Accurate
Marker7027	Chr. 1(	23527242	G	A	G	A	G	Accurate
Marker7028	Chr. 1(	23545482	A	A	C	N	C	Uncertain
Marker7029	Chr. 1(	23549626	T	T	G	T	G	Accurate
Marker7030	Chr. 1(	23897722	C	C	T	C	T	Accurate
Marker7032	Chr. 1(	24082693	C	C	T	C	T	Accurate
Marker7033	Chr. 1(	24103064	G	A	G	A	G	Accurate
Marker7040	Chr. 1(	24619009	G	A	G	A	G	Accurate
Marker7041	Chr. 1(	24639842	T	T	C	N	C	Uncertain
Marker7042	Chr. 1(	24641252	G	G	A	G	A	Accurate
Marker7044	Chr. 1(	24821477	A	G	A	G	A	Accurate
Marker7045	Chr. 1(	25158973	C	T	C	T	C	Accurate
Marker7046	Chr. 1(	25159531	C	T	C	T	C	Accurate
Marker7048	Chr. 1(	25187106	C	A	C	A	C	Accurate
Marker7050	Chr. 1(	25188128	C	G	C	G	C	Accurate
Marker7051	Chr. 1(	25188832	C	A	C	A	C	Accurate
Marker7052	Chr. 1(	25333429	C	T	C	T	C	Accurate
Marker7055	Chr. 1(	26472883	T	T	C	T	C	Accurate
Marker7056	Chr. 1(	26517282	C	C	A	C	A	Accurate
Marker7058	Chr. 1(	26557877	T	T	G	T	G	Accurate
Marker7062	Chr. 1(	26669383	C	C	T	C	T	Accurate
Marker7064	Chr. 1(	26678023	T	T	A	T	A	Accurate
Marker7065	Chr. 1(	26678237	A	A	G	A	G	Accurate
Marker7068	Chr. 1(	26690334	A	A	G	A	G	Accurate
Marker7069	Chr. 1(	26800394	G	T	G	T	G	Accurate
Marker7070	Chr. 1(	26831276	G	A	G	A	G	Accurate
Marker7071	Chr. 1(	26885694	A	A	G	A	G	Accurate
Marker7072	Chr. 1(	26885928	C	C	G	C	G	Accurate
Marker7075	Chr. 1(	26951727	G	A	G	A	G	Accurate
Marker7076	Chr. 1(	26955198	G	A	G	A	G	Accurate
Marker7077	Chr. 1(	26960947	A	G	A	G	A	Accurate
Marker7078	Chr. 1(	26960993	T	G	T	G	T	Accurate

Marker7079	Chr. 1(	26961003	A	G	A	G	A	Accurate
Marker7080	Chr. 1(	26969874	A	G	A	G	A	Accurate
Marker7083	Chr. 1(	26974404	C	T	C	T	C	Accurate
Marker7085	Chr. 1(	26982347	T	C	T	C	T	Accurate
Marker7086	Chr. 1(	26988162	C	T	C	T	C	Accurate
Marker7088	Chr. 1(	27055823	G	G	A	G	A	Accurate
Marker7089	Chr. 1(	27055983	A	A	G	A	G	Accurate
Marker7090	Chr. 1(	27057974	G	G	A	G	A	Accurate
Marker7092	Chr. 1(	27062350	A	A	G	A	G	Accurate
Marker7096	Chr. 1(	27151606	G	A	G	A	G	Accurate
Marker7097	Chr. 1(	27172406	T	A	T	A	T	Accurate
Marker7098	Chr. 1(	27172422	C	T	C	T	C	Accurate
Marker7102	Chr. 1(	27777455	C	T	C	T	C	Accurate
Marker7108	Chr. 11	728764	G	A	G	A	G	Accurate
Marker7109	Chr. 11	728871	C	T	C	T	C	Accurate
Marker7110	Chr. 11	731064	A	G	A	G	A	Accurate
Marker7111	Chr. 11	736761	A	G	A	G	A	Accurate
Marker7113	Chr. 11	762368	T	A	T	A	T	Accurate
Marker7115	Chr. 11	788396	G	C	G	C	G	Accurate
Marker7116	Chr. 11	788408	A	G	A	G	A	Accurate
Marker7122	Chr. 11	850712	T	C	T	C	T	Accurate
Marker7124	Chr. 11	890282	G	A	G	A	G	Accurate
Marker7128	Chr. 11	1119740	A	G	A	G	A	Accurate
Marker7130	Chr. 11	1271222	T	C	T	C	T	Accurate
Marker7131	Chr. 11	1279581	A	C	A	C	A	Accurate
Marker7132	Chr. 11	1279738	C	G	C	G	C	Accurate
Marker7137	Chr. 11	1411020	T	A	T	A	T	Accurate
Marker7138	Chr. 11	1411023	T	A	T	A	T	Accurate
Marker7139	Chr. 11	1411026	T	A	T	A	T	Accurate
Marker7140	Chr. 11	1421262	T	C	T	C	T	Accurate
Marker7141	Chr. 11	1421266	G	T	G	T	G	Accurate
Marker7144	Chr. 11	1625962	A	C	A	C	A	Accurate
Marker7145	Chr. 11	1643594	T	A	T	A	T	Accurate
Marker7149	Chr. 11	2140360	C	T	C	T	C	Accurate
Marker7154	Chr. 11	2586701	C	T	C	T	C	Accurate
Marker7156	Chr. 11	2587214	G	A	G	A	G	Accurate
Marker7157	Chr. 11	2592607	G	T	G	T	G	Accurate
Marker7158	Chr. 11	2628242	C	T	C	T	C	Accurate
Marker7159	Chr. 11	2660667	G	C	G	C	G	Accurate
Marker7162	Chr. 11	2699534	G	A	G	A	G	Accurate
Marker7164	Chr. 11	2713583	G	A	G	A	G	Accurate
Marker7167	Chr. 11	2741902	C	T	C	T	C	Accurate
Marker7168	Chr. 11	2741958	T	G	T	G	T	Accurate
Marker7170	Chr. 11	2794456	C	T	C	T	C	Accurate
Marker7171	Chr. 11	2806843	T	C	T	C	T	Accurate
Marker7172	Chr. 11	2815198	G	A	G	A	G	Accurate
Marker7174	Chr. 11	2896816	G	G	A	G	A	Accurate

Marker7175	Chr. 11	2918864	A	A	T	N	T	Uncertain
Marker7176	Chr. 11	2918884	T	T	C	N	C	Uncertain
Marker7179	Chr. 11	2948736	G	G	C	G	C	Accurate
Marker7180	Chr. 11	3032102	G	G	A	G	A	Accurate
Marker7182	Chr. 11	3142578	A	G	A	G	A	Accurate
Marker7183	Chr. 11	3143173	C	T	C	T	C	Accurate
Marker7184	Chr. 11	3151526	G	A	G	A	G	Accurate
Marker7185	Chr. 11	3166133	C	T	C	T	C	Accurate
Marker7186	Chr. 11	3179903	A	C	A	C	A	Accurate
Marker7187	Chr. 11	3180074	T	C	T	C	T	Accurate
Marker7188	Chr. 11	3181601	C	T	C	T	C	Accurate
Marker7189	Chr. 11	3181814	T	C	T	C	T	Accurate
Marker7190	Chr. 11	3331743	T	A	T	A	T	Accurate
Marker7193	Chr. 11	3384818	C	T	C	T	C	Accurate
Marker7194	Chr. 11	3384869	A	G	A	G	A	Accurate
Marker7195	Chr. 11	3386010	C	T	C	T	C	Accurate
Marker7196	Chr. 11	3386028	C	A	C	A	C	Accurate
Marker7197	Chr. 11	3386029	C	T	C	T	C	Accurate
Marker7198	Chr. 11	3395483	T	C	T	C	T	Accurate
Marker7199	Chr. 11	3395718	T	C	T	C	T	Accurate
Marker7200	Chr. 11	3400295	G	A	G	A	G	Accurate
Marker7201	Chr. 11	3400627	G	A	G	A	G	Accurate
Marker7202	Chr. 11	3496517	C	C	T	C	T	Accurate
Marker7204	Chr. 11	3642394	T	C	T	C	T	Accurate
Marker7205	Chr. 11	3650837	A	C	A	C	A	Accurate
Marker7206	Chr. 11	3675497	T	G	T	G	T	Accurate
Marker7208	Chr. 11	3685167	A	G	A	G	A	Accurate
Marker7212	Chr. 11	3737807	G	A	G	A	G	Accurate
Marker7213	Chr. 11	3738040	A	G	A	G	A	Accurate
Marker7214	Chr. 11	3738046	A	G	A	G	A	Accurate
Marker7215	Chr. 11	3738047	T	G	T	G	T	Accurate
Marker7216	Chr. 11	3739915	C	T	C	T	C	Accurate
Marker7217	Chr. 11	3739941	T	C	T	C	T	Accurate
Marker7218	Chr. 11	3740105	T	G	T	G	T	Accurate
Marker7219	Chr. 11	3740191	T	G	T	G	T	Accurate
Marker7220	Chr. 11	3740199	T	C	T	C	T	Accurate
Marker7222	Chr. 11	3767842	A	A	T	A	T	Accurate
Marker7223	Chr. 11	3768099	G	G	A	G	A	Accurate
Marker7227	Chr. 11	3826662	C	A	C	A	N	Uncertain
Marker7228	Chr. 11	3830420	T	C	T	C	T	Accurate
Marker7230	Chr. 11	3884711	G	C	G	C	G	Accurate
Marker7232	Chr. 11	3989481	C	T	C	T	C	Accurate
Marker7234	Chr. 11	4019458	G	T	G	T	G	Accurate
Marker7235	Chr. 11	4019514	C	T	C	T	C	Accurate
Marker7236	Chr. 11	4039057	T	A	T	A	T	Accurate
Marker7241	Chr. 11	4162942	G	A	G	A	G	Accurate
Marker7242	Chr. 11	4162971	C	T	C	T	C	Accurate

Marker7243	Chr. 11	4163157	G	A	G	A	G	Accurate
Marker7244	Chr. 11	4163184	A	G	A	G	A	Accurate
Marker7247	Chr. 11	4239661	A	G	A	G	A	Accurate
Marker7248	Chr. 11	4239668	C	T	C	T	C	Accurate
Marker7250	Chr. 11	4259753	A	C	A	C	A	Accurate
Marker7252	Chr. 11	4263244	C	T	C	T	C	Accurate
Marker7254	Chr. 11	4266510	T	A	T	A	T	Accurate
Marker7255	Chr. 11	4267867	G	A	G	A	G	Accurate
Marker7256	Chr. 11	4327623	A	G	A	G	A	Accurate
Marker7259	Chr. 11	4386906	T	C	T	C	T	Accurate
Marker7260	Chr. 11	4386935	T	A	T	A	T	Accurate
Marker7261	Chr. 11	4390594	C	T	C	T	C	Accurate
Marker7265	Chr. 11	4472009	C	T	C	T	C	Accurate
Marker7266	Chr. 11	4477662	T	C	T	C	T	Accurate
Marker7267	Chr. 11	4482937	T	C	T	C	T	Accurate
Marker7268	Chr. 11	4521954	G	A	G	A	G	Accurate
Marker7271	Chr. 11	4585754	T	C	T	C	T	Accurate
Marker7272	Chr. 11	4677065	A	T	A	T	A	Accurate
Marker7273	Chr. 11	4781614	C	T	C	T	C	Accurate
Marker7275	Chr. 11	4785255	C	T	C	T	C	Accurate
Marker7276	Chr. 11	4785376	G	A	G	A	G	Accurate
Marker7277	Chr. 11	4789132	G	C	G	C	G	Accurate
Marker7278	Chr. 11	4795156	C	T	C	T	C	Accurate
Marker7279	Chr. 11	4827852	C	T	C	T	C	Accurate
Marker7280	Chr. 11	4845242	T	C	T	C	T	Accurate
Marker7281	Chr. 11	4868887	G	G	A	G	A	Accurate
Marker7285	Chr. 11	6131401	C	C	T	C	T	Accurate
Marker7286	Chr. 11	6353829	G	A	G	A	G	Accurate
Marker7287	Chr. 11	6396211	T	A	T	A	T	Accurate
Marker7288	Chr. 11	6437541	T	C	T	C	T	Accurate
Marker7291	Chr. 11	7125700	T	C	T	C	T	Accurate
Marker7292	Chr. 11	7132165	T	C	T	C	T	Accurate
Marker7293	Chr. 11	7152462	A	G	A	G	A	Accurate
Marker7294	Chr. 11	7153321	A	G	A	G	A	Accurate
Marker7295	Chr. 11	7192965	A	C	A	C	A	Accurate
Marker7298	Chr. 11	7290382	G	A	G	A	G	Accurate
Marker7300	Chr. 11	7316913	C	T	C	T	C	Accurate
Marker7301	Chr. 11	7379683	T	G	T	G	T	Accurate
Marker7303	Chr. 11	7424306	C	T	C	T	C	Accurate
Marker7304	Chr. 11	7458445	A	G	A	G	A	Accurate
Marker7305	Chr. 11	7458537	C	T	C	T	C	Accurate
Marker7306	Chr. 11	7468195	C	T	C	T	C	Accurate
Marker7307	Chr. 11	7468405	T	C	T	C	T	Accurate
Marker7308	Chr. 11	7512993	T	C	T	C	T	Accurate
Marker7310	Chr. 11	7575571	T	C	T	C	T	Accurate
Marker7311	Chr. 11	7600464	A	T	A	T	A	Accurate
Marker7312	Chr. 11	7602861	T	C	T	C	T	Accurate

Marker7314	Chr. 11	7656507	A	G	A	G	A	Accurate
Marker7316	Chr. 11	7720205	G	A	G	A	G	Accurate
Marker7317	Chr. 11	7725225	T	C	T	C	T	Accurate
Marker7318	Chr. 11	7725318	T	C	T	C	T	Accurate
Marker7319	Chr. 11	7753238	T	C	T	C	T	Accurate
Marker7324	Chr. 11	8736229	A	A	C	A	C	Accurate
Marker7328	Chr. 11	8895051	G	G	T	G	T	Accurate
Marker7329	Chr. 11	8898091	G	G	A	G	A	Accurate
Marker7330	Chr. 11	8898103	C	C	T	C	T	Accurate
Marker7332	Chr. 11	8901578	A	A	G	A	G	Accurate
Marker7333	Chr. 11	8901620	A	A	G	A	G	Accurate
Marker7337	Chr. 11	9029703	A	A	G	A	G	Accurate
Marker7338	Chr. 11	9664498	C	C	T	C	T	Accurate
Marker7339	Chr. 11	9691545	C	C	A	C	A	Accurate
Marker7340	Chr. 11	9700906	A	A	T	A	T	Accurate
Marker7341	Chr. 11	9712667	C	C	T	C	T	Accurate
Marker7342	Chr. 11	9712707	T	T	C	T	C	Accurate
Marker7343	Chr. 11	9715659	C	C	T	C	T	Accurate
Marker7344	Chr. 11	9715687	T	T	C	T	C	Accurate
Marker7345	Chr. 11	9720802	A	A	G	A	G	Accurate
Marker7346	Chr. 11	9723677	T	T	A	T	A	Accurate
Marker7347	Chr. 11	9732410	A	A	G	A	G	Accurate
Marker7348	Chr. 11	9732465	A	A	G	A	G	Accurate
Marker7349	Chr. 11	9753557	T	T	C	T	C	Accurate
Marker7350	Chr. 11	9756391	G	G	T	G	T	Accurate
Marker7351	Chr. 11	9762230	C	C	T	C	T	Accurate
Marker7352	Chr. 11	9884843	T	C	T	C	C	Inconsistent
Marker7353	Chr. 11	10122997	C	T	C	T	C	Accurate
Marker7358	Chr. 11	10416888	A	A	G	A	G	Accurate
Marker7360	Chr. 11	10416929	C	C	T	C	T	Accurate
Marker7361	Chr. 11	10417689	C	C	A	C	A	Accurate
Marker7363	Chr. 11	10419946	C	C	T	C	T	Accurate
Marker7364	Chr. 11	10472615	C	T	C	T	C	Accurate
Marker7366	Chr. 11	10652261	T	C	T	C	T	Accurate
Marker7368	Chr. 11	10872498	A	A	G	A	G	Accurate
Marker7369	Chr. 11	10881500	T	T	C	T	C	Accurate
Marker7370	Chr. 11	10959142	T	T	G	T	G	Accurate
Marker7371	Chr. 11	11143489	T	T	G	T	G	Accurate
Marker7372	Chr. 11	11146280	T	T	C	T	C	Accurate
Marker7373	Chr. 11	11148624	G	G	A	G	A	Accurate
Marker7374	Chr. 11	11153233	G	G	T	G	T	Accurate
Marker7375	Chr. 11	11153259	T	T	C	T	C	Accurate
Marker7376	Chr. 11	11155051	A	A	T	A	T	Accurate
Marker7377	Chr. 11	11160754	T	T	A	T	A	Accurate
Marker7378	Chr. 11	11170935	C	C	G	C	G	Accurate
Marker7379	Chr. 11	11174742	T	T	G	T	G	Accurate
Marker7380	Chr. 11	11185087	C	C	T	C	T	Accurate

Marker7381	Chr. 11	11195339	T	T	C	T	C	Accurate
Marker7382	Chr. 11	11199592	G	G	A	G	A	Accurate
Marker7383	Chr. 11	11199833	A	A	G	A	G	Accurate
Marker7384	Chr. 11	11199839	C	C	A	C	A	Accurate
Marker7385	Chr. 11	11200133	T	T	C	T	C	Accurate
Marker7386	Chr. 11	11207112	A	T	C	N	T	Inconsistent
Marker7387	Chr. 11	11208802	A	A	G	A	G	Accurate
Marker7388	Chr. 11	11244170	C	C	T	C	T	Accurate
Marker7389	Chr. 11	11259784	T	T	C	T	C	Accurate
Marker7390	Chr. 11	11259797	G	G	A	G	A	Accurate
Marker7391	Chr. 11	11262275	A	A	G	A	G	Accurate
Marker7392	Chr. 11	11283824	T	T	C	T	C	Accurate
Marker7393	Chr. 11	11283865	G	G	A	G	A	Accurate
Marker7394	Chr. 11	11283993	T	T	C	T	C	Accurate
Marker7395	Chr. 11	11336958	T	T	C	T	C	Accurate
Marker7396	Chr. 11	11343404	T	T	G	T	G	Accurate
Marker7397	Chr. 11	11344244	C	C	A	C	A	Accurate
Marker7398	Chr. 11	11344269	T	T	A	T	A	Accurate
Marker7399	Chr. 11	11356948	G	G	T	G	T	Accurate
Marker7400	Chr. 11	11363390	C	C	T	C	T	Accurate
Marker7401	Chr. 11	11364255	G	G	A	G	A	Accurate
Marker7402	Chr. 11	11364281	T	T	C	T	C	Accurate
Marker7403	Chr. 11	11364349	T	T	C	T	C	Accurate
Marker7404	Chr. 11	11371667	T	T	C	T	C	Accurate
Marker7405	Chr. 11	11371934	C	C	T	C	T	Accurate
Marker7406	Chr. 11	11376875	T	T	C	T	C	Accurate
Marker7407	Chr. 11	11419154	C	C	T	C	T	Accurate
Marker7408	Chr. 11	11419409	G	G	A	G	A	Accurate
Marker7409	Chr. 11	11427485	G	G	A	G	A	Accurate
Marker7410	Chr. 11	11466971	A	A	G	A	G	Accurate
Marker7411	Chr. 11	11474576	A	A	G	A	G	Accurate
Marker7412	Chr. 11	11501086	A	A	G	A	G	Accurate
Marker7413	Chr. 11	11553211	G	G	A	G	A	Accurate
Marker7414	Chr. 11	11553364	A	A	C	A	C	Accurate
Marker7415	Chr. 11	11561123	A	A	G	A	G	Accurate
Marker7416	Chr. 11	11571820	A	A	G	A	G	Accurate
Marker7417	Chr. 11	11572081	G	G	T	G	T	Accurate
Marker7418	Chr. 11	11572332	G	G	A	G	A	Accurate
Marker7419	Chr. 11	11591929	C	C	T	C	T	Accurate
Marker7420	Chr. 11	11592140	A	A	G	A	G	Accurate
Marker7421	Chr. 11	11956083	A	A	G	A	G	Accurate
Marker7422	Chr. 11	11977084	G	G	A	G	A	Accurate
Marker7423	Chr. 11	11977238	A	A	G	A	G	Accurate
Marker7425	Chr. 11	12009114	A	A	G	A	G	Accurate
Marker7426	Chr. 11	12051519	A	A	T	A	T	Accurate
Marker7427	Chr. 11	12051770	C	C	G	C	G	Accurate
Marker7428	Chr. 11	12060416	G	G	C	G	C	Accurate

Marker7431	Chr. 11	12182719	A	A	G	A	G	Accurate
Marker7432	Chr. 11	12203405	T	T	C	T	C	Accurate
Marker7433	Chr. 11	12204105	A	A	G	A	G	Accurate
Marker7434	Chr. 11	12204355	G	G	A	G	A	Accurate
Marker7435	Chr. 11	12205350	T	T	C	T	C	Accurate
Marker7437	Chr. 11	12210535	T	T	C	T	C	Accurate
Marker7438	Chr. 11	12220813	A	A	G	A	G	Accurate
Marker7439	Chr. 11	12238271	T	T	A	T	A	Accurate
Marker7440	Chr. 11	12521727	A	A	G	A	G	Accurate
Marker7441	Chr. 11	12528998	A	A	G	A	G	Accurate
Marker7442	Chr. 11	12529004	A	A	G	A	G	Accurate
Marker7443	Chr. 11	12529196	A	A	T	A	T	Accurate
Marker7449	Chr. 11	12630568	C	C	T	Y	T	Uncertain
Marker7450	Chr. 11	12674592	G	G	C	G	C	Accurate
Marker7451	Chr. 11	12686098	T	T	C	T	C	Accurate
Marker7454	Chr. 11	12727574	C	C	T	C	T	Accurate
Marker7456	Chr. 11	12787443	A	A	T	A	T	Accurate
Marker7457	Chr. 11	12804691	C	C	T	C	T	Accurate
Marker7459	Chr. 11	12858933	T	T	C	T	C	Accurate
Marker7460	Chr. 11	12899708	A	A	C	A	C	Accurate
Marker7461	Chr. 11	13166347	C	T	C	T	C	Accurate
Marker7462	Chr. 11	13214043	G	G	A	G	A	Accurate
Marker7463	Chr. 11	13302890	G	G	A	N	A	Uncertain
Marker7464	Chr. 11	13366440	T	T	C	T	C	Accurate
Marker7465	Chr. 11	13383867	A	G	A	G	A	Accurate
Marker7466	Chr. 11	13383869	A	G	A	G	A	Accurate
Marker7467	Chr. 11	13385112	G	A	G	A	G	Accurate
Marker7468	Chr. 11	13387113	A	C	A	C	A	Accurate
Marker7469	Chr. 11	13389241	T	C	T	C	N	Uncertain
Marker7470	Chr. 11	13389528	A	G	A	G	A	Accurate
Marker7473	Chr. 11	13507299	C	C	T	C	T	Accurate
Marker7474	Chr. 11	13523541	A	A	G	A	G	Accurate
Marker7475	Chr. 11	13588562	C	C	A	C	A	Accurate
Marker7476	Chr. 11	13754694	C	T	C	T	C	Accurate
Marker7478	Chr. 11	13866185	A	G	A	G	A	Accurate
Marker7479	Chr. 11	13866430	A	T	A	T	A	Accurate
Marker7481	Chr. 11	13959932	A	T	A	T	N	Uncertain
Marker7482	Chr. 11	13961548	T	G	T	G	T	Accurate
Marker7487	Chr. 11	14384501	G	A	G	A	N	Uncertain
Marker7489	Chr. 11	14384778	G	A	G	A	G	Accurate
Marker7490	Chr. 11	14389585	A	G	A	G	A	Accurate
Marker7500	Chr. 11	14882851	G	G	A	G	A	Accurate
Marker7501	Chr. 11	15259610	G	G	C	G	C	Accurate
Marker7502	Chr. 11	15259783	G	G	A	G	A	Accurate
Marker7505	Chr. 11	15513579	C	C	A	C	A	Accurate
Marker7510	Chr. 11	15576394	A	A	G	A	G	Accurate
Marker7512	Chr. 11	15594922	A	C	A	C	A	Accurate

Marker7513	Chr. 11	15595107	A	G	A	G	A	Accurate
Marker7514	Chr. 11	15595180	C	T	C	T	C	Accurate
Marker7517	Chr. 11	15615428	T	C	T	C	T	Accurate
Marker7518	Chr. 11	15615732	G	C	G	C	G	Accurate
Marker7520	Chr. 11	15649548	T	T	G	T	G	Accurate
Marker7521	Chr. 11	15719157	T	T	A	T	A	Accurate
Marker7522	Chr. 11	15767392	T	T	A	T	A	Accurate
Marker7523	Chr. 11	15770956	T	T	A	T	A	Accurate
Marker7524	Chr. 11	15802596	C	C	G	N	G	Uncertain
Marker7527	Chr. 11	15811846	G	G	A	G	A	Accurate
Marker7531	Chr. 11	15844887	C	C	T	C	T	Accurate
Marker7535	Chr. 11	15890553	A	G	A	G	A	Accurate
Marker7536	Chr. 11	15890559	A	C	A	C	A	Accurate
Marker7537	Chr. 11	15894662	A	C	A	C	A	Accurate
Marker7538	Chr. 11	15901657	A	C	A	C	A	Accurate
Marker7540	Chr. 11	15929046	G	A	G	A	G	Accurate
Marker7541	Chr. 11	15929112	A	C	A	C	A	Accurate
Marker7542	Chr. 11	15952998	G	T	G	T	G	Accurate
Marker7546	Chr. 11	15993937	A	A	T	N	T	Uncertain
Marker7547	Chr. 11	15994195	C	C	A	C	A	Accurate
Marker7548	Chr. 11	16003022	C	T	C	T	C	Accurate
Marker7554	Chr. 11	16609761	T	C	T	C	T	Accurate
Marker7556	Chr. 11	16965042	G	A	G	A	G	Accurate
Marker7557	Chr. 11	16965075	C	G	C	G	C	Accurate
Marker7558	Chr. 11	16982952	G	A	G	A	G	Accurate
Marker7561	Chr. 11	17003215	C	T	C	T	C	Accurate
Marker7562	Chr. 11	17003447	A	C	A	C	A	Accurate
Marker7569	Chr. 11	17005877	A	T	A	T	A	Accurate
Marker7570	Chr. 11	17005925	C	T	C	T	C	Accurate
Marker7571	Chr. 11	17645149	C	C	T	C	T	Accurate
Marker7572	Chr. 11	17665888	T	T	G	T	G	Accurate
Marker7573	Chr. 11	17688666	A	A	G	A	G	Accurate
Marker7574	Chr. 11	17706321	A	A	C	A	C	Accurate
Marker7579	Chr. 11	18508701	T	C	T	C	T	Accurate
Marker7581	Chr. 11	18601223	T	G	T	G	T	Accurate
Marker7582	Chr. 11	18639255	G	A	G	A	G	Accurate
Marker7583	Chr. 11	18639291	G	T	G	T	G	Accurate
Marker7587	Chr. 11	21158153	G	A	G	A	G	Accurate
Marker7588	Chr. 11	21158175	T	C	T	C	T	Accurate
Marker7589	Chr. 11	21169040	C	T	C	T	C	Accurate
Marker7592	Chr. 11	21192528	A	G	A	G	A	Accurate
Marker7593	Chr. 11	21201832	G	G	A	G	A	Accurate
Marker7594	Chr. 11	21202033	C	C	T	C	T	Accurate
Marker7595	Chr. 11	21202038	C	C	T	C	T	Accurate
Marker7596	Chr. 11	21230687	G	G	C	G	C	Accurate
Marker7597	Chr. 11	21230866	A	G	A	A	R	Inconsistent
Marker7598	Chr. 11	21232821	G	G	A	G	A	Accurate

Marker7599	Chr. 11	21233103	G	G	A	G	A	Accurate
Marker7600	Chr. 11	21233126	G	G	C	G	C	Accurate
Marker7601	Chr. 11	21237380	G	G	A	G	A	Accurate
Marker7602	Chr. 11	21237406	T	T	G	T	G	Accurate
Marker7603	Chr. 11	21248584	T	T	C	T	C	Accurate
Marker7604	Chr. 11	21261083	A	A	C	A	C	Accurate
Marker7605	Chr. 11	21298983	A	A	T	A	T	Accurate
Marker7606	Chr. 11	21333861	G	G	A	G	A	Accurate
Marker7608	Chr. 11	21799202	A	C	A	C	A	Accurate
Marker7609	Chr. 11	21822266	T	G	T	G	T	Accurate
Marker7610	Chr. 11	21826865	T	C	T	C	T	Accurate
Marker7611	Chr. 11	21827314	A	G	A	G	A	Accurate
Marker7612	Chr. 11	21827320	A	G	A	G	A	Accurate
Marker7614	Chr. 11	21846988	A	T	A	T	A	Accurate
Marker7615	Chr. 11	21848321	A	G	A	G	A	Accurate
Marker7618	Chr. 11	21854842	C	T	C	T	N	Uncertain
Marker7619	Chr. 11	21855293	G	A	G	A	G	Accurate
Marker7626	Chr. 11	23146172	A	G	A	G	A	Accurate
Marker7627	Chr. 11	23156818	A	T	A	T	A	Accurate
Marker7631	Chr. 11	23259431	C	T	C	T	N	Uncertain
Marker7632	Chr. 11	23263876	A	T	A	T	A	Accurate
Marker7633	Chr. 11	23314243	T	C	T	C	T	Accurate
Marker7637	Chr. 11	23404275	T	G	T	G	T	Accurate
Marker7638	Chr. 11	23418183	G	A	G	A	G	Accurate
Marker7639	Chr. 11	23421381	A	G	A	G	A	Accurate
Marker7644	Chr. 11	23512595	T	C	T	C	T	Accurate
Marker7645	Chr. 11	23512761	T	C	T	C	T	Accurate
Marker7648	Chr. 11	23562980	G	A	G	A	G	Accurate
Marker7653	Chr. 11	23642091	T	C	T	C	T	Accurate
Marker7655	Chr. 11	23645392	T	C	T	C	T	Accurate
Marker7658	Chr. 11	23667623	T	G	T	G	T	Accurate
Marker7670	Chr. 11	24288153	C	T	C	T	C	Accurate
Marker7672	Chr. 11	25220661	A	C	A	C	A	Accurate
Marker7677	Chr. 0	834330	T	C	T	C	T	Accurate
Marker7680	Chr. 0	864658	C	T	C	T	C	Accurate
Marker7681	Chr. 0	878280	T	A	T	A	T	Accurate
Marker7685	Chr. 0	882219	T	C	T	C	T	Accurate
Marker7686	Chr. 0	983297	T	G	T	G	T	Accurate
Marker7688	Chr. 0	987155	G	A	G	A	G	Accurate
Marker7690	Chr. 0	987676	G	A	G	A	G	Accurate
Marker7691	Chr. 0	1000953	C	C	T	C	T	Accurate
Marker7692	Chr. 0	1387216	C	T	C	T	C	Accurate
Marker7693	Chr. 0	1387248	C	T	C	T	C	Accurate
Marker7694	Chr. 0	1638285	C	G	C	S	C	Uncertain
Marker7696	Chr. 0	2280567	A	G	A	G	A	Accurate
Marker7697	Chr. 0	2324564	C	T	C	T	C	Accurate
Marker7699	Chr. 0	2466745	A	G	A	G	A	Accurate

Marker7701	Chr. 0	2751789	A	T	A	T	A	Accurate
Marker7703	Chr. 0	2763993	C	A	C	A	C	Accurate
Marker7704	Chr. 0	2772264	T	C	T	C	T	Accurate
Marker7706	Chr. 0	3554060	C	T	C	T	C	Accurate
Marker7707	Chr. 0	3554125	G	A	G	R	G	Uncertain
Marker7708	Chr. 0	3664088	A	G	A	G	A	Accurate
Marker7711	Chr. 0	4352224	T	C	T	C	T	Accurate
Marker7714	Chr. 0	4463624	C	T	C	T	C	Accurate
Marker7715	Chr. 0	4484253	A	G	A	G	A	Accurate
Marker7716	Chr. 0	4487392	T	C	T	C	T	Accurate
Marker7717	Chr. 0	4487686	A	G	A	G	A	Accurate
Marker7718	Chr. 0	4497589	C	T	C	T	C	Accurate
Marker7719	Chr. 0	4497888	G	A	G	A	G	Accurate
Marker7720	Chr. 0	4497901	T	C	T	C	T	Accurate
Marker7721	Chr. 0	4498318	T	C	T	C	T	Accurate
Marker7722	Chr. 0	4498344	T	C	T	C	T	Accurate
Marker7723	Chr. 0	4552823	A	G	A	G	A	Accurate
Marker7724	Chr. 0	4565340	G	A	G	A	G	Accurate
Marker7727	Chr. 0	4731502	C	A	G	C	T	Inconsistent
Marker7733	Chr. 0	5113381	A	G	A	G	A	Accurate
Marker7735	Chr. 0	5124356	A	G	A	G	A	Accurate
Marker7736	Chr. 0	5126545	A	C	A	C	A	Accurate
Marker7742	Chr. 0	5669308	C	C	T	C	T	Accurate
Marker7743	Chr. 0	5669625	G	G	A	G	A	Accurate
Marker7746	Chr. 0	5743508	A	A	G	A	G	Accurate
Marker7747	Chr. 0	5807933	A	A	G	A	G	Accurate
Marker7748	Chr. 0	5808814	G	G	A	G	A	Accurate
Marker7753	Chr. 0	6164236	T	A	T	A	T	Accurate
Marker7755	Chr. 0	6532250	T	C	T	C	T	Accurate
Marker7761	Chr. 0	6594770	T	C	T	C	T	Accurate
Marker7769	Chr. 0	7685109	T	C	T	C	T	Accurate
Marker7774	Chr. 0	8045556	G	A	G	A	G	Accurate
Marker7775	Chr. 0	8080644	C	T	C	T	C	Accurate
Marker7776	Chr. 0	8080682	A	G	A	G	A	Accurate
Marker7781	Chr. 0	8949327	A	G	A	G	A	Accurate
Marker7782	Chr. 0	8950028	T	C	T	C	T	Accurate
Marker7786	Chr. 0	9626438	T	C	T	C	T	Accurate
Marker7788	Chr. 0	9652102	T	G	T	G	T	Accurate
Marker7790	Chr. 0	9693779	G	A	G	A	G	Accurate
Marker7791	Chr. 0	9695127	G	A	G	A	G	Accurate
Marker7792	Chr. 0	9697440	C	T	C	T	C	Accurate
Marker7794	Chr. 0	9703617	G	T	G	T	G	Accurate
Marker7795	Chr. 0	9716713	G	A	G	A	G	Accurate
Marker7796	Chr. 0	9846367	G	A	G	A	G	Accurate
Marker7798	Chr. 0	10697624	G	A	G	A	G	Accurate
Marker7803	Chr. 0	11663376	A	G	A	G	A	Accurate
Marker7805	Chr. 0	11698515	C	C	T	C	T	Accurate

Marker7810	Chr. 0	12368345	T	C	T	C	T	Accurate
Marker7812	Chr. 0	12425912	C	A	C	A	C	Accurate
Marker7818	Chr. 0	12700223	G	A	G	A	G	Accurate
Marker7836	Chr. 0	13819280	C	T	C	T	Y	Uncertain
Marker7839	Chr. 0	14512053	T	C	T	C	T	Accurate
Marker7842	Chr. 0	14792469	T	C	T	C	T	Accurate
Marker7843	Chr. 0	14806303	C	T	C	T	C	Accurate
Marker7845	Chr. 0	14815676	A	G	A	G	A	Accurate
Marker7849	Chr. 0	14838289	A	G	A	G	A	Accurate
Marker7850	Chr. 0	14851532	T	C	T	C	T	Accurate
Marker7851	Chr. 0	14861847	T	C	T	C	T	Accurate
Marker7852	Chr. 0	14865071	A	G	A	G	A	Accurate
Marker7853	Chr. 0	14865103	T	C	T	C	T	Accurate
Marker7854	Chr. 0	14865116	C	T	C	T	C	Accurate
Marker7857	Chr. 0	14880051	G	A	G	A	G	Accurate
Marker7860	Chr. 0	14917108	G	A	G	A	G	Accurate
Marker7863	Chr. 0	14936088	A	T	A	T	A	Accurate
Marker7864	Chr. 0	14936327	G	C	G	C	G	Accurate
Marker7865	Chr. 0	14941641	A	G	A	G	A	Accurate
Marker7867	Chr. 0	14952364	A	G	A	G	A	Accurate
Marker7870	Chr. 0	15213096	G	A	G	A	G	Accurate
Marker7871	Chr. 0	15213108	T	G	T	G	T	Accurate
Marker7873	Chr. 0	15221729	C	A	C	A	C	Accurate
Marker7880	Chr. 0	16228371	T	C	T	C	T	Accurate
Marker7882	Chr. 0	16504383	A	G	A	G	R	Uncertain
Marker7884	Chr. 0	16530309	T	C	T	C	T	Accurate
Marker7885	Chr. 0	16530343	G	C	G	C	G	Accurate
Marker7886	Chr. 0	16536348	A	C	A	C	A	Accurate
Marker7888	Chr. 0	16783510	C	T	C	Y	C	Uncertain
Marker7889	Chr. 0	16784499	G	A	G	A	G	Accurate
Marker7893	Chr. 0	16796179	C	T	C	T	C	Accurate
Marker7894	Chr. 0	16796486	C	C	T	C	T	Accurate
Marker7895	Chr. 0	16838891	G	A	G	A	G	Accurate
Marker7896	Chr. 0	16838940	A	G	A	G	A	Accurate
Marker7899	Chr. 0	16872370	G	A	G	A	G	Accurate
Marker7903	Chr. 0	17224355	G	A	G	A	G	Accurate
Marker7904	Chr. 0	17224377	T	G	T	G	T	Accurate
Marker7906	Chr. 0	17604807	G	A	G	A	G	Accurate
Marker7908	Chr. 0	17633452	T	C	T	C	T	Accurate
Marker7909	Chr. 0	17633517	C	T	C	T	C	Accurate
Marker7912	Chr. 0	17637968	T	C	T	Y	T	Uncertain
Marker7915	Chr. 0	17798450	C	T	C	T	C	Accurate
Marker7916	Chr. 0	17798481	T	G	T	G	T	Accurate
Marker7917	Chr. 0	17802268	C	T	C	T	C	Accurate
Marker7918	Chr. 0	17802438	C	G	C	G	C	Accurate
Marker7920	Chr. 0	17811046	A	G	A	G	A	Accurate
Marker7921	Chr. 0	18172203	G	A	G	A	G	Accurate

Marker7922	Chr. 0	18176778	G	T	G	T	G	Accurate
Marker7923	Chr. 0	18192133	C	T	C	T	C	Accurate
Marker7924	Chr. 0	18517536	G	T	G	T	G	Accurate
Marker7926	Chr. 0	18525187	A	T	A	T	A	Accurate
Marker7927	Chr. 0	18526527	A	G	A	G	A	Accurate
Marker7928	Chr. 0	18571369	C	C	A	N	A	Uncertain
Marker7931	Chr. 0	18599419	A	G	A	G	A	Accurate
Marker7936	Chr. 0	18606074	C	C	A	C	A	Accurate
Marker7937	Chr. 0	18610149	A	A	G	A	G	Accurate
Marker7938	Chr. 0	18612132	T	T	C	T	C	Accurate
Marker7941	Chr. 0	18660668	G	G	A	G	A	Accurate
Marker7942	Chr. 0	18686469	T	T	G	T	G	Accurate
Marker7950	Chr. 0	18805079	G	T	G	T	G	Accurate
Marker7951	Chr. 0	18863229	T	T	A	T	A	Accurate
Marker7953	Chr. 0	18972570	G	A	G	A	N	Uncertain
Marker7954	Chr. 0	18975473	T	G	T	G	T	Accurate
Marker7957	Chr. 0	19917374	A	G	A	G	A	Accurate
Marker7958	Chr. 0	19931867	C	T	C	T	C	Accurate
Marker7959	Chr. 0	19943263	C	T	C	T	C	Accurate
Marker7961	Chr. 0	19959504	A	G	A	G	A	Accurate
Marker7962	Chr. 0	19959550	C	T	C	T	C	Accurate
Marker7963	Chr. 0	19990578	A	C	A	C	A	Accurate
Marker7964	Chr. 0	19990587	C	T	C	T	C	Accurate
Marker7967	Chr. 0	19991939	C	T	C	T	C	Accurate
Marker7969	Chr. 0	20240167	C	T	C	T	C	Accurate
Marker7970	Chr. 0	20245244	T	A	T	A	T	Accurate
Marker7972	Chr. 0	20250078	T	C	T	C	T	Accurate
Marker7974	Chr. 0	20410918	T	A	T	A	T	Accurate
Marker7976	Chr. 0	20411206	A	C	A	C	A	Accurate
Marker7977	Chr. 0	20411237	A	G	A	G	A	Accurate
Marker7978	Chr. 0	20425194	T	C	T	C	T	Accurate
Marker7979	Chr. 0	20452203	C	T	C	T	C	Accurate
Marker7980	Chr. 0	20459558	A	G	A	G	A	Accurate
Marker7981	Chr. 0	20496008	G	T	G	T	G	Accurate
Marker7982	Chr. 0	21021477	A	A	G	A	G	Accurate
Marker7987	Chr. 0	21203615	G	G	A	G	A	Accurate
Marker7989	Chr. 0	21248990	G	G	A	G	A	Accurate
Marker7991	Chr. 0	21282477	C	C	T	C	T	Accurate
Marker7992	Chr. 0	21284930	C	C	T	C	T	Accurate
Marker7993	Chr. 0	21313190	C	C	A	C	A	Accurate
Marker7994	Chr. 0	21377356	C	C	T	C	T	Accurate
Marker7996	Chr. 0	21400830	A	A	G	A	G	Accurate
Marker7999	Chr. 0	21408048	T	T	C	T	C	Accurate
Marker8002	Chr. 0	21665345	C	T	C	T	C	Accurate
Marker8003	Chr. 0	21813917	T	C	T	C	T	Accurate
Marker8004	Chr. 0	22040458	A	G	A	G	A	Accurate
Marker8005	Chr. 0	22101636	C	T	C	T	C	Accurate

Marker8006	Chr. 0	22130165	C	T	C	T	C	Accurate
Marker8007	Chr. 0	22178100	T	C	T	C	T	Accurate
Marker8008	Chr. 0	22181190	G	A	G	A	G	Accurate
Marker8012	Chr. 0	22517444	A	A	G	A	G	Accurate
Marker8015	Chr. 0	22627206	A	G	A	G	A	Accurate
Marker8023	Chr. 0	23702995	T	T	G	T	G	Accurate
Marker8024	Chr. 0	23703100	T	T	C	T	C	Accurate
Marker8025	Chr. 0	23706595	T	T	G	T	G	Accurate
Marker8026	Chr. 0	23711647	C	C	T	C	T	Accurate

[a]: N indicates unknow,R indicates A or G, Y indicates C or T, M indicates A or C, K indicates G or T, S indicates C or G, W indicates A or T.

Supplementary Table 3 Detailed information on the primer pairs.

Marker	F-primer (5' to 3')	R-primer ((5' to 3')	product (bp)	Length of indel (bp)	Enzyme	Chr	Position (bp)	REF	ALT	ZXG01478[a]	14CB11
indel11_S6	ACACTAGTATCACCGCATAC	ACTTTATGATTGAAATGGAG	292	42		Chr.6	5058957	TATTCCACCATTCTGACTGTAATACACTGAACAAAACCTTGTC	T	0/0	1/1
caps1_S6	AGTGGATGAACCATTAGCGA	ACAAGGACTACAATTGAACAAACT	366		DraI	Chr.6	5281878	C	T	0/0	1/1
indel13_S6	TGGTAGCAACATGGAAGAAG	AACTGCCAAGGTGAGAAAAC	176	18		Chr.6	5300529	GGAGAGAGAGAGAGAGAGA	G	1/1	0/0
dcaps6_S6	AATTATCTAAAACACCCAAGGTC	AACTTTCACTATTTTAACTCA	177		TaqI	Chr.6	5362844	G	A	1/1	0/0
dcaps7_S6	TAGAAGTATAAAGCAACCCC	GAAGCCGAAGAGATCAAATCAGAAGGTGC	134		HpyCH4V	Chr.6	5373351	T	C	1/1	0/0
dcaps9_S6	CTAAAAATACAGGATTAAAATTGTACATTC	TGTAAAACACATATATAACG	142		TaqI	Chr.6	5418365	G	A	1/1	0/0
indel14_S6	TGGTTGTTTCTTTTAGGTGGTA	AGCGATTATGAGCAATTTTA	238	46		Chr.6	5478024	CTAGCAAGAGCGTTACTACTTCAACTAAAAATATCCATTTCATTTTA	C	0/0	1/1
caps4_S6	ACAGAAAGACCAAGAGGATA	TTGATGAAATAAGAAAGGAC	419		BfaI	Chr.6	5540206	A	G	0/0	1/1
indel15_S6	CATCGAGGTAAGTAGTTTCT	GATTTGTCTGATGAAGTGGC	302	21		Chr.6	5580040	AAAAGAATTAAAATGATATTTT	A	0/0	1/1

caps5_S6	TTCAAGGCAATAGCAACCTC	CGTTAATACGCTTCCAGATC	443		AluI	Chr.6	5598954	G	T	0/0	1/1	
indel16_S6	AAACTTTATGGGATGAAGAT	AAATAAACTCACCTAGTTGC	345	50		Chr.6	5602653	T	TTTTTTTTTAAATTAAAGTTATAAACACCATTTTCATCTCTGAAACTCTTGC	0/0	1/1	
qRT-PCR for *Cla009289*	CACAGTCGATTCTCAAATCCCTA	AAACCACATATCAATACATCCCAC	167									
qRT-PCR for *Cla009290*	CCGGTCCGGTTTACATAACA	GAATTTCAACGTCGCCTTTT	126									
qRT-PCR for *Cla009291*	GGCGTTTGTTTGGAAATCTG	CCTTCCTCACGTATGGCTGT	198									
qRT-PCR for *Cla009292*	CGCCTCGATTACTGCTTCCT	GCCTGCACCTACACTCCACA	106									
Actin	ATTCTCCGTTTGGACCTTGCT	TCGTAGTTTTTCTCAATGGAGG	201									

[a]: 0/0 and 1/1 indicate the same sequence with REF and ALT, respectively.
